# Distribution patterns of *Quercus ilex* from the last interglacial period to the future by ecological niche modeling

**DOI:** 10.1002/ece3.10606

**Published:** 2023-10-19

**Authors:** Burak Suicmez, Meral Avci

**Affiliations:** ^1^ Istanbul University, Institute of Social Sciences Istanbul Türkiye; ^2^ Department of Geography, Faculty of Letters Istanbul University Istanbul Türkiye

**Keywords:** Anatolia, climate change, keystone species, Mediterranean Basin, paleovegetation, Quaternary

## Abstract

The plants' geographic distribution is affected by natural or human‐induced climate change. Numerous studies at both the global and regional levels currently focus on the potential changes in plant distribution areas. Ecological niche modeling can help predict the likely distribution of species according to environmental variables under different climate scenarios. In this study, we predicted the potential geographic distributions of *Quercus ilex* L. (holm oak), a keystone species of the Mediterranean ecosystem, for the Last Interglacial period (LIG: ~130 Ka), the Last Glacial Maximum (LGM: ~22 Ka), mid‐Holocene (MH: ~6 Ka), and future climate scenarios (Representative Concentration Pathway (RCP) 4.5 and 8.5 scenarios) for 2050–2070 obtained from CCSM4 and MIROC‐ESM global climate scenarios respectively. The models were produced with algorithms from the R‐package “biomod2” and assessed by AUC of the receiver operating characteristic plot and true skill statistics. Aside from BIOCLIM (SRE), all model algorithms performed similarly and produced projections that are supported by good evaluation scores, although random forest (RF) slightly outperformed all the others. Additionally, distribution maps generated for the past period were validated through a comparison with pollen data acquired from the Neotoma Pollen Database. The results revealed that southern areas of the Mediterranean Basin, particularly coastal regions, served as long‐term refugia for *Q. ilex*, which was supported by fossil pollen data. Furthermore, the models suggest long‐term refugia role for Anatolia and we argue that Anatolia may have served as a founding population for the species. Future climate scenarios indicated that *Q. ilex* distribution varied by region, with some areas experiencing range contractions and others range expands. This study provides significant insights into the vulnerability of the *Q. ilex* to future climate change in the Mediterranean ecosystem and highlights the crucial role of Anatolia in the species' historical distribution.

## Graphical abstract

Future climate scenarios indicated that *Q. ilex* distribution varied by region. This study provides significant insights on the vulnerability of a keystone species, *Q. ilex*, due to the future climate change in the Mediterranean ecosystem and highlights the crucial role of Anatolia in the species' historical distribution.

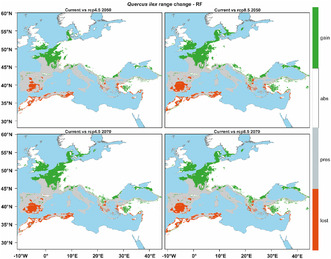

## 1 INTRODUCTION

The climate of the world has been in perpetual change, throughout the geological ages as a result of various internal and external dynamics. In particular, the Quaternary is characterized as a period in which the effects of climate changes were highly pronounced (Rull, 2020). The Pleistocene epoch was marked by recurring cycles of climatic oscillations between glacial and interglacial periods (Lisiecki & Raymo, 2007). Notably, the Last Interglacial period (LIG) is widely recognized as a period of relative warmth in comparison with the present day (Kukla et al., 2002). Conversely, the Last Glacial Maximum (LGM: 22 Ka) is marked by the widespread advance of large ice sheets across North America, northern Europe, and Asia, leading to significant drops in temperature and the emergence of arid environments (Hughes et al., 2013). Climate is widely recognized as one of the most significant environmental variables influencing the geographic distribution of species, including plants, leading to changes in habitat suitability, biotic interactions, and species physiology (Araújo & Rahbek, 2006; Bennett & Provan, 2008; Hewitt, 2000; Svenning & Skov, 2007). The glacial and interglacial periods of the Pleistocene resulted in significant shifts in the distribution of plant communities (Hampe & Jump, 2011), with the most influential changes occurring during the LGM. The LGM caused the range contraction of many temperate species toward suitable areas where the climate was favorable for tree survival, known as glacial refugia in southern Europe, the Iberian, Italian, and Balkan peninsulas (Bennett et al., 1991; Bhagwat & Willis, 2008; Hewitt, 1996, 1999; Svenning et al., 2008), areas forming the Mediterranean Basin, our research area. The Holocene period is characterized by the warming of the climate and the retreat of glaciers following the ice ages, leading to the reappearance of favorable conditions for animal and plant species adapted to temperate conditions. These species exhibited a tendency to expand their ranges northward from refuge areas and have recolonized (Hewitt, 1999). Due to climatic oscillations, refuge areas where the species persist during unfavorable conditions and recolonize stand out as rich genetic diversity areas (Badgley et al., 2017; Davis & Shaw, 2001; Hewitt, 2004). Anatolia's diverse topographical and geomorphological structure has contributed to its rich flora and the high rate of endemism among plant species (Avci, 2005; Médail & Diadema, 2009; Şekercioğlu et al., 2011). It encompasses three biodiversity hotspots (Mittermeier et al., 2004), including the Mediterranean (Caucasus, Iran‐Anatolia, and the Mediterranean), and is considered one of the possible origins of the European lineage of certain species (Rokas et al., 2003). In addition, numerous studies suggest the role of Anatolia, another peninsula in the Mediterranean Basin, as a refugia during the LGM (Bagnoli et al., 2016; Bilgin, 2011; Médail & Diadema, 2009; Schmitt, 2007; Tekpinar et al., 2021). The ecological processes observed during the LGM led to the rich and diverse plant diversity of the Mediterranean Basin, which plays the role of refugia for European flora. This heritage of plant diversity is still preserved in the Mediterranean Basin today (Médail & Diadema, 2009).

In recent times, the global climate has undergone substantial changes, primarily due to human influence, contrasting with the natural causes of climate change observed during the Pleistocene (IPCC, 2021). These changes have had a wide‐ranging impact on ecosystems, including the loss of biodiversity, alteration of ecosystem functions, and shifts in species distributions (Bellard et al., 2012; Parmesan & Yohe, 2003; Walther et al., 2002). The scale and the intensity of climate change are expected to increase in the near future, with potentially catastrophic consequences for numerous species and ecosystems (IPCC, 2021). Species must adapt in the face of climate change or move toward climate conditions suitable for them (Parmesan & Yohe, 2003). Species persist in their habitat via phenotypic plasticity and genetic adaptations which allow them to survive under new environmental conditions (Hoffmann & Sgrò, 2011; Waldvogel et al., 2020). Species that are unable to adjust to the changing climatic conditions and cannot migrate to areas with suitable climatic conditions are at high risk of extinction (Pacifici et al., 2015; Parmesan, 2006; Thomas et al., 2004; Walther et al., 2002). Due to the rapid pace of climate change, many species may be unable to adapt to the changing environmental conditions, which may ultimately lead to significant consequences for global biodiversity (Corlett & Westcott, 2013). The Mediterranean Basin is recognized as one of the regions that is most sensitive to climate change due to the substantial decrease in average precipitation and increase in precipitation variability during the dry season (Giorgi, 2006; Tramblay et al., 2020; Turkes et al., 2020). Projections indicate that by the end of the 21st century, there could be a 50% reduction in precipitation in some parts of Northwest Africa and a negative precipitation change of up to 20%–25% in the Iberian Peninsula, Balkan Peninsula, and a significant portion of Anatolia. Conversely, some regions in the northern flank of the Mediterranean Basin, such as northern Italy, Slovenia, and the Black Sea region, may experience a transition to “humid‐wet” conditions that are more prevalent in mid‐latitudes (Barcikowska et al., 2018). In addition to the impact of climate change observed around the Mediterranean Basin, human influence also threatens the richness of the Mediterranean Basin's flora (Guo et al., 2022), therefore the Mediterranean Basin is considered a biodiversity hotspot (Médail & Myers, 2004).

Ecological niche modeling can help predict the likely distribution of species based on occurrence data and environmental variables under different climate scenarios in the past, present, and future times (Elith & Leathwick, 2009; Guisan et al., 2013; Guisan & Zimmermann, 2000; Pearson & Dawson, 2003). Currently, numerous studies are conducted both globally and regionally focusing on predicting changes in the distribution of plant species across different time periods, including past and future scenarios. Knowledge of species range shifts as a result of climate change provides valuable information to make more accurate risk assessments and conservation management for the future, and ecological niche models are increasingly being used to support conservation decision making (Guisan & Thuiller, 2005). As a result, the Mediterranean flora has been extensively studied using ecological niche modeling approaches for predicting past and future distributions, with an increasing number of studies being conducted (Almeida et al., 2022; Benito Garzón et al., 2007; Fyllas et al., 2019; Salvà‐Catarineu et al., 2021).

*Quercus ilex* is a broad‐leaved evergreen tree or shrub that can grow well in a wide variety of soils in different Mediterranean climates, from semi‐arid to very humid and from hot to very cold, native to the central and western parts of the Mediterranean Basin, the Balkan regions, the Aegean Islands and North Africa and limited areas in the Aegean and Black Sea coastal areas in Turkey (Barbero et al., 1992; Browicz & Zieliński, 1982; de Rigo & Caudullo, 2016; Günal, 2003; Zohary, 1973). The ecological significance of *Q. ilex* forests is highlighted by the current endangered or critically endangered status of many arthropod species that rely on this tree for sustenance but have not yet undergone conservation status assessments (Hernández‐Agüero et al., 2022). It serves as a habitat and breeding ground for various vertebrates in the Iberian Peninsula and provide valuable resources, such as carpentry tools, firewood, and acorns, which are used to feed pigs. Furthermore, it is also utilized for recreational activities in parks and gardens (de Rigo & Caudullo, 2016). Due to these significant ecological and economic roles, *Q. ilex* is considered as a keystone tree species in the midwestern Mediterranean Basin (Hernández‐Agüero et al., 2022) and has been the subject of numerous ecological niche studies in the region. These studies were carried out at local scale in high resolution to predict suitable areas for reforestation or the response of species to future climate change scenarios and conservation concerns (López‐Tirado et al., 2018; López‐Tirado & Hidalgo, 2018; Quinto et al., 2021; Tabet et al., 2018). Consequently, these studies pointed to the need for an ecological niche modeling approach that covers the entire distribution areas of *Q. ilex*. Furthermore, the prediction of ecological niche models to past distribution of *Q. ilex* could lead to a more reliable understanding of the species ecological behavior, at least in terms of response to climatic variables (Nogués‐Bravo, 2009; Vessella et al., 2015).

In this study, potential geographic distributions of *Quercus ilex* L. (holm oak), a keystone species of the Mediterranean ecosystem, were predicted by using ecological niche modeling approach with ten algorithms located in the R‐package “biomod2” for the LIG: ~130 Ka, the LGM: ~22 Ka, mid‐Holocene (MH: ~6 Ka) and future climate scenarios (Representative Concentration Pathway (RCP) 4.5 and 8.5 scenarios for years 2050–2070) obtained from CCSM4 and MIROC‐ESM climate models. For this context, we pursue the following objectives: (i) to model the present distribution of *Q. ilex*, (ii) to investigate Quaternary range dynamics by predicting potential distributions of *Q. ilex*, (iii) to project the future distribution of *Q. ilex* under different climate change scenarios. (iv) to identify potential *Q. ilex* climate refugia for Anatolia under past and future climate change.

## 2 MATERIALS AND METHODS

### 2.1 Occurrence data

**FIGURE 1:**
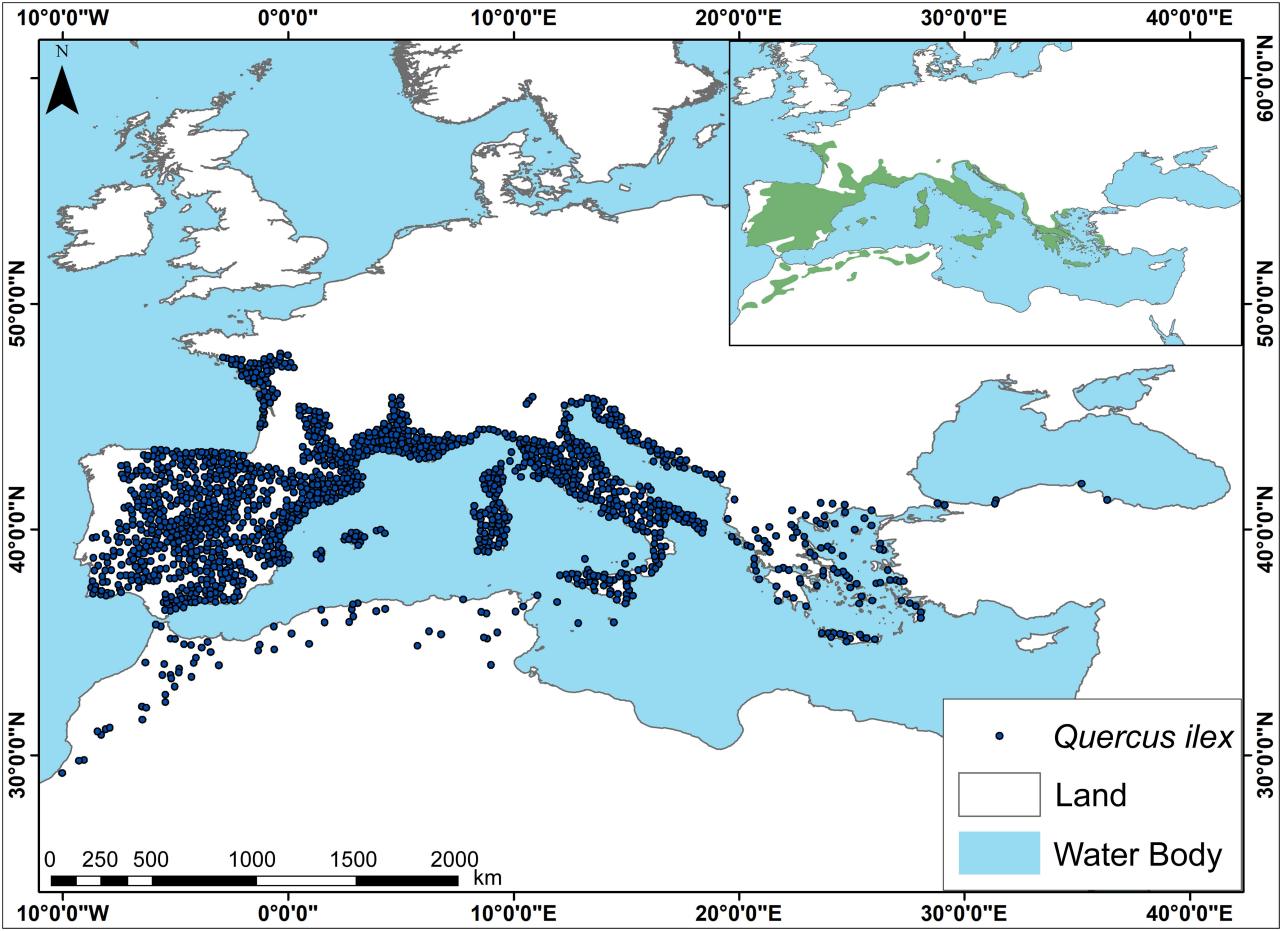
All occurrence records (1606) used in the modeling process representing the geographic distribution of *Quercus ilex* L. (Akkemik et al., 2021; Browicz & Zieliński, 1982; Global Biodiversity Information Facility (GBIF), 2022; Hedge & Yaltırık, 1982; including herbarium specimens) with distribution map representing natural populations of *Q. ilex* form EUFORGEN adapted from Caudullo et al. (2017). The area visible on the maps represents the study area.

We obtained the occurrence records of *Q. ilex* from the Global Biodiversity Information Facility (GBIF) for the Mediterranean Basin (Global Biodiversity Information Facility (GBIF), 2022). Turkey was excluded in the first query because GBIF's data from Anatolia were incomplete and partly incorrect. Thus, 102 occurrence records for Anatolia from the scientific literature and herbaria were added to the GBIF dataset (Akkemik et al., 2021; Browicz & Zieliński, 1982; Hedge & Yaltırık, 1982) As a result, a total of 90,653 occurrence records were considered for the beginning. Then we cleaned the occurrence records step by step. First, we excluded all duplicate records. Since *Q. ilex* does not naturally occur (introduced) in northern Europe and particularly in southern England (Fisher et al., 1994; Southwood et al., 2004), and as GBIF also collects data with citizen science, and not all data refer to natural populations, we removed occurrence records that do not refer to natural populations of the *Q. ilex* according to the limits in the EUFORGEN database (Caudullo et al., 2017). While doing that, we notice that there were some occurrence records in Africa and the Balkans outside of the EUFORGEN limits, we checked those occurrences one by one and included them in our study without removing them because they refer to natural populations. After that, to reduce the effects of sample bias and spatial autocorrelation and improve the prediction performance of the models (Beck et al., 2013; Boria et al., 2014), the records were thinned at 15 km using SDMtoolbox v2.4 (Brown et al., 2017) in ArcGIS version 10.8 (ESRI, 2020). This filter was chosen taking into account the natural distribution of *Q. ilex*, the fact that clusters can occur for ecological reasons, and therefore, removing records from highly clustered areas may obscure true patterns (Barber et al., 2022). This process resulted in 1606 occurrence records for use in ecological niche modeling (Figure 1, Table S1).

### 2.2 Bioclimatic variables and study area

To use in the modeling process, the historical (the LIG: ~130 Ka, the LGM: ~22 Ka, and the MH: ~6 Ka), current (1960–1990), and future (2050–2070) climate projections under intermediate and pessimistic Representative Concentration Pathways (RCP4.5 and RCP8.5), 19 bioclimatic variables with 2.5′ (~4.5 km at the equator) were downloaded from the WorldClim (version 1.4) database (Table S2, Hijmans et al., 2005). The potential equilibrium between species distributions and climate in model predictions contributes to a relatively increased reliability of climate predictions over time (Nogués‐Bravo, 2009). However, model predictions may fail to accurately predict the paleo distribution of a species; this failure indicates that the current distribution data may not fully represent the entire climate range within which the modeled species could exist, and it demonstrates that the reliability of niche models' transferability over time cannot be solely achieved based on current climate equilibrium (Varela et al., 2009). Therefore, in this study, model predictions have been utilized from the LIG to the future. We are aware that various options are available, such as WorldClim v.2.1 (representing the 1971–2000 climate) containing 23 different global climate models (Fick & Hijmans, 2017) or CHELSA (encompassing the 1981–2010 climate; Karger et al., 2017). However, the WorldClim 2.1 version still does not contain data for the past periods and the CHELSA dataset does not provide data for the LIG. Furthermore, due to differences arising from computer limitations, inadequate understanding of physical processes, and climate sensitivity among global climate simulations (Randall et al., 2007), we chose not to use WorldClim v.2.1 for the present and future climate scenarios and WorldClim v.1.4 for the past periods within the same study. In conclusion, despite being an older data version, we utilized WorldClim v.1.4 for all periods included in the study. The present study utilized the Community Climate System Model Version 4 (CCSM4) and Model for Interdisciplinary Research on Climate‐Earth System Model (MIROC‐ESM) global climate models for ecological niche modeling, as they were the only models providing data for all time periods except for the LIG, which was not available for MIROC‐ESM. Therefore, the results for the LIG period were conducted using the CCSM4 exclusively. Initially, we cropped all climate data to the boundaries of our study area (11° W to 45° E; 28°–60° N), which was determined by the known distribution range of *Q. ilex* and possible extensions for past or future range expansions (Figure 1). LIG climate data used in this study were obtained from Otto‐Bliesner et al. (2006). The original resolution of the LIG climate data was 30″ (~1 km), which we resampled to 2.5′ resolution to match the other time periods used in our analysis. Pearson's correlation coefficient was then used to eliminate highly correlated bioclimatic variables, retaining only those with a correlation coefficient below .75 (Table S3, Dormann et al., 2013), which left us seven variables (Table S4): Bio 5 (Max Temperature of Warmest Month), Bio 7 (Annual Temperature Range), Bio 8 (Mean Temperature of Wettest Quarter), Bio 11 (Mean Temperature of Coldest Quarter), Bio 15 (Precipitation Seasonality), Bio 16 (Precipitation of Wettest Quarter), and Bio 17 (Precipitation of Driest Quarter). While selecting bioclimatic variables, the ecological demands of *Q. ilex* were taken into account while reducing collinearity within environmental parameters. Previous studies have shown that both temperature and precipitation play a key role in the distribution of *Q. ilex*. We specifically chose to use seasonal climate variables—the hottest, coldest, wettest, and driest seasonal values—in our study as they have been found to have a stronger influence on *Q. ilex* distribution than annual values (Campelo et al., 2009; Gea‐Izquierdo et al., 2009; Terradas & Savé, 1992). All process and analyses on climate data were performed in R statistics software (version 4.2.2, R Core Team, 2022) with the “terra” package (Hijmans, 2023).

### 2.3 Ecological niche modeling

To generate potential distribution maps of *Q. ilex*, we utilized 10 different algorithms available in the “biomod2” package (version 3.5.1, Thuiller et al., 2009) in R software (version 4.2.2, R Core Team, 2022). The algorithms used were artificial neural network (ANN), classification tree analysis (CTA), flexible discriminant analysis (FDA), generalized additive model (GAM), generalized boosting model (GBM), generalized linear model (GLM), multiple adaptive regression splines (MARS), maximum entropy (MAXENT), random forest (RF), and surface range envelope (SRE). We built individual models using default settings provided by biomod2. Most of the utilized algorithms require both presence and absence data. In contrast, MAXENT uses presence‐only data, and BIOCLIM uses pseudo‐absence data (Thuiller et al., 2009), thus, using the biomod2, we generated 10 sets of an equal number of randomly distributed background points (1606) within the study area to increase precision and avoid random bias (Barbet‐Massin et al., 2012). To generate the MAXENT distribution model, we chose the more common approach and selected 10,000 background points within the study area, again using biomod2, for modeling purposes, which has been recommended in previous literature (Phillips et al., 2006). The models were created with training sets of 80% and 20% data for the validation set. In total, 1000 model runs were conducted, consisting of 10 algorithms, 10 pseudo‐absence selections, and 10 evaluation runs (Figure S1). The relevant code is provided in the Supporting Information (File S1). In order to evaluate the prediction accuracy of the models, the area under the receiver operating characteristic curve (AUC) and true skill statistics (TSS) values are considered together (Guisan et al., 2017). In general, predictive capabilities of AUC values greater than 0.7 indicate good performance, while greater than 0.9 indicate excellent performance (Elith, 2000; Phillips & Dudík, 2008); values above 0.6 and 0.8 for TSS indicate good and excellent performance (Allouche et al., 2006), respectively. Model outputs were presented as means of individual algorithms and were converted into binary predictions (presence/absence) with a given cut‐off value (Table 1) in which the TSS having the highest value was defined as the threshold (Liu et al., 2013). To assess the uncertainty of the results of the individual models, the clamping mask was created, which defines the positions when extrapolation is involved (Figure S2, Elith et al., 2010; Yates et al., 2018).

**TABLE 1:** Table of model performance (AUC, TSS, Sensitivity, Specificity, and Threshold) for 100 repetitions of each algorithm, including artificial neural network (ANN), classification tree analysis (CTA), flexible discriminant analysis (FDA), generalized additive model (GAM), generalized boosting model (GBM), generalized linear model (GLM), multiple adaptive regression splines (MARS), maximum entropy (MAXENT), random forest (RF), and surface range envelope (SRE).

Models	AUC		TSS		Sensitivity	Specificity	Threshold
	Mean	SD	Mean	SD			
ANN	0.932	0.01	0.797	0.03	96.525	83.168	0.371
CTA	0.915	0.01	0.802	0.03	96.037	84.125	0.348
FDA	0.934	0.01	0.791	0.03	95.463	83.593	0.435
GAM	0.938	0.01	0.813	0.02	96.268	85.011	0.500
GBM	0.943	0.01	0.819	0.02	96.428	85.489	0.515
GLM	0.931	0.01	0.794	0.03	95.277	84.065	0.518
MARS	0.936	0.01	0.802	0.02	95.500	84.704	0.483
MAXENT	0.940	0.01	0.808	0.02	95.410	85.413	0.166
RF	0.946	0.01	0.834	0.02	97.232	86.145	0.424
SRE	0.829	0.02	0.659	0.03	75.919	89.950	0.498

## 3 RESULTS

### 3.1 Model evaluation and variable contribution

The results indicate that models demonstrated a high predictive capacity, with true skill statistics (TSS) ranging from 0.659 to 0.834, and ROC curve (AUC) values ranging from 0.829 to 0.946 (Table 1). According to the results, RF (mean TSS = 0.834 ± 0.02 and AUC = 0.946 ± 0.01) was the best‐fitting algorithm and SREs (mean TSS = 0.659 ± 0.03 and AUC = 0.829 ± 0.02) were the worst‐fitting algorithm. Therefore, we will focus on the “biomod2”‐generated RF algorithm, which is observed to be the most suitable for *Q. ilex*. Although the environmental variables made different contributions to different models, in general, “Mean Temperature of Coldest Quarter” (bio 11) had the largest contribution of the majority of the models, except ANN and MAXENT. In the case of RF, bio 11 (34%), bio 5, bio 16, and bio 17 (8%) had the largest contribution (Figure S4).

Based on variable importance and response curves (Figures S3,S4), *Q. ilex* prefers locations where the mean temperature of the coldest quarter is near or above the freezing point, and the probability of distribution is optimum at 5°C in the same quarter. Response curves indicate that the species prefers locations where precipitation is concentrated in the winter months. Considering the response curves representing the driest quarter's rainfall, the species can tolerate locations with less than 50 mm of summer rainfall, while more than 100 mm of rainfall in the summer season is optimal. All these findings indicate that the species prefers mild and rainy winter months, adapting to the Mediterranean climate. As these results align with the literature information, it is concluded that the model's results can successfully describe the ecological requirements of the species.

### 3.2 Present prediction

**FIGURE 2:**
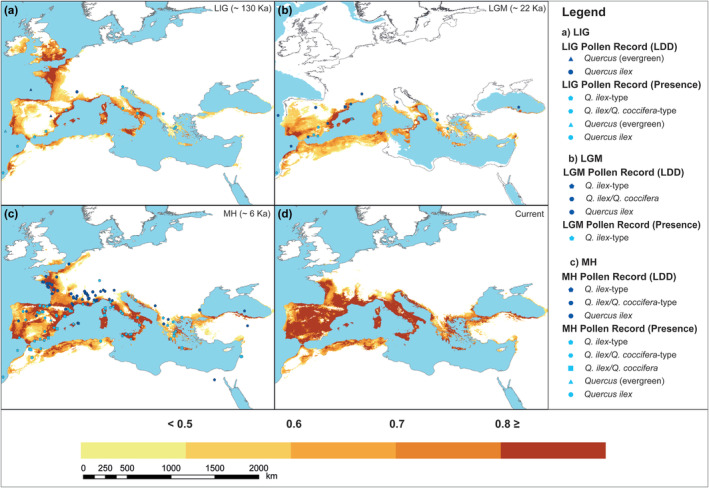
Past and current potential distribution of *Quercus ilex* from the mean of all repetition for random forest algorithm, here only CCSM4 is displayed (for MIROC‐ESM see Figures S15–S24). Darker colors indicate a higher probability of occurrence. Both light and dark blue figures refer to fossil pollen records (Table S5).

Based on models we produced, suitable habitat for *Q. ilex* was predicted to be widespread across the Mediterranean Basin, from the Iberian Peninsula, the Balearic Islands, to the north African coast, the Atlantic region of France (west coast of France), southern France, Corsica, southern Italy, Sardinia, the Dalmatian coast, Greece, the Aegean Islands, Crete, and Asia Minor which is throughout the Black Sea of Turkey and the Turkish Aegean Sea coast. Under current conditions, the model prediction was highly compatible with the known distribution range of *Q. ilex*, which covers the Mediterranean Basin, but extending further east from the true formation (Figure 2d). In contrast to the scattered occurrence of *Q. ilex* in Turkey along the Black Sea, the model predicted suitable habitats further east of the current known distribution, forming a continuous range. However, although not represented with high probability (brighter colors in Figure 2d), distributions were detected in the Black Sea coast of Georgia, around the Iskenderun Bay from the South‐West Mediterranean coast of Turkey, in southern Cyprus and in the Levant region.

### 3.3 Historical prediction

Prediction for the past periods, obtained from two different global climate models and 10 different algorithms, gave consistent results despite regional differences, pointing to similar patterns of contractions and expansions for past periods (Figures S5–S24). The predictions generated by the RF algorithm for past periods are presented alongside fossil pollen records spanning the LIG, LGM, and MH periods (Figure 2). During the LIG period, our model predicted that *Q. ilex* was potentially present along the Mediterranean Basin but was more narrowly scattered along the coasts of Europe, North Africa, and Asia Minor. In the western Mediterranean, a slight northward shift of suitable areas was also observed. A high level of suitability was predicted along the Atlantic coast (Portugal, Gibraltar, the south‐western Iberian Peninsula, Cantabrian Mountains, western France, and western Morocco), Corsica, south Italy, Sicily, Sardinia, Aegean Islands, Crete, and a small part of Turkey in the eastern Black Sea coast (Figure 2a). Transitions between LIG and LGM have been toward climatically favorable areas on the coasts of the Mediterranean Basin (especially North Africa, Balearic Islands, southern France, southern Italy, Adriatic Sea, Aegean Islands, Crete, and Peloponnese), with contraction in distribution in northwestern France and north of the Iberian Peninsula predicted. It is worth noting that the majority of the species' potential range in all of the Mediterranean Basin coasts during the LGM persisted in areas currently submerged. A high level of suitability was predicted along the Aegean and Black Sea coasts of Turkey, around Iskenderun Bay, Levant region, south Iberian Peninsula, Balearic Islands, southern France, southern Italy, and the Adriatic Sea (Figure 2b). During the MH, the potential distribution was similar to the present‐day distribution, as the model prediction indicates. Differences are observed in the central part of the Iberian Peninsula, such as a narrowing of the distribution compared to the current prediction, and a northward shift in suitable areas in western France (Figure 2c).

### 3.4 Fossil pollen data

To compare and validate our model predictions for past periods, we compiled 177 records from the Neotoma Paleoecology Database and the literature, covering the LIG, LGM, and MH periods. These records were described as “*Q. ilex*”, “*Q. ilex*‐type”, “*Q. ilex/Q. coccifera*”, “*Quercus* (evergreen)”, or “*Q. ilex/Q. coccifera*‐type” (Table S5). Some pollen fields contained both continuous and discontinuous low numbers of fossil pollen records. We considered these data to represent long‐distance distribution (LDD) and included them in the analysis, as it can contribute to the validation of our model. Thirteen out of sixteen pollen records for the LIG period are consistent with our model prediction. Three pollen records show low values (indicating LDD) and are 50 km away from our model prediction which indicate nearby presence. The majority of pollen records for the LGM period are generally low values and show LDD. Seven of the pollen records in the south of the Iberian Peninsula and in the Balearic Islands are profiles that record abundant *Q. ilex* and overlap our model prediction. Thirteen pollen records for the LGM period contained low values of *Q. ilex*, five of them overlap our prediction and others were nearby our prediction so that they indicate nearby refugia. Unlike LIG and LGM periods, 81 of the 150 records for the MH period were abundant and continuous throughout the current distribution range and northwest France. There were 69 records indicating LDD. Fifty‐six of the eighty‐one pollen records indicating abundant and continuous *Q. ilex* presence overlap and validate our model predictions.

### 3.5 Future predictions

**FIGURE 3:**
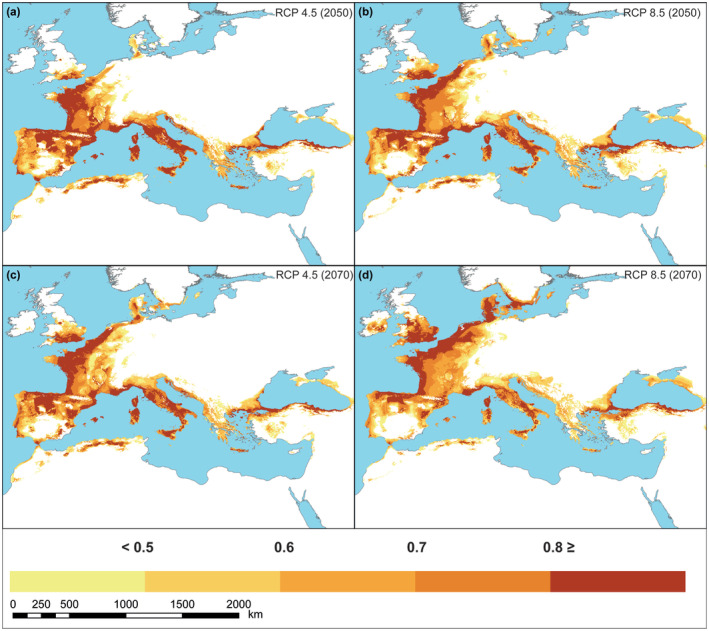
Potential distribution of *Quercus ilex* under climate change scenarios from the mean of all repetition for random forest algorithm outputs, here only CCSM4 displayed (for MIROC‐ESM see Figures S15–S24). Darker colors indicate a higher probability of occurrence.

**FIGURE 4:**
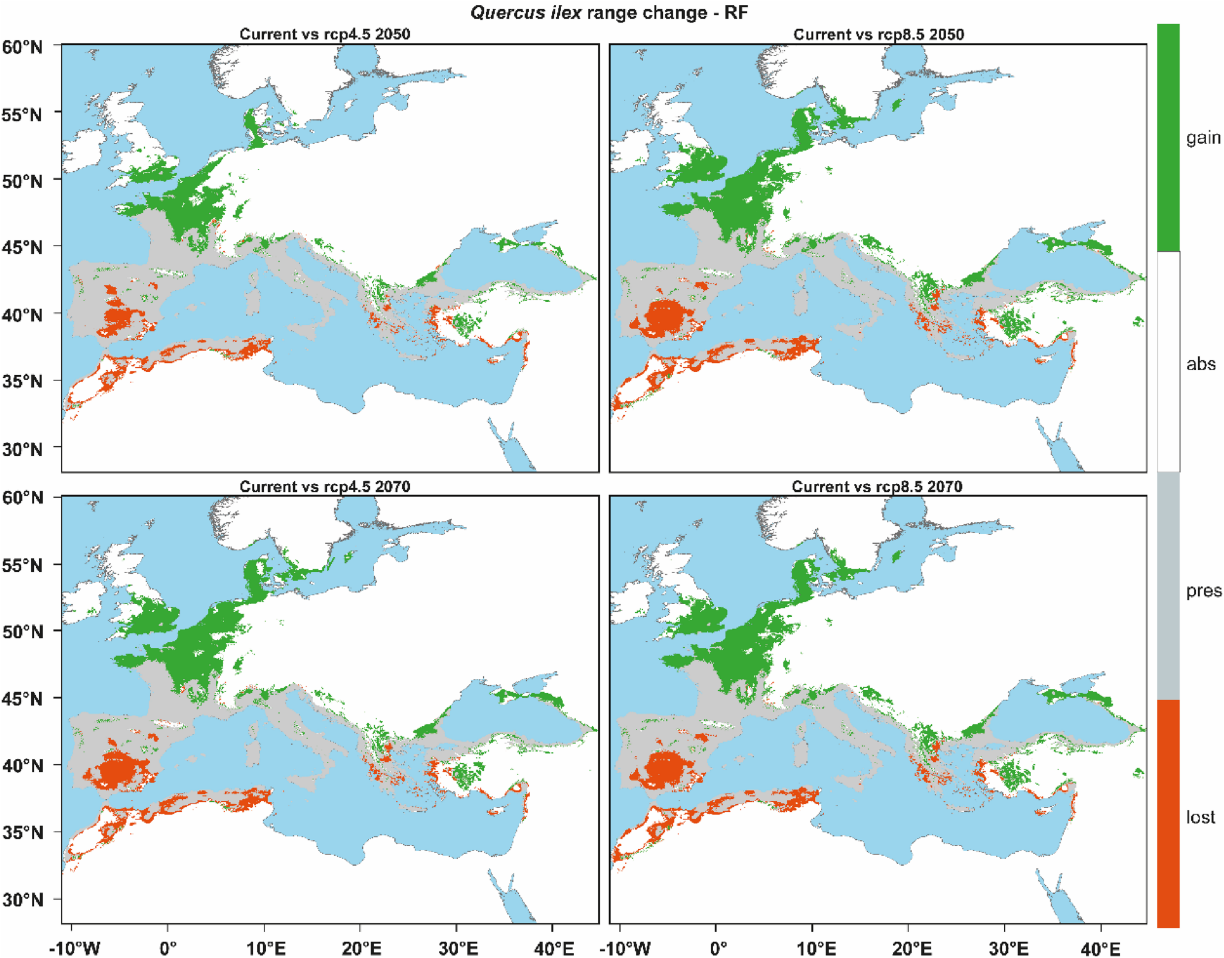
Range change maps of *Quercus ilex* produced from the current and future projections binary map of the RF algorithm outputs. Green areas refer to the expansion of species occurrence based on current predictions and future projections, red areas refer to the contraction, white areas refer to areas predicted to be absent in the future, gray areas refer to the areas predicted to be present in the future.

Based on future predictions, the main geographic shift of *Q. ilex* will be toward the north‐northwest of its current distribution. Moreover, our model revealed potential suitable areas in the Inner‐west Anatolia region for *Q. ilex* under both climate scenarios, despite the species not currently occupying these areas. However, these areas are predicted to have relatively lower levels of suitability. In addition, a serious range contraction is expected in the *Q. ilex* distribution under these scenarios (Figure 3). These contractions are larger in the models obtained with the 2070 RCP 8.5 scenario. Changes in the range of *Q. ilex*, including habitat loss, habitat gain, and habitat stability over time, are shown in Figure 4, Tables S6,S7 for both CCSM4 and MIROC‐ESM GCM. In both climate scenarios (RCP 4.5 and RCP 8.5) and in both models produced for 2050–2070, areas that act as refugia for *Q. ilex* against the effects of the Quaternary climate oscillation in the past on the southern Mediterranean coasts (southern Iberian Peninsula, Sicily, Corsica, Sardinia, and Crete) were predicted by our model to act as a climate refuge in future scenarios (Figure 4), even though the suitability have decreased compared to current prediction (brighter colors in Figure 3). In the RCP 4.5 scenario, a high probability of predicted range change toward northwestern France is observed, whereas in the RCP 8.5 scenario, there is a decrease in the likelihood of suitable areas, leading to a contraction in their extent. (Figure 3d).

## 4 DISCUSSION

The results indicated that the models successfully distinguished occurrence data from the background based on their AUC and TSS values varying from 0.829 to 0.946 for AUC, and from 0.659 to 0.834 (all >0.7 except BIOCLIM, Table 1, Elith et al., 2011) for TSS. Also, consistent results between modeling performances from different algorithms, low standard deviations are noteworthy (Table 1, Figure S25), which is a clue for reproducibility. It is worth mentioning that our past predictions are in agreement with pollen data, suggesting that the ecological niche of *Q. ilex* is species‐climatic equilibrium based on bioclimatic data (Nogués‐Bravo, 2009). This justified us to use the trained model for past and future simulations. The known distribution of *Q. ilex* in Anatolia is characterized by scattered occurrences and does not form pure forests (Barbero et al., 1992; Browicz & Zieliński, 1982; de Rigo & Caudullo, 2016; Zohary, 1973). Nevertheless, our model predicts a continuous distribution of the species along the Black Sea coast of Anatolia (Figure 2d), indicating that it is subjected to significant human impact in this region. Indeed, the observed distributions in Samsun and Zonguldak were limited to urban areas, and the two occurrences near Sinop were fragmented by anthropogenic activities, as reported by Akkemik et al. (2020). The potential presence of *Q. ilex* in the Levant is questionable because the species is not currently found in that region. On the other hand, there is information in the literature that the species exists in Lebanon with fragmented distribution (Menitsky, 2005).

The model outputs indicate that the distribution of the species encompasses regions characterized by the Mediterranean climate, following a significant range contraction during the LIG‐LGM transition from the distribution observed on the Atlantic coast. According to Vessella et al. (2015), based on the average results of seven different algorithms, the distribution of *Q. suber*, another evergreen oak species in the Mediterranean Basin, was found to have extended along the Atlantic coast (from the western Iberian Peninsula to northwestern France) during the LIG period. Subsequently, its distribution contracted to suitable areas in the southern Mediterranean due to the cooling climate during the LGM period, which is in line with the findings of our model. The results concerning the LIG period are reinforced by pollen data, which show continuous pollen values along the southern and eastern coastlines of the Iberian Peninsula, offshore the western shores of North Africa, as well as at the Valle di Castiglione and Lago Grande di Monticchio sites in central Italy, along with the southern Balkans region (Figure 2a, Table S5). This evidence indicates the presence of the *Q. ilex* during LIG period across southern Iberia, western North Africa, the central Mediterranean, and the southern Balkans.

The potential area of *Q. ilex* predicted by our model during LGM was limited to suitable areas in southern Europe along the Mediterranean coast due to the colder and drier climate during LGM (Figure 2b). These areas are consistent with the areas identified as glacial refugia during the LGM (Médail & Diadema, 2009) and support our model predictions. Our findings are further supported by molecular studies. Lumaret et al. (2002) revealed that the recolonization of *Q. ilex* following the last glacial period originated from the three Mediterranean peninsulas. Additionally, our model predicted suitable areas for the species in the south and southeast of the Iberian Peninsula, which is consistent with both molecular and palynological data (López de Heredia et al., 2007) and modeling studies (Benito Garzón et al., 2007) as well. Continuous and abundant pollen records are available from the southern Iberian Peninsula and the Balearic Islands, which are consistent with the areas predicted by our model. In addition, continuous but low pollen records from southern France, southern Italy, southern Greece, and the Black Sea coast of Turkey suggest the presence of nearby refugia during the LGM. Although there are no pollen records from Crete and Sicily, our model predicts these regions as refugia. This prediction is supported by molecular studies (Fineschi et al., 2005; Lumaret et al., 2002), which indicate the diversity of haplotypes in Sicily and its role as one of the colonization centers. Additionally, abundant pollen records from the MH demonstrate the dispersal of the species from refugia to the surrounding environment after the LGM, further supporting our predictions (Table S5). Our models predicted the role of refugia in the Aegean, Black Sea, and Mediterranean coasts, and Amanos Mountains in Anatolia. However, these predictions lack support from both pollen records and molecular studies, with the exception of the Black Sea coast where discontinuous low pollen records exist.

Our model predicted a *Q. ilex* distribution during the MH period that was similar to the present day. As anticipated, the more favorable climatic conditions during the MH allowed for an expansion of the *Q. ilex* range, in contrast to the LGM. However, our model predicted a northward extension along the northwestern coast of France during the MH, which is not observed in the present distribution. Furthermore, our model predicted a more restricted distribution of *Q. ilex* in southern Europe, including the central Iberian Peninsula, Italy, the southern Balkans, and Anatolia, compared to its current range. Moreover, the model was constrained in its ability to predict areas of high climate suitability during the MH period, resulting in a more limited projection compared to the present distribution. Paleoclimatic reconstructions indicate that the MH had a warmer climate than the present, varying by season and region (Mauri et al., 2015; Strandberg et al., 2022). Specifically, in northern Europe, winter temperatures were higher than today, which may have led to a shift in the predicted distribution of *Q. ilex* further northwest into France. Moreover, drier conditions in the Mediterranean region due to reduced summer precipitation and relatively stable winter precipitation (Strandberg et al., 2022) may have contributed to the predicted distribution of *Q. ilex* being more similar to its current distribution. These findings are supported by numerous continuous and discontinuous pollen records obtained from various locations spanning northwest France and the entire Mediterranean Basin (Figure 2c, Table S5). The continuous and abundant pollen records found in the Ammiq and Chamsine locations in Lebanon, Steerenmoos in Germany, and the low but continuous pollen records in Qarun Lake in Egypt do not match the output of our model for the MH period. These records indicate the presence of evergreen oaks in the Levant region, while the records from Germany and Egypt reflect human impact rather than the natural occurrence of the species. Therefore, it is natural that our model outputs do not match these records (Cheddadi & Khater, 2016; Hajar et al., 2010; Hamdan et al., 2020; Rösch et al., 2012).

Our model projections suggest that the distribution of *Q. ilex* in the Mediterranean Basin will experience significant range contractions as a result of climate change, with the southern and central Iberian Peninsula, North Africa, and the Aegean coast of Anatolia being particularly vulnerable under the RCP 4.5 scenario. Under the more severe RCP 8.5 scenario, the contractions are predicted to be even more severe and widespread throughout the basin. These results are consistent with other modeling studies that also predicted range contractions in North Africa. For example, in Algeria, Tabet et al. (2018) predicted a strong negative impact of climate change on *Q. ilex*, resulting in a substantial contraction of its distribution in Algeria. Our results on the distribution of *Q. ilex* in the Iberian and Italian Peninsula contradict the predictions made by López‐Tirado et al. (2018) and López‐Tirado and Hidalgo (2018), who suggested that evergreen oak species, including *Q. ilex*, may expand their range of distribution and benefit from climate change in the western Mediterranean region. However, our approach takes into account the ecological requirements of *Q. ilex* and eliminates highly correlated variables, which helps to prevent overestimation by producing more simple models. Furthermore, by incorporating the *Q. ilex*'s entire range distribution in the model, we established the climate equilibrium for current conditions, which may provide a more realistic prediction of its potential response to future climate change. However, there is an agreement in our mutual prediction that *Q. ilex* will expand its distribution in France, which is a result of other modeling studies in literature (Cheaib et al., 2012). Climate projections for the central and southern regions of the Iberian Peninsula, particularly under the RCP8.5 scenario, indicate a significant decrease in annual precipitation, pointing to pronounced summer droughts (Andrade et al., 2021; Cardoso Pereira et al., 2020). Furthermore, these projections forecast a striking increase in temperatures in these same regions compared to those in others (Viceto et al., 2019). Despite *Q. ilex* being known for its resistance to summer droughts, the increasing temperatures and more frequent occurrences of extreme drought events may strain its capacity for recovery, leading to an increase in its sensitivity to climate change (Barbeta & Peñuelas, 2016; Gea‐Izquierdo et al., 2009). In addition, experimental studies have shown that the drought‐adaptive characteristics of *Q. ilex* leaves increase their tolerance to moderate drought, but do not confer resistance to severe water stress (Limousin et al., 2022). Furthermore, the percentage of seed germination and the development of normal seedlings are closely related to the water content of the seeds after winter, and it has been suggested that drying in situ is a significant cause of seed mortality. Findings reveal that seed drying sensitivity is an important functional trait that may affect the propagation success in temperate climate resistant seed species. Furthermore, reduced winter precipitation can negatively impact the germination success of seeds (Joët et al., 2013, 2016). Moreover, (Martínez‐Vilalta et al., 2003; Ogaya & Peñuelas, 2003) suggest that *Q. ilex* could gradually be replaced by other species that are better adapted to summer droughts. The model outputs for the central and southern regions of the Iberian Peninsula align with these findings and are supported by this information within the context of future scenarios. Therefore, the increase in temperature in the western Mediterranean Basin, the decrease in precipitation, and the changes in the seasonality of precipitation may result in severe suitable area contractions in the southern Iberian Peninsula in our models, in Africa, and in the reduction of high suitable areas along the Mediterranean Basin. Based on future predictions, the main geographic shift of *Q. ilex* will be toward the north‐northwest of its current distribution (Figure 4). It is worth emphasizing that *Q. ilex* is an introduced species in these newly acquired areas in northwestern Europe, especially in southern England (Fisher et al., 1994; Southwood et al., 2004). Regarding the Atlantic coast of France, Delzon et al. (2013) suggest that the rates of *Q. ilex* dispersion and establishment may not keep up with the velocity of climate change, thus limiting its ability to expand into emerging suitable areas. Additionally, our model predicts only moderate levels of suitability for *Q. ilex* in the Inner West Anatolia subregion (Figure 4), which is not currently occupied by the species. Due to the effect of climate change, including increasing temperatures, decreasing frost days, and an extended vegetation period in the region, it is likely that new species, especially evergreen oaks, may colonize the area (Sar et al., 2019). However, considering the precipitation conditions, the colonization of *Q. ilex* in this region appears unlikely. Indeed, our model predictions also show low probability.

**FIGURE 5:**
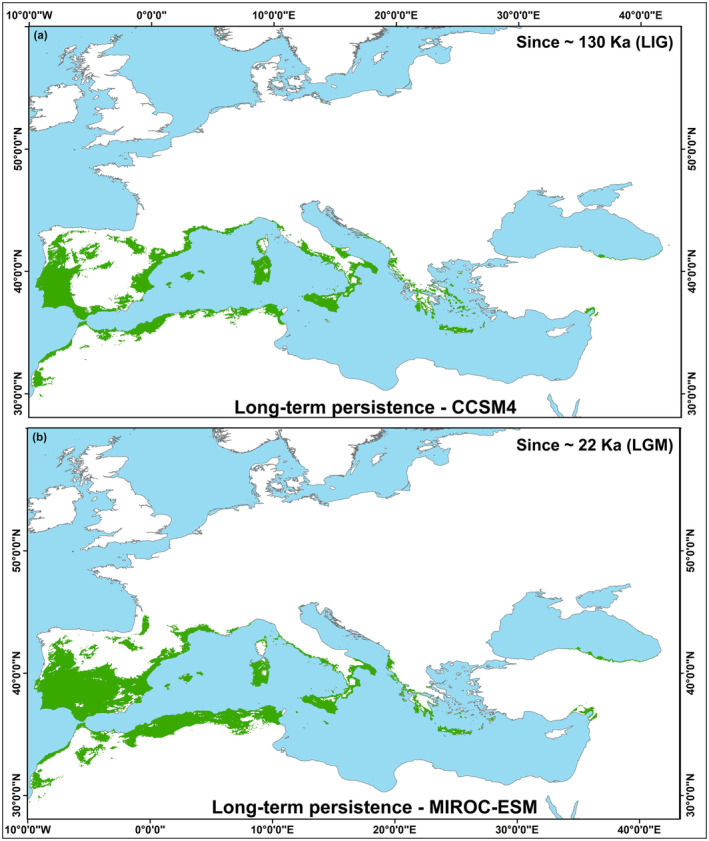
The areas identified as continuously suitable for *Quercus ilex* under the LIG, the LGM, the MH, and the current climate conditions have been inferred as long‐term persistence or putative “long‐term refugia”. This inference was based on the outputs of the random forest (RF) algorithm, using global climate models CCSM4 (a) and MIROC‐ESM (b).

In our past period predictions, areas that are consistently predicted as suitable are noteworthy as long‐term refugia (Figure 5) correspond with modeling studies, phylogeographic studies, and DNA studies specific to *Q. ilex* (López de Heredia et al., 2007; Lumaret et al., 2002; Médail & Diadema, 2009). Furthermore, our model suggests that the Black Sea coast in Anatolia and around Iskenderun Bay may serve as a long‐term refugium for *Q. ilex*. The identification of multiple oak species, subspecies, and varieties in Anatolia suggests its role as a refugia (Hedge & Yaltırık, 1982; Rokas et al., 2003) such that genetic evidence and ecological niche modeling have further supported the notion that Anatolia may have served as a refuge for several evergreen and deciduous oak species during the LGM period (Di Pasquale et al., 2020; Tekpinar et al., 2021; Toumi & Lumaret, 2010; Ülker et al., 2018; Vessella et al., 2015). Unfortunately, there is no evidence of the existence of *Q. ilex* in Anatolia during the LIG period. Nevertheless, Anatolia is considered the origin of the eastern populations of another evergreen oak species, *Q. coccifera* (Toumi & Lumaret, 2010), and is also believed to be one of the possible origins of the deciduous oak species *Q. cerris* (Bagnoli et al., 2016), suggesting that Anatolia may have served as a founding population for *Q. ilex*. Therefore, more detailed molecular studies, particularly in Anatolia, are required to gain a better understanding of the phylogeography of *Q. ilex*.

A noteworthy difference is detected in the long‐term refugia areas observed between the CCSM4 and MIROC‐ESM models in the southern and central parts of the Iberian Peninsula. This difference may arise due to uncertainties within the models themselves or, considering the resemblance of the two climate models' outputs at other locations in the Mediterranean region, be linked to the inclusion/exclusion of the LIG period. The predictions from CCSM4 climate models indicate unsuitable habitats for the LIG period in the southern and central Iberian Peninsula, aligning with the outputs under future climate scenarios. It is known that the ecological trends and responses of the LIG period are recognized as a potential analog to enhance future climate change projections (Kukla et al., 2002; Otto‐Bliesner et al., 2021). The long‐term pollen record from Villarquemado, an area in the interior of the Iberian Peninsula, indicated the presence of *Juniperus* and *Artemisia* pollen records during the LIG period, demonstrating suitability for intense evapotranspiration conditions triggering higher soil water deficit and high thermal seasonality in the region (González‐Sampériz et al., 2020); in contrast, pollen records from Padul, a location in the southern Iberian Peninsula characterized by oceanic influences and mountainous areas, revealed the emergence of both evergreen and deciduous oak species, and *Pistacia*, as well as moisture‐demanding species such as *Betula*, *Alnus*, and *Fraxinus* during the LIG period, indicating warm and humid interglacial climate conditions (Camuera et al., 2019). Climate projections covering the Iberian Peninsula notably point to a significant decrease in annual precipitation and an increase in precipitation seasonality in the central region (Andrade et al., 2021; Cardoso Pereira et al., 2020), positioning it as an area with the highest projected increase in annual mean temperatures on the Iberian Peninsula (Viceto et al., 2019). The similarities in model predictions within the central Iberian Peninsula between the LIG period and future climate change scenarios can be attributed to decreased precipitation and increased evapotranspiration due to rising temperatures and continental conditions.

## 5 CONCLUSIONS

In this study, we investigated the late Quaternary range changes of *Q. ilex* by ecological niche modeling and predicted the future distribution of the species in response to global climate change. For that we applied 10 different algorithms provided by “biomod2” among them, the RF algorithm performed best with more plausible results for both past and future distributions. We then compared the model outputs with palynological records from LIG, LGM, and MH to validate that the results were in agreement with the palynological data. Our model defined a long‐term refugia role for *Q. ilex* in the Mediterranean Basin (Figure 5) in past prediction. These areas overlap with chloroplast DNA studies. In addition, the long‐term refugia role of the east Black Sea coast in Anatolia and around Iskenderun Bay has been observed. While our model predictions do not have sufficient genetic or pollen data to support the potential role of Anatolia as a long‐term refuge for *Q. ilex* populations, they do emphasize the need for further, more detailed molecular studies involving Anatolia to gain a better understanding of the phylogeography of *Q. ilex*. The results of our future projection analyses indicate that both RCP 4.5 and RCP 8.5 scenarios will have a considerable impact on the distribution range of *Q. ilex*. While significant reductions in suitable areas were observed in the central and southern regions of the Iberian Peninsula and North Africa, the model predicts a general contraction of suitable areas in northern Italy, southern Greece, and the Aegean coasts of Anatolia, due to less favorable climate conditions. Notably, our model predicts a substantial contraction of suitable areas in the maps produced for the year 2070, particularly under the RCP 8.5 scenario, in regions that were identified as long‐term refugia in the past period. It should be noted that the current study excludes local adaptation. Although the correlative modeling approach has been emphasized in the literature for potentially underestimating species range dynamics (Benito Garzón et al., 2019; Hoffmann & Sgrò, 2011; Waldvogel et al., 2020), we would like to emphasize that this study identifies areas within the entire distribution range of *Q. ilex* where the species may be susceptible to the consequences of climate change, even if there is some potential for underestimation. These findings identify regions that require prioritization for conservation efforts. Indeed, the observed habitat losses in genetically diverse areas that exhibit high suitability for the species, acting as long‐term refugia, underscore the need for studies that integrate local adaptation and ENM approaches at a local level in these regions. Our models predicted a range shift toward the north and northwest of the current *Q. ilex* distribution under both scenarios, resulting in a significant increase in available area for the species in these regions in the future. However, the ability of *Q. ilex* to migrate to these potential suitable areas remains uncertain due to the limitations of dispersal and establishment in the relatively short time frame considered. This study may be extended by incorporating additional environmental variables, such as soil type, elevation, and aspect, in addition to climate variables, and by employing alternative climate models, as appropriate. Nevertheless, the inclusion of these variables in ecological niche models is subject to the availability of data within the range of the species, which may be lacking or require certain assumptions that may affect the reliability of the results, particularly for distant past time periods. Despite being an older data version, we utilized WorldClim v.1.4 for all the periods included in the study. However, it is important to note that WorldClim v.2.1 has benefited from data recorded by a higher number of meteorological stations, especially at higher latitudes and different elevations. As a result, there is a possibility of inconsistencies in model predictions compared to those generated by WorldClim v.1.4 across Europe (Cerasoli et al., 2022). Once WorldClim v.2.1 version for past periods becomes available, it would allow the study to be replicated using more up‐to‐date data, thereby strengthening the representation of outputs within future scenarios. The Mediterranean Basin is one of the most vulnerable climate change hotspots in the world, and thus, understanding how climate change will affect the distribution of plant species in the Mediterranean Basin allows better species risk assessment and conservation management to be prepared. This study reveals important findings about the sensitivity of the *Q. ilex* species, which can be considered as a key species for the Mediterranean ecosystem, future climate change, and points out the importance of Anatolia for the species and our study suggests that more detailed molecular studies involving Anatolia are needed to understand the phylogeography of *Q. ilex*.

## AUTHOR CONTRIBUTIONS

**Burak Suicmez:** Conceptualization (equal); data curation (equal); formal analysis (lead); investigation (equal); methodology (equal); resources (equal); software (lead); validation (equal); visualization (equal); writing – original draft (lead). **Meral Avci:** Conceptualization (equal); data curation (equal); investigation (equal); methodology (equal); project administration (lead); resources (equal); supervision (lead); validation (equal); visualization (equal); writing – review and editing (lead).

## CONFLICT OF INTEREST STATEMENT

The authors declare that they have no known competing financial interests or personal relationships that could have appeared to influence the work reported in this paper.

## Supporting information


File S1.

Figure S1–S25.

Table S1.

Table S2.

Table S3.

Table S4.

Table S5.

Table S6.

Table S7.

## Data Availability

Datasets and programming codes are available in Supporting Information.
