# Supplementary material for: Distribution patterns of Quercus ilex from the last interglacial period to the future by ecological niche modeling
Source: Ecol Evol. 2023 Oct 19;13(10):e10606. doi: 10.1002/ece3.10606 (PMC10585444; doi:10.1002/ece3.10606)
Supplement: Supplementary file 1 — File S1. [file ECE3-13-e10606-s003.zip › S1_File_SuppInfo.docx]

**S1 File.** R code sheet for biomod2 package to perform SDM.

### Set working directory:

setwd("C:/Users/Hp/Desktop/Quercus_7_3.5.1")

## setting seed for repeatability

set.seed(1)

## Load the required library into the R environment

library(biomod2)

library(raster)

## Import occurrence data into R environment

quercus <- read.csv(

"C:/Users/Hp/Desktop/sdm_data/occurrence/Quercus_ilex.csv",sep = ";")

# Add the columns that contains "1" to indicate occurrence data

quercus <- cbind(quercus, rep.int(1, length(nrow(quercus))))

colnames(quercus)[4] <- c("Quercus ilex")

## Import current climate data into R environment

current_bio <- stack(

c(

bio_5 = ("C:/Users/Hp/Desktop/sdm_data/Layers/Wordclim_v1.4/Quercus_Extent_Layer/Current_2.5/bio5.tif"),

bio_7 = ("C:/Users/Hp/Desktop/sdm_data/Layers/Wordclim_v1.4/Quercus_Extent_Layer/Current_2.5/bio7.tif"),

bio_8 = ("C:/Users/Hp/Desktop/sdm_data/Layers/Wordclim_v1.4/Quercus_Extent_Layer/Current_2.5/bio8.tif"),

bio_11=("C:/Users/Hp/Desktop/sdm_data/Layers/Wordclim_v1.4/Quercus_Extent_Layer/Current_2.5/bio11.tif"),

bio_15=("C:/Users/Hp/Desktop/sdm_data/Layers/Wordclim_v1.4/Quercus_Extent_Layer/Current_2.5/bio15.tif"),

bio_16=("C:/Users/Hp/Desktop/sdm_data/Layers/Wordclim_v1.4/Quercus_Extent_Layer/Current_2.5/bio16.tif"),

bio_17=("C:/Users/Hp/Desktop/sdm_data/Layers/Wordclim_v1.4/Quercus_Extent_Layer/Current_2.5/bio17.tif")

)

)

## Format the data as biomod2 required

respname <- "Quercus.ilex"

respvar <- quercus[,4]

respxy <- quercus[,2:3]

quercus_data <-

BIOMOD_FormatingData(

resp.var = respvar,

expl.var = current_bio,

resp.xy = respxy,

resp.name = respname,

PA.nb.rep = 10,

PA.nb.absences = 1606,

PA.strategy = "random"

)

## Define individual models options

# Model setting same as biomod2 optimal options except for MAXENT which we gave seperate pseudo absence data

modeloption <- BIOMOD_ModelingOptions(

MAXENT = list( path_to_maxent.jar = "C:/Users/Hp/Desktop/sdm_data/maxent",

memory_allocated = 2048,

background_data_dir = "C:/Users/Hp/Desktop/sdm_data/maxent/background_data",

maximumbackground = 10000

)

)

## Modelling process

# by algorithm

quercus_model_output <-

BIOMOD_Modeling(

data = quercus_data,

models = c("GLM", "GBM", "GAM", "CTA", "ANN", "SRE", "FDA", "MARS", "RF", "MAXENT.Phillips"),

models.options = modeloption,

NbRunEval = 10,

DataSplit = 80,

VarImport = 3,

models.eval.meth = c("TSS", "ROC"),

do.full.models = FALSE,

rescal.all.models = FALSE,

SaveObj = TRUE,

modeling.id = paste(respname,"modelling", sep = "_")

)

## get models evaluation scores as data frame

quercus_model_eva <- get_evaluations(quercus_model_output)

quercus_model_eva_df <- get_evaluations(quercus_model_output, as.data.frame = T)

## Check variable importance

# Variable importance as data frame

quercus_model_var_import <- get_variables_importance(quercus_model_output, as.data.frame = T)

## Make the mean of variable importance by algorithm and save to solid disk as csv file

quercus_mean_variable <- round(apply(quercus_model_var_import, c(1,2), mean), digits = 2)

write.csv(quercus_mean_variable, file = "C:/Users/Hp/Desktop/Quercus_7_3.5.1/variables.csv")

## Current projections

quercusprojcurrent <-

BIOMOD_Projection(

modeling.output = quercus_model_output,

new.env = current_bio,

proj.name = "current",

selected.models = "all",

binary.meth = "TSS",

clamping.mask = TRUE, #Uncertainty

do.stack = FALSE

)

### Climate change projections

# CCSM4 #

## Import lig period climate data into R environment

lig_bio <- stack(

c(

bio_5 = ("C:/Users/Hp/Desktop/sdm_data/Layers/Wordclim_v1.4/Quercus_Extent_Layer/LIG.2.5/bio5.tif"),

bio_7 = ("C:/Users/Hp/Desktop/sdm_data/Layers/Wordclim_v1.4/Quercus_Extent_Layer/LIG.2.5/bio7.tif"),

bio_8 = ("C:/Users/Hp/Desktop/sdm_data/Layers/Wordclim_v1.4/Quercus_Extent_Layer/LIG.2.5/bio8.tif"),

bio_11=("C:/Users/Hp/Desktop/sdm_data/Layers/Wordclim_v1.4/Quercus_Extent_Layer/LIG.2.5/bio11.tif"),

bio_15=("C:/Users/Hp/Desktop/sdm_data/Layers/Wordclim_v1.4/Quercus_Extent_Layer/LIG.2.5/bio15.tif"),

bio_16=("C:/Users/Hp/Desktop/sdm_data/Layers/Wordclim_v1.4/Quercus_Extent_Layer/LIG.2.5/bio16.tif"),

bio_17=("C:/Users/Hp/Desktop/sdm_data/Layers/Wordclim_v1.4/Quercus_Extent_Layer/LIG.2.5/bio17.tif")

)

)

## Lig projections

quercusprojlig <-

BIOMOD_Projection(

modeling.output = quercus_model_output,

new.env = lig_bio,

proj.name = "lig",

selected.models = "all",

binary.meth = "TSS",

clamping.mask = TRUE,

do.stack = FALSE

)

## Import lgm period climate data into R environment

lgm_bio <- stack(

c(

bio_5 = ("C:/Users/Hp/Desktop/sdm_data/Layers/Wordclim_v1.4/Quercus_Extent_Layer/CCSM4_LGM_2.5/bio5.tif"),

bio_7 = ("C:/Users/Hp/Desktop/sdm_data/Layers/Wordclim_v1.4/Quercus_Extent_Layer/CCSM4_LGM_2.5/bio7.tif"),

bio_8 = ("C:/Users/Hp/Desktop/sdm_data/Layers/Wordclim_v1.4/Quercus_Extent_Layer/CCSM4_LGM_2.5/bio8.tif"),

bio_11 = ("C:/Users/Hp/Desktop/sdm_data/Layers/Wordclim_v1.4/Quercus_Extent_Layer/CCSM4_LGM_2.5/bio11.tif"),

bio_15 = ("C:/Users/Hp/Desktop/sdm_data/Layers/Wordclim_v1.4/Quercus_Extent_Layer/CCSM4_LGM_2.5/bio15.tif"),

bio_16 = ("C:/Users/Hp/Desktop/sdm_data/Layers/Wordclim_v1.4/Quercus_Extent_Layer/CCSM4_LGM_2.5/bio16.tif"),

bio_17 = ("C:/Users/Hp/Desktop/sdm_data/Layers/Wordclim_v1.4/Quercus_Extent_Layer/CCSM4_LGM_2.5/bio17.tif")

)

)

## Lgm projections

quercusprojlgm <-

BIOMOD_Projection(

modeling.output = quercus_model_output,

new.env = lgm_bio,

proj.name = "lgm",

selected.models = "all",

binary.meth = "TSS",

clamping.mask = TRUE,

do.stack = FALSE

)

## Import mh period climate data into R environment

mh_bio <- stack(

c(

bio_5 = ("C:/Users/Hp/Desktop/sdm_data/Layers/Wordclim_v1.4/Quercus_Extent_Layer/CCSM4_MH_2.5/bio5.tif"),

bio_7 = ("C:/Users/Hp/Desktop/sdm_data/Layers/Wordclim_v1.4/Quercus_Extent_Layer/CCSM4_MH_2.5/bio7.tif"),

bio_8 = ("C:/Users/Hp/Desktop/sdm_data/Layers/Wordclim_v1.4/Quercus_Extent_Layer/CCSM4_MH_2.5/bio8.tif"),

bio_11=("C:/Users/Hp/Desktop/sdm_data/Layers/Wordclim_v1.4/Quercus_Extent_Layer/CCSM4_MH_2.5/bio11.tif"),

bio_15=("C:/Users/Hp/Desktop/sdm_data/Layers/Wordclim_v1.4/Quercus_Extent_Layer/CCSM4_MH_2.5/bio15.tif"),

bio_16=("C:/Users/Hp/Desktop/sdm_data/Layers/Wordclim_v1.4/Quercus_Extent_Layer/CCSM4_MH_2.5/bio16.tif"),

bio_17=("C:/Users/Hp/Desktop/sdm_data/Layers/Wordclim_v1.4/Quercus_Extent_Layer/CCSM4_MH_2.5/bio17.tif")

)

)

## Mh projections

quercusprojmh <-

BIOMOD_Projection(

modeling.output = quercus_model_output,

new.env = mh_bio,

proj.name = "mh",

selected.models = "all",

binary.meth = "TSS",

clamping.mask = TRUE,

do.stack = FALSE

)

## Import rcp4.5_2050 period climate data into R environment

rcp4.5_bio_2050 <- stack(

c(

bio_5 = ("C:/Users/Hp/Desktop/sdm_data/Layers/Wordclim_v1.4/Quercus_Extent_Layer/CCSM4_2050_rcp4.5_2.5/bio5.tif"),

bio_7 = ("C:/Users/Hp/Desktop/sdm_data/Layers/Wordclim_v1.4/Quercus_Extent_Layer/CCSM4_2050_rcp4.5_2.5/bio7.tif"),

bio_8 = ("C:/Users/Hp/Desktop/sdm_data/Layers/Wordclim_v1.4/Quercus_Extent_Layer/CCSM4_2050_rcp4.5_2.5/bio8.tif"),

bio_11=("C:/Users/Hp/Desktop/sdm_data/Layers/Wordclim_v1.4/Quercus_Extent_Layer/CCSM4_2050_rcp4.5_2.5/bio11.tif"),

bio_15=("C:/Users/Hp/Desktop/sdm_data/Layers/Wordclim_v1.4/Quercus_Extent_Layer/CCSM4_2050_rcp4.5_2.5/bio15.tif"),

bio_16=("C:/Users/Hp/Desktop/sdm_data/Layers/Wordclim_v1.4/Quercus_Extent_Layer/CCSM4_2050_rcp4.5_2.5/bio16.tif"),

bio_17=("C:/Users/Hp/Desktop/sdm_data/Layers/Wordclim_v1.4/Quercus_Extent_Layer/CCSM4_2050_rcp4.5_2.5/bio17.tif")

)

)

## rcp4.5_2050 projections

quercusprojrcp4.5_2050 <-

BIOMOD_Projection(

modeling.output = quercus_model_output,

new.env = rcp4.5_bio_2050,

proj.name = "rcp4.5_2050",

selected.models = "all",

binary.meth = "TSS",

clamping.mask = TRUE,

do.stack = FALSE

)

## Import rcp8.5_2050 period climate data into R environment

rcp8.5_bio_2050 <- stack(

c(

bio_5=("C:/Users/Hp/Desktop/sdm_data/Layers/Wordclim_v1.4/Quercus_Extent_Layer/CCSM4_2050_rcp8.5_2.5/bio5.tif"),

bio_7=("C:/Users/Hp/Desktop/sdm_data/Layers/Wordclim_v1.4/Quercus_Extent_Layer/CCSM4_2050_rcp8.5_2.5/bio7.tif"),

bio_8=("C:/Users/Hp/Desktop/sdm_data/Layers/Wordclim_v1.4/Quercus_Extent_Layer/CCSM4_2050_rcp8.5_2.5/bio8.tif"),

bio_11=("C:/Users/Hp/Desktop/sdm_data/Layers/Wordclim_v1.4/Quercus_Extent_Layer/CCSM4_2050_rcp8.5_2.5/bio11.tif"),

bio_15=("C:/Users/Hp/Desktop/sdm_data/Layers/Wordclim_v1.4/Quercus_Extent_Layer/CCSM4_2050_rcp8.5_2.5/bio15.tif"),

bio_16=("C:/Users/Hp/Desktop/sdm_data/Layers/Wordclim_v1.4/Quercus_Extent_Layer/CCSM4_2050_rcp8.5_2.5/bio16.tif"),

bio_17=("C:/Users/Hp/Desktop/sdm_data/Layers/Wordclim_v1.4/Quercus_Extent_Layer/CCSM4_2050_rcp8.5_2.5/bio17.tif")

)

)

## rcp8.5_2050 projections

quercusprojrcp8.5_2050 <-

BIOMOD_Projection(

modeling.output = quercus_model_output,

new.env = rcp8.5_bio_2050,

proj.name = "rcp8.5_2050",

selected.models = "all",

binary.meth = "TSS",

clamping.mask = TRUE,

do.stack = FALSE

)

## Import rcp4.5_2070 period climate data into R environment

rcp4.5_bio_2070 <- stack(

c(

bio_5=("C:/Users/Hp/Desktop/sdm_data/Layers/Wordclim_v1.4/quercus_Extent_Layer/CCSM4_2070_rcp4.5_2.5/bio5.tif"),

bio_7=("C:/Users/Hp/Desktop/sdm_data/Layers/Wordclim_v1.4/quercus_Extent_Layer/CCSM4_2070_rcp4.5_2.5/bio7.tif"),

bio_8=("C:/Users/Hp/Desktop/sdm_data/Layers/Wordclim_v1.4/quercus_Extent_Layer/CCSM4_2070_rcp4.5_2.5/bio8.tif"),

bio_11=("C:/Users/Hp/Desktop/sdm_data/Layers/Wordclim_v1.4/quercus_Extent_Layer/CCSM4_2070_rcp4.5_2.5/bio11.tif"),

bio_15=("C:/Users/Hp/Desktop/sdm_data/Layers/Wordclim_v1.4/quercus_Extent_Layer/CCSM4_2070_rcp4.5_2.5/bio15.tif"),

bio_16=("C:/Users/Hp/Desktop/sdm_data/Layers/Wordclim_v1.4/quercus_Extent_Layer/CCSM4_2070_rcp4.5_2.5/bio16.tif"),

bio_17=("C:/Users/Hp/Desktop/sdm_data/Layers/Wordclim_v1.4/quercus_Extent_Layer/CCSM4_2070_rcp4.5_2.5/bio17.tif")

)

)

## rcp4.5_2070 projections

quercusprojrcp4.5_2070 <-

BIOMOD_Projection(

modeling.output = quercus_model_output,

new.env = rcp4.5_bio_2070,

proj.name = "rcp4.5_2070",

selected.models = "all",

binary.meth = "TSS",

clamping.mask = TRUE,

do.stack = FALSE

)

## Import rcp8.5_2070 period climate data into R environment

rcp8.5_bio_2070 <- stack(

c(

bio_5=("C:/Users/Hp/Desktop/sdm_data/Layers/Wordclim_v1.4/Quercus_Extent_Layer/CCSM4_2070_rcp8.5_2.5/bio5.tif"),

bio_7=("C:/Users/Hp/Desktop/sdm_data/Layers/Wordclim_v1.4/Quercus_Extent_Layer/CCSM4_2070_rcp8.5_2.5/bio7.tif"),

bio_8=("C:/Users/Hp/Desktop/sdm_data/Layers/Wordclim_v1.4/Quercus_Extent_Layer/CCSM4_2070_rcp8.5_2.5/bio8.tif"),

bio_11=("C:/Users/Hp/Desktop/sdm_data/Layers/Wordclim_v1.4/Quercus_Extent_Layer/CCSM4_2070_rcp8.5_2.5/bio11.tif"),

bio_15=("C:/Users/Hp/Desktop/sdm_data/Layers/Wordclim_v1.4/Quercus_Extent_Layer/CCSM4_2070_rcp8.5_2.5/bio15.tif"),

bio_16=("C:/Users/Hp/Desktop/sdm_data/Layers/Wordclim_v1.4/Quercus_Extent_Layer/CCSM4_2070_rcp8.5_2.5/bio16.tif"),

bio_17=("C:/Users/Hp/Desktop/sdm_data/Layers/Wordclim_v1.4/Quercus_Extent_Layer/CCSM4_2070_rcp8.5_2.5/bio17.tif")

)

)

## rcp8.5_2070 projections

quercusprojrcp8.5_2070 <-

BIOMOD_Projection(

modeling.output = quercus_model_output,

new.env = rcp8.5_bio_2070,

proj.name = "rcp8.5_2070",

selected.models = "all",

binary.meth = "TSS",

clamping.mask = TRUE,

do.stack = FALSE

)

# MIROC-ESM #

## Import lgm period climate data into R environment

lgm_bio_miroc <- stack(

c(

bio_5 = ("C:/Users/Hp/Desktop/sdm_data/Layers/Wordclim_v1.4/Quercus_Extent_Layer/MIROC-ESM_LGM_2.5/bio5.tif"),

bio_7 = ("C:/Users/Hp/Desktop/sdm_data/Layers/Wordclim_v1.4/Quercus_Extent_Layer/MIROC-ESM_LGM_2.5/bio7.tif"),

bio_8 = ("C:/Users/Hp/Desktop/sdm_data/Layers/Wordclim_v1.4/Quercus_Extent_Layer/MIROC-ESM_LGM_2.5/bio8.tif"),

bio_11 = ("C:/Users/Hp/Desktop/sdm_data/Layers/Wordclim_v1.4/Quercus_Extent_Layer/MIROC-ESM_LGM_2.5/bio11.tif"),

bio_15 = ("C:/Users/Hp/Desktop/sdm_data/Layers/Wordclim_v1.4/Quercus_Extent_Layer/MIROC-ESM_LGM_2.5/bio15.tif"),

bio_16 = ("C:/Users/Hp/Desktop/sdm_data/Layers/Wordclim_v1.4/Quercus_Extent_Layer/MIROC-ESM_LGM_2.5/bio16.tif"),

bio_17 = ("C:/Users/Hp/Desktop/sdm_data/Layers/Wordclim_v1.4/Quercus_Extent_Layer/MIROC-ESM_LGM_2.5/bio17.tif")

)

)

## Lgm projections-MIROC

quercusprojlgm_miroc <-

BIOMOD_Projection(

modeling.output = quercus_model_output,

new.env = lgm_bio_miroc,

proj.name = "lgm_miroc",

selected.models = "all",

binary.meth = "TSS",

clamping.mask = TRUE,

do.stack = FALSE

)

## Import mh period climate data into R environment

mh_bio_miroc <- stack(

c(

bio_5 = ("C:/Users/Hp/Desktop/sdm_data/Layers/Wordclim_v1.4/Quercus_Extent_Layer/MIROC-ESM_MH_2.5/bio5.tif"),

bio_7 = ("C:/Users/Hp/Desktop/sdm_data/Layers/Wordclim_v1.4/Quercus_Extent_Layer/MIROC-ESM_MH_2.5/bio7.tif"),

bio_8 = ("C:/Users/Hp/Desktop/sdm_data/Layers/Wordclim_v1.4/Quercus_Extent_Layer/MIROC-ESM_MH_2.5/bio8.tif"),

bio_11=("C:/Users/Hp/Desktop/sdm_data/Layers/Wordclim_v1.4/Quercus_Extent_Layer/MIROC-ESM_MH_2.5/bio11.tif"),

bio_15=("C:/Users/Hp/Desktop/sdm_data/Layers/Wordclim_v1.4/Quercus_Extent_Layer/MIROC-ESM_MH_2.5/bio15.tif"),

bio_16=("C:/Users/Hp/Desktop/sdm_data/Layers/Wordclim_v1.4/Quercus_Extent_Layer/MIROC-ESM_MH_2.5/bio16.tif"),

bio_17=("C:/Users/Hp/Desktop/sdm_data/Layers/Wordclim_v1.4/Quercus_Extent_Layer/MIROC-ESM_MH_2.5/bio17.tif")

)

)

quercusprojmh_miroc <-

BIOMOD_Projection(

modeling.output = quercus_model_output,

new.env = mh_bio_miroc,

proj.name = "mh_miroc",

selected.models = "all",

binary.meth = "TSS",

clamping.mask = TRUE,

do.stack = FALSE

)

## Import rcp4.5_2050 period climate data into R environment

rcp4.5_bio_2050_miroc <- stack(

c(

bio_5 = ("C:/Users/Hp/Desktop/sdm_data/Layers/Wordclim_v1.4/Quercus_Extent_Layer/MIROC-ESM_2050_rcp4.5_2.5/bio5.tif"),

bio_7 = ("C:/Users/Hp/Desktop/sdm_data/Layers/Wordclim_v1.4/Quercus_Extent_Layer/MIROC-ESM_2050_rcp4.5_2.5/bio7.tif"),

bio_8 = ("C:/Users/Hp/Desktop/sdm_data/Layers/Wordclim_v1.4/Quercus_Extent_Layer/MIROC-ESM_2050_rcp4.5_2.5/bio8.tif"),

bio_11=("C:/Users/Hp/Desktop/sdm_data/Layers/Wordclim_v1.4/Quercus_Extent_Layer/MIROC-ESM_2050_rcp4.5_2.5/bio11.tif"),

bio_15=("C:/Users/Hp/Desktop/sdm_data/Layers/Wordclim_v1.4/Quercus_Extent_Layer/MIROC-ESM_2050_rcp4.5_2.5/bio15.tif"),

bio_16=("C:/Users/Hp/Desktop/sdm_data/Layers/Wordclim_v1.4/Quercus_Extent_Layer/MIROC-ESM_2050_rcp4.5_2.5/bio16.tif"),

bio_17=("C:/Users/Hp/Desktop/sdm_data/Layers/Wordclim_v1.4/Quercus_Extent_Layer/MIROC-ESM_2050_rcp4.5_2.5/bio17.tif")

)

)

## rcp4.5_2050 projections

quercusprojrcp4.5_2050_miroc <-

BIOMOD_Projection(

modeling.output = quercus_model_output,

new.env = rcp4.5_bio_2050_miroc,

proj.name = "rcp4.5_2050_miroc",

selected.models = "all",

binary.meth = "TSS",

clamping.mask = TRUE,

do.stack = FALSE

)

## Import rcp8.5_2050 period climate data into R environment

rcp8.5_bio_2050_miroc <- stack(

c(

bio_5 = ("C:/Users/Hp/Desktop/sdm_data/Layers/Wordclim_v1.4/Quercus_Extent_Layer/MIROC-ESM_2050_rcp8.5_2.5/bio5.tif"),

bio_7 = ("C:/Users/Hp/Desktop/sdm_data/Layers/Wordclim_v1.4/Quercus_Extent_Layer/MIROC-ESM_2050_rcp8.5_2.5/bio7.tif"),

bio_8 = ("C:/Users/Hp/Desktop/sdm_data/Layers/Wordclim_v1.4/Quercus_Extent_Layer/MIROC-ESM_2050_rcp8.5_2.5/bio8.tif"),

bio_11=("C:/Users/Hp/Desktop/sdm_data/Layers/Wordclim_v1.4/Quercus_Extent_Layer/MIROC-ESM_2050_rcp8.5_2.5/bio11.tif"),

bio_15=("C:/Users/Hp/Desktop/sdm_data/Layers/Wordclim_v1.4/Quercus_Extent_Layer/MIROC-ESM_2050_rcp8.5_2.5/bio15.tif"),

bio_16=("C:/Users/Hp/Desktop/sdm_data/Layers/Wordclim_v1.4/Quercus_Extent_Layer/MIROC-ESM_2050_rcp8.5_2.5/bio16.tif"),

bio_17=("C:/Users/Hp/Desktop/sdm_data/Layers/Wordclim_v1.4/Quercus_Extent_Layer/MIROC-ESM_2050_rcp8.5_2.5/bio17.tif")

)

)

## rcp8.5_2050 projections

quercusprojrcp8.5_2050_miroc <-

BIOMOD_Projection(

modeling.output = quercus_model_output,

new.env = rcp8.5_bio_2050_miroc,

proj.name = "rcp8.5_2050_miroc",

selected.models = "all",

binary.meth = "TSS",

clamping.mask = TRUE,

do.stack = FALSE

)

## Import rcp4.5_2070 period climate data into R environment

rcp4.5_bio_2070_miroc <- stack(

c(

bio_5 = ("C:/Users/Hp/Desktop/sdm_data/Layers/Wordclim_v1.4/quercus_Extent_Layer/MIROC-ESM_2070_rcp4.5_2.5/bio5.tif"),

bio_7 = ("C:/Users/Hp/Desktop/sdm_data/Layers/Wordclim_v1.4/quercus_Extent_Layer/MIROC-ESM_2070_rcp4.5_2.5/bio7.tif"),

bio_8 = ("C:/Users/Hp/Desktop/sdm_data/Layers/Wordclim_v1.4/quercus_Extent_Layer/MIROC-ESM_2070_rcp4.5_2.5/bio8.tif"),

bio_11=("C:/Users/Hp/Desktop/sdm_data/Layers/Wordclim_v1.4/quercus_Extent_Layer/MIROC-ESM_2070_rcp4.5_2.5/bio11.tif"),

bio_15=("C:/Users/Hp/Desktop/sdm_data/Layers/Wordclim_v1.4/quercus_Extent_Layer/MIROC-ESM_2070_rcp4.5_2.5/bio15.tif"),

bio_16=("C:/Users/Hp/Desktop/sdm_data/Layers/Wordclim_v1.4/quercus_Extent_Layer/MIROC-ESM_2070_rcp4.5_2.5/bio16.tif"),

bio_17=("C:/Users/Hp/Desktop/sdm_data/Layers/Wordclim_v1.4/quercus_Extent_Layer/MIROC-ESM_2070_rcp4.5_2.5/bio17.tif")

)

)

## rcp4.5_2070 projections

quercusprojrcp4.5_2070_miroc <-

BIOMOD_Projection(

modeling.output = quercus_model_output,

new.env = rcp4.5_bio_2070_miroc,

proj.name = "rcp4.5_2070_miroc",

selected.models = "all",

binary.meth = "TSS",

clamping.mask = TRUE,

do.stack = FALSE

)

## Import rcp8.5_2070 period climate data into R environment

rcp8.5_bio_2070_miroc <- stack(

c(

bio_5 = ("C:/Users/Hp/Desktop/sdm_data/Layers/Wordclim_v1.4/Quercus_Extent_Layer/MIROC-ESM_2070_rcp8.5_2.5/bio5.tif"),

bio_7 = ("C:/Users/Hp/Desktop/sdm_data/Layers/Wordclim_v1.4/Quercus_Extent_Layer/MIROC-ESM_2070_rcp8.5_2.5/bio7.tif"),

bio_8 = ("C:/Users/Hp/Desktop/sdm_data/Layers/Wordclim_v1.4/Quercus_Extent_Layer/MIROC-ESM_2070_rcp8.5_2.5/bio8.tif"),

bio_11=("C:/Users/Hp/Desktop/sdm_data/Layers/Wordclim_v1.4/Quercus_Extent_Layer/MIROC-ESM_2070_rcp8.5_2.5/bio11.tif"),

bio_15=("C:/Users/Hp/Desktop/sdm_data/Layers/Wordclim_v1.4/Quercus_Extent_Layer/MIROC-ESM_2070_rcp8.5_2.5/bio15.tif"),

bio_16=("C:/Users/Hp/Desktop/sdm_data/Layers/Wordclim_v1.4/Quercus_Extent_Layer/MIROC-ESM_2070_rcp8.5_2.5/bio16.tif"),

bio_17=("C:/Users/Hp/Desktop/sdm_data/Layers/Wordclim_v1.4/Quercus_Extent_Layer/MIROC-ESM_2070_rcp8.5_2.5/bio17.tif")

)

)

## rcp8.5_2070 projections

quercusprojrcp8.5_2070_miroc <-

BIOMOD_Projection(

modeling.output = quercus_model_output,

new.env = rcp8.5_bio_2070_miroc,

proj.name = "rcp8.5_2070_miroc",

selected.models = "all",

binary.meth = "TSS",

clamping.mask = TRUE,

do.stack = FALSE

)
