# Supplementary material for: Distribution patterns of Quercus ilex from the last interglacial period to the future by ecological niche modeling
Source: Ecol Evol. 2023 Oct 19;13(10):e10606. doi: 10.1002/ece3.10606 (PMC10585444; doi:10.1002/ece3.10606)
Supplement: Supplementary file 1 — File S1. [file ECE3-13-e10606-s003.zip › Supplements.pdf]

S1 File. R code sheet for biomod2 package to perform SDM.

```
### Set working directory:
setwd("C:/Users/Hp/Desktop/Quercus_7_3.5.1")

## setting seed for repeatability
set.seed(1)

## Load the required library into the R environment
library(biomod2)
library(raster)


## Import occurrence data into R environment
quercus <- read.csv(
  "C:/Users/Hp/Desktop/sdm_data/occurrence/Quercus_ilex.csv",sep = ";")


# Add the columns that contains "1" to indicate occurrence data
quercus <- cbind(quercus, rep.int(1, length(nrow(quercus))))
colnames(quercus)[4] <- c("Quercus ilex")


## Import current climate data into R environment
current_bio <- stack(
  c(
    bio_5 =
("C:/Users/Hp/Desktop/sdm_data/Layers/Wordclim_v1.4/Quercus_Extent_Layer/Current_2.5/bio5.tif
"),
    bio_7 =
("C:/Users/Hp/Desktop/sdm_data/Layers/Wordclim_v1.4/Quercus_Extent_Layer/Current_2.5/bio7.tif
"),
    bio_8 =
("C:/Users/Hp/Desktop/sdm_data/Layers/Wordclim_v1.4/Quercus_Extent_Layer/Current_2.5/bio8.tif
"),

bio_11=("C:/Users/Hp/Desktop/sdm_data/Layers/Wordclim_v1.4/Quercus_Extent_Layer/Current_2.5
/bio11.tif"),

bio_15=("C:/Users/Hp/Desktop/sdm_data/Layers/Wordclim_v1.4/Quercus_Extent_Layer/Current_2.5
/bio15.tif"),

bio_16=("C:/Users/Hp/Desktop/sdm_data/Layers/Wordclim_v1.4/Quercus_Extent_Layer/Current_2.5
/bio16.tif"),

bio_17=("C:/Users/Hp/Desktop/sdm_data/Layers/Wordclim_v1.4/Quercus_Extent_Layer/Current_2.5
/bio17.tif")
  )
)


## Format the data as biomod2 required
respname <- "Quercus.ilex"
respvar <- quercus[,4]
respxy <- quercus[,2:3]
```

```
quercus_data <-
  BIOMOD_FormatingData(
    resp.var = respvar,
    expl.var = current_bio,
    resp.xy = respxy,
    resp.name = respname,
    PA.nb.rep = 10,
    PA.nb.absences = 1606,
    PA.strategy = "random"
  )


## Define individual models options
# Model setting same as biomod2 optimal options except for MAXENT which we gave seperate
pseudo absence data
modeloption <- BIOMOD_ModelingOptions(
  MAXENT = list( path_to_maxent.jar = "C:/Users/Hp/Desktop/sdm_data/maxent",
                 memory_allocated = 2048,
                 background_data_dir = "C:/Users/Hp/Desktop/sdm_data/maxent/background_data",
                 maximumbackground = 10000
  )
)


## Modelling process
# by algorithm
quercus_model_output <-
  BIOMOD_Modeling(
    data = quercus_data,
    models = c("GLM", "GBM", "GAM", "CTA", "ANN", "SRE", "FDA", "MARS", "RF",
"MAXENT.Phillips"),
    models.options = modeloption,
    NbRunEval = 10,
    DataSplit = 80,
    VarImport = 3,
    models.eval.meth = c("TSS", "ROC"),
    do.full.models = FALSE,
    rescal.all.models = FALSE,
    SaveObj = TRUE,
    modeling.id = paste(respname,"modelling", sep = "_")
  )


## get models evaluation scores as data frame
quercus_model_eva <- get_evaluations(quercus_model_output)
quercus_model_eva_df <- get_evaluations(quercus_model_output, as.data.frame = T)


## Check variable importance
# Variable importance as data frame
quercus_model_var_import <- get_variables_importance(quercus_model_output, as.data.frame = T)

## Make the mean of variable importance by algorithm and save to solid disk as csv file
quercus_mean_variable <- round(apply(quercus_model_var_import, c(1,2), mean), digits = 2)
```

```
write.csv(quercus_mean_variable, file = "C:/Users/Hp/Desktop/Quercus_7_3.5.1/variables.csv")


## Current projections
quercusprojcurrent <-
  BIOMOD_Projection(
    modeling.output = quercus_model_output,
    new.env = current_bio,
    proj.name = "current",
    selected.models = "all",
    binary.meth = "TSS",
    clamping.mask = TRUE, #Uncertainty
    do.stack = FALSE
  )

### Climate change projections
# CCSM4 #
## Import lig period climate data into R environment
lig_bio <- stack(
  c(
    bio_5 =
("C:/Users/Hp/Desktop/sdm_data/Layers/Wordclim_v1.4/Quercus_Extent_Layer/LIG.2.5/bio5.tif"),
    bio_7 =
("C:/Users/Hp/Desktop/sdm_data/Layers/Wordclim_v1.4/Quercus_Extent_Layer/LIG.2.5/bio7.tif"),
    bio_8 =
("C:/Users/Hp/Desktop/sdm_data/Layers/Wordclim_v1.4/Quercus_Extent_Layer/LIG.2.5/bio8.tif"),

bio_11=("C:/Users/Hp/Desktop/sdm_data/Layers/Wordclim_v1.4/Quercus_Extent_Layer/LIG.2.5/bio
11.tif"),

bio_15=("C:/Users/Hp/Desktop/sdm_data/Layers/Wordclim_v1.4/Quercus_Extent_Layer/LIG.2.5/bio
15.tif"),

bio_16=("C:/Users/Hp/Desktop/sdm_data/Layers/Wordclim_v1.4/Quercus_Extent_Layer/LIG.2.5/bio
16.tif"),

bio_17=("C:/Users/Hp/Desktop/sdm_data/Layers/Wordclim_v1.4/Quercus_Extent_Layer/LIG.2.5/bio
17.tif")
  )
)


## Lig projections
quercusprojlig <-
  BIOMOD_Projection(
    modeling.output = quercus_model_output,
    new.env = lig_bio,
    proj.name = "lig",
    selected.models = "all",
    binary.meth = "TSS",
    clamping.mask = TRUE,
    do.stack = FALSE
  )
```

```
## Import lgm period climate data into R environment
lgm_bio <- stack(
  c(
    bio_5 =
("C:/Users/Hp/Desktop/sdm_data/Layers/Wordclim_v1.4/Quercus_Extent_Layer/CCSM4_LGM_2.5/
bio5.tif"),
    bio_7 =
("C:/Users/Hp/Desktop/sdm_data/Layers/Wordclim_v1.4/Quercus_Extent_Layer/CCSM4_LGM_2.5/
bio7.tif"),
    bio_8 =
("C:/Users/Hp/Desktop/sdm_data/Layers/Wordclim_v1.4/Quercus_Extent_Layer/CCSM4_LGM_2.5/
bio8.tif"),
    bio_11 =
("C:/Users/Hp/Desktop/sdm_data/Layers/Wordclim_v1.4/Quercus_Extent_Layer/CCSM4_LGM_2.5/
bio11.tif"),
    bio_15 =
("C:/Users/Hp/Desktop/sdm_data/Layers/Wordclim_v1.4/Quercus_Extent_Layer/CCSM4_LGM_2.5/
bio15.tif"),
    bio_16 =
("C:/Users/Hp/Desktop/sdm_data/Layers/Wordclim_v1.4/Quercus_Extent_Layer/CCSM4_LGM_2.5/
bio16.tif"),
    bio_17 =
("C:/Users/Hp/Desktop/sdm_data/Layers/Wordclim_v1.4/Quercus_Extent_Layer/CCSM4_LGM_2.5/
bio17.tif")
  )
)

## Lgm projections
quercusprojlgm <-
  BIOMOD_Projection(
    modeling.output = quercus_model_output,
    new.env = lgm_bio,
    proj.name = "lgm",
    selected.models = "all",
    binary.meth = "TSS",
    clamping.mask = TRUE,
    do.stack = FALSE
  )


## Import mh period climate data into R environment
mh_bio <- stack(
  c(
    bio_5 =
("C:/Users/Hp/Desktop/sdm_data/Layers/Wordclim_v1.4/Quercus_Extent_Layer/CCSM4_MH_2.5/bi
o5.tif"),
    bio_7 =
("C:/Users/Hp/Desktop/sdm_data/Layers/Wordclim_v1.4/Quercus_Extent_Layer/CCSM4_MH_2.5/bi
o7.tif"),
    bio_8 =
("C:/Users/Hp/Desktop/sdm_data/Layers/Wordclim_v1.4/Quercus_Extent_Layer/CCSM4_MH_2.5/bi
o8.tif"),

bio_11=("C:/Users/Hp/Desktop/sdm_data/Layers/Wordclim_v1.4/Quercus_Extent_Layer/CCSM4_M
H_2.5/bio11.tif"),
```

```
bio_15=("C:/Users/Hp/Desktop/sdm_data/Layers/Wordclim_v1.4/Quercus_Extent_Layer/CCSM4_M
H_2.5/bio15.tif"),

bio_16=("C:/Users/Hp/Desktop/sdm_data/Layers/Wordclim_v1.4/Quercus_Extent_Layer/CCSM4_M
H_2.5/bio16.tif"),

bio_17=("C:/Users/Hp/Desktop/sdm_data/Layers/Wordclim_v1.4/Quercus_Extent_Layer/CCSM4_M
H_2.5/bio17.tif")
  )
)


## Mh projections
quercusprojmh <-
  BIOMOD_Projection(
    modeling.output = quercus_model_output,
    new.env = mh_bio,
    proj.name = "mh",
    selected.models = "all",
    binary.meth = "TSS",
    clamping.mask = TRUE,
    do.stack = FALSE
  )


## Import rcp4.5_2050 period climate data into R environment
rcp4.5_bio_2050 <- stack(
  c(
    bio_5 =
("C:/Users/Hp/Desktop/sdm_data/Layers/Wordclim_v1.4/Quercus_Extent_Layer/CCSM4_2050_rcp4.
5_2.5/bio5.tif"),
    bio_7 =
("C:/Users/Hp/Desktop/sdm_data/Layers/Wordclim_v1.4/Quercus_Extent_Layer/CCSM4_2050_rcp4.
5_2.5/bio7.tif"),
    bio_8 =
("C:/Users/Hp/Desktop/sdm_data/Layers/Wordclim_v1.4/Quercus_Extent_Layer/CCSM4_2050_rcp4.
5_2.5/bio8.tif"),

bio_11=("C:/Users/Hp/Desktop/sdm_data/Layers/Wordclim_v1.4/Quercus_Extent_Layer/CCSM4_20
50_rcp4.5_2.5/bio11.tif"),

bio_15=("C:/Users/Hp/Desktop/sdm_data/Layers/Wordclim_v1.4/Quercus_Extent_Layer/CCSM4_20
50_rcp4.5_2.5/bio15.tif"),

bio_16=("C:/Users/Hp/Desktop/sdm_data/Layers/Wordclim_v1.4/Quercus_Extent_Layer/CCSM4_20
50_rcp4.5_2.5/bio16.tif"),

bio_17=("C:/Users/Hp/Desktop/sdm_data/Layers/Wordclim_v1.4/Quercus_Extent_Layer/CCSM4_20
50_rcp4.5_2.5/bio17.tif")
  )
)


## rcp4.5_2050 projections
```

```
quercusprojrcp4.5_2050 <-
  BIOMOD_Projection(
    modeling.output = quercus_model_output,
    new.env = rcp4.5_bio_2050,
    proj.name = "rcp4.5_2050",
    selected.models = "all",
    binary.meth = "TSS",
    clamping.mask = TRUE,
    do.stack = FALSE
  )


## Import rcp8.5_2050 period climate data into R environment
rcp8.5_bio_2050 <- stack(
  c(

bio_5=("C:/Users/Hp/Desktop/sdm_data/Layers/Wordclim_v1.4/Quercus_Extent_Layer/CCSM4_2050_rcp8.5_2.5/bio5.tif"),

bio_7=("C:/Users/Hp/Desktop/sdm_data/Layers/Wordclim_v1.4/Quercus_Extent_Layer/CCSM4_2050_rcp8.5_2.5/bio7.tif"),

bio_8=("C:/Users/Hp/Desktop/sdm_data/Layers/Wordclim_v1.4/Quercus_Extent_Layer/CCSM4_2050_rcp8.5_2.5/bio8.tif"),

bio_11=("C:/Users/Hp/Desktop/sdm_data/Layers/Wordclim_v1.4/Quercus_Extent_Layer/CCSM4_2050_rcp8.5_2.5/bio11.tif"),

bio_15=("C:/Users/Hp/Desktop/sdm_data/Layers/Wordclim_v1.4/Quercus_Extent_Layer/CCSM4_2050_rcp8.5_2.5/bio15.tif"),

bio_16=("C:/Users/Hp/Desktop/sdm_data/Layers/Wordclim_v1.4/Quercus_Extent_Layer/CCSM4_2050_rcp8.5_2.5/bio16.tif"),

bio_17=("C:/Users/Hp/Desktop/sdm_data/Layers/Wordclim_v1.4/Quercus_Extent_Layer/CCSM4_2050_rcp8.5_2.5/bio17.tif")
  )
)


## rcp8.5_2050 projections
quercusprojrcp8.5_2050 <-
  BIOMOD_Projection(
    modeling.output = quercus_model_output,
    new.env = rcp8.5_bio_2050,
    proj.name = "rcp8.5_2050",
    selected.models = "all",
    binary.meth = "TSS",
    clamping.mask = TRUE,
    do.stack = FALSE
  )


## Import rcp4.5_2070 period climate data into R environment
rcp4.5_bio_2070 <- stack(
```

```
  c(

bio_5=("C:/Users/Hp/Desktop/sdm_data/Layers/Wordclim_v1.4/quercus_Extent_Layer/CCSM4_2070_rcp4.5_2.5/bio5.tif"),

bio_7=("C:/Users/Hp/Desktop/sdm_data/Layers/Wordclim_v1.4/quercus_Extent_Layer/CCSM4_2070_rcp4.5_2.5/bio7.tif"),

bio_8=("C:/Users/Hp/Desktop/sdm_data/Layers/Wordclim_v1.4/quercus_Extent_Layer/CCSM4_2070_rcp4.5_2.5/bio8.tif"),

bio_11=("C:/Users/Hp/Desktop/sdm_data/Layers/Wordclim_v1.4/quercus_Extent_Layer/CCSM4_2070_rcp4.5_2.5/bio11.tif"),

bio_15=("C:/Users/Hp/Desktop/sdm_data/Layers/Wordclim_v1.4/quercus_Extent_Layer/CCSM4_2070_rcp4.5_2.5/bio15.tif"),

bio_16=("C:/Users/Hp/Desktop/sdm_data/Layers/Wordclim_v1.4/quercus_Extent_Layer/CCSM4_2070_rcp4.5_2.5/bio16.tif"),

bio_17=("C:/Users/Hp/Desktop/sdm_data/Layers/Wordclim_v1.4/quercus_Extent_Layer/CCSM4_2070_rcp4.5_2.5/bio17.tif")
  )
)


## rcp4.5_2070 projections
quercusprojrcp4.5_2070 <-
  BIOMOD_Projection(
    modeling.output = quercus_model_output,
    new.env = rcp4.5_bio_2070,
    proj.name = "rcp4.5_2070",
    selected.models = "all",
    binary.meth = "TSS",
    clamping.mask = TRUE,
    do.stack = FALSE
  )


## Import rcp8.5_2070 period climate data into R environment
rcp8.5_bio_2070 <- stack(
  c(

bio_5=("C:/Users/Hp/Desktop/sdm_data/Layers/Wordclim_v1.4/Quercus_Extent_Layer/CCSM4_2070_rcp8.5_2.5/bio5.tif"),

bio_7=("C:/Users/Hp/Desktop/sdm_data/Layers/Wordclim_v1.4/Quercus_Extent_Layer/CCSM4_2070_rcp8.5_2.5/bio7.tif"),

bio_8=("C:/Users/Hp/Desktop/sdm_data/Layers/Wordclim_v1.4/Quercus_Extent_Layer/CCSM4_2070_rcp8.5_2.5/bio8.tif"),

bio_11=("C:/Users/Hp/Desktop/sdm_data/Layers/Wordclim_v1.4/Quercus_Extent_Layer/CCSM4_2070_rcp8.5_2.5/bio11.tif"),
```

```
bio_15=("C:/Users/Hp/Desktop/sdm_data/Layers/Wordclim_v1.4/Quercus_Extent_Layer/CCSM4_2070_rcp8.5_2.5/bio15.tif"),

bio_16=("C:/Users/Hp/Desktop/sdm_data/Layers/Wordclim_v1.4/Quercus_Extent_Layer/CCSM4_2070_rcp8.5_2.5/bio16.tif"),

bio_17=("C:/Users/Hp/Desktop/sdm_data/Layers/Wordclim_v1.4/Quercus_Extent_Layer/CCSM4_2070_rcp8.5_2.5/bio17.tif")
  )
)


## rcp8.5_2070 projections
quercusprojrcp8.5_2070 <-
  BIOMOD_Projection(
    modeling.output = quercus_model_output,
    new.env = rcp8.5_bio_2070,
    proj.name = "rcp8.5_2070",
    selected.models = "all",
    binary.meth = "TSS",
    clamping.mask = TRUE,
    do.stack = FALSE
  )




# MIROC-ESM #
## Import lgm period climate data into R environment
lgm_bio_miroc <- stack(
  c(
    bio_5 = ("C:/Users/Hp/Desktop/sdm_data/Layers/Wordclim_v1.4/Quercus_Extent_Layer/MIROC-ESM_LGM_2.5/bio5.tif"),
    bio_7 = ("C:/Users/Hp/Desktop/sdm_data/Layers/Wordclim_v1.4/Quercus_Extent_Layer/MIROC-ESM_LGM_2.5/bio7.tif"),
    bio_8 = ("C:/Users/Hp/Desktop/sdm_data/Layers/Wordclim_v1.4/Quercus_Extent_Layer/MIROC-ESM_LGM_2.5/bio8.tif"),
    bio_11 = ("C:/Users/Hp/Desktop/sdm_data/Layers/Wordclim_v1.4/Quercus_Extent_Layer/MIROC-ESM_LGM_2.5/bio11.tif"),
    bio_15 = ("C:/Users/Hp/Desktop/sdm_data/Layers/Wordclim_v1.4/Quercus_Extent_Layer/MIROC-ESM_LGM_2.5/bio15.tif"),
    bio_16 = ("C:/Users/Hp/Desktop/sdm_data/Layers/Wordclim_v1.4/Quercus_Extent_Layer/MIROC-ESM_LGM_2.5/bio16.tif"),
    bio_17 = ("C:/Users/Hp/Desktop/sdm_data/Layers/Wordclim_v1.4/Quercus_Extent_Layer/MIROC-ESM_LGM_2.5/bio17.tif")
  )
)

## Lgm projections-MIROC
quercusprojlgm_miroc <-
```

```
  BIOMOD_Projection(
    modeling.output = quercus_model_output,
    new.env = lgm_bio_miroc,
    proj.name = "lgm_miroc",
    selected.models = "all",
    binary.meth = "TSS",
    clamping.mask = TRUE,
    do.stack = FALSE
  )


## Import mh period climate data into R environment
mh_bio_miroc <- stack(
  c(
    bio_5 = ("C:/Users/Hp/Desktop/sdm_data/Layers/Wordclim_v1.4/Quercus_Extent_Layer/MIROC-ESM_MH_2.5/bio5.tif"),
    bio_7 = ("C:/Users/Hp/Desktop/sdm_data/Layers/Wordclim_v1.4/Quercus_Extent_Layer/MIROC-ESM_MH_2.5/bio7.tif"),
    bio_8 = ("C:/Users/Hp/Desktop/sdm_data/Layers/Wordclim_v1.4/Quercus_Extent_Layer/MIROC-ESM_MH_2.5/bio8.tif"),
    bio_11=("C:/Users/Hp/Desktop/sdm_data/Layers/Wordclim_v1.4/Quercus_Extent_Layer/MIROC-ESM_MH_2.5/bio11.tif"),
    bio_15=("C:/Users/Hp/Desktop/sdm_data/Layers/Wordclim_v1.4/Quercus_Extent_Layer/MIROC-ESM_MH_2.5/bio15.tif"),
    bio_16=("C:/Users/Hp/Desktop/sdm_data/Layers/Wordclim_v1.4/Quercus_Extent_Layer/MIROC-ESM_MH_2.5/bio16.tif"),
    bio_17=("C:/Users/Hp/Desktop/sdm_data/Layers/Wordclim_v1.4/Quercus_Extent_Layer/MIROC-ESM_MH_2.5/bio17.tif")
  )
)


quercusprojmh_miroc <-
  BIOMOD_Projection(
    modeling.output = quercus_model_output,
    new.env = mh_bio_miroc,
    proj.name = "mh_miroc",
    selected.models = "all",
    binary.meth = "TSS",
    clamping.mask = TRUE,
    do.stack = FALSE
  )


## Import rcp4.5_2050 period climate data into R environment
rcp4.5_bio_2050_miroc <- stack(
  c(
    bio_5 = ("C:/Users/Hp/Desktop/sdm_data/Layers/Wordclim_v1.4/Quercus_Extent_Layer/MIROC-ESM_2050_rcp4.5_2.5/bio5.tif"),
    bio_7 = ("C:/Users/Hp/Desktop/sdm_data/Layers/Wordclim_v1.4/Quercus_Extent_Layer/MIROC-ESM_2050_rcp4.5_2.5/bio7.tif"),
    bio_8 = ("C:/Users/Hp/Desktop/sdm_data/Layers/Wordclim_v1.4/Quercus_Extent_Layer/MIROC-ESM_2050_rcp4.5_2.5/bio8.tif"),
    bio_11=("C:/Users/Hp/Desktop/sdm_data/Layers/Wordclim_v1.4/Quercus_Extent_Layer/MIROC-ESM_2050_rcp4.5_2.5/bio11.tif"),
```

```
    bio_15=("C:/Users/Hp/Desktop/sdm_data/Layers/Wordclim_v1.4/Quercus_Extent_Layer/MIROC-ESM_2050_rcp4.5_2.5/bio15.tif"),
    bio_16=("C:/Users/Hp/Desktop/sdm_data/Layers/Wordclim_v1.4/Quercus_Extent_Layer/MIROC-ESM_2050_rcp4.5_2.5/bio16.tif"),
    bio_17=("C:/Users/Hp/Desktop/sdm_data/Layers/Wordclim_v1.4/Quercus_Extent_Layer/MIROC-ESM_2050_rcp4.5_2.5/bio17.tif")
  )
)


## rcp4.5_2050 projections
quercusprojrcp4.5_2050_miroc <-
  BIOMOD_Projection(
    modeling.output = quercus_model_output,
    new.env = rcp4.5_bio_2050_miroc,
    proj.name = "rcp4.5_2050_miroc",
    selected.models = "all",
    binary.meth = "TSS",
    clamping.mask = TRUE,
    do.stack = FALSE
  )


## Import rcp8.5_2050 period climate data into R environment
rcp8.5_bio_2050_miroc <- stack(
  c(
    bio_5 = ("C:/Users/Hp/Desktop/sdm_data/Layers/Wordclim_v1.4/Quercus_Extent_Layer/MIROC-ESM_2050_rcp8.5_2.5/bio5.tif"),
    bio_7 = ("C:/Users/Hp/Desktop/sdm_data/Layers/Wordclim_v1.4/Quercus_Extent_Layer/MIROC-ESM_2050_rcp8.5_2.5/bio7.tif"),
    bio_8 = ("C:/Users/Hp/Desktop/sdm_data/Layers/Wordclim_v1.4/Quercus_Extent_Layer/MIROC-ESM_2050_rcp8.5_2.5/bio8.tif"),
    bio_11=("C:/Users/Hp/Desktop/sdm_data/Layers/Wordclim_v1.4/Quercus_Extent_Layer/MIROC-ESM_2050_rcp8.5_2.5/bio11.tif"),
    bio_15=("C:/Users/Hp/Desktop/sdm_data/Layers/Wordclim_v1.4/Quercus_Extent_Layer/MIROC-ESM_2050_rcp8.5_2.5/bio15.tif"),
    bio_16=("C:/Users/Hp/Desktop/sdm_data/Layers/Wordclim_v1.4/Quercus_Extent_Layer/MIROC-ESM_2050_rcp8.5_2.5/bio16.tif"),
    bio_17=("C:/Users/Hp/Desktop/sdm_data/Layers/Wordclim_v1.4/Quercus_Extent_Layer/MIROC-ESM_2050_rcp8.5_2.5/bio17.tif")
  )
)


## rcp8.5_2050 projections
quercusprojrcp8.5_2050_miroc <-
  BIOMOD_Projection(
    modeling.output = quercus_model_output,
    new.env = rcp8.5_bio_2050_miroc,
    proj.name = "rcp8.5_2050_miroc",
    selected.models = "all",
    binary.meth = "TSS",
    clamping.mask = TRUE,
    do.stack = FALSE
  )
```

```
## Import rcp4.5_2070 period climate data into R environment
rcp4.5_bio_2070_miroc <- stack(
  c(
    bio_5 = ("C:/Users/Hp/Desktop/sdm_data/Layers/Wordclim_v1.4/quercus_Extent_Layer/MIROC-ESM_2070_rcp4.5_2.5/bio5.tif"),
    bio_7 = ("C:/Users/Hp/Desktop/sdm_data/Layers/Wordclim_v1.4/quercus_Extent_Layer/MIROC-ESM_2070_rcp4.5_2.5/bio7.tif"),
    bio_8 = ("C:/Users/Hp/Desktop/sdm_data/Layers/Wordclim_v1.4/quercus_Extent_Layer/MIROC-ESM_2070_rcp4.5_2.5/bio8.tif"),
    bio_11=("C:/Users/Hp/Desktop/sdm_data/Layers/Wordclim_v1.4/quercus_Extent_Layer/MIROC-ESM_2070_rcp4.5_2.5/bio11.tif"),
    bio_15=("C:/Users/Hp/Desktop/sdm_data/Layers/Wordclim_v1.4/quercus_Extent_Layer/MIROC-ESM_2070_rcp4.5_2.5/bio15.tif"),
    bio_16=("C:/Users/Hp/Desktop/sdm_data/Layers/Wordclim_v1.4/quercus_Extent_Layer/MIROC-ESM_2070_rcp4.5_2.5/bio16.tif"),
    bio_17=("C:/Users/Hp/Desktop/sdm_data/Layers/Wordclim_v1.4/quercus_Extent_Layer/MIROC-ESM_2070_rcp4.5_2.5/bio17.tif")
  )
)


## rcp4.5_2070 projections
quercusprojrcp4.5_2070_miroc <-
  BIOMOD_Projection(
    modeling.output = quercus_model_output,
    new.env = rcp4.5_bio_2070_miroc,
    proj.name = "rcp4.5_2070_miroc",
    selected.models = "all",
    binary.meth = "TSS",
    clamping.mask = TRUE,
    do.stack = FALSE
  )


## Import rcp8.5_2070 period climate data into R environment
rcp8.5_bio_2070_miroc <- stack(
  c(
    bio_5 = ("C:/Users/Hp/Desktop/sdm_data/Layers/Wordclim_v1.4/Quercus_Extent_Layer/MIROC-ESM_2070_rcp8.5_2.5/bio5.tif"),
    bio_7 = ("C:/Users/Hp/Desktop/sdm_data/Layers/Wordclim_v1.4/Quercus_Extent_Layer/MIROC-ESM_2070_rcp8.5_2.5/bio7.tif"),
    bio_8 = ("C:/Users/Hp/Desktop/sdm_data/Layers/Wordclim_v1.4/Quercus_Extent_Layer/MIROC-ESM_2070_rcp8.5_2.5/bio8.tif"),
    bio_11=("C:/Users/Hp/Desktop/sdm_data/Layers/Wordclim_v1.4/Quercus_Extent_Layer/MIROC-ESM_2070_rcp8.5_2.5/bio11.tif"),
    bio_15=("C:/Users/Hp/Desktop/sdm_data/Layers/Wordclim_v1.4/Quercus_Extent_Layer/MIROC-ESM_2070_rcp8.5_2.5/bio15.tif"),
    bio_16=("C:/Users/Hp/Desktop/sdm_data/Layers/Wordclim_v1.4/Quercus_Extent_Layer/MIROC-ESM_2070_rcp8.5_2.5/bio16.tif"),
    bio_17=("C:/Users/Hp/Desktop/sdm_data/Layers/Wordclim_v1.4/Quercus_Extent_Layer/MIROC-ESM_2070_rcp8.5_2.5/bio17.tif")
  )
)
```

```
## rcp8.5_2070 projections
quercusprojrcp8.5_2070_miroc <-
  BIOMOD_Projection(
    modeling.output = quercus_model_output,
    new.env = rcp8.5_bio_2070_miroc,
    proj.name = "rcp8.5_2070_miroc",
    selected.models = "all",
    binary.meth = "TSS",
    clamping.mask = TRUE,
    do.stack = FALSE
  )
```

S1 Table. All occurrence records (1606) used in the modeling process (Browicz & Zieliński, 1982; Hedge & Yaltirik, 1982; Akkemik et al., 2021; Global Biodiversity Information Facility (GBIF), 2022; including herbarium specimens).

species	Longitude	Latitude
Quercus ilex	12.650	43.063
Quercus ilex	14.976	44.461
Quercus ilex	11.164	43.711
Quercus ilex	1.817	41.758
Quercus ilex	11.012	43.437
Quercus ilex	1.788	41.610
Quercus ilex	-2.153	43.288
Quercus ilex	12.059	43.238
Quercus ilex	2.818	41.687
Quercus ilex	4.038	43.731
Quercus ilex	-4.934	43.457
Quercus ilex	11.513	43.187
Quercus ilex	13.641	45.071
Quercus ilex	11.516	43.000
Quercus ilex	5.691	43.673
Quercus ilex	4.227	43.751
Quercus ilex	13.806	44.894
Quercus ilex	5.217	43.800
Quercus ilex	2.699	39.696
Quercus ilex	16.217	43.817
Quercus ilex	6.379	43.742
Quercus ilex	16.595	43.305
Quercus ilex	-2.713	47.579
Quercus ilex	14.125	44.972
Quercus ilex	13.974	44.830
Quercus ilex	17.131	42.955
Quercus ilex	15.414	39.997
Quercus ilex	16.384	43.342
Quercus ilex	13.396	41.847
Quercus ilex	14.720	44.359
Quercus ilex	3.856	43.717
Quercus ilex	-4.801	43.309
Quercus ilex	17.364	42.775
Quercus ilex	12.936	43.251
Quercus ilex	3.181	39.736
Quercus ilex	2.811	39.850
Quercus ilex	2.494	39.537
Quercus ilex	16.653	42.986
Quercus ilex	16.070	43.487
Quercus ilex	9.000	40.071
Quercus ilex	14.732	44.768
Quercus ilex	16.751	43.128
Quercus ilex	3.912	39.937
Quercus ilex	2.097	41.385
Quercus ilex	3.206	41.965
Quercus ilex	11.342	43.320
Quercus ilex	9.349	42.396
Quercus ilex	14.761	44.981
Quercus ilex	2.926	39.524
Quercus ilex	4.403	44.183
Quercus ilex	3.415	43.733
Quercus ilex	2.000	41.646
Quercus ilex	15.769	43.770
Quercus ilex	2.834	42.350
Quercus ilex	12.590	38.037
Quercus ilex	9.965	44.042
Quercus ilex	10.590	43.089
Quercus ilex	2.816	43.200
Quercus ilex	26.977	37.757
Quercus ilex	20.895	37.791
Quercus ilex	13.693	45.735
Quercus ilex	10.833	45.883
Quercus ilex	1.167	45.050
Quercus ilex	-2.410	38.410
Quercus ilex	-4.280	37.370
Quercus ilex	-2.500	43.070
Quercus ilex	6.476	43.216
Quercus ilex	9.321	41.880
Quercus ilex	-1.071	46.121
Quercus ilex	-0.805	46.076
Quercus ilex	7.458	43.764
Quercus ilex	2.849	42.594
Quercus ilex	5.399	43.536
Quercus ilex	4.725	44.503
Quercus ilex	1.536	43.307
Quercus ilex	2.387	43.167
Quercus ilex	4.496	43.752
Quercus ilex	-0.904	45.882
Quercus ilex	7.029	43.628
Quercus ilex	4.076	44.084
Quercus ilex	6.847	43.458
Quercus ilex	6.652	43.429
Quercus ilex	-1.768	46.602
Quercus ilex	4.496	44.378

Quercus ilex	4.115	44.392	*Quercus ilex*	5.817	43.510
Quercus ilex	1.867	43.688	*Quercus ilex*	4.662	43.547
Quercus ilex	6.277	43.374	*Quercus ilex*	4.448	44.555
Quercus ilex	2.089	43.307	*Quercus ilex*	-1.225	45.833
Quercus ilex	9.083	42.270	*Quercus ilex*	4.141	44.234
Quercus ilex	9.119	42.526	*Quercus ilex*	-1.108	44.983
Quercus ilex	8.796	42.128	*Quercus ilex*	1.286	43.558
Quercus ilex	9.186	41.435	*Quercus ilex*	6.975	44.028
Quercus ilex	9.529	42.288	*Quercus ilex*	6.913	43.859
Quercus ilex	8.903	42.519	*Quercus ilex*	4.894	44.965
Quercus ilex	8.935	41.572	*Quercus ilex*	-2.670	43.150
Quercus ilex	9.425	42.686	*Quercus ilex*	-3.420	43.440
Quercus ilex	9.496	42.106	*Quercus ilex*	3.060	42.140
Quercus ilex	8.707	42.408	*Quercus ilex*	-3.440	42.510
Quercus ilex	8.629	42.226	*Quercus ilex*	-3.580	43.200
Quercus ilex	8.983	42.047	*Quercus ilex*	-2.990	42.360
Quercus ilex	8.946	41.788	*Quercus ilex*	-3.420	42.770
Quercus ilex	9.254	41.593	*Quercus ilex*	-3.090	42.760
Quercus ilex	9.437	42.920	*Quercus ilex*	-5.826	39.669
Quercus ilex	8.701	41.935	*Quercus ilex*	2.966	43.578
Quercus ilex	9.293	42.052	*Quercus ilex*	-5.751	39.875
Quercus ilex	9.279	42.220	*Quercus ilex*	-6.264	39.826
Quercus ilex	8.753	41.769	*Quercus ilex*	-3.891	40.509
Quercus ilex	9.396	42.537	*Quercus ilex*	-1.320	42.801
Quercus ilex	9.204	42.672	*Quercus ilex*	4.870	43.700
Quercus ilex	9.116	41.750	*Quercus ilex*	-4.562	43.161
Quercus ilex	4.711	43.936	*Quercus ilex*	5.141	44.357
Quercus ilex	-1.772	47.207	*Quercus ilex*	4.761	44.827
Quercus ilex	-0.741	47.336	*Quercus ilex*	-2.668	41.027
Quercus ilex	-1.261	47.340	*Quercus ilex*	-3.931	38.170
Quercus ilex	-1.517	47.208	*Quercus ilex*	-4.551	36.953
Quercus ilex	6.054	43.581	*Quercus ilex*	-6.048	39.824
Quercus ilex	4.687	44.361	*Quercus ilex*	-5.272	41.396
Quercus ilex	-2.498	47.315	*Quercus ilex*	-1.951	42.669
Quercus ilex	6.087	43.160	*Quercus ilex*	3.716	43.484
Quercus ilex	5.622	43.536	*Quercus ilex*	6.688	43.650
Quercus ilex	5.688	43.311	*Quercus ilex*	2.544	43.360
Quercus ilex	5.106	44.200	*Quercus ilex*	3.843	44.135
Quercus ilex	1.869	43.109	*Quercus ilex*	0.599	42.125
Quercus ilex	-0.653	47.478	*Quercus ilex*	6.378	43.600
Quercus ilex	5.040	43.996	*Quercus ilex*	-2.844	38.936
Quercus ilex	1.541	44.425	*Quercus ilex*	-5.357	39.443
Quercus ilex	1.172	43.358	*Quercus ilex*	-5.084	36.661
Quercus ilex	2.522	42.600	*Quercus ilex*	-5.491	39.168
Quercus ilex	5.958	44.119	*Quercus ilex*	-3.530	42.335
Quercus ilex	5.068	43.646	*Quercus ilex*	-6.255	43.113

Quercus ilex	-2.290	39.420	*Quercus ilex*	-3.060	38.580
Quercus ilex	-2.880	40.510	*Quercus ilex*	-3.340	40.790
Quercus ilex	-4.900	39.600	*Quercus ilex*	-3.480	40.530
Quercus ilex	-4.640	39.850	*Quercus ilex*	-3.560	38.520
Quercus ilex	-4.730	40.060	*Quercus ilex*	-1.520	39.170
Quercus ilex	-3.040	38.310	*Quercus ilex*	-3.930	39.560
Quercus ilex	-4.910	39.310	*Quercus ilex*	-6.530	39.980
Quercus ilex	-4.540	39.150	*Quercus ilex*	-4.940	39.760
Quercus ilex	-4.170	38.700	*Quercus ilex*	-4.850	38.720
Quercus ilex	-5.210	39.640	*Quercus ilex*	-5.150	39.930
Quercus ilex	-4.350	39.860	*Quercus ilex*	-3.500	39.690
Quercus ilex	-2.840	40.320	*Quercus ilex*	-2.840	40.950
Quercus ilex	-2.700	39.550	*Quercus ilex*	-2.910	40.120
Quercus ilex	-3.250	40.500	*Quercus ilex*	-2.420	40.400
Quercus ilex	-3.050	38.890	*Quercus ilex*	-4.060	38.840
Quercus ilex	-3.180	39.980	*Quercus ilex*	-4.500	40.150
Quercus ilex	-4.290	38.840	*Quercus ilex*	-2.640	40.500
Quercus ilex	-2.630	40.740	*Quercus ilex*	-5.040	39.160
Quercus ilex	-3.610	40.670	*Quercus ilex*	-4.050	40.420
Quercus ilex	-3.360	40.160	*Quercus ilex*	-1.720	38.910
Quercus ilex	-2.940	40.750	*Quercus ilex*	-4.600	38.940
Quercus ilex	-5.400	39.800	*Quercus ilex*	-4.300	38.400
Quercus ilex	-3.270	38.540	*Quercus ilex*	-3.870	39.710
Quercus ilex	-4.150	39.690	*Quercus ilex*	-3.230	40.930
Quercus ilex	-4.320	40.010	*Quercus ilex*	-4.290	38.980
Quercus ilex	-2.730	40.030	*Quercus ilex*	-3.470	39.880
Quercus ilex	-1.740	39.440	*Quercus ilex*	-4.300	39.260
Quercus ilex	-2.840	38.600	*Quercus ilex*	-2.570	39.380
Quercus ilex	-3.010	38.730	*Quercus ilex*	-3.010	40.260
Quercus ilex	-6.200	40.260	*Quercus ilex*	-3.610	39.070
Quercus ilex	-2.980	39.900	*Quercus ilex*	-2.150	39.200
Quercus ilex	-2.580	38.380	*Quercus ilex*	-1.320	38.780
Quercus ilex	-4.010	40.280	*Quercus ilex*	-1.600	39.670
Quercus ilex	-4.110	40.050	*Quercus ilex*	-3.080	39.120
Quercus ilex	-4.400	39.460	*Quercus ilex*	-3.770	38.700
Quercus ilex	-3.230	40.330	*Quercus ilex*	-1.300	39.100
Quercus ilex	-4.970	39.980	*Quercus ilex*	-3.600	39.210
Quercus ilex	-3.050	40.400	*Quercus ilex*	-3.900	39.120
Quercus ilex	-2.390	40.770	*Quercus ilex*	-4.060	39.040
Quercus ilex	-4.460	39.630	*Quercus ilex*	-2.720	38.210
Quercus ilex	-3.020	40.990	*Quercus ilex*	-0.860	38.630
Quercus ilex	-2.790	39.290	*Quercus ilex*	-1.940	39.620
Quercus ilex	-2.810	39.780	*Quercus ilex*	-2.100	38.360
Quercus ilex	-3.120	39.400	*Quercus ilex*	-3.680	40.490
Quercus ilex	-3.380	37.720	*Quercus ilex*	-2.630	38.570
Quercus ilex	-3.370	38.990	*Quercus ilex*	-2.370	39.660

Quercus ilex	-2.550	38.100	*Quercus ilex*	3.520	43.890
Quercus ilex	-4.190	40.230	*Quercus ilex*	-3.487	42.171
Quercus ilex	3.280	42.320	*Quercus ilex*	3.020	39.857
Quercus ilex	2.480	42.070	*Quercus ilex*	9.516	40.222
Quercus ilex	-4.535	43.397	*Quercus ilex*	-7.187	39.955
Quercus ilex	10.351	43.665	*Quercus ilex*	-1.645	42.666
Quercus ilex	12.257	44.815	*Quercus ilex*	-9.046	29.779
Quercus ilex	-2.936	43.413	*Quercus ilex*	12.833	37.585
Quercus ilex	5.077	43.398	*Quercus ilex*	-6.736	41.394
Quercus ilex	-1.663	42.812	*Quercus ilex*	2.925	43.399
Quercus ilex	15.979	43.908	*Quercus ilex*	9.244	39.990
Quercus ilex	9.578	40.690	*Quercus ilex*	-2.232	42.643
Quercus ilex	15.009	37.619	*Quercus ilex*	-0.727	42.355
Quercus ilex	10.844	43.683	*Quercus ilex*	-2.646	36.884
Quercus ilex	14.228	40.549	*Quercus ilex*	9.714	40.395
Quercus ilex	1.984	42.110	*Quercus ilex*	-4.101	42.637
Quercus ilex	28.060	36.341	*Quercus ilex*	1.830	43.330
Quercus ilex	13.297	38.215	*Quercus ilex*	-4.012	40.663
Quercus ilex	12.524	42.504	*Quercus ilex*	-1.474	42.487
Quercus ilex	15.968	43.707	*Quercus ilex*	21.722	36.836
Quercus ilex	17.763	40.309	*Quercus ilex*	0.414	40.404
Quercus ilex	1.485	38.932	*Quercus ilex*	3.186	39.430
Quercus ilex	25.912	36.845	*Quercus ilex*	-5.544	40.549
Quercus ilex	3.664	43.639	*Quercus ilex*	-1.835	42.929
Quercus ilex	16.812	42.766	*Quercus ilex*	-2.911	37.903
Quercus ilex	-5.567	42.581	*Quercus ilex*	-2.220	37.500
Quercus ilex	8.663	44.416	*Quercus ilex*	10.212	42.733
Quercus ilex	1.754	41.250	*Quercus ilex*	14.527	44.962
Quercus ilex	12.248	42.490	*Quercus ilex*	12.804	41.966
Quercus ilex	12.335	45.095	*Quercus ilex*	14.516	44.610
Quercus ilex	2.257	42.081	*Quercus ilex*	17.013	43.294
Quercus ilex	15.147	44.197	*Quercus ilex*	13.906	42.047
Quercus ilex	16.086	41.742	*Quercus ilex*	24.912	35.167
Quercus ilex	18.051	42.686	*Quercus ilex*	11.518	42.721
Quercus ilex	4.541	43.954	*Quercus ilex*	14.904	44.161
Quercus ilex	13.606	45.316	*Quercus ilex*	14.353	45.111
Quercus ilex	-3.271	36.993	*Quercus ilex*	15.448	43.933
Quercus ilex	-5.585	33.548	*Quercus ilex*	14.325	44.873
Quercus ilex	-1.694	42.502	*Quercus ilex*	1.544	43.028
Quercus ilex	-4.775	37.905	*Quercus ilex*	1.985	42.969
Quercus ilex	-1.154	42.661	*Quercus ilex*	1.350	43.044
Quercus ilex	-5.645	43.535	*Quercus ilex*	1.689	44.039
Quercus ilex	2.585	42.831	*Quercus ilex*	2.104	43.467
Quercus ilex	8.308	40.586	*Quercus ilex*	3.048	43.088
Quercus ilex	-4.856	37.248	*Quercus ilex*	1.364	43.704
Quercus ilex	-4.826	36.672	*Quercus ilex*	0.960	44.338

Quercus ilex	0.635	43.668	*Quercus ilex*	1.631	44.851
Quercus ilex	1.958	43.913	*Quercus ilex*	6.246	44.100
Quercus ilex	1.474	43.539	*Quercus ilex*	5.326	44.183
Quercus ilex	1.635	42.833	*Quercus ilex*	7.172	43.828
Quercus ilex	3.029	42.497	*Quercus ilex*	3.933	44.428
Quercus ilex	2.816	42.820	*Quercus ilex*	0.680	43.240
Quercus ilex	3.607	44.103	*Quercus ilex*	-2.313	47.549
Quercus ilex	3.795	43.875	*Quercus ilex*	4.789	44.196
Quercus ilex	5.876	43.843	*Quercus ilex*	4.821	44.645
Quercus ilex	2.671	42.453	*Quercus ilex*	5.117	44.512
Quercus ilex	6.125	43.728	*Quercus ilex*	4.577	44.848
Quercus ilex	5.389	43.799	*Quercus ilex*	-1.516	46.461
Quercus ilex	5.379	43.950	*Quercus ilex*	4.532	44.993
Quercus ilex	5.586	43.919	*Quercus ilex*	2.376	43.486
Quercus ilex	2.157	42.881	*Quercus ilex*	5.046	43.812
Quercus ilex	7.575	44.019	*Quercus ilex*	2.497	42.408
Quercus ilex	7.280	44.044	*Quercus ilex*	4.168	43.921
Quercus ilex	-0.612	45.860	*Quercus ilex*	2.351	42.804
Quercus ilex	4.232	44.533	*Quercus ilex*	1.130	44.649
Quercus ilex	5.244	45.057	*Quercus ilex*	0.733	44.859
Quercus ilex	0.978	44.898	*Quercus ilex*	-1.276	45.983
Quercus ilex	2.576	43.037	*Quercus ilex*	2.721	43.450
Quercus ilex	5.796	43.139	*Quercus ilex*	-1.130	45.321
Quercus ilex	0.686	45.259	*Quercus ilex*	9.159	41.918
Quercus ilex	3.997	43.918	*Quercus ilex*	3.841	44.285
Quercus ilex	-1.311	47.104	*Quercus ilex*	2.216	43.127
Quercus ilex	-0.792	45.616	*Quercus ilex*	3.120	43.717
Quercus ilex	1.326	44.823	*Quercus ilex*	6.199	43.957
Quercus ilex	1.541	44.697	*Quercus ilex*	2.290	43.325
Quercus ilex	2.770	42.959	*Quercus ilex*	2.339	42.965
Quercus ilex	-1.042	44.649	*Quercus ilex*	4.243	44.693
Quercus ilex	5.113	44.811	*Quercus ilex*	5.233	43.592
Quercus ilex	1.204	45.229	*Quercus ilex*	9.355	41.743
Quercus ilex	-2.049	46.802	*Quercus ilex*	-1.245	44.525
Quercus ilex	-1.065	45.181	*Quercus ilex*	4.845	45.185
Quercus ilex	3.300	43.555	*Quercus ilex*	6.471	43.425
Quercus ilex	5.952	43.363	*Quercus ilex*	0.891	44.142
Quercus ilex	-0.372	47.460	*Quercus ilex*	-1.101	45.664
Quercus ilex	5.572	44.354	*Quercus ilex*	5.976	43.983
Quercus ilex	0.245	47.172	*Quercus ilex*	3.037	44.078
Quercus ilex	4.590	44.209	*Quercus ilex*	2.987	42.909
Quercus ilex	5.335	43.371	*Quercus ilex*	6.862	43.680
Quercus ilex	4.902	44.413	*Quercus ilex*	5.416	43.233
Quercus ilex	3.467	43.317	*Quercus ilex*	3.291	43.262
Quercus ilex	2.337	42.578	*Quercus ilex*	3.089	43.232
Quercus ilex	-2.473	41.351	*Quercus ilex*	3.488	43.516

Quercus ilex	8.874	42.279	*Quercus ilex*	1.527	42.257
Quercus ilex	1.151	44.811	*Quercus ilex*	10.608	43.767
Quercus ilex	1.401	44.975	*Quercus ilex*	8.978	40.614
Quercus ilex	-1.126	44.835	*Quercus ilex*	9.043	40.234
Quercus ilex	1.307	43.212	*Quercus ilex*	18.168	40.357
Quercus ilex	4.368	43.953	*Quercus ilex*	23.965	35.413
Quercus ilex	5.868	43.645	*Quercus ilex*	8.991	39.705
Quercus ilex	1.307	44.578	*Quercus ilex*	14.618	37.147
Quercus ilex	5.682	44.064	*Quercus ilex*	-8.004	37.201
Quercus ilex	5.058	45.108	*Quercus ilex*	14.295	45.319
Quercus ilex	6.260	43.217	*Quercus ilex*	23.931	39.241
Quercus ilex	2.609	43.181	*Quercus ilex*	13.883	45.379
Quercus ilex	-2.905	47.614	*Quercus ilex*	10.288	43.998
Quercus ilex	1.836	44.582	*Quercus ilex*	0.547	40.642
Quercus ilex	3.116	43.430	*Quercus ilex*	22.315	40.210
Quercus ilex	-1.080	47.394	*Quercus ilex*	12.369	44.243
Quercus ilex	1.071	43.893	*Quercus ilex*	16.708	40.880
Quercus ilex	-1.798	46.740	*Quercus ilex*	20.683	38.717
Quercus ilex	-2.318	47.296	*Quercus ilex*	10.884	42.365
Quercus ilex	4.307	44.368	*Quercus ilex*	14.526	40.621
Quercus ilex	-0.893	46.499	*Quercus ilex*	-4.027	43.435
Quercus ilex	6.501	43.972	*Quercus ilex*	10.264	43.819
Quercus ilex	13.994	45.665	*Quercus ilex*	21.362	39.015
Quercus ilex	17.415	40.703	*Quercus ilex*	10.670	45.693
Quercus ilex	22.460	40.074	*Quercus ilex*	16.292	39.603
Quercus ilex	2.526	39.685	*Quercus ilex*	10.450	43.509
Quercus ilex	13.585	43.523	*Quercus ilex*	11.470	43.703
Quercus ilex	14.027	38.000	*Quercus ilex*	8.474	44.430
Quercus ilex	12.804	45.565	*Quercus ilex*	10.627	43.453
Quercus ilex	12.100	42.006	*Quercus ilex*	11.298	43.091
Quercus ilex	9.726	44.107	*Quercus ilex*	16.263	39.845
Quercus ilex	15.167	43.940	*Quercus ilex*	10.448	43.870
Quercus ilex	-0.987	47.205	*Quercus ilex*	2.451	42.259
Quercus ilex	11.282	42.420	*Quercus ilex*	8.932	44.435
Quercus ilex	23.735	37.975	*Quercus ilex*	13.603	45.538
Quercus ilex	20.840	39.615	*Quercus ilex*	12.069	43.020
Quercus ilex	2.355	41.823	*Quercus ilex*	17.569	42.724
Quercus ilex	11.133	42.754	*Quercus ilex*	12.438	41.884
Quercus ilex	1.881	42.263	*Quercus ilex*	12.875	35.858
Quercus ilex	12.691	42.302	*Quercus ilex*	12.336	37.998
Quercus ilex	2.324	41.527	*Quercus ilex*	13.232	38.015
Quercus ilex	-5.828	43.387	*Quercus ilex*	13.259	37.663
Quercus ilex	11.589	43.377	*Quercus ilex*	15.000	37.130
Quercus ilex	1.551	41.620	*Quercus ilex*	13.734	37.944
Quercus ilex	8.892	40.888	*Quercus ilex*	13.403	38.075
Quercus ilex	10.422	42.781	*Quercus ilex*	13.377	37.883

Quercus ilex	11.921	36.787	*Quercus ilex*	3.283	44.147
Quercus ilex	13.159	38.706	*Quercus ilex*	0.784	44.515
Quercus ilex	14.931	38.488	*Quercus ilex*	4.785	45.502
Quercus ilex	12.057	37.970	*Quercus ilex*	0.090	47.385
Quercus ilex	13.417	37.650	*Quercus ilex*	-1.862	46.902
Quercus ilex	14.000	37.850	*Quercus ilex*	-1.120	46.383
Quercus ilex	12.858	38.004	*Quercus ilex*	1.850	43.516
Quercus ilex	13.605	37.622	*Quercus ilex*	1.646	43.794
Quercus ilex	15.270	40.228	*Quercus ilex*	0.680	43.026
Quercus ilex	9.096	41.094	*Quercus ilex*	-1.055	45.459
Quercus ilex	14.669	37.926	*Quercus ilex*	-1.613	46.927
Quercus ilex	15.533	38.210	*Quercus ilex*	0.001	47.193
Quercus ilex	14.202	40.859	*Quercus ilex*	-1.500	47.362
Quercus ilex	15.770	39.910	*Quercus ilex*	1.148	44.407
Quercus ilex	10.915	42.893	*Quercus ilex*	0.931	43.187
Quercus ilex	9.301	44.325	*Quercus ilex*	-0.348	47.799
Quercus ilex	7.779	43.857	*Quercus ilex*	-1.329	46.700
Quercus ilex	9.624	44.261	*Quercus ilex*	23.816	40.185
Quercus ilex	8.338	44.212	*Quercus ilex*	1.917	41.416
Quercus ilex	9.116	44.400	*Quercus ilex*	2.741	41.949
Quercus ilex	11.869	43.571	*Quercus ilex*	26.402	39.096
Quercus ilex	11.471	43.519	*Quercus ilex*	13.237	41.603
Quercus ilex	11.655	43.510	*Quercus ilex*	1.056	41.338
Quercus ilex	11.857	43.432	*Quercus ilex*	2.765	42.118
Quercus ilex	10.905	43.821	*Quercus ilex*	-2.688	43.388
Quercus ilex	10.490	42.953	*Quercus ilex*	12.247	44.672
Quercus ilex	10.544	44.017	*Quercus ilex*	1.689	45.060
Quercus ilex	10.979	43.145	*Quercus ilex*	1.360	44.084
Quercus ilex	10.792	43.125	*Quercus ilex*	0.823	44.676
Quercus ilex	11.460	42.476	*Quercus ilex*	1.327	44.367
Quercus ilex	10.744	42.963	*Quercus ilex*	-0.993	46.686
Quercus ilex	12.291	43.760	*Quercus ilex*	-2.064	47.097
Quercus ilex	11.094	42.892	*Quercus ilex*	-0.533	47.287
Quercus ilex	8.859	39.194	*Quercus ilex*	21.254	39.329
Quercus ilex	9.058	40.442	*Quercus ilex*	21.165	39.166
Quercus ilex	2.358	41.669	*Quercus ilex*	12.753	42.725
Quercus ilex	1.621	41.796	*Quercus ilex*	15.785	38.263
Quercus ilex	14.254	44.642	*Quercus ilex*	-0.954	41.140
Quercus ilex	-6.970	38.880	*Quercus ilex*	26.691	37.751
Quercus ilex	-6.630	38.910	*Quercus ilex*	10.773	43.307
Quercus ilex	12.980	41.860	*Quercus ilex*	22.980	36.597
Quercus ilex	-5.490	34.030	*Quercus ilex*	10.573	45.558
Quercus ilex	0.740	41.980	*Quercus ilex*	-2.739	42.897
Quercus ilex	0.495	45.457	*Quercus ilex*	23.735	38.169
Quercus ilex	2.884	43.848	*Quercus ilex*	3.370	39.660
Quercus ilex	4.994	45.842	*Quercus ilex*	0.220	40.660

Quercus ilex	0.800	41.280	*Quercus ilex*	12.504	43.577
Quercus ilex	-5.390	43.500	*Quercus ilex*	12.301	44.431
Quercus ilex	27.215	36.854	*Quercus ilex*	-4.757	41.644
Quercus ilex	14.781	41.123	*Quercus ilex*	-2.430	42.456
Quercus ilex	23.636	39.179	*Quercus ilex*	15.504	44.213
Quercus ilex	17.554	40.322	*Quercus ilex*	-2.775	42.616
Quercus ilex	2.531	41.611	*Quercus ilex*	12.612	43.861
Quercus ilex	19.687	39.678	*Quercus ilex*	14.127	42.424
Quercus ilex	-6.436	41.562	*Quercus ilex*	13.447	43.298
Quercus ilex	16.140	38.330	*Quercus ilex*	-5.637	40.964
Quercus ilex	25.490	35.200	*Quercus ilex*	11.225	43.479
Quercus ilex	16.436	38.467	*Quercus ilex*	14.273	41.140
Quercus ilex	18.435	40.269	*Quercus ilex*	12.047	44.215
Quercus ilex	14.538	42.224	*Quercus ilex*	15.041	37.989
Quercus ilex	-2.148	42.967	*Quercus ilex*	18.219	42.613
Quercus ilex	-2.957	42.988	*Quercus ilex*	15.196	40.521
Quercus ilex	-3.060	43.220	*Quercus ilex*	0.867	41.051
Quercus ilex	-3.228	43.186	*Quercus ilex*	12.497	41.665
Quercus ilex	-1.240	42.480	*Quercus ilex*	4.932	45.704
Quercus ilex	-2.463	43.347	*Quercus ilex*	9.415	39.665
Quercus ilex	-1.600	42.310	*Quercus ilex*	16.564	38.982
Quercus ilex	18.535	42.450	*Quercus ilex*	-4.480	41.611
Quercus ilex	15.111	41.060	*Quercus ilex*	16.051	40.530
Quercus ilex	13.668	42.773	*Quercus ilex*	18.863	42.282
Quercus ilex	11.925	42.674	*Quercus ilex*	9.496	40.928
Quercus ilex	-3.849	37.771	*Quercus ilex*	17.147	40.916
Quercus ilex	-8.675	39.629	*Quercus ilex*	-3.605	40.021
Quercus ilex	-0.609	38.768	*Quercus ilex*	10.527	43.249
Quercus ilex	-4.547	36.728	*Quercus ilex*	18.035	40.178
Quercus ilex	12.301	42.094	*Quercus ilex*	-0.748	47.007
Quercus ilex	-7.388	42.442	*Quercus ilex*	16.298	38.267
Quercus ilex	9.496	39.943	*Quercus ilex*	16.423	41.280
Quercus ilex	16.239	39.368	*Quercus ilex*	14.319	41.918
Quercus ilex	0.031	40.078	*Quercus ilex*	13.185	43.063
Quercus ilex	22.959	40.639	*Quercus ilex*	17.301	40.467
Quercus ilex	-4.260	40.513	*Quercus ilex*	12.185	42.737
Quercus ilex	11.797	42.312	*Quercus ilex*	-3.822	40.293
Quercus ilex	-2.497	47.518	*Quercus ilex*	0.153	42.382
Quercus ilex	-6.754	42.454	*Quercus ilex*	16.548	43.519
Quercus ilex	16.717	40.416	*Quercus ilex*	-4.113	40.874
Quercus ilex	13.867	42.872	*Quercus ilex*	9.691	39.988
Quercus ilex	4.095	39.986	*Quercus ilex*	13.056	43.518
Quercus ilex	-0.406	39.468	*Quercus ilex*	-0.103	40.335
Quercus ilex	13.013	43.838	*Quercus ilex*	-6.382	39.473
Quercus ilex	-3.653	43.398	*Quercus ilex*	12.970	41.369
Quercus ilex	13.141	41.753	*Quercus ilex*	12.260	45.507

Quercus ilex	8.065	43.926	*Quercus ilex*	-1.420	46.962
Quercus ilex	-2.049	47.515	*Quercus ilex*	13.839	42.694
Quercus ilex	0.165	40.484	*Quercus ilex*	-0.727	42.527
Quercus ilex	11.111	43.901	*Quercus ilex*	14.534	45.123
Quercus ilex	14.475	41.485	*Quercus ilex*	-6.311	37.982
Quercus ilex	-1.795	40.842	*Quercus ilex*	12.357	42.347
Quercus ilex	12.650	41.798	*Quercus ilex*	-2.559	42.303
Quercus ilex	9.088	39.291	*Quercus ilex*	-3.702	42.329
Quercus ilex	15.286	37.068	*Quercus ilex*	14.288	37.270
Quercus ilex	9.307	39.216	*Quercus ilex*	-4.321	40.369
Quercus ilex	-0.511	39.755	*Quercus ilex*	16.295	41.115
Quercus ilex	-1.313	41.056	*Quercus ilex*	13.684	43.296
Quercus ilex	-0.438	39.157	*Quercus ilex*	12.287	43.016
Quercus ilex	19.244	42.446	*Quercus ilex*	8.921	39.441
Quercus ilex	-4.091	41.592	*Quercus ilex*	-5.928	42.304
Quercus ilex	9.349	40.193	*Quercus ilex*	13.254	43.696
Quercus ilex	8.529	39.181	*Quercus ilex*	3.915	43.570
Quercus ilex	18.390	40.008	*Quercus ilex*	16.283	40.403
Quercus ilex	12.125	43.583	*Quercus ilex*	-7.181	40.123
Quercus ilex	-2.434	42.778	*Quercus ilex*	16.479	41.115
Quercus ilex	-1.235	44.695	*Quercus ilex*	2.085	41.818
Quercus ilex	-2.891	43.250	*Quercus ilex*	13.895	40.731
Quercus ilex	-4.781	41.915	*Quercus ilex*	12.770	43.698
Quercus ilex	11.634	42.423	*Quercus ilex*	1.645	43.592
Quercus ilex	-7.102	42.398	*Quercus ilex*	1.738	41.396
Quercus ilex	0.916	45.275	*Quercus ilex*	-5.156	36.878
Quercus ilex	15.006	40.420	*Quercus ilex*	-1.171	40.351
Quercus ilex	16.301	38.652	*Quercus ilex*	-5.685	42.000
Quercus ilex	1.500	42.003	*Quercus ilex*	12.572	44.061
Quercus ilex	23.444	40.379	*Quercus ilex*	12.985	41.550
Quercus ilex	8.513	39.417	*Quercus ilex*	1.457	41.328
Quercus ilex	16.490	39.666	*Quercus ilex*	-1.095	41.718
Quercus ilex	14.448	41.150	*Quercus ilex*	-5.261	36.596
Quercus ilex	-4.461	40.782	*Quercus ilex*	14.564	36.881
Quercus ilex	-3.641	37.160	*Quercus ilex*	15.630	41.496
Quercus ilex	-2.236	37.710	*Quercus ilex*	-5.044	38.968
Quercus ilex	-0.932	46.890	*Quercus ilex*	-5.045	41.883
Quercus ilex	21.635	39.713	*Quercus ilex*	8.721	39.463
Quercus ilex	-2.368	41.028	*Quercus ilex*	-0.402	42.145
Quercus ilex	12.936	43.673	*Quercus ilex*	15.796	40.649
Quercus ilex	-1.804	41.839	*Quercus ilex*	20.675	38.421
Quercus ilex	-1.195	42.271	*Quercus ilex*	-6.345	38.560
Quercus ilex	1.333	41.178	*Quercus ilex*	1.497	44.277
Quercus ilex	16.692	41.109	*Quercus ilex*	0.183	41.902
Quercus ilex	1.084	41.661	*Quercus ilex*	-2.497	41.993
Quercus ilex	13.278	45.823	*Quercus ilex*	3.053	41.800

Quercus ilex	12.408	42.784	*Quercus ilex*	-5.818	41.709
Quercus ilex	14.843	40.708	*Quercus ilex*	-3.730	40.950
Quercus ilex	14.805	40.914	*Quercus ilex*	-4.658	40.654
Quercus ilex	-2.141	40.073	*Quercus ilex*	-4.789	42.283
Quercus ilex	15.474	40.987	*Quercus ilex*	4.192	43.567
Quercus ilex	2.983	39.693	*Quercus ilex*	14.961	41.689
Quercus ilex	25.656	35.178	*Quercus ilex*	16.123	38.082
Quercus ilex	-4.302	43.226	*Quercus ilex*	-4.550	41.975
Quercus ilex	-0.044	40.802	*Quercus ilex*	-0.819	42.153
Quercus ilex	-5.876	38.183	*Quercus ilex*	13.994	40.970
Quercus ilex	-0.189	38.962	*Quercus ilex*	12.134	42.160
Quercus ilex	-1.507	46.725	*Quercus ilex*	-7.193	37.975
Quercus ilex	8.707	39.046	*Quercus ilex*	4.606	45.843
Quercus ilex	17.707	40.656	*Quercus ilex*	0.639	41.623
Quercus ilex	-3.611	42.026	*Quercus ilex*	2.643	41.790
Quercus ilex	12.836	43.499	*Quercus ilex*	0.307	41.066
Quercus ilex	-5.338	42.771	*Quercus ilex*	-0.948	47.027
Quercus ilex	13.551	37.976	*Quercus ilex*	-3.603	38.298
Quercus ilex	13.012	37.681	*Quercus ilex*	-7.045	38.583
Quercus ilex	17.918	40.449	*Quercus ilex*	-4.868	39.097
Quercus ilex	14.244	37.609	*Quercus ilex*	-3.515	40.323
Quercus ilex	13.474	42.306	*Quercus ilex*	15.233	40.816
Quercus ilex	-6.725	42.600	*Quercus ilex*	-0.422	40.112
Quercus ilex	15.650	40.334	*Quercus ilex*	-5.068	40.159
Quercus ilex	-5.516	40.828	*Quercus ilex*	-3.520	41.359
Quercus ilex	-5.538	36.756	*Quercus ilex*	-7.393	38.439
Quercus ilex	19.175	42.209	*Quercus ilex*	-0.526	42.687
Quercus ilex	16.687	40.709	*Quercus ilex*	-5.809	42.649
Quercus ilex	-7.261	40.967	*Quercus ilex*	-3.245	43.397
Quercus ilex	-3.953	41.410	*Quercus ilex*	-1.014	39.466
Quercus ilex	-4.787	37.052	*Quercus ilex*	-4.073	41.046
Quercus ilex	13.654	41.286	*Quercus ilex*	14.426	40.793
Quercus ilex	0.821	41.787	*Quercus ilex*	-2.991	41.829
Quercus ilex	12.999	45.631	*Quercus ilex*	-7.510	40.293
Quercus ilex	0.171	42.223	*Quercus ilex*	-3.409	38.023
Quercus ilex	9.595	39.398	*Quercus ilex*	-3.752	42.775
Quercus ilex	14.729	42.093	*Quercus ilex*	0.952	41.519
Quercus ilex	-0.920	41.618	*Quercus ilex*	-2.091	41.784
Quercus ilex	-0.198	38.677	*Quercus ilex*	-2.115	42.084
Quercus ilex	16.759	40.564	*Quercus ilex*	-4.420	41.830
Quercus ilex	-3.662	43.034	*Quercus ilex*	3.004	41.977
Quercus ilex	1.284	41.792	*Quercus ilex*	0.141	42.597
Quercus ilex	14.465	41.656	*Quercus ilex*	-5.196	41.056
Quercus ilex	-4.990	36.496	*Quercus ilex*	-4.112	41.848
Quercus ilex	12.092	42.346	*Quercus ilex*	-1.974	43.266
Quercus ilex	-0.087	40.598	*Quercus ilex*	0.833	43.429

Quercus ilex	-2.386	36.835	*Quercus ilex*	-5.740	41.502
Quercus ilex	1.115	41.081	*Quercus ilex*	-6.904	40.416
Quercus ilex	-4.961	41.145	*Quercus ilex*	-5.951	43.533
Quercus ilex	-3.163	41.851	*Quercus ilex*	15.290	37.854
Quercus ilex	14.559	41.284	*Quercus ilex*	-5.378	36.754
Quercus ilex	16.818	43.336	*Quercus ilex*	-3.060	40.579
Quercus ilex	-0.175	42.218	*Quercus ilex*	16.911	41.064
Quercus ilex	8.748	39.667	*Quercus ilex*	-7.579	39.679
Quercus ilex	1.263	42.153	*Quercus ilex*	-3.192	42.419
Quercus ilex	12.452	43.930	*Quercus ilex*	14.438	37.764
Quercus ilex	9.835	43.040	*Quercus ilex*	22.728	37.635
Quercus ilex	15.341	41.250	*Quercus ilex*	-1.866	38.103
Quercus ilex	1.231	42.575	*Quercus ilex*	-4.530	42.833
Quercus ilex	17.321	42.940	*Quercus ilex*	-6.410	38.942
Quercus ilex	-1.641	42.111	*Quercus ilex*	1.085	42.114
Quercus ilex	-4.832	38.098	*Quercus ilex*	-0.893	39.655
Quercus ilex	-3.947	36.873	*Quercus ilex*	-0.773	39.238
Quercus ilex	-6.470	37.687	*Quercus ilex*	-2.723	41.480
Quercus ilex	-3.466	36.909	*Quercus ilex*	-5.266	42.489
Quercus ilex	-4.337	36.949	*Quercus ilex*	-6.180	40.786
Quercus ilex	-6.014	40.142	*Quercus ilex*	8.530	39.929
Quercus ilex	-3.940	43.249	*Quercus ilex*	1.292	41.360
Quercus ilex	25.127	39.992	*Quercus ilex*	13.600	37.301
Quercus ilex	-6.163	41.215	*Quercus ilex*	11.707	43.743
Quercus ilex	-1.501	40.889	*Quercus ilex*	9.256	41.005
Quercus ilex	-7.469	37.347	*Quercus ilex*	11.112	42.410
Quercus ilex	-6.182	39.461	*Quercus ilex*	-3.775	40.652
Quercus ilex	0.748	42.320	*Quercus ilex*	-0.282	47.201
Quercus ilex	20.367	39.292	*Quercus ilex*	11.868	42.991
Quercus ilex	-3.052	37.163	*Quercus ilex*	-6.895	40.186
Quercus ilex	-8.084	37.061	*Quercus ilex*	17.642	40.498
Quercus ilex	-4.306	37.140	*Quercus ilex*	-4.695	41.468
Quercus ilex	0.493	42.383	*Quercus ilex*	0.557	41.881
Quercus ilex	26.269	39.377	*Quercus ilex*	-5.814	43.127
Quercus ilex	-4.026	37.497	*Quercus ilex*	-1.977	39.436
Quercus ilex	-4.315	37.540	*Quercus ilex*	-2.467	41.757
Quercus ilex	-2.191	37.039	*Quercus ilex*	0.151	40.910
Quercus ilex	1.450	38.710	*Quercus ilex*	-2.990	38.038
Quercus ilex	1.450	39.070	*Quercus ilex*	-3.610	37.771
Quercus ilex	-5.253	37.838	*Quercus ilex*	-3.483	41.629
Quercus ilex	-4.620	42.690	*Quercus ilex*	12.799	37.826
Quercus ilex	18.173	40.018	*Quercus ilex*	15.707	41.708
Quercus ilex	8.217	40.774	*Quercus ilex*	14.443	35.886
Quercus ilex	8.564	40.747	*Quercus ilex*	-5.176	37.017
Quercus ilex	15.968	41.901	*Quercus ilex*	-2.039	42.236
Quercus ilex	11.694	42.626	*Quercus ilex*	-3.839	43.423

Quercus ilex	1.961	42.818	*Quercus ilex*	2.127	43.650
Quercus ilex	-0.360	47.641	*Quercus ilex*	13.886	42.364
Quercus ilex	-5.091	38.457	*Quercus ilex*	-0.924	42.377
Quercus ilex	17.307	40.875	*Quercus ilex*	-2.353	37.983
Quercus ilex	-8.317	37.115	*Quercus ilex*	-5.032	42.091
Quercus ilex	-3.147	41.612	*Quercus ilex*	-6.520	42.626
Quercus ilex	-5.594	38.767	*Quercus ilex*	24.670	35.324
Quercus ilex	13.529	45.805	*Quercus ilex*	-0.236	39.868
Quercus ilex	19.802	41.328	*Quercus ilex*	-6.174	37.732
Quercus ilex	14.254	42.096	*Quercus ilex*	-1.170	46.849
Quercus ilex	0.032	42.081	*Quercus ilex*	-8.433	38.405
Quercus ilex	17.113	40.602	*Quercus ilex*	16.736	39.244
Quercus ilex	13.195	41.438	*Quercus ilex*	-1.530	41.830
Quercus ilex	8.557	40.475	*Quercus ilex*	15.116	37.309
Quercus ilex	17.401	43.079	*Quercus ilex*	17.942	40.622
Quercus ilex	-4.731	36.905	*Quercus ilex*	-1.043	38.409
Quercus ilex	-6.964	41.720	*Quercus ilex*	14.444	42.068
Quercus ilex	13.709	43.159	*Quercus ilex*	-5.782	41.331
Quercus ilex	9.377	41.180	*Quercus ilex*	-1.794	41.272
Quercus ilex	15.330	41.512	*Quercus ilex*	-5.430	36.965
Quercus ilex	-6.626	39.173	*Quercus ilex*	13.703	41.550
Quercus ilex	8.555	40.213	*Quercus ilex*	12.871	42.226
Quercus ilex	4.674	45.272	*Quercus ilex*	-8.013	38.537
Quercus ilex	14.251	41.365	*Quercus ilex*	11.827	43.149
Quercus ilex	-2.459	37.836	*Quercus ilex*	-4.560	40.286
Quercus ilex	-3.483	42.933	*Quercus ilex*	-1.242	46.537
Quercus ilex	-8.036	37.678	*Quercus ilex*	16.202	43.024
Quercus ilex	-7.449	37.963	*Quercus ilex*	20.765	38.163
Quercus ilex	-4.183	42.941	*Quercus ilex*	0.576	40.945
Quercus ilex	9.785	40.552	*Quercus ilex*	13.698	42.326
Quercus ilex	9.366	40.403	*Quercus ilex*	1.050	42.304
Quercus ilex	9.169	40.853	*Quercus ilex*	0.009	38.864
Quercus ilex	16.669	40.217	*Quercus ilex*	14.597	41.032
Quercus ilex	-2.933	37.537	*Quercus ilex*	-2.848	36.861
Quercus ilex	-8.500	39.662	*Quercus ilex*	23.664	35.412
Quercus ilex	-7.077	41.033	*Quercus ilex*	12.950	42.386
Quercus ilex	17.649	43.059	*Quercus ilex*	13.531	43.104
Quercus ilex	-4.706	37.401	*Quercus ilex*	-5.613	42.741
Quercus ilex	-4.310	41.401	*Quercus ilex*	-1.518	41.088
Quercus ilex	-2.121	42.787	*Quercus ilex*	0.258	42.051
Quercus ilex	1.776	42.024	*Quercus ilex*	-5.223	42.266
Quercus ilex	-4.140	42.289	*Quercus ilex*	-3.252	40.654
Quercus ilex	-3.568	41.192	*Quercus ilex*	-3.487	41.825
Quercus ilex	-5.179	40.360	*Quercus ilex*	-5.036	37.831
Quercus ilex	-5.464	40.112	*Quercus ilex*	7.242	43.690
Quercus ilex	8.772	40.174	*Quercus ilex*	13.494	42.881

Quercus ilex	13.437	42.003	*Quercus ilex*	-6.646	38.208
Quercus ilex	-6.136	42.401	*Quercus ilex*	-4.499	37.288
Quercus ilex	12.433	37.802	*Quercus ilex*	-0.835	40.133
Quercus ilex	5.324	44.783	*Quercus ilex*	-3.002	37.394
Quercus ilex	17.325	43.468	*Quercus ilex*	21.649	37.481
Quercus ilex	12.583	43.337	*Quercus ilex*	-1.522	37.868
Quercus ilex	14.994	36.903	*Quercus ilex*	15.941	39.897
Quercus ilex	-6.949	37.971	*Quercus ilex*	-5.655	40.129
Quercus ilex	0.726	40.722	*Quercus ilex*	-3.354	39.374
Quercus ilex	-5.978	37.703	*Quercus ilex*	-6.124	37.917
Quercus ilex	5.317	44.369	*Quercus ilex*	5.008	45.517
Quercus ilex	8.108	44.132	*Quercus ilex*	2.694	39.554
Quercus ilex	23.363	38.834	*Quercus ilex*	24.151	41.151
Quercus ilex	-4.490	41.154	*Quercus ilex*	13.698	42.945
Quercus ilex	1.096	41.977	*Quercus ilex*	21.947	38.301
Quercus ilex	12.648	42.156	*Quercus ilex*	-6.434	41.012
Quercus ilex	-0.873	47.518	*Quercus ilex*	10.917	43.956
Quercus ilex	13.747	41.689	*Quercus ilex*	-7.751	38.830
Quercus ilex	-4.657	39.566	*Quercus ilex*	-4.368	38.668
Quercus ilex	-4.909	41.304	*Quercus ilex*	12.462	42.977
Quercus ilex	15.802	41.106	*Quercus ilex*	-5.705	40.696
Quercus ilex	-5.891	40.765	*Quercus ilex*	14.778	38.070
Quercus ilex	-8.633	38.757	*Quercus ilex*	-2.790	41.173
Quercus ilex	16.067	41.302	*Quercus ilex*	-7.096	39.435
Quercus ilex	-3.664	37.300	*Quercus ilex*	13.236	43.320
Quercus ilex	11.960	42.513	*Quercus ilex*	-0.585	38.619
Quercus ilex	-6.396	41.286	*Quercus ilex*	4.684	43.754
Quercus ilex	-6.197	41.531	*Quercus ilex*	-3.192	39.737
Quercus ilex	11.099	42.606	*Quercus ilex*	-1.625	41.539
Quercus ilex	-2.043	37.563	*Quercus ilex*	-4.522	41.409
Quercus ilex	-2.266	37.232	*Quercus ilex*	21.128	39.499
Quercus ilex	-0.741	39.983	*Quercus ilex*	-2.281	41.164
Quercus ilex	-2.289	40.558	*Quercus ilex*	13.543	37.479
Quercus ilex	13.889	41.303	*Quercus ilex*	-0.468	38.940
Quercus ilex	14.183	41.791	*Quercus ilex*	-3.913	40.023
Quercus ilex	-0.416	38.780	*Quercus ilex*	-6.819	41.562
Quercus ilex	-6.416	38.415	*Quercus ilex*	-5.464	40.328
Quercus ilex	-4.033	39.871	*Quercus ilex*	-5.755	37.927
Quercus ilex	-0.354	42.490	*Quercus ilex*	-6.912	40.707
Quercus ilex	-4.686	41.286	*Quercus ilex*	-3.619	38.136
Quercus ilex	-3.866	41.113	*Quercus ilex*	-0.794	40.272
Quercus ilex	-2.889	37.010	*Quercus ilex*	12.133	44.475
Quercus ilex	-1.158	40.042	*Quercus ilex*	-0.695	39.778
Quercus ilex	15.544	40.154	*Quercus ilex*	22.695	37.807
Quercus ilex	13.431	41.355	*Quercus ilex*	22.885	39.683
Quercus ilex	-0.569	39.543	*Quercus ilex*	11.305	43.859

Quercus ilex	-4.255	43.384	*Quercus ilex*	-0.413	39.297
Quercus ilex	-6.677	41.216	*Quercus ilex*	-6.104	38.593
Quercus ilex	-4.276	41.630	*Quercus ilex*	16.923	40.793
Quercus ilex	-3.250	37.192	*Quercus ilex*	-6.527	38.587
Quercus ilex	-7.396	39.634	*Quercus ilex*	-8.659	37.143
Quercus ilex	-0.386	40.776	*Quercus ilex*	-4.556	40.431
Quercus ilex	16.494	40.249	*Quercus ilex*	-2.464	42.604
Quercus ilex	-0.738	41.741	*Quercus ilex*	-5.679	38.260
Quercus ilex	-4.523	38.162	*Quercus ilex*	22.382	39.627
Quercus ilex	0.512	45.029	*Quercus ilex*	18.362	39.846
Quercus ilex	9.972	44.210	*Quercus ilex*	8.872	39.009
Quercus ilex	-6.619	37.414	*Quercus ilex*	16.057	40.751
Quercus ilex	-1.227	41.336	*Quercus ilex*	23.163	38.975
Quercus ilex	-4.321	38.254	*Quercus ilex*	-3.318	41.290
Quercus ilex	-4.158	36.979	*Quercus ilex*	9.056	39.856
Quercus ilex	-5.132	43.352	*Quercus ilex*	-7.839	38.206
Quercus ilex	12.730	41.540	*Quercus ilex*	3.093	42.340
Quercus ilex	-5.792	39.152	*Quercus ilex*	-6.847	39.063
Quercus ilex	-4.259	42.794	*Quercus ilex*	-8.230	38.809
Quercus ilex	14.067	42.603	*Quercus ilex*	13.334	43.489
Quercus ilex	16.090	40.969	*Quercus ilex*	-3.686	41.671
Quercus ilex	15.343	44.281	*Quercus ilex*	-8.747	37.419
Quercus ilex	-5.419	38.262	*Quercus ilex*	-3.392	38.752
Quercus ilex	-3.245	41.130	*Quercus ilex*	-1.161	38.873
Quercus ilex	-7.102	37.440	*Quercus ilex*	16.378	43.552
Quercus ilex	-6.671	41.001	*Quercus ilex*	9.317	40.568
Quercus ilex	14.278	37.127	*Quercus ilex*	-0.033	38.640
Quercus ilex	-5.730	40.400	*Quercus ilex*	-4.293	36.763
Quercus ilex	-2.731	42.419	*Quercus ilex*	5.904	44.342
Quercus ilex	-2.433	37.655	*Quercus ilex*	23.784	40.592
Quercus ilex	-3.460	37.317	*Quercus ilex*	26.083	37.604
Quercus ilex	-5.952	37.981	*Quercus ilex*	10.650	43.599
Quercus ilex	-5.435	41.530	*Quercus ilex*	14.987	37.844
Quercus ilex	16.404	40.530	*Quercus ilex*	-1.793	40.583
Quercus ilex	-5.606	42.394	*Quercus ilex*	-3.552	40.925
Quercus ilex	-3.902	41.760	*Quercus ilex*	12.717	42.552
Quercus ilex	-6.712	37.899	*Quercus ilex*	-8.020	38.776
Quercus ilex	0.087	40.274	*Quercus ilex*	20.535	39.236
Quercus ilex	-3.230	37.500	*Quercus ilex*	15.020	37.469
Quercus ilex	16.522	39.861	*Quercus ilex*	-3.700	36.831
Quercus ilex	-4.074	37.179	*Quercus ilex*	-8.539	37.484
Quercus ilex	-6.460	37.843	*Quercus ilex*	-5.903	41.072
Quercus ilex	-0.348	39.716	*Quercus ilex*	9.380	39.522
Quercus ilex	-2.899	42.151	*Quercus ilex*	-4.751	40.482
Quercus ilex	-4.639	36.554	*Quercus ilex*	-2.661	37.853
Quercus ilex	16.416	40.816	*Quercus ilex*	10.857	43.547

Quercus ilex	12.577	45.670	*Quercus ilex*	-3.957	37.337
Quercus ilex	-7.842	37.153	*Quercus ilex*	16.052	40.240
Quercus ilex	-1.799	42.338	*Quercus ilex*	-2.157	38.548
Quercus ilex	14.706	41.690	*Quercus ilex*	25.865	40.856
Quercus ilex	-7.066	42.188	*Quercus ilex*	12.802	43.871
Quercus ilex	13.633	42.583	*Quercus ilex*	-6.325	40.604
Quercus ilex	18.767	42.426	*Quercus ilex*	22.946	39.360
Quercus ilex	-6.452	40.872	*Quercus ilex*	-7.508	39.430
Quercus ilex	-4.730	40.293	*Quercus ilex*	-4.673	38.442
Quercus ilex	22.095	37.122	*Quercus ilex*	-5.788	42.882
Quercus ilex	8.753	40.710	*Quercus ilex*	-5.966	41.866
Quercus ilex	13.766	45.235	*Quercus ilex*	-6.776	39.616
Quercus ilex	9.604	40.535	*Quercus ilex*	-4.104	38.067
Quercus ilex	-5.942	39.316	*Quercus ilex*	-2.915	37.759
Quercus ilex	16.594	38.807	*Quercus ilex*	14.944	36.712
Quercus ilex	24.920	37.420	*Quercus ilex*	11.903	42.154
Quercus ilex	-6.512	42.783	*Quercus ilex*	-6.415	43.292
Quercus ilex	10.952	42.749	*Quercus ilex*	-0.786	38.483
Quercus ilex	-0.025	47.718	*Quercus ilex*	-6.265	42.603
Quercus ilex	-6.059	40.638	*Quercus ilex*	-0.804	39.491
Quercus ilex	-6.580	40.225	*Quercus ilex*	-6.769	38.354
Quercus ilex	5.081	44.652	*Quercus ilex*	-8.231	37.653
Quercus ilex	-0.077	41.846	*Quercus ilex*	-7.753	39.060
Quercus ilex	12.619	41.975	*Quercus ilex*	-0.217	38.518
Quercus ilex	-6.006	43.256	*Quercus ilex*	-5.272	40.112
Quercus ilex	11.334	42.688	*Quercus ilex*	-5.225	36.403
Quercus ilex	12.385	44.071	*Quercus ilex*	0.237	41.636
Quercus ilex	13.129	42.784	*Quercus ilex*	13.062	37.506
Quercus ilex	-5.983	41.537	*Quercus ilex*	-6.419	41.865
Quercus ilex	-8.667	37.711	*Quercus ilex*	24.764	35.023
Quercus ilex	25.358	35.335	*Quercus ilex*	-0.161	47.356
Quercus ilex	-6.100	39.039	*Quercus ilex*	14.470	45.316
Quercus ilex	19.500	40.473	*Quercus ilex*	12.491	44.954
Quercus ilex	-5.466	42.115	*Quercus ilex*	-6.102	40.458
Quercus ilex	-6.962	40.891	*Quercus ilex*	16.522	39.248
Quercus ilex	-7.472	38.996	*Quercus ilex*	25.431	37.028
Quercus ilex	-2.002	40.287	*Quercus ilex*	-7.467	40.014
Quercus ilex	-8.000	39.530	*Quercus ilex*	13.940	41.774
Quercus ilex	-5.448	37.129	*Quercus ilex*	-1.677	38.024
Quercus ilex	15.681	40.836	*Quercus ilex*	16.265	38.812
Quercus ilex	-8.103	38.185	*Quercus ilex*	12.731	42.890
Quercus ilex	-1.544	40.323	*Quercus ilex*	-2.630	41.689
Quercus ilex	-6.066	39.974	*Quercus ilex*	15.574	41.908
Quercus ilex	16.216	39.043	*Quercus ilex*	13.037	42.925
Quercus ilex	-6.303	39.243	*Quercus ilex*	22.891	38.341
Quercus ilex	-2.513	41.493	*Quercus ilex*	6.677	43.962

Quercus ilex	4.904	43.456	*Quercus ilex*	-5.455	32.677
Quercus ilex	4.464	43.504	*Quercus ilex*	-6.309	32.097
Quercus ilex	7.075	44.188	*Quercus ilex*	-5.033	33.033
Quercus ilex	4.941	44.814	*Quercus ilex*	-4.460	42.441
Quercus ilex	-5.884	39.531	*Quercus ilex*	-4.917	40.433
Quercus ilex	8.833	35.167	*Quercus ilex*	-2.785	38.063
Quercus ilex	1.384	45.204	*Quercus ilex*	-7.166	42.850
Quercus ilex	-0.481	46.034	*Quercus ilex*	-9.280	29.756
Quercus ilex	1.168	44.107	*Quercus ilex*	8.680	35.205
Quercus ilex	-1.679	47.363	*Quercus ilex*	8.792	36.288
Quercus ilex	0.702	45.436	*Quercus ilex*	8.254	41.045
Quercus ilex	0.485	45.300	*Quercus ilex*	-6.474	31.576
Quercus ilex	24.204	35.406	*Quercus ilex*	-5.210	33.560
Quercus ilex	24.483	35.367	*Quercus ilex*	-3.835	34.767
Quercus ilex	24.250	40.250	*Quercus ilex*	-5.020	35.150
Quercus ilex	22.200	38.200	*Quercus ilex*	-4.185	34.090
Quercus ilex	14.250	37.750	*Quercus ilex*	-8.100	31.154
Quercus ilex	2.833	36.467	*Quercus ilex*	-3.584	35.017
Quercus ilex	9.000	34.000	*Quercus ilex*	-10.034	29.203
Quercus ilex	15.731	40.102	*Quercus ilex*	24.200	38.247
Quercus ilex	26.233	39.127	*Quercus ilex*	21.339	40.104
Quercus ilex	-5.464	36.428	*Quercus ilex*	23.621	41.181
Quercus ilex	9.265	39.849	*Quercus ilex*	22.342	40.852
Quercus ilex	-7.529	37.534	*Quercus ilex*	21.749	38.232
Quercus ilex	19.918	39.441	*Quercus ilex*	8.400	39.042
Quercus ilex	-5.679	39.305	*Quercus ilex*	15.227	38.809
Quercus ilex	-4.450	42.990	*Quercus ilex*	10.087	36.365
Quercus ilex	8.504	40.613	*Quercus ilex*	28.055	36.093
Quercus ilex	23.600	40.260	*Quercus ilex*	11.043	37.070
Quercus ilex	-8.244	37.925	*Quercus ilex*	6.780	35.363
Quercus ilex	24.680	41.090	*Quercus ilex*	10.316	42.330
Quercus ilex	-3.297	38.357	*Quercus ilex*	11.101	42.253
Quercus ilex	-4.953	36.844	*Quercus ilex*	10.075	42.581
Quercus ilex	-6.648	37.730	*Quercus ilex*	9.899	43.431
Quercus ilex	9.345	39.353	*Quercus ilex*	8.714	39.820
Quercus ilex	22.406	36.964	*Quercus ilex*	8.465	39.566
Quercus ilex	-5.645	35.702	*Quercus ilex*	9.590	39.710
Quercus ilex	-4.622	34.907	*Quercus ilex*	-4.086	34.307
Quercus ilex	-5.882	35.787	*Quercus ilex*	27.833	36.600
Quercus ilex	-3.442	34.566	*Quercus ilex*	26.267	37.633
Quercus ilex	-5.217	35.161	*Quercus ilex*	24.883	37.850
Quercus ilex	-5.695	41.160	*Quercus ilex*	24.700	40.767
Quercus ilex	-2.404	36.992	*Quercus ilex*	24.117	39.100
Quercus ilex	-6.483	32.150	*Quercus ilex*	25.317	36.717
Quercus ilex	-8.300	30.900	*Quercus ilex*	24.600	38.833
Quercus ilex	-3.233	36.783	*Quercus ilex*	24.750	37.967

Quercus ilex	20.733	39.967
Quercus ilex	24.617	38.133
Quercus ilex	26.017	35.133
Quercus ilex	25.217	37.583
Quercus ilex	7.796	44.002
Quercus ilex	-0.270	40.280
Quercus ilex	-0.410	39.960
Quercus ilex	-0.250	39.180
Quercus ilex	-1.070	39.870
Quercus ilex	-1.090	39.060
Quercus ilex	-1.410	39.510
Quercus ilex	-0.270	40.430
Quercus ilex	-0.630	40.620
Quercus ilex	-0.250	40.570
Quercus ilex	-0.940	39.230
Quercus ilex	-0.220	40.740
Quercus ilex	-1.360	40.020
Quercus ilex	-0.660	38.930
Quercus ilex	-0.400	38.520
Quercus ilex	1.465	36.457
Quercus ilex	3.896	36.378
Quercus ilex	10.419	36.576
Quercus ilex	3.243	43.931
Quercus ilex	0.390	40.790
Quercus ilex	0.740	40.890
Quercus ilex	2.630	42.260
Quercus ilex	1.920	41.270
Quercus ilex	2.150	42.260
Quercus ilex	1.310	41.620
Quercus ilex	1.410	42.430
Quercus ilex	0.490	41.160
Quercus ilex	0.140	41.060
Quercus ilex	0.600	41.340
Quercus ilex	1.300	41.980
Quercus ilex	-2.011	37.727
Quercus ilex	-4.300	34.883
Quercus ilex	24.371	38.081
Quercus ilex	0.409	40.929
Quercus ilex	-8.281	37.279
Quercus ilex	-1.311	39.719
Quercus ilex	1.249	38.899
Quercus ilex	-5.821	37.729
Quercus ilex	4.289	39.829
Quercus ilex	-0.911	41.309
Quercus ilex	-0.071	41.289
Quercus ilex	-3.181	42.229

Quercus ilex	-7.911	31.229
Quercus ilex	8.559	36.349
Quercus ilex	-4.781	43.109
Quercus ilex	-7.421	42.819
Quercus ilex	-4.101	39.429
Quercus ilex	-3.751	40.809
Quercus ilex	-0.161	42.009
Quercus ilex	-6.731	39.409
Quercus ilex	-6.321	38.139
Quercus ilex	-6.871	38.689
Quercus ilex	-1.057	47.555
Quercus ilex	-0.798	47.654
Quercus ilex	-2.093	47.244
Quercus ilex	-0.698	46.215
Quercus ilex	3.564	44.293
Quercus ilex	4.713	45.001
Quercus ilex	4.602	45.543
Quercus ilex	-3.080	33.990
Quercus ilex	-5.190	33.410
Quercus ilex	25.550	40.500
Quercus ilex	-1.740	41.680
Quercus ilex	-0.860	42.750
Quercus ilex	-8.098	39.801
Quercus ilex	-3.608	37.564
Quercus ilex	-3.043	42.617
Quercus ilex	24.088	35.218
Quercus ilex	6.255	35.490
Quercus ilex	1.621	35.879
Quercus ilex	-4.839	33.829
Quercus ilex	-4.854	33.664
Quercus ilex	3.084	36.731
Quercus ilex	2.717	35.911
Quercus ilex	0.147	35.387
Quercus ilex	22.561	39.875
Quercus ilex	-8.570	37.319
Quercus ilex	-8.589	38.183
Quercus ilex	23.459	37.500
Quercus ilex	9.270	35.436
Quercus ilex	-1.330	34.636
Quercus ilex	-0.621	34.676
Quercus ilex	0.917	34.934
Quercus ilex	5.733	34.850
Quercus ilex	-1.254	34.886
Quercus ilex	-8.457	31.050
Quercus ilex	-5.441	32.348
Quercus ilex	4.292	36.481

Quercus ilex	6.649	43.261	*Quercus ilex*	-2.221	41.409
Quercus ilex	-0.633	35.697	*Quercus ilex*	-2.221	41.589
Quercus ilex	-6.343	34.100	*Quercus ilex*	-0.091	41.019
Quercus ilex	-3.454	37.123	*Quercus ilex*	1.849	42.399
Quercus ilex	2.876	36.681	*Quercus ilex*	-2.031	38.169
Quercus ilex	-4.297	33.472	*Quercus ilex*	-7.111	42.659
Quercus ilex	7.755	36.902	*Quercus ilex*	-4.031	42.039
Quercus ilex	2.897	36.147	*Quercus ilex*	-5.031	43.199
Quercus ilex	-5.080	39.370	*Quercus ilex*	-4.771	42.839
Quercus ilex	-4.140	38.390	*Quercus ilex*	-6.391	42.379
Quercus ilex	-3.580	39.380	*Quercus ilex*	-3.311	43.039
Quercus ilex	-6.930	42.340	*Quercus ilex*	-5.121	42.919
Quercus ilex	3.110	39.290	*Quercus ilex*	-6.231	43.269
Quercus ilex	-0.180	40.070	*Quercus ilex*	-6.131	41.869
Quercus ilex	-6.950	37.740	*Quercus ilex*	35.188	42.021
Quercus ilex	-0.920	38.830	*Quercus ilex*	31.405	41.296
Quercus ilex	-5.580	43.230	*Quercus ilex*	31.345	41.143
Quercus ilex	-7.420	37.210	*Quercus ilex*	27.126	37.689
Quercus ilex	-4.120	36.770	*Quercus ilex*	27.311	37.705
Quercus ilex	-7.300	42.250	*Quercus ilex*	27.569	36.733
Quercus ilex	-4.850	39.460	*Quercus ilex*	28.798	41.167
Quercus ilex	-1.840	39.110	*Quercus ilex*	29.119	41.074
Quercus ilex	14.000	37.500	*Quercus ilex*	25.867	40.164
Quercus ilex	-2.821	41.689	*Quercus ilex*	26.599	38.204
Quercus ilex	-1.981	41.499	*Quercus ilex*	36.330	41.319

S2 Table. 19 bioclimatic variables obtained from WorldClim version 1.4.

Abbreviations	Variable name	Unit	Original resouliton	Source
Bio1	Annual Mean Temperature	°C	2.5 minutes	Worldclim
Bio2	Mean Diurnal Range	°C	2.5 minutes	Worldclim
Bio3	Isothermality	Bio_2/Bio_7	2.5 minutes	Worldclim
Bio4	Temperature Seasonality (standard deviation *100)	°C	2.5 minutes	Worldclim
Bio5	Max Temperature of Warmest Month	°C	2.5 minutes	Worldclim
Bio6	Min Temperature of Coldest Month	°C	2.5 minutes	Worldclim
Bio7	Temperature Annual Range	°C	2.5 minutes	Worldclim
Bio8	Mean Temperature of Wettest Quarter	°C	2.5 minutes	Worldclim
Bio9	Mean Temperature of Driest Quarter	°C	2.5 minutes	Worldclim
Bio10	Mean Temperature of Warmest Quarter	°C	2.5 minutes	Worldclim
Bio11	Mean Temperature of Coldest Quarter	°C	2.5 minutes	Worldclim
Bio12	Annual Precipitation	mm/year	2.5 minutes	Worldclim
Bio13	Precipitation of Wettest Month	mm/month	2.5 minutes	Worldclim
Bio14	Precipitation of Driest Month	mm/month	2.5 minutes	Worldclim
Bio15	Precipitation Seasonality	%	2.5 minutes	Worldclim
Bio16	Precipitation of Wettest Quarter	mm/quarter	2.5 minutes	Worldclim
Bio17	Precipitation of Driest Quarter	mm/quarter	2.5 minutes	Worldclim
Bio18	Precipitation of Warmest Quarter	mm/quarter	2.5 minutes	Worldclim
Bio19	Precipitation of Coldest Quarter	mm/quarter	2.5 minutes	Worldclim

S3 Table. Correlation matrix between 19 bioclimatic variables.

Variables	bio1	bio2	bio3	bio4	**bio5**	bio6	**bio7**	**bio8**	bio9	bio10	**bio11**	bio12	bio13	bio14	**bio15**	**bio16**	**bio17**	bio18	bio19
bio1	1																		
bio2	*0.81*	1																	
bio3	*0.79*	0.72	1																
bio4	-0.28	0.02	-0.66	1															
**bio5**	*0.91*	*0.9*	0.58	0.11	1														
bio6	*0.88*	0.52	*0.85*	-0.68	0.62	1													
**bio7**	0.16	0.53	-0.2	*0.85*	0.55	-0.32	1												
**bio8**	-0.06	-0.03	-0.33	0.48	0.08	-0.27	0.38	1											
bio9	*0.91*	*0.75*	*0.8*	-0.38	*0.81*	*0.86*	0.06	-0.35	1										
bio10	*0.93*	*0.84*	0.57	0.08	*0.99*	0.67	0.48	0.11	*0.81*	1									
**bio11**	*0.95*	0.67	*0.88*	-0.58	0.74	*0.98*	-0.15	-0.22	*0.91*	*0.77*	1								
bio12	-0.69	-0.75	-0.43	-0.21	-0.79	-0.38	-0.55	-0.29	-0.54	-0.79	-0.51	1							
bio13	-0.61	-0.68	-0.39	-0.17	-0.7	-0.34	-0.48	-0.29	-0.46	-0.69	-0.46	*0.94*	1						
bio14	-0.69	-0.72	-0.44	-0.16	-0.79	-0.42	-0.51	-0.12	-0.63	-0.77	-0.54	*0.86*	0.67	1					
**bio15**	0.71	0.67	0.58	-0.1	0.69	0.55	0.25	-0.12	0.67	0.7	0.63	-0.59	-0.38	-0.77	1				
**bio16**	-0.62	-0.69	-0.39	-0.19	-0.71	-0.33	-0.5	-0.31	-0.46	-0.7	-0.45	*0.95*	1	0.69	-0.39	1			
**bio17**	-0.7	-0.74	-0.44	-0.17	-0.8	-0.42	-0.52	-0.13	-0.62	-0.78	-0.53	*0.88*	0.7	*0.99*	-0.78	0.71	1		
bio18	-0.84	-0.77	-0.66	0.11	-0.85	-0.68	-0.3	0.15	-0.86	-0.84	-0.76	*0.78*	0.67	*0.87*	-0.72	0.68	*0.87*	1	
bio19	-0.31	-0.49	-0.1	-0.38	-0.46	0	-0.57	-0.53	-0.1	-0.45	-0.13	*0.83*	*0.87*	0.53	-0.23	*0.88*	0.56	0.33	1

S4 Table. Bioclimatic variables used as environmental input in the models.

Abbreviations	Variable name	Unit	Original resouliton	Source
Bio5	Max Temperature of Warmest Month	°C	2.5 minutes	Worldclim
Bio7	Temperature Annual Range	°C	2.5 minutes	Worldclim
Bio8	Mean Temperature of Wettest Quarter	°C	2.5 minutes	Worldclim
Bio11	Mean Temperature of Coldest Quarter	°C	2.5 minutes	Worldclim
Bio15	Precipitation Seasonality	%	2.5 minutes	Worldclim
Bio16	Precipitation of Wettest Quarter	mm/quarter	2.5 minutes	Worldclim
Bio17	Precipitation of Driest Quarter	mm/quarter	2.5 minutes	Worldclim

S5 Table. Fossil pollen records for the *Quercus ilex* from LIG, LGM and MH periods. Age oldest refers to age of the oldest *Quercus ilex* fossil pollen record in the given locality. LDD refers to both continous and discountinous low number of fossil pollen records which indicate either nearby refugia or long distance dispersal. Full references are given at the bottom of the table.

Taxon	Country	Locality/site	Latitude	Longitude	Age Oldest	LIG	LGM	MH	Reference
Quercus ilex	France	Lac du Bouchet	44.917	3.783	123400	LDD	Absent	LDD	(Reille & de Beaulieu, 1990; Thouveny et al., 1994)
Quercus ilex	Lebanon	Chamsine	33.753	35.948	12900	No data	No data	LDD	(Hajar et al., 2010)
Quercus ilex	Lebanon	Aammiq	33.732	35.794	14000	No data	No data	LDD	(Hajar et al., 2010)
Quercus ilex	Greece	Ioannina I	39.762	20.730	57971	No data	LDD	LDD	(Bottema, 1974)
Quercus ilex	Greece	Tenaghi Philippon	40.964	24.263	6232	No data	No data	Presence	(Turner & Greig, 1975)
Quercus ilex	Italy	Lago Trifoglietti	39.549	16.022	11439	No data	No data	Presence	(Joannin et al., 2012)
Quercus ilex	Italy	Lago Grande di Monticchio	40.931	15.605	132809	Presence	Absent	Presence	(Watts et al., 1996; Huntley et al., 1999; Allen et al., 2000; Allen et al., 2002; Allen & Huntley, 2009)
Quercus ilex	Italy	Colfiorito	43.019	12.875	20252	No data	LDD	No data	(Brugiapaglia & de Beaulieu, 1995)
Quercus ilex	Italy	Lago di Massaciuccoli	43.837	10.330	12150	No data	No data	Presence	(Mariotti Lippi et al., 2007)
Quercus ilex	Italy	Lac du Verney-Dessus	45.693	6.872	10933	No data	No data	LDD	(Miras et al., 2006)
Quercus ilex	Italy	Torveraz	45.695	6.860	9301	No data	No data	LDD	(Miras et al., 2006)
Quercus ilex	Morroco	Lake Hachlaf	33.550	-4.99	6227	No data	No data	Presence	(Nourelbait et al., 2016)
Quercus ilex	Tunisia	Djebel El Ghorra	36.597	8.394	6726	No data	No data	Presence	(Ben Tiba, 2014)
Quercus ilex	Spain	Quintanar de la Sierra	42.033	-3.016	23757	No data	LDD	Presence	(Penalba, 1994; Peñalba et al., 1997)
Quercus ilex	Spain	Laguna Negra	41.999	-2.847	17708	No data	No data	Presence	(von Engelbrechten, 1998)
Quercus ilex	Spain	Laguna Guallar	41.400	-0.217	9095	No data	No data	Presence	
Quercus ilex	Spain	Puerto de Los Tornos	43.150	-3.433	7235	No data	No data	Presence	(Penalba, 1989)
Quercus ilex	Spain	Saldropo	43.050	-2.717	9098	No data	No data	Presence	(Penalba, 1989; Penalba, 1994)
Quercus ilex	Spain	Puerto de Belate	43.033	-2.050	7650	No data	No data	Presence	(Penalba, 1989; Penalba, 1994)
Quercus ilex	Spain	Atxuri	43.250	-1.551	6437	No data	No data	LDD	(Penalba, 1989; Penalba, 1994)
Quercus ilex	Spain	Lake Banyoles	42.129	2.752	31540	No data	LDD	Presence	(Pèrez-Obiol & Julià, 1994)
Quercus ilex	Andorra	Bosc dels Estanyons	42.480	1.628	11899	No data	No data	LDD	(de Beaulieu et al., 2005)
Quercus ilex	Andorra	Planell de Perafita	42.479	1.566	10158	No data	No data	LDD	(Miras et al., 2010)
Quercus ilex	France	Watten	50.833	2.213	8169	No data	No data	LDD	(Gandouin et al., 2009)
Quercus ilex	France	La vie	48.548	-2.258	18702	No data	No data	LDD	(Barbier & Visset, 2000)
Quercus ilex	France	Le Fourneau	48.444	-0.191	25679	No data	Absent	LDD	(Barbier & Visset, 2000)
Quercus ilex	France	Locmariaquer	47.554	-2.932	11400	No data	No data	LDD	(Visset et al., 1996)
Quercus ilex	France	Castle Suscinio	47.512	-2.727	7518	No data	No data	LDD	(Visset & Bernard, 2006)
Quercus ilex	France	Ilot Sully	47.222	-1.551	7438	No data	No data	Presence	(Visset et al., 2001)
Quercus ilex	France	Le Gesvres	47.272	-1.595	8275	No data	No data	LDD	(Ouguerram & Visset, 2001)
Quercus ilex	France	Saint Viaud Cantin	47.265	-2.010	6434	No data	No data	LDD	(Bernard, 1996)
Quercus ilex	France	Petit Marais	47.217	-2.102	7611	No data	No data	LDD	(Marchand et al., 1999)
Quercus ilex	France	Petit Rocher	46.663	-1.915	8952	No data	No data	LDD	(Joly & Visset, 2009)
Quercus ilex	France	Jaunay	46.662	-1.892	9010	No data	No data	LDD	(Joly & Visset, 2009)
Quercus ilex	France	Vertonne	46.549	-1.764	7893	No data	No data	LDD	(Joly & Visset, 2009)
Quercus ilex	France	La Grange	46.089	-0.775	9744	No data	No data	LDD	(Joly & Visset, 2009)
Quercus ilex	France	Pierre Folle	47.018	-1.089	6351	No data	No data	LDD	(Bernard, 1996)
Quercus ilex	France	Chantemerle	45.916	0.016	8093	No data	No data	LDD	(Fauquette et al., 1999)
Quercus ilex	France France	Lac du Mont de Belier	45.339	2.645	12476	No data	No data	LDD	(de Beaulieu et al., 1985)
Quercus ilex	France	Bonnecombe	44.566	3.125	13555	No data	No data	LDD	(de Beaulieu et al., 1985)
Quercus ilex	France	Lac du Bouchet (BOUCHB53)	44.916	3.783	69779	No data	Absent	LDD	(Reille & de Beaulieu, 1988)
Quercus ilex	France	Lac du Bouchet	44.916	3.783	50000	No data	Absent	LDD	(Reille & de Beaulieu, 1988)
Quercus ilex	France	Hières sur Amby(AMBY80)	45.790	5.283	16333	No data	No data	LDD	(Clerc, 1988)
Quercus ilex	France	Le Grand Lemps(LEMPS85)	45.473	5.416	9286	No data	No data	LDD	(Clerc, 1988)
Quercus ilex	France	Le Grand Lemps(LEMPS83)	45.473	5.416	16268	No data	No data	LDD	(Clerc, 1988)
Quercus ilex	France	Grand Ratz le Pellet	45.425	5.608	15500	No data	No data	LDD	(Clerc, 1988)
Quercus ilex	France	Saint Julien de Ratz(RATZ81)	45.348	5.655	19727	No data	Absent	LDD	(Clerc, 1988)
Quercus ilex	France	Saint Julien de Ratz(RATZ81)	45.348	5.655	12348	No data	No data	LDD	(Clerc, 1988)
Quercus ilex	France	Lac des Boites	45.056	5.882	15614	No data	No data	LDD	(Nakagawa, 1998)
Q.ilex/Q.coccifera	Greece	Lake Kournas	35.329	24.275	9554	No data	No data	Presence	(Jouffroy-Bapicot et al., 2021)
Q.ilex/Q.coccifera	Greece	Lake Lerna	37.578	22.728	6788	No data	No data	Presence	(Jahns, 1993)
Q.ilex/Q.coccifera	France	Piantarella lagoon	41.373	9.219	6615	No data	No data	Presence	(Revelles et al., 2019)
Q.ilex/Q.coccifera	France	Saint Florent	42.672	9.304	6301	No data	No data	Presence	(Revelles et al., 2019)
Q.ilex/Q.coccifera	Spain	Les Palanques	42.162	2.438	10098	No data	No data	Presence	(Piqué et al., 2018; Revelles et al., 2018)
Q.ilex/Q.coccifera	Spain	Creixell	41.155	1.433	6575	No data	No data	Presence	(Burjachs & Expósito, 2015)
Q.ilex/Q.coccifera	Spain	Pego-Oliva	38.876	-0.088	7003	No data	No data	Presence	(Brisset et al., 2020)
Q.ilex/Q.coccifera	Spain	Antas	37.208	-1.823	10498	No data	No data	Presence	(Yll et al., 1997; Pantaléon-Cano et al., 2003)
Q.ilex/Q.coccifera	Morroco	Dayet M'Had	35.128	-5.438	5741	No data	No data	Presence	(Cheddadi et al., 2015, 2017)
Q.ilex/Q.coccifera	Morroco	Bab El Karn	35.022	-5.206	9163	No data	No data	Presence	(Cheddadi et al., 2016, 2017)
Q.ilex/Q. coccifera-type	Turkey	Tecer Gölü	39.431	37.083	5850	No data	No data	LDD	(Kuzucuoğlu et al., 2011)
Q.ilex/Q. coccifera-type	France	Bastani	42.065	9.134	11732	No data	No data	Presence	(Leroy & Roiron, 1996)
Q.ilex-type	Black Sea	MD72/5-22	42.201	36.492	17923	No data	No data	LDD	(Shumilovskikh et al., 2012)
Q.ilex-type	Black Sea	MD72/5-25	42.100	36.616	63928	No data	LDD	No data	(Nowaczyk et al., 2012; Shumilovskikh et al., 2014)
Q.ilex-type	Morocco	Ifri Oudadane	35.212	-3.258	10860	No data	No data	Presence	(Linstädter & Kehl, 2012; Morales et al., 2013; Zapata et al., 2013)
Q.ilex-type	Greece	Akotivika	37.036	22.076	6395	No data	No data	Presence	(Engel et al., 2009)
Q.ilex-type	Greece	Pertouli	39.524	21.477	6178	No data	No data	Presence	(Athanasiadis, 1975)

Q.ilex-type	Greece	Litochoro	40.138	22.546	5135	No data	No data	Presence	(Athanasiadis, 1975)
Q.ilex-type	Greece	Khimaditis Ib	40.627	21.599	17503	No data	No data	LDD	(Bottema, 1974)
Q.ilex-type	Montenegro	Zminje Jezero	43.155	19.07	12740	No data	No data	LDD	(Cagliero. et al., 2023)
Q.ilex-type	Croatia	Bokanjacko	44.183	15.233	9559	No data	No data	Presence	(Grüger, 1996)
Q.ilex-type	Switzerland	Lago di Origlio	46.051	8.934	19423	No data	No data	LDD	(Tinner et al., 1999)
Q.ilex-type	Italy	Biviere di Gela	37.018	14.344	7334	No data	No data	Presence	(Colombaroli et al., 2009; Noti et al., 2009)
Q.ilex-type	Italy	Gorgo Basso(GBASSO)	37.600	12.650	10186	No data	No data	Presence	(Tinner et al., 2009)
Q.ilex-type	Italy	Gorgo Basso(GBASSO)	37.600	12.650	10187	No data	No data	Presence	(Calò et al., 2012; Tinner et al., 2009)
Q.ilex-type	Italy	Lago Preola	37.616	12.633	10299	No data	No data	Presence	(Calò et al., 2012)
Q.ilex-type	Italy	Gorgo Tondo	37.901	13.412	5597	No data	No data	Presence	(Tinner et al., 2016)
Q.ilex-type	Italy	Urgo Pietra Giordano	37.852	14.127	6993	No data	No data	Presence	(Tinner et al., 2016)
Q.ilex-type	Italy	Urio Quattrocchi	37.901	14.395	10238	No data	No data	Presence	(Bisculm et al., 2012)
Q.ilex-type	Italy	Lago di Nemi	41.716	12.700	11386	No data	No data	Presence	(Mercuri et al., 2002; Mercuri et al., 2013)
Q.ilex-type	Italy	Lago Albano	41.750	12.666	14039	No data	No data	Presence	(Mercuri et al., 2002; Mercuri et al., 2013)
Q.ilex-type	Italy	Lago di Martignano	42.113	12.317	13335	No data	No data	Presence	(Kelly & Huntley, 1991)
Q.ilex-type	Italy	Lago dell'Accesa	42.987	10.895	11551	No data	No data	Presence	(Vannière et al., 2008; Colombaroli et al., 2009)
Q.ilex-type	Italy	Lago di Massaciuccoli(MASSACHR)	42.837	10.330	6070	No data	No data	Presence	(Colombaroli et al., 2007, 2009)
Q.ilex-type	Italy	Lago di Massaciuccoli(MASSACHR)	42.837	10.330	6941	No data	No data	Presence	(Colombaroli et al., 2007, 2009)
Q.ilex-type	Italy	Lago del Greppo	44.119	10.673	14873	No data	No data	LDD	(Vescovi et al., 2010)
Q.ilex-type	Italy	Lago Verdarolo	44.359	10.123	14427	No data	No data	LDD	(Samartin et al., 2017; Morales-Molino. et al., 2021)
Q.ilex-type	Italy	Bondone	46.017	11.041	18596	No data	Absent	LDD	(Grüger, 1968)
Q.ilex-type	Italy	Lago del Segrino	45.828	9.264	13157	No data	No data	LDD	(Gobet et al., 2000)
Q.ilex-type	Italy	Ortasee	45.808	8.396	15965	No data	No data	LDD	(Rösch. et al., 2012)
Q.ilex-type	Italy	Lago Piccolo di Avigliana	45.053	7.391	20423	No data	Absent	LDD	(Finsinger & Tinner, 2006; Finsinger et al., 2008; Finsinger et al., 2011)
Q.ilex-type	Italy	Laghi dell'Orgials	44.233	7.133	25445	No data	Absent	LDD	(Ortu et al., 2008)
Q.ilex-type	Italy	Torbiera del Biecai	44.185	7.717	19013	No data	Absent	LDD	(Ortu et al., 2008)
Q.ilex-type	Portugal	Beliche-Guadiana	37.266	-7.450	13091	No data	No data	Presence	(Fletcher et al., 2007)
Q.ilex-type	Portugal	Lagoacho das Favas	40.357	-7.635	8153	No data	No data	Presence	(van der Knaap & van Leeuwen, 1995)
Q.ilex-type	Portugal	Lagoa Comprida 1	40.358	-7.638	13295	No data	No data	LDD	(Janssen & Woldringh, 1981; van der Knaap & van Leeuwen, 1997)
Q.ilex-type	Portugal	Lagoa Comprida 2	40.326	-7.636	12444	No data	No data	Presence	(Janssen & Woldringh, 1981; Van Den Brink & Janssen, 1985)
Q.ilex-type	Spain	Bajondillo	36.619	-4.496	142228	Presenve	LDD	Presence	(Cortés-Sánchez et al., 2008)
Q.ilex-type	Spain	Padul	37.000	-3.400	23600	No data	Presence	Presence	(Pons & Reille, 1988)
Q.ilex-type	Spain	Siles	38.400	-2.500	20300	No data	Presence	Presence	(Carrión, 2002)
Q.ilex-type	Spain	San Rafael	36.773	-2.601	20927	No data	Presence	Presence	(Yll et al., 1997; Pantaléon-Cano et al., 2003)
Q.ilex-type	Spain	Roquetas de Mar	36.794	-2.588	6999	No data	No data	Presence	(Yll et al., 1997; Pantaléon-Cano et al., 2003)
Q.ilex-type	Spain	Cucú cueva	37.638	-2.258	130000	Presence	No data	No data	(González-Ramón et al., 2012)
Q.ilex-type	Spain	Navarrés(NAVA1)	39.100	-0.683	19516	No data	LDD	Presence	(Carrion & Dupre, 1996)
Q.ilex-type	Spain	Navarrés(NAVA2)	39.100	0.683	19583	No data	LDD	Presence	(Carrion & Dupre, 1996)
Q.ilex-type	Spain	Navarrés(NAVA3)	39.100	0.683	36577	No data	Absent	Presence	(Carrión & Van Geel, 1999)
Q.ilex-type	Spain	Albufera Alcudia	39.792	3.119	8107	No data	No data	Presence	(Burjachs, 1994)
Q.ilex-type	Spain	Algendar	39.94	3.958	10066	No data	No data	Presence	(Yll et al., 1997)
Q.ilex-type	Spain	Cala Galdana	39.936	3.965	8386	No data	No data	LDD	(Yll et al., 1994)
Q.ilex-type	Spain	Son Bou	39.924	4.027	24418	No data	Presence	LDD	(Yll et al., 1997)
Q.ilex-type	Spain	Hort Timoner	39.875	4.126	9477	No data	No data	Presence	(Yll et al., 1997)
Q.ilex-type	Spain	Cala'n Porter	39.870	4.131	8998	No data	No data	Presence	(Yll et al., 1997)
Q.ilex-type	Spain	MD95-2039	40.578	-10.348	44875	No data	LDD	No data	(Schönfeld et al., 2003; Roucoux et al., 2005)
Q.ilex-type	Spain	El Maíllo mire	40.546	-6.209	10553	No data	No data	Presence	(Morales-Molino et al., 2013)
Q.ilex-type	Spain	El Carrizal lake	41.318	-4.144	9784	No data	No data	LDD	(Franco-Múgica et al., 2005)
Q.ilex-type	Spain	Canaleja	40.900	-2.450	10241	No data	No data	Presence	(Cerrillo-Cuenca & González-Cordero, 2011)
Q.ilex-type	Spain	Sanabria Marsh	42.100	-6.733	18089	No data	No data	Presence	(Allen et al., 1996)
Q.ilex-type	Spain	Ayoó de Vidriales	42.126	-6.070	18468	No data	No data	LDD	(Morales-Molino & García-Antón, 2014)
Q.ilex-type	Spain	Espinosa de Cerrato	41.952	-3.939	11500	No data	No data	Presence	(Morales-Molino et al., 2017)
Q.ilex-type	Spain	Tubilla del Lago	41.808	-3.572	7458	No data	No data	LDD	(Morales-Molino et al., 2017)
Q.ilex-type	Spain	Las Pardillas Lake	42.043	-3.045	10604	No data	No data	Presence	(Goñi & Hannon, 1999)
Q.ilex-type	Spain	Puerto de Belate	43.033	-2.050	8097	No data	No data	Presence	(Penalba, 1989)
Q.ilex-type	France	Lake Racou	42.554	2.008	12216	No data	No data	LDD	(Guiter et al., 2005)
Q.ilex-type	France	MD99-2348 (103)	42.692	3.841	24975	No data	LDD	No data	(Beaudouin et al., 2005; Célia et al., 2007)
Q.ilex-type	France	Tourbière de Gatimort	43.575	2.785	7892	No data	No data	LDD	(Pulido, 2006)
Q.ilex-type	France	Marais de Fos	43.436	4.936	6134	No data	No data	Presence	(Vella & Provansal, 2000)
Q.ilex-type	France	Le Marais de la Perge (South)	45.382	-1.115	6314	No data	No data	LDD	(Tastet & Diot, 1995)
Q.ilex-type	France	Marais de la Perge	45.379	-1.01	7888	No data	No data	LDD	(Tastet & Diot, 1995)
Q.ilex-type	France	Tourbière des Nassettes	44.466	3.641	8850	No data	No data	LDD	(Pulido, 2006)
Q.ilex-type	France	Tourbière des Narses Mortes	44.433	3.600	8850	No data	No data	LDD	(Jouffroy-Bapicot et al., 2007; Pulido, 2006)
Q.ilex-type	France	Le Grand Etang de Suze-La-Rousse	44.323	4.833	18514	No data	Absent	LDD	(Argant, 1990)
Q.ilex-type	France	Lac de Praver	45.073	5.856	7210	No data	No data	LDD	(Nakagawa, 1998)

Q.ilex-type	France	Lac des Boites	45.056	5.885	11345	No data	No data	LDD	(Nakagawa, 1998)
Q.ilex-type	France	La Gourre	44.385	6.003	15717	No data	No data	Presence	(Muller et al., 2012)
Q.ilex-type	France	Correo	44.555	5.995	29475	No data	LDD	LDD	(Nakagawa, 1998)
Q.ilex-type	France	Pelléautier	44.522	6.183	7768	No data	No data	LDD	(de Beaulieu, 1977)
Q.ilex-type	France	Biot	43.800	7.100	13882	No data	No data	Presence	(Nicol-Pichard & Dubar, 1998)
Q.ilex-type	France	Lac Long Inférieur	44.057	7.450	20811	No data	Absent	LDD	(de Beaulieu, 1977)
Q.ilex-type	France	Lac des Grenouilles	44.098	7.483	14662	No data	No data	Presence	(Finsinger et al., 2021)
Q.ilex-type	France	Sabion	44.13	7.473	13300	No data	No data	LDD	(de Beaulieu, 1977)
Q.ilex-type	France	Etang d'Ouveillan	43.266	3.000	8934	No data	No data	LDD	(Goñi & Fernanda, 1995)
Q.ilex-type	Egypt	Lake Qarun	29.47	30.637	9828	No data	No data	LDD	(Hamdan et al., 2020)
Q.ilex-type	Germany	Steerenmoos	47.805	8.2	10352	No data	No data	Presence	(Rösch et al., 2012)

References

Allen, J.R.M, Watts, W. ., McGee, E., & Huntley, B. (2002). Holocene environmental variability—the record from Lago Grande di Monticchio, Italy. *Quaternary International*, *88*(1), 69–80. https://doi.org/10.1016/S1040-6182(01)00074-X

Allen, J. R.M., Huntley, B., & Watts, W. A. (1996). The vegetation and climate of northwest Iberia over the last 14000 yr. *Journal of Quaternary Science*, *11*(2), 125–147. https://doi.org/10.1002/(SICI)1099-1417(199603/04)11:2<125::AID-JQS232>3.0.CO;2-U

Allen, J. R. M, & Huntley, B. (2009). Last Interglacial palaeovegetation, palaeoenvironments and chronology: a new record from Lago Grande di Monticchio, southern Italy. *Quaternary Science Reviews*, *28*(15), 1521–1538. https://doi.org/10.1016/j.quascirev.2009.02.013

Allen, J. R. M, Watts, W. A., & Huntley, B. (2000). Weichselian palynostratigraphy, palaeovegetation and palaeoenvironment; the record from Lago Grande di Monticchio, southern Italy. *Quaternary International*, *73–74*, 91–110. https://doi.org/10.1016/S1040-6182(00)00067-7

Argant, J. (1990). Climat et environnement au Quaternaire dans le Bassin du Rhône d'après les données palynologiques. In *Documents des Laboratoires de Géologie, Lyon* (pp. 3–199).

Athanasiadis, N. (1975). Zur postglazialen Vegetationsentwicklung von Litochoro Katerinis und Pertouli Trikalon (Griechenland). *Flora*, *164*(1), 99–132. https://doi.org/10.1016/S0367-2530(17)31791-7

Barbier, D., & Visset, L. (2000). Les spécificités d'un Tardiglaciaire armoricain: étude pollinique synthétique à partir de trois tourbières du Nord-Est mayennais (France). *Quaternaire*, *11*, 99–106. https://doi.org/10.3406/quate.2000.1659

Beaudouin, C., Suc, J.-P., Acherki, N., Courtois, L., Rabineau, M., Aloïsi, J.-C., Sierro, F. J., & Oberlin, C. (2005). Palynology of the northwestern Mediterranean shelf (Gulf of Lions): First vegetational record for the last climatic cycle. *Marine and Petroleum Geology*, *22*(6), 845–863. https://doi.org/https://doi.org/10.1016/j.marpetgeo.2005.03.005

Beaulieu, J.-L. D., Miras, Y., Andrieu-Ponel, V., & Guiter, F. (2005). Vegetation dynamics in north-western Mediterranean regions: Instability of the Mediterranean bioclimate. Plant Biosystems. *An International Journal Dealing with All Aspects of Plant Biology*, *132*(2), 114–126. https://doi.org/10.1080/11263500500197858

Ben Tiba, B. (2014). Pollen profile GHORRA01, Djebel El Ghorra, Tunisia. In *European Pollen Database (EPD)*. PANGAEA. https://doi.org/10.1594/PANGAEA.835810

Bernard, J. (1996). *Paléoenvironnement du Pays de Retz et du marais breton-vendéen.* Université de Nantes. Doctoral dissertation. Université de Nantes, Nantes, France

Bisculm, M., Colombaroli, D., Vescovi, E., van Leeuwen, J. F. N., Henne, P. D., Rothen, J., Procacci, G., Pasta, S., La Mantia, T., & Tinner, W. (2012). Holocene vegetation and fire dynamics in the supra-mediterranean belt of the Nebrodi Mountains (Sicily, Italy). *Journal of Quaternary Science*, *27*(7), 687–698. https://doi.org/https://doi.org/10.1002/jqs.2551

Bottema, S. (1974). Late quaternary vegetation history of northwestern Greece. *s.n*, 190.

Brisset, E., Revelles, J., Expósito, I., Bernabeu Aubán, J., & Burjachs, F. (2020). Socio-Ecological Contingencies with Climate Changes over the Prehistory in the Mediterranean Iberia. In *Quaternary* (Vol. 3, Issue 3). https://doi.org/10.3390/quat3030019

Brugiapaglia, E., & J.L. de Beaulieu. (1995). Etude de la dynamique végétale tardiglaciaire et Holocène en Italie centrale: le marais de Colfiorito (Ombrie). *Comptes Rendus de l'Académie*

Des Sciences, Serie II(321), 617–622.

Burjachs, F. (1994). Dinámica de la vegetación durante el Holoceno en la isla de Mallorca. In M. E. B. I. Mateu, M. Dupré, J. Güemes (Ed.), *Trabajos de Palinología básica y aplicada* (pp. 199–210).

Burjachs, F., & Expósito, I. (2015). Charcoal and pollen analysis: Examples of Holocene fire dynamics in Mediterranean Iberian Peninsula. *CATENA*, *135*, 340–349. https://doi.org/10.1016/j.catena.2014.10.006

Cagliero, E., L. Paradis, Marchi, N., Lisztes-Szabó, Z., Braun, M., Hubay, K., Sabatier, P., Curovic, M., Spalević, V., Motta, R., Lingua, E., & Finsinger., W. (2023). The role of fire disturbances, human activities and climate change for long-term forest dynamics in upper-montane forests of the central Dinaric Alps. *The Holocene:In Press*.

Calò, C., Henne, P. D., Curry, B., Magny, M., Vescovi, E., La Mantia, T., Pasta, S., Vannière, B., & Tinner, W. (2012). Spatio-temporal patterns of Holocene environmental change in southern Sicily. *Palaeogeography, Palaeoclimatology, Palaeoecology*, *323–325*, 110–122. https://doi.org/https://doi.org/10.1016/j.palaeo.2012.01.038

Carrion, J. S., & Dupre, M. (1996). Late Quaternary Vegetational History at Navarres, Eastern Spain. A Two Core Approach. The New Phytologist, 134(1), 177–191. 10.1111/J.1469-8137.1996.TB01157.X

Carrión, J S, & Van Geel, B. (1999). Fine-resolution Upper Weichselian and Holocene palynological record from Navarrés (Valencia, Spain) and a discussion about factors of Mediterranean forest succession. *Review of Palaeobotany and Palynology*, *106*(3), 209–236. https://doi.org/10.1016/S0034-6667(99)00009-3

Carrión, J. S. (2002). Patterns and processes of Late Quaternary environmental change in a montane region of southwestern Europe. *Quaternary Science Reviews*, *21*(18), 2047–2066. https://doi.org/10.1016/S0277-3791(02)00010-0

Célia, B., Gwenael, J., Suc, J.-P., Berne, S., & Escarguel, G. (2007). Vegetation dynamics in Southern France during the last 30 ky BP in the light of marine palynology. *Quaternary Science Reviews*, *26*, 1037–1054. https://doi.org/10.1016/j.quascirev.2006.12.009

Cerrillo-Cuenca, E., & González-Cordero, A. (2011). Burial prehistoric caves in the interior basin of river Tagus: the complex at Canaleja gorge (Romangordo, Caceres, Spain). *BAR International Series*, *2219*, 21–42.

Cheddadi, R., Bouaissa, O., Rhoujjati, A., & Dezileau, L. (2016). Environmental changes in the Moroccan western Rif mountains over the last 9,000 years. *Quaternaire*, *27*, 15–25. https://doi.org/10.4000/quaternaire.7517

Cheddadi, R., Henrot, A.-J., François, L., Boyer, F., Bush, M., Carré, M., Coissac, E., De Oliveira, P. E., Ficetola, F., Hambuckers, A., Huang, K., Lézine, A.-M., Nourelbait, M., Rhoujjati, A., Taberlet, P., Sarmiento, F., Abel-Schaad, D., Alba-Sánchez, F., & Zheng, Z. (2017). Microrefugia, Climate Change, and Conservation of Cedrus atlantica in the Rif Mountains, Morocco . In *Frontiers in Ecology and Evolution* (Vol. 5). 10.3389/fevo.2017.00114

Cheddadi, R., Nourelbait, M., Bouaissa, O., Tabel, J., Rhoujjati, A., López-Sáez, J. A., Alba-Sánchez, F., Khater, C., Ballouche, A., Dezileau, L., & Lamb, H. (2015). A History of Human Impact on Moroccan Mountain Landscapes. *African Archaeological Review*, *32*, 233–248. https://doi.org/10.1007/s10437-015-9186-7

Clerc, J. (1988). *Recherches pollenanalytiques sur la paléo-écologie Tardiglaciaire et Holocène du Bas-Dauphiné.* PhD Thesis, Université St. Jérôme, Marseille, France

Colombaroli, D., Marchetto, A., & Tinner, W. (2007). Long-term interactions between Mediterranean climate, vegetation and fire regime at Lago di Massaciuccoli (Tuscany, Italy). *Journal of Ecology, 95*(4), 755–770. https://doi.org/10.1111/j.1365-2745.2007.01240.x

Colombaroli, D., Tinner, W., Leeuwen, J., Noti, R., Vescovi, E., Vannière, B., Magny, M., Schmidt, R., & Bugmann, H. (2009). Response of broadleaved evergreen Mediterranean forest vegetation to fire disturbance during the Holocene: Insights from the peri-Adriatic region. *Journal of Biogeography, 36*, 314–326. https://doi.org/10.1111/j.1365-2699.2008.01987.x

Cortés-Sánchez, M., Morales-Muñiz, A., Simón-Vallejo, M. D., Bergadà-Zapata, M. M., Delgado-Huertas, A., López-García, P., López-Sáez, J. A., Lozano-Francisco, M. C., Riquelme-Cantal, J. A., Roselló-Izquierdo, E., Sánchez-Marco, A., & Vera-Peláez, J. L. (2008). Palaeoenvironmental and cultural dynamics of the coast of Málaga (Andalusia, Spain) during the Upper Pleistocene and early Holocene. *Quaternary Science Reviews*, *27*(23), 2176–2193. https://doi.org/10.1016/j.quascirev.2008.03.010

de Beaulieu, J. L. (1977). *Contribution pollenanalytique à l'histoire tardiglaciaire et Holocène de la végétation des Alpes méridionales françaices*. Doctoral dissertation. Université d'Aix-Marseille, Marseille, France.

de Beaulieu, J. L., Pons, A., & Reille, M. (1985). Recherches pollenanalytiques sur l'histoire tardiglaciaire et holocene de la vegetation des Monts d'Aubrac (Massif Central, France). *Review of Palaeobotany and Palynology, 44*(1), 37–80. https://doi.org/https://doi.org/10.1016/0034-6667(85)90028-4

Engel, M., Knipping, M., Brückner, H., Kiderlen, M., & Kraft, J. (2009). Reconstructing middle to late Holocene palaeogeographies of the lower Messenian plain (southwestern Peloponnese, Greece): Coastline migration, vegetation history and sea level change. *Palaeogeography Palaeoclimatology Palaeoecology*, 257–270. https://doi.org/10.1016/j.palaeo.2009.10.005

Fauquette, S., Guiot, J., Menut, M., de Beaulieu, J.-L., Reille, M., & Guenet, P. (1999). Vegetation and climate since the last interglacial in the Vienne area (France). *Global and Planetary Change, 20*(1), 1–17. https://doi.org/10.1016/S0921-8181(98)00054-X

Finsinger, W, Lane, C. S., van Den Brand, G. J., Wagner-Cremer, F., Blockley, S. P. E., & Lotter, A. F. (2011). The lateglacial Quercus expansion in the southern European Alps: rapid vegetation response to a late Allerød climate warming? *Journal of Quaternary Science*, *26*(7), 694–702. https://doi.org/https://doi.org/10.1002/jqs.1493

Finsinger, Walter, Belis, C., Blockley, S., Eicher, U., Leuenberger, M., Lotter, A., & Ammann, B. (2008). Temporal patterns in lacustrine stable isotopes as evidence for climate change during the late glacial in the Southern European Alps. *Journal of Paleolimnology*, *40*, 885–895. https://doi.org/10.1007/s10933-008-9205-7

Finsinger, W., & Tinner, W. (2006). Holocene vegetation and land-use changes in response to climatic changes in the forelands of the southwestern Alps, Italy. *Journal of Quaternary Science*, *21*(3), 243–258. https://doi.org/10.1002/jqs.971

Finsinger, W., Vanel, Q., Ribolini, A., & Tinner, W. (2021). Early to late Holocene vegetation and fire dynamics at the treeline in the Maritime Alps. *Vegetation History and Archaeobotany*, *30*. https://doi.org/10.1007/s00334-020-00795-x

Fletcher, W., Boski, T., & Moura, D. (2007). Palynological evidence for environmental and climatic change in the lower Guadiana valley, Portugal, during the last 13 000 years. *Holocene*, *17*, 481–494. https://doi.org/10.1177/0959683607077027

Franco-Múgica, F., García-Antón, M., Maldonado-Ruiz, J., Morla-Juaristi, C., & Sainz-Ollero, H.

(2005). Ancient pine forest on inland dunes in the Spanish northern meseta. *Quaternary Research*, *63*(1), 1–14. https://doi.org/10.1016/j.yqres.2004.08.004

Gandouin, E., Ponel, P., Andrieu-Ponel, V., Guiter, F., de Beaulieu, jacques-L., Djamali, M., Franquet, E., Vliet-Lanoë, B., Alvitre, M., Meurisse, M., Brocandel, M., & Brulhet, J. (2009). 10,000 years of vegetation history of the Aa palaeoestuary, St-Omer Basin, northern France. *Review of Palaeobotany and Palynology*, *156*, 307–318. https://doi.org/10.1016/j.revpalbo.2009.03.008

Gobet, E., Tinner, W., Hubschmid, P., Jansen, I., Wehrli, M., Ammann, B., & Wick, L. (2000). Influence of human impact and bedrock differences on the vegetational history of the Insubrian Southern Alps. *Vegetation History and Archaeobotany*, *9*(3), 175–187. https://doi.org/10.1007/BF01299802

Goñi, M. F. S., & Hannon, G. E. (1999). High-altitude vegetational pattern on the Iberian Mountain Chain (north-central Spain) during the Holocene. *The Holocene*, *9*(1), 39–57. https://doi.org/10.1191/095968399671230625

Goñi, S., & Fernanda, M. (1995). Analyse palynologique de l'Etang d'Ouveillan. In *Temps et espace da ns le bassin de lAude du Neolithique a lAge du Fer* (pp. 265–275). CNRS.

González-Ramón, A., Andreo, B., Ruiz-Bustos, A., Richards, D. A., López-Sáez, J. A., & Alba-Sánchez, F. (2012). Late Quaternary paleoenvironmental record from a sedimentary fill in Cucú cave, Almería, SE Spain. *Quaternary Research*, *77*(2), 264–272. https://doi.org/https://doi.org/10.1016/j.yqres.2011.12.001

Grüger, E. (1996). Vegetation change. In and S. B. J. Chapman, R. Shiel (Ed.), *The changing face of Dalmatia. Archaeological and ecological investigations in a Mediterranean Landscape* (pp. 33–44). Leicester University Press,.

Grüger, J. (1968). Untersuchungen zur spätglazialen und frühpostglazialen Vegetationsentwicklung der Südalpen im Umkreis des Gardassees. *Botanische Jahrbücher Für Systematik Pflanzengeschichte Und Pflanzengeographie*, *88*, 163–199.

Guiter, F., Andrieu-Ponel, V., Digerfeldt, G., Reille, M., de Beaulieu, jacques-L., & Ponel, P. (2005). Vegetation history and lake-level changes from the Younger Dryas to the present in Eastern Pyrenees (France): Pollen, plant macrofossils and lithostratigraphy from Lake Racou (2000 m a.s.l.). *Vegetation History and Archaeobotany*, *14*, 99–118. https://doi.org/10.1007/s00334-005-0065-z

Hamdan, M. A., R. J. Flower, F. A. Hassan, and S. A. G. Leroy. 2020. "Geochemical and Palynological Analysis of Faiyum Lake Sediments, Egypt: Implications for Holocene Paleoclimate." Journal of African Earth Sciences 167:103864. doi: https://doi.org/10.1016/j.jafrearsci.2020.103864.

Hajar, L., Haïdar-Boustani, M., Khater, C., & Cheddadi, R. (2010). Environmental changes in Lebanon during the Holocene: Man vs. climate impacts. *Journal of Arid Environments*, *74*(7), 746–755. https://doi.org/10.1016/j.jaridenv.2008.11.002

Huntley, B., Watts, W. A., Allen, J. R. M., & Zolitschka, B. (1999). Palaeoclimate, chronology and vegetation history of the Weichselian Lateglacial: comparative analysis of data from three cores at Lago Grande di Monticchio, southern Italy. *Quaternary Science Reviews*, *18*(7), 945–960. https://doi.org/10.1016/S0277-3791(99)00007-4

Jahns, S. (1993). On the Holocene vegetation history of the Argive Plain (Peloponnese, southern Greece). *Vegetation History and Archaeobotany*, *2*(4), 187–203.

https://doi.org/10.1007/BF00198161

Janssen, C., & Woldringh, R. (1981). A preliminary radiocarbon dated pollen sequence from the Serra da Estrela, Portugal. *Finisterra*, *16*(32 SE-Artigos). https://doi.org/10.18055/Finis2176

Joannin, S., Elisabetta, B., de Beaulieu, Jacques-L., Bernardo, L., Magny, M., Peyron, O., & Vannière, B. (2012). Pollen-based reconstruction of Holocene vegetation and climate in Southern Italy: the case of Lago di Trifoglietti. *Climate of the Past Discussions, 8,* 2223–2279. https://doi.org/10.5194/cpd-8-2223-2012

Joly, C., & Visset, L. (2009). Evolution of vegetation landscapes since the Late Mesolithic on the French West Atlantic coast. *Review of Palaeobotany and Palynology*, *154*, 124–179. https://doi.org/10.1016/j.revpalbo.2008.12.011

Jouffroy-Bapicot, I., Pedrotta, T., Debret, M., Field, S., Sulpizio, R., Zanchetta, G., Sabatier, P., Roberts, N., Tinner, W., Walsh, K., & Vannière, B. (2021). Olive groves around the lake. A ten-thousand-year history of a Cretan landscape (Greece) reveals the dominant role of humans in making this Mediterranean ecosystem. *Quaternary Science Reviews*, *267*, 107072. https://doi.org/10.1016/j.quascirev.2021.107072

Jouffroy-Bapicot, I., Pulido, M., Baron, S., Galop, D., Monna, F., Lavoie, M., Ploquin, A., Petit, C., de Beaulieu, jacques-L., & Richard, H. (2007). Environmental impact of early palaeometallurgy: Pollen and geochemical analysis. *Vegetation History and Archaeobotany*, *16*, 251–258. https://doi.org/10.1007/s00334-006-0039-9

Kelly, M. G., & Huntley, B. (1991). An 11 000-year record of vegetation and environment from Lago di Martignano, Latium, Italy. *Journal of Quaternary Science*, *6*(3), 209–224. https://doi.org/https://doi.org/10.1002/jqs.3390060304

Kuzucuoğlu, C., Dörfler, W., Kunesch, S., & Goupille, F. (2011). Mid- to late-Holocene climate change in central Turkey: The Tecer Lake record. *The Holocene*, *21*(1), 173–188. https://doi.org/10.1177/0959683610384163

Leroy, S. A. G., & Roiron, P. (1996). Latest Pliocene pollen and leaf floras from Bernasso palaeolake (Escandorgue Massif, Hérault, France). *Review of Palaeobotany and Palynology*, *94*(3), 295–328. https://doi.org/https://doi.org/10.1016/S0034-6667(96)00016-4

Linstädter, J., & Kehl, M. (2012). The Holocene archaeological sequence and sedimentological processes at Ifri Oudadane, NE Morocco. *Journal of Archaeological Science*, *39*(10), 3306–3323. https://doi.org/https://doi.org/10.1016/j.jas.2012.05.025

Marchand, G., Tessier, M., & Bernard, J. (1999). Les occupations mésolithiques et néolithiques de la Fillauderie (Saint-Père-en-Retz, Loire-Atlantique) et la préhistoire récente de la basse vallée du Boivre. *Revue Archéologique De l'Ouest*, *16*(1), 39–65. https://doi.org/10.3406/RAO.1999.1087

Mariotti Lippi, M., Lippi, M. M., Guido, M., Menozzi, B. I., Bellini, C., & Montanari, C. (2007). The Massaciuccoli Holocene pollen sequence and the vegetation history of the coastal plains by the Mar Ligure (Tuscany and Liguria, Italy). *Vegetation History and Archaeobotany*, *16*(4), 267–277. https://doi.org/10.1007/S00334-006-0090-6

Mercuri, A M, Bandini Mazzanti, M., Florenzano, A., Montecchi, M. C., & Rattighieri, E. (2013). Olea, Juglans and Castanea: The OJC group as pollen evidence of the development of human-induced environments in the Italian peninsula. *Quaternary International*, *303*, 24–42. https://doi.org/10.1016/j.quaint.2013.01.005

Mercuri, A., Accorsi, C. & Bandini Mazzanti, M (2002). The long history of Cannabis and its cultivation by the Romans in central Italy, shown by pollen records from Lago Albano and Lago di Nemi. *Vegetation History and Archaeobotany*, *11*(4), 263–276.

https://doi.org/10.1007/s003340200039

Miras, Y., Millet, L., Guiter, F., Ponel, P., Beaulieu, J. L. de, & Goslar, T. (2006). Dynamique des écosystèmes et impact de l'homme dans le secteur du col du Petit Saint Bernard au cours de l'Holocène. Actes du Programme Interreg "Alpis Graïa." *Aoste*, 31–50.

Miras, Y, Ejarque, A., Orengo, H., Riera, S., Martínez, J. M., & Alexandre, P. (2010). Prehistoric impact on landscape and vegetation at high altitudes: An integrated palaeoecological and archaeological approach in the eastern Pyrenees (Perafita valley, Andorra). *Plant Biosystems, 144*. https://doi.org/10.1080/11263504.2010.491980

Morales-Molino, C., Steffen, M., Samartin, S., van Leeuwen, J. F. N., Hürlimann, D., Vescovi, E., & Tinner, W. (2021). Long-Term Responses of Mediterranean Mountain Forests to Climate Change, Fire and Human Activities in the Northern Apennines (Italy). *Ecosystems (New York, N.Y.)*, *24*(6), 1361–1377. https://doi.org/10.1007/s10021-020-00587-4

Morales-Molino, C., & García-Antón, M. (2014). Vegetation and fire history since the last glacial maximum in an inland area of the western Mediterranean Basin (Northern Iberian Plateau, NW Spain). *Quaternary Research*, *81*(1), 63–77. https://doi.org/10.1016/j.yqres.2013.10.010

Morales-Molino, C., García-Antón, M., Postigo-Mijarra, J. M., & Morla, C. (2013). Holocene vegetation, fire and climate interactions on the westernmost fringe of the Mediterranean Basin. *Quaternary Science Reviews*, *59*, 5–17. https://doi.org/10.1016/j.quascirev.2012.10.027

Morales-Molino, C., Tinner, W., García-Antón, M., & Colombaroli, D. (2017). The historical demise of Pinus nigra forests in the Northern Iberian Plateau (south-western Europe). *Journal of Ecology*, *105*(3), 634–646. https://doi.org/10.1111/1365-2745.12702

Morales, J., Pérez-Jordà, G., Peña-Chocarro, L., Zapata, L., Ruíz-Alonso, M., López-Sáez, J. A., & Linstädter, J. (2013). The origins of agriculture in North-West Africa: macro-botanical remains from Epipalaeolithic and Early Neolithic levels of Ifri Oudadane (Morocco). *Journal of Archaeological Science*, *40*(6), 2659–2669. https://doi.org/10.1016/j.jas.2013.01.026

Muller, S. D., Miramont, C., Bruneton, H., Carré, M., Sottocornola, M., Court-Picon, M., de Beaulieu, J.-L., Nakagawa, T., & Schevin, P. (2012). A palaeoecological perspective for the conservation and restoration of wetland plant communities in the central French Alps, with particular emphasis on alder carr vegetation. *Review of Palaeobotany and Palynology*, *171*, 124–139. https://doi.org/10.1016/j.revpalbo.2011.12.005

Nakagawa, T. (1998). Etudes palynologiques dans les Alpes Françaises centrales et méridionales: histoire de la végétation tardiglaciaire et holocène. *PhD Thesis, Université de Droit dEconomie et des Sciences dAix-Marseille III, France*, 206 pp

Nicol-Pichard, S., & Dubar, M. (1998). Reconstruction of Late-glacial and Holocene environments in southeast France based on the study of a 66-m long core from Biot, Alpes Maritimes. *Vegetation History and Archaeobotany*, *7*(1), 11–15. https://doi.org/10.1007/BF01393413

Noti, R., Leeuwen, J., Colombaroli, D., Vescovi, E., Salvatore, P., La Mantia, T., & Tinner, W. (2009). Mid- and late-Holocene vegetation and fire history at Biviere di Gela, a coastal lake in southern Sicily, Italy. *Vegetation History and Archaeobotany*, *18*, 371–387. https://doi.org/10.1007/s00334-009-0211-0

Nourelbait, M., Rhoujjati, A., Benkaddour, A., Carré, M., Eynaud, F., Martinez, P., & Cheddadi, R. (2016). Climate change and ecosystems dynamics over the last 6000 years in the Middle Atlas, Morocco. *Climate of the Past*, *12*, 1029–1042. https://doi.org/10.5194/cp-12-1029-2016

Nowaczyk, N. R., Arz, H. W., Frank, U., Kind, J., & Plessen, B. (2012). Dynamics of the Laschamp geomagnetic excursion from Black Sea sediments. *Earth and Planetary Science Letters, 351–*

352, 54–69. https://doi.org/10.1016/j.epsl.2012.06.050

Ortu, E., Peyron, O., Bordon, A., de Beaulieu, Jacques-L., Siniscalco, C., & Caramiello, R. (2008). Lateglacial and Holocene climate oscillations in the South-western Alps: An attempt at quantitative reconstruction. *Quaternary International - QUATERN INT, 190,* 71–88. https://doi.org/10.1016/j.quaint.2008.04.004

Ouguerram, A., & Visset, L. (2001). Histoire de la végétation et première mise en évidence d’un milieu marin pendant l’HoIocène dans la vallée de l’Erdre et le Val de Gesvres (Bassin versant de la Loire, Massif armoricain, France). *Quaternaire, 12*(3), 189–199.

Pantaléon-Cano, J., Yll, E. I., Pérez-Obiol, R., & Roure, J. M. (2003). Palynological evidence for vegetational history in semi-arid areas of the western Mediterranean (Almería, Spain). *Holocene, 13*(1), 109–119. https://doi.org/10.1191/0959683603hl598rp

Peñalba, M. C., Arnold, M., Guiot, J., Duplessy, J.-C., & de Beaulieu, J.-L. (1997). Termination of the Last Glaciation in the Iberian Peninsula Inferred from the Pollen Sequence of Quintanar de la Sierra. *Quaternary Research, 48*(2), 205–214. https://doi.org/10.1006/qres.1997.1922

Penalba, M.C. (1989). Dynamique de vegetation Tardiglaciaire et Holocene du centre-nord de l'Espagne d'après l'analyse pollinique. *PhD Thesis, Université dAix-Marseille, Marseille, France*

Penalba, M.C.. (1994). The History of the Holocene Vegetation in Northern Spain from Pollen Analysis. *Journal of Ecology, 82*(4), 815–832. https://doi.org/10.2307/2261446

Pèrez-Obiol, R., & Julià, R. (1994). Climatic Change on the Iberian Peninsula Recorded in a 30,000-Yr Pollen Record from Lake Banyoles. *Quaternary Research*, *41*(1), 91–98. https://doi.org/DOI: 10.1006/qres.1994.1010

Piqué, R., Revelles, J., Burjachs, F., Caruso Fermé, L., & Pérez-Obiol, R. (2018). Interdisciplinary approach to the landscape and firewood exploitation during the Holocene at La Garrotxa (Girona, NE Iberia). *Quaternary International*, *463,* 401–413. https://doi.org/10.1016/j.quaint.2016.11.025

Pons, A., & Reille, M. (1988). The holocene- and upper pleistocene pollen record from Padul (Granada, Spain): A new study. *Palaeogeography, Palaeoclimatology, Palaeoecology, 66*(3), 243–263. https://doi.org/10.1016/0031-0182(88)90202-7

Pulido, M. (2006). *Conséquenses de l’anthropisation sur la dynamique postglaciaire de la végétation dans le sud du Massif Central, France*. Université d’Aix-Marseille, Marseille, France.

Reille, M., & de Beaulieu, J. L. (1988). History of the Würm and Holocene vegetation in western velay (Massif Central, France): A comparison of pollen analysis from three corings at Lac du Bouchet. *Review of Palaeobotany and Palynology, 54*(3), 233–248. https://doi.org/10.1016/0034-6667(88)90016-4

Reille, M., & de Beaulieu, J. L. (1990). Pollen analysis of a long upper Pleistocene continental sequence in a Velay maar (Massif Central, France). *Palaeogeography, Palaeoclimatology, Palaeoecology, 80*(1), 35–48. https://doi.org/10.1016/0031-0182(90)90032-3

Revelles, J., Burjachs, F., Palomo, A., Piqué, R., Iriarte, E., Pérez-Obiol, R., & Terradas, X. (2018). Human-environment interaction during the Mesolithic- Neolithic transition in the NE Iberian Peninsula. Vegetation history, climate change and human impact during the Early-Middle Holocene in the Eastern Pre-Pyrenees. *Quaternary Science Reviews, 184*, 183–200. https://doi.org/10.1016/j.quascirev.2017.08.025

Revelles, J., Ghilardi, M., Rossi, V., Currás, A., López-Bultó, O., Brkojewitsch, G., & Vacchi, M. (2019). Coastal landscape evolution of Corsica island (W. Mediterranean): palaeoenvironments,

vegetation history and human impacts since the early Neolithic period. *Quaternary Science Reviews, 225*, 105993. https://doi.org/10.1016/j.quascirev.2019.105993

Rösch, M., E. Fischer, Lechterbeck, J., & Wick., L. (2012). Pollenanalysen an drei Bohrkernen aus dem Profundal des Ortasees (Piemont, Italien). In A. S. and U. Tegtmeier (Ed.), *Verzweigungen, eine Würdigung für A.J. Kalis und J. Meurers- Balke.* (pp. 225–247). Frankfurter Archäologische Schriften.

Roucoux, K. H., de Abreu, L., Shackleton, N. J., & Tzedakis, P. C. (2005). The response of NW Iberian vegetation to North Atlantic climate oscillations during the last 65kyr. *Quaternary Science Reviews*, *24*(14), 1637–1653. https://doi.org/10.1016/j.quascirev.2004.08.022

Samartin, S., Heiri, O., Joos, F., Renssen, H., Franke, J., Brönnimann, S., & Tinner, W. (2017). Warm Mediterranean mid-Holocene summers inferred from fossil midge assemblages. *Nature Geoscience*, *10*(3), 207–212. https://doi.org/10.1038/ngeo2891

Schönfeld, J., Zahn, R., & Abreu, L. (2003). Surface and deep water response to rapid climate changes at the Western Iberian Margin. *Global and Planetary Change*, *36*, 237–264. https://doi.org/10.1016/S0921-8181(02)00197-2

Shumilovskikh, L., Fleitmann, D., Nowaczyk, N., Behling, H., Marret, F., Wegwerth, A., & Arz, H. (2014). Orbital- and millennial-scale environmental changes between 64 and 20 ka BP recorded in Black Sea sediments. *Climate of the Past*, *10,* 939–954. https://doi.org/10.5194/cp-10-939-2014

Shumilovskikh, L. S., Tarasov, P., Arz, H. W., Fleitmann, D., Marret, F., Nowaczyk, N., Plessen, B., Schlütz, F., & Behling, H. (2012). Vegetation and environmental dynamics in the southern Black Sea region since 18kyr BP derived from the marine core 22-GC3. *Palaeogeography, Palaeoclimatology, Palaeoecology*, *337–338*, 177–193. https://doi.org/10.1016/j.palaeo.2012.04.015

Tastet, J.-P., & Diot, M.-F. (1995). Paléo-environnements holocènes et limites chronoclimatiques enregistrés dans un marais estuarien de la Gironde (France) [Holocene paleo-environments and chrono-climatic limits recorded in a reclaimed marsh of the Gironde estuary (France).]. *Quaternaire,* 63–75.

Thouveny, N., de Beaulieu, J.-L., Bonifay, E., Creer, K. M., Guiot, J., Icole, M., Johnsen, S., Jouzel, J., Reille, M., Williams, T., & Williamson, D. (1994). Climate variations in Europe over the past 140 kyr deduced from rock magnetism. *Nature*, *371*(6497), 503–506. https://doi.org/10.1038/371503a0

Tinner, W., Hubschmid, P., Wehrli, M., Ammann, B., & Conedera, M. (1999). Long-term forest fire ecology and dynamics in southern Switzerland. *Journal of Ecology*, *87*(2), 273–289. https://doi.org/10.1046/j.1365-2745.1999.00346.x

Tinner, W., Leeuwen, J., Colombaroli, D., Vescovi, E., Knaap, W. O., Henne, P., Salvatore, P., D'Angelo, S., & La Mantia, T. (2009). Holocene environmental and climatic changes at Gorgo Basso, a coastal lake in southern Sicily, Italy. *Quaternary Science Reviews, 28*, 1498–1510. https://doi.org/10.1016/j.quascirev.2009.02.001

Tinner, W., Vescovi, E., Leeuwen, J., Colombaroli, D., Henne, P., Kaltenrieder, P., Morales-Molino, C., Beffa, G., Gnägi, B., Knaap, W., La Mantia, T., & Salvatore, P. (2016). Holocene vegetation and fire history of the mountains of Northern Sicily (Italy). *Vegetation History and Archaeobotany, 25*. https://doi.org/10.1007/s00334-016-0569-8

Turner, J., & Greig, J. R. A. (1975). Some Holocene pollen diagrams from Greece. *Review of Palaeobotany and Palynology, 20*(3), 171–204. https://doi.org/10.1016/0034-6667(75)90020-2

van den Brink, L. M., & Janssen, C. R. (1985). The effect of human activities during cultural phases on the development of montane vegetation in the Serra de Estrela, Portugal. *Review of Palaeobotany and Palynology*, *44*(3), 193–215. https://doi.org/10.1016/0034-6667(85)90016-8

van der Knaap, W. O., & van Leeuwen, J. F. N. (1995). Holocene vegetation succession and degradation as responses to climatic change and human activity in the Serra de Estrela, Portugal. *Review of Palaeobotany and Palynology*, *89*(3), 153–211. https://doi.org/10.1016/0034-6667(95)00048-0

van der Knaap, W. O., & van Leeuwen, J. F. N. (1997). Late Glacial and early Holocene vegetation succession, altitudinal vegetation zonation, and climatic change in the Serra da Estrela, Portugal. *Review of Palaeobotany and Palynology*, *97*(3), 239–285. https://doi.org/10.1016/S0034-6667(97)00008-0

Vannière, B., Colombaroli, D., Charpon, E., Leroux, A., Tinner, W., & Magny, M. (2008). Climate versus human-driven fire regimes in Mediterranean landscapes: the Holocene record of Lago dell'Accesa (Tuscany, Italy). *Quaternary Science Reviews*, *27*, 1181–1196. https://doi.org/10.1016/j.quascirev.2008.02.011

Vella, C., & Provansal, M. (2000). Relative sea-level rise and neotectonic events during the last 6500yr on the southern eastern Rhône delta, France. *Marine Geology*, *170*(1), 27–39. https://doi.org/10.1016/S0025-3227(00)00063-3

Vescovi, E., Ammann, B., Ravazzi, C., & Tinner, W. (2010). A new Late-glacial and Holocene record of vegetation and fire history from Lago del Greppo, northern Apennines, Italy. *Vegetation History and Archaeobotany*, *19*, 219–233. https://doi.org/10.1007/s00334-010-0243-5

Visset, L., & Bernard, J. (2006). Évolution du littoral et du paysage, de la presqu'île de Rhuys à la rivière d'Étel (Massif armoricain – France), du Néolithique au Moyen Âge. *ArcheoSciences*, *30*, 143–156.

Visset, L., Hauray, G., Charrieau, L., & Rouzeau, N. (2001). Paléoenvironnement urbain : Histoire du comblement des vallées de la métropole nantaise, du Tardiglaciaire à la fin de l'Holocène. *Annales de Bretagne et Des Pays de L'Ouest*, *108*, 147–165.

Visset, L., L'helgouach, J., & Bernard, J. (1996). La tourbière submergée de la pointe de Kerpenhir à Locmariaquer (Morbihan). *Etude Environnementale et Mise En Évidence de Déforestations et de Pratiques Agricoles Néolithiques.*

von Engelbrechten, S. (1998). *Late-glacial and Holocene Vegetation and Environmental History of the Sierra de Urbion, North-Central Spain. Doctoral dissertation.* University of Dublin.

Watts, W. A., Allen, J. R. M., & Huntley, B. (1996). Vegetation history and palaeoclimate of the last glacial period at Lago Grande di Monticchio, Southern Italy. *Quaternary Science Reviews*, *15*(2), 133–153. https://doi.org/10.1016/0277-3791(95)00093-3

Yll, E.-I., Perez-Obiol, R., Pantaleon-Cano, J., & Roure, J. M. (1997). Palynological Evidence for Climatic Change and Human Activity during the Holocene on Minorca (Balearic Islands). *Quaternary Research*, *48*(3), 339–347. https://doi.org/10.1006/qres.1997.1925

Yll, E. I., Pérez-Obiol, R., & Julià, R. (1994). Vegetational change in the Balearic Islands (Spain) during the Holocene. *Historical Biology*, *9*(1–2), 83–89. https://doi.org/10.1080/10292389409380490

Zapata, L., López-Sáez, J. A., Ruiz-Alonso, M., Linstädter, J., Pérez-Jordà, G., Morales, J., Kehl, M., & Peña-Chocarro, L. (2013). Holocene environmental change and human impact in NE Morocco: Palaeobotanical evidence from Ifri Oudadane. *The Holocene*, *23*(9), 1286–1296. https://doi.org/10.1177/0959683613486944

S6 Table. Areas spatial cell numbers of habitat loss, habitat gain and habitat stability over time under future conditions, for each time period using CCSM4 climate model.

Models	Year	Scenario	Stable absent	Stable presence	Habitat Loss	Habitat Loss (%)	Habitat Gain	Habitat Gain (%)	Species Range Change (%)
ANN	2050	RCP4.5	545030	119247	6436	5	37248	30	25
		RCP8.5	519257	114600	7516	6	66588	55	48
	2070	RCP4.5	536532	116313	9370	7	45746	36	29
		RCP8.5	506823	116076	9607	8	75455	60	52
CTA	2050	RCP4.5	542762	115151	6965	6	43083	35	30
		RCP8.5	519257	114600	7516	6	66588	55	48
	2070	RCP4.5	524737	113511	8605	7	61108	50	43
		RCP8.5	483911	111556	10560	9	101934	83	75
FDA	2050	RCP4.5	546715	116426	7795	6	37025	30	24
		RCP8.5	532463	116145	8076	7	51277	41	35
	2070	RCP4.5	536468	117091	7130	6	47272	38	32
		RCP8.5	505919	112465	11756	9	77821	63	53
GAM	2050	RCP4.5	564784	100893	13184	12	29100	26	14
		RCP8.5	551222	95578	18499	16	42662	37	21
	2070	RCP4.5	554135	95758	18319	16	39749	35	19
		RCP8.5	527204	84892	29185	26	66680	58	33
GBM	2050	RCP4.5	567705	98475	13707	12	28074	25	13
		RCP8.5	554358	92922	19260	17	41421	37	20
	2070	RCP4.5	557340	93078	19104	17	38439	34	17
		RCP8.5	530529	81697	30485	27	65250	58	31
GLM	2050	RCP4.5	555140	93872	24618	21	34331	29	8
		RCP8.5	542159	83957	34533	29	47312	40	11
	2070	RCP4.5	545310	87569	30921	26	44161	37	11
		RCP8.5	522307	68512	49978	42	67164	57	15
MARS	2050	RCP4.5	558650	103026	11699	10	34586	30	20
		RCP8.5	543925	99925	14800	13	49311	43	30
	2070	RCP4.5	546874	99195	15530	14	46362	40	27
		RCP8.5	519091	91682	23043	20	74145	65	45
MAXENT	2050	RCP4.5	558036	100981	12319	11	36625	32	21
		RCP8.5	542272	100688	12612	11	52389	46	35
	2070	RCP4.5	542825	99229	14071	12	51836	46	33
		RCP8.5	520231	94688	18612	16	74430	66	49
RF	2050	RCP4.5	570080	90364	14921	14	32596	31	17
		RCP8.5	551219	85157	20128	19	51457	49	30
	2070	RCP4.5	553769	86022	19263	18	48907	46	28
		RCP8.5	527176	77219	28066	27	75500	72	45
SRE	2050	RCP4.5	612648	46838	26878	36	21597	29	-7
		RCP8.5	599873	40207	33509	45	34372	47	1
	2070	RCP4.5	605157	41287	32429	44	29088	39	-5
		RCP8.5	586595	30079	43637	59	47650	65	5

S7 Table. Areas spatial cell numbers of habitat loss, habitat gain and habitat stability over time under future conditions, for each time period using MIROC-ESM climate model.

Models	Year	Scenario	Stable absent	Stable presence	Habitat Loss	Habitat Loss (%)	Habitat Gain	Habitat Gain (%)	Species Range Change (%)
ANN	2050	RCP4.5	527537	124150	1533	1	54741	44	42
		RCP8.5	456909	115463	6653	5	128936	106	100
	2070	RCP4.5	516974	120841	4842	4	65304	52	48
		RCP8.5	485961	110626	15057	12	96317	77	65
CTA	2050	RCP4.5	490888	118741	3375	3	94957	78	75
		RCP8.5	456909	115463	6653	5	128936	106	100
	2070	RCP4.5	465175	116810	5306	4	120670	99	94
		RCP8.5	409707	108377	13739	11	176138	144	133
FDA	2050	RCP4.5	510457	111490	12731	10	73283	59	49
		RCP8.5	489425	104132	20089	16	94315	76	60
	2070	RCP4.5	499481	106081	18140	15	84259	68	53
		RCP8.5	464531	91975	32246	26	119209	96	70
GAM	2050	RCP4.5	537747	87318	26759	23	56137	49	26
		RCP8.5	520934	75391	38686	34	72950	64	30
	2070	RCP4.5	523310	68941	45136	40	70574	62	22
		RCP8.5	486987	57796	56281	49	106897	94	44
GBM	2050	RCP4.5	541135	84639	27543	25	54644	49	24
		RCP8.5	524922	72551	39631	35	70857	63	28
	2070	RCP4.5	527076	66185	45997	41	68703	61	20
		RCP8.5	492899	55238	56944	51	102880	92	41
GLM	2050	RCP4.5	535769	76558	41932	35	53702	45	10
		RCP8.5	531012	63874	54616	46	58459	49	3
	2070	RCP4.5	529946	61916	56574	48	59525	50	2
		RCP8.5	527602	45452	73038	62	61869	52	-9
MARS	2050	RCP4.5	538167	92851	21874	19	55069	48	29
		RCP8.5	525964	81994	32731	29	67272	59	30
	2070	RCP4.5	528177	80006	34719	30	65059	57	26
		RCP8.5	511406	62711	52014	45	81830	71	26
MAXENT	2050	RCP4.5	513560	98352	14948	13	81101	72	58
		RCP8.5	496547	90412	22888	20	98114	87	66
	2070	RCP4.5	502600	88829	24471	22	92061	81	60
		RCP8.5	467709	71750	41550	37	126952	112	75
RF	2050	RCP4.5	535664	88418	16867	16	67012	64	48
		RCP8.5	516889	78862	26423	25	85787	81	56
	2070	RCP4.5	524816	76781	28504	27	77860	74	47
		RCP8.5	493575	64542	40743	39	109101	104	65
SRE	2050	RCP4.5	613545	34787	38929	53	20700	28	-25
		RCP8.5	611889	23112	50604	69	22356	30	-38
	2070	RCP4.5	605169	23681	50035	68	29076	39	-28
		RCP8.5	605838	15230	58486	79	28407	39	-41

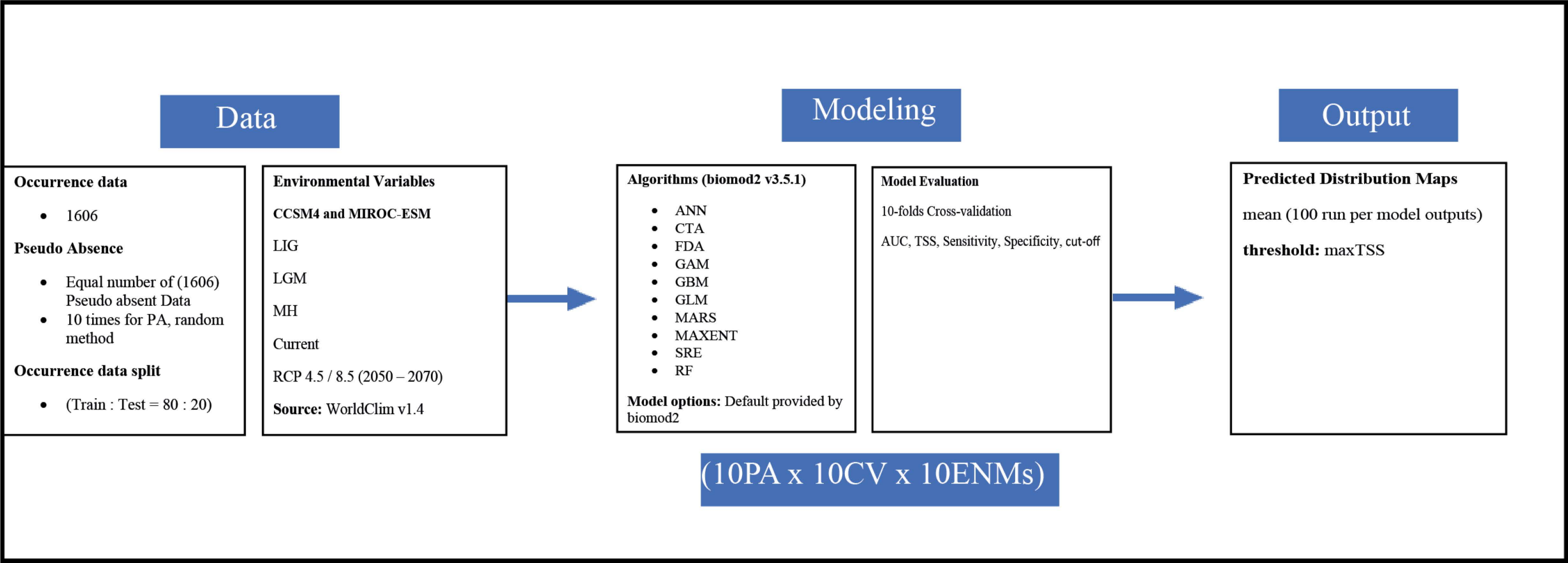

Data
Modeling
Output
Occurrence data
1606
Pseudo Absence
Equal number of (1606) Pseudo absent Data
10 times for PA, random method
Occurrence data split
(Train : Test = 80 : 20)
Environmental Variables
CCSM4 and MIROC-ESM
LIG
LGM
MH
Current
RCP 4.5 / 8.5 (2050 – 2070)
Source: WorldClim v1.4
Algorithms (biomod2 v3.5.1)
ANN
CTA
FDA
GAM
GBM
GLM
MARS
MAXENT
SRE
RF
Model options: Default provided by biomod2
Model Evaluation
10-folds Cross-validation
AUC, TSS, Sensitivity, Specificity, cut-off
Predicted Distribution Maps
mean (100 run per model outputs)
threshold: maxTSS
(10PA x 10CV x 10ENMs)

a) Uncertinity maps (CCSM4)

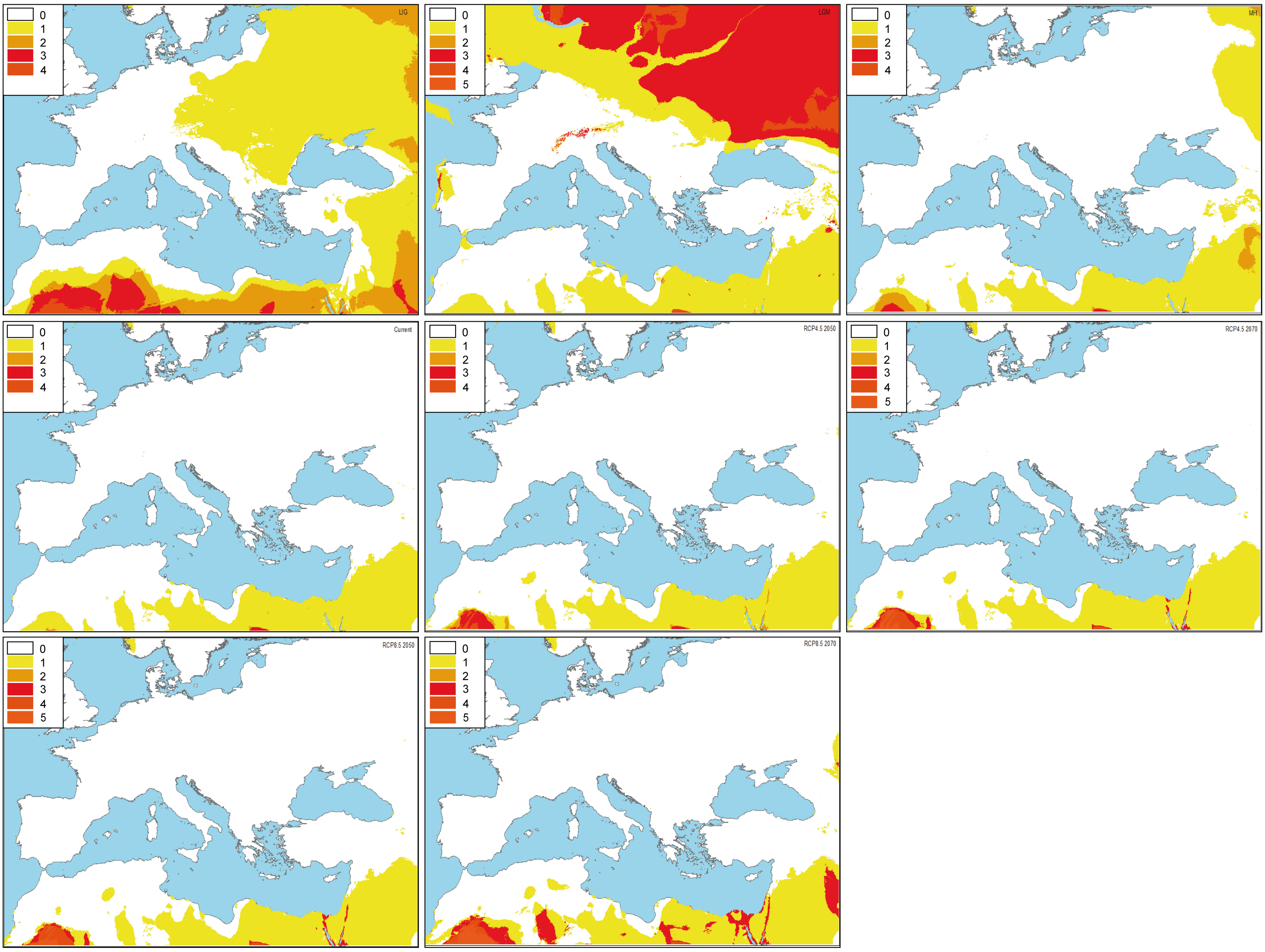

b) Uncertinity maps (MIROC-ESM)

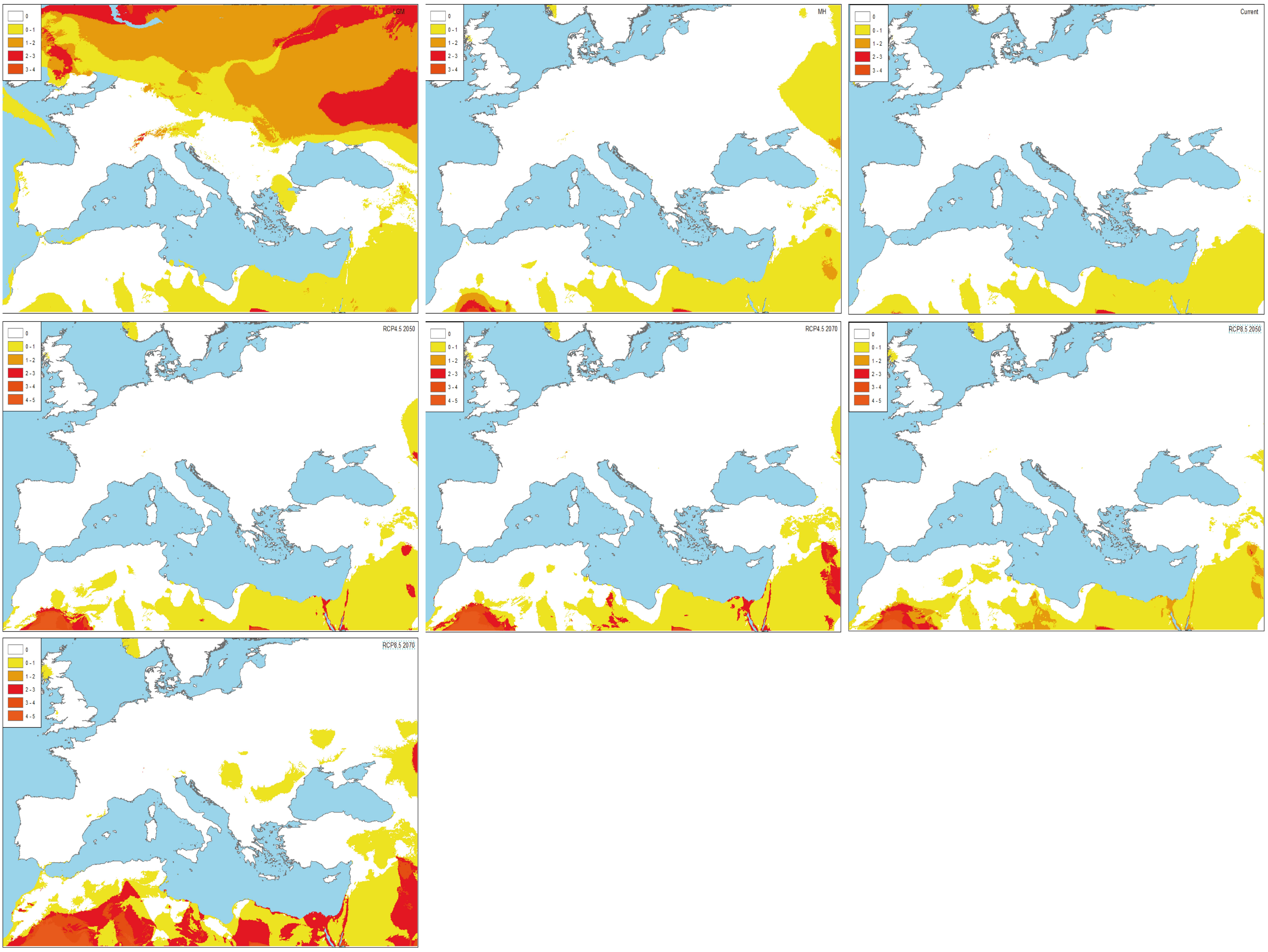

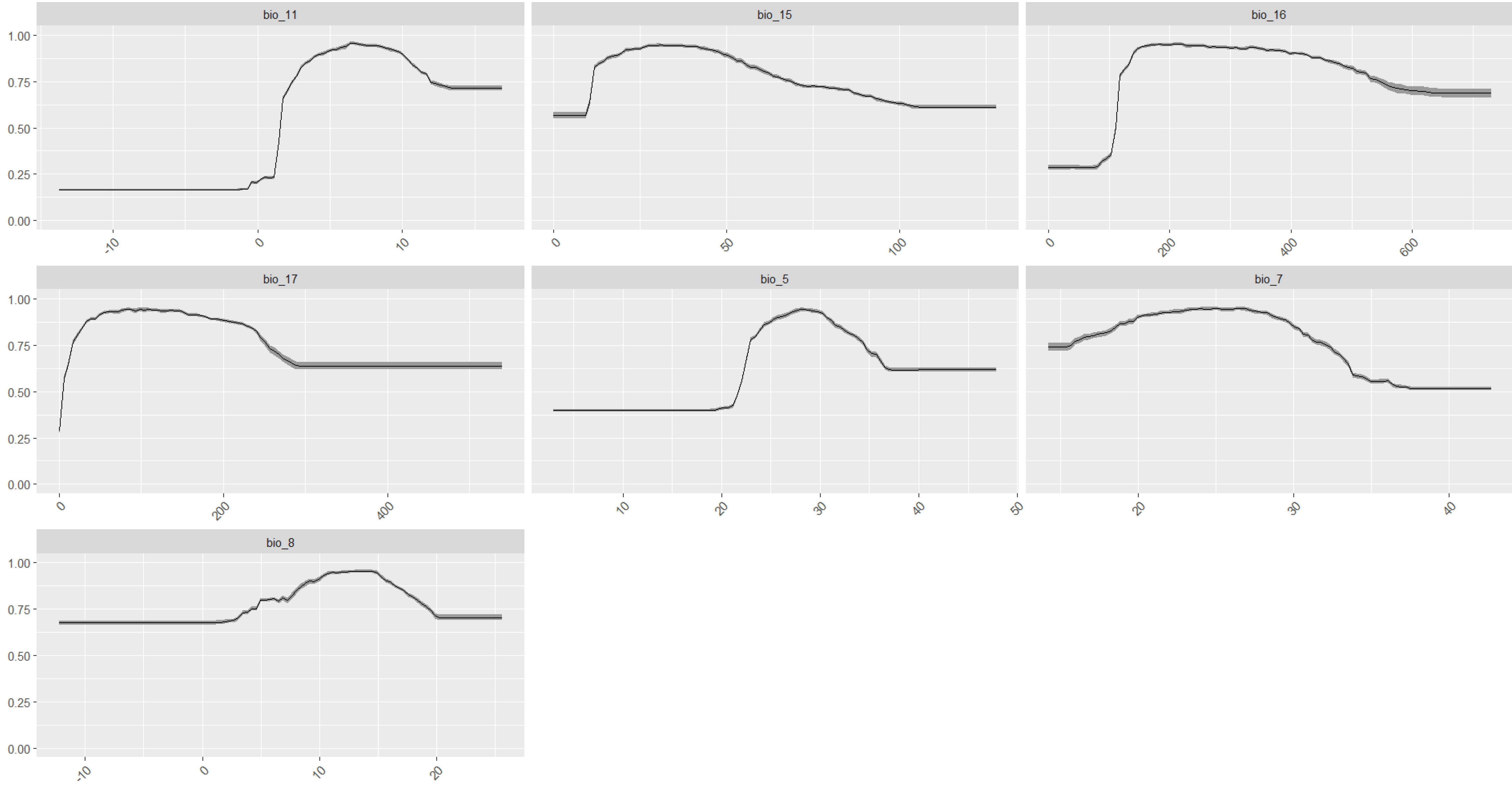

bio_11
bio_15
bio_16
bio_17
bio_5
bio_7
bio_8
0.00
0.25
0.50
0.75
1.00
-10
0
10
20
50
100
200
400
600
30
40

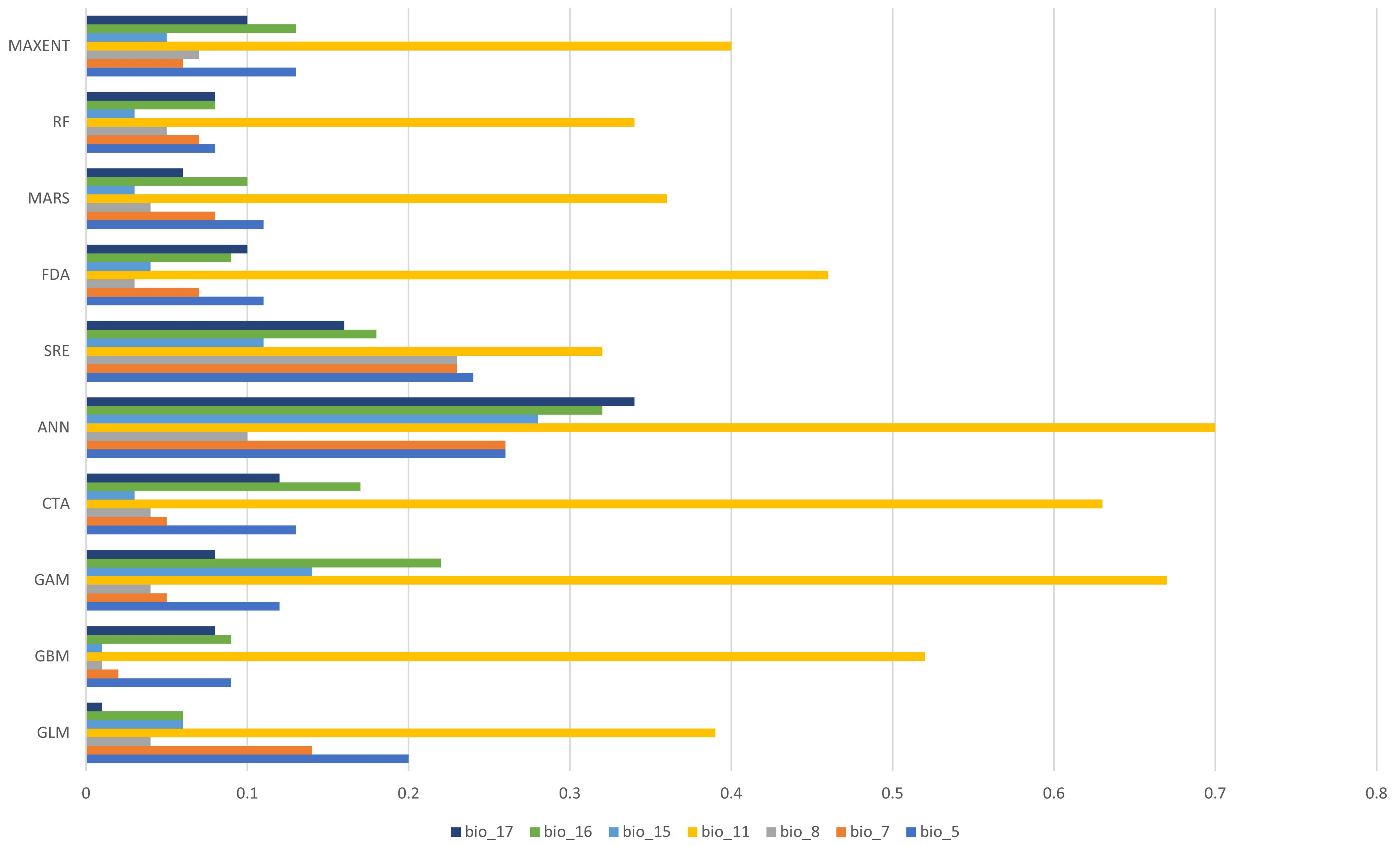

	GLM	GBM	GAM	CTA	ANN	SRE	FDA	MARS	RF	MAXENT
bio_5	0.2	0.09	0.12	0.13	0.26	0.24	0.11	0.11	0.08	**0.13**
bio_7	0.14	0.02	0.05	0.05	0.26	0.23	0.07	0.08	0.07	0.06
bio_8	0.04	0.01	0.04	0.04	0.1	0.23	0.03	0.04	0.05	0.07
bio_11	**0.39**	**0.52**	**0.67**	**0.63**	0.7	**0.32**	**0.46**	**0.36**	**0.34**	0.4
bio_15	0.06	0.01	0.14	0.03	0.28	0.11	0.04	0.03	0.03	0.05
bio_16	0.06	0.09	0.22	0.17	0.32	0.18	0.09	0.1	0.08	**0.13**
bio_17	0.01	0.08	0.08	0.12	**0.34**	0.16	0.1	0.06	0.08	0.1

ANN Projections (CCSM4)

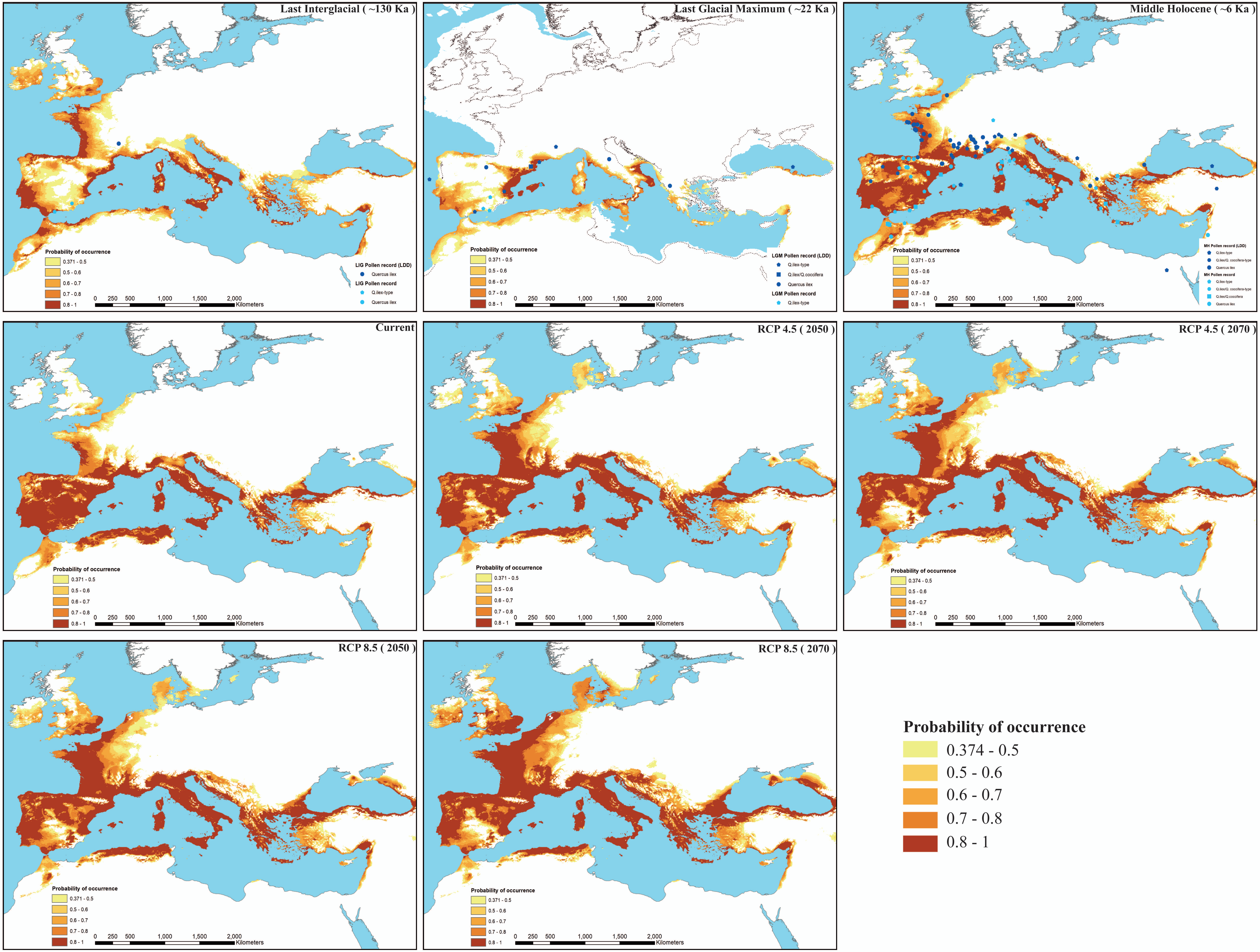

CTA Projections (CCSM4)

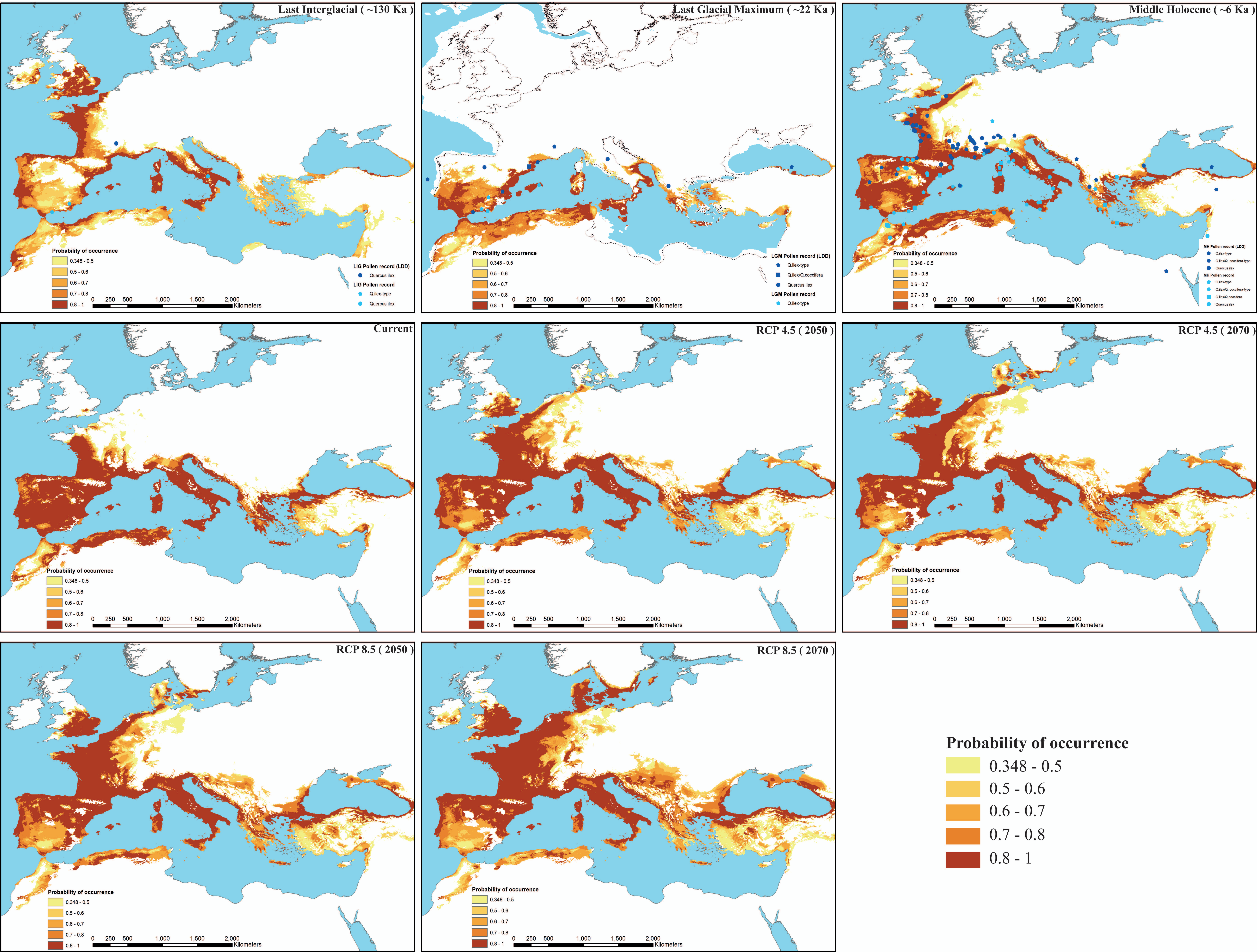

FDA Projections (CCSM4)

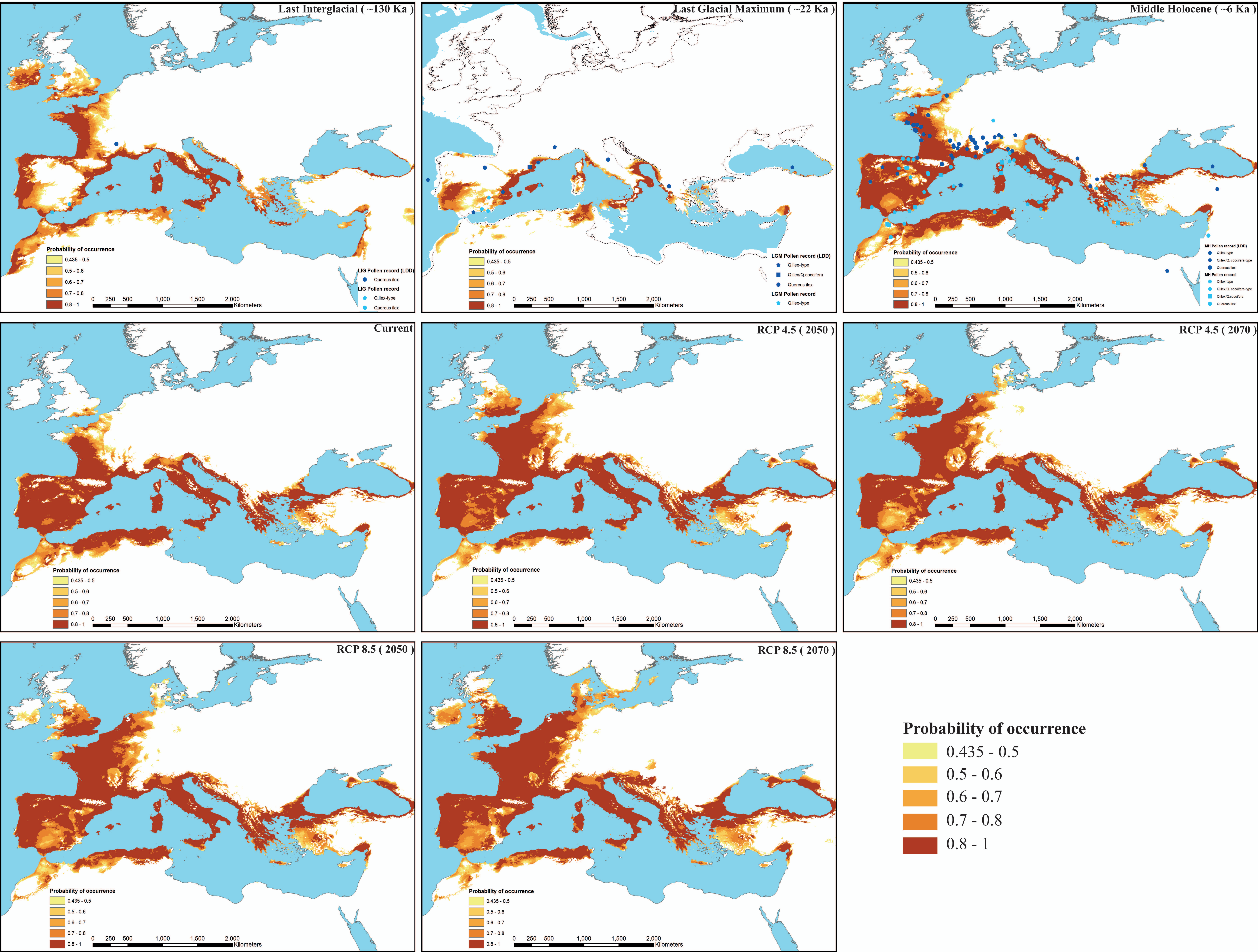

GAM Projections (CCSM4)

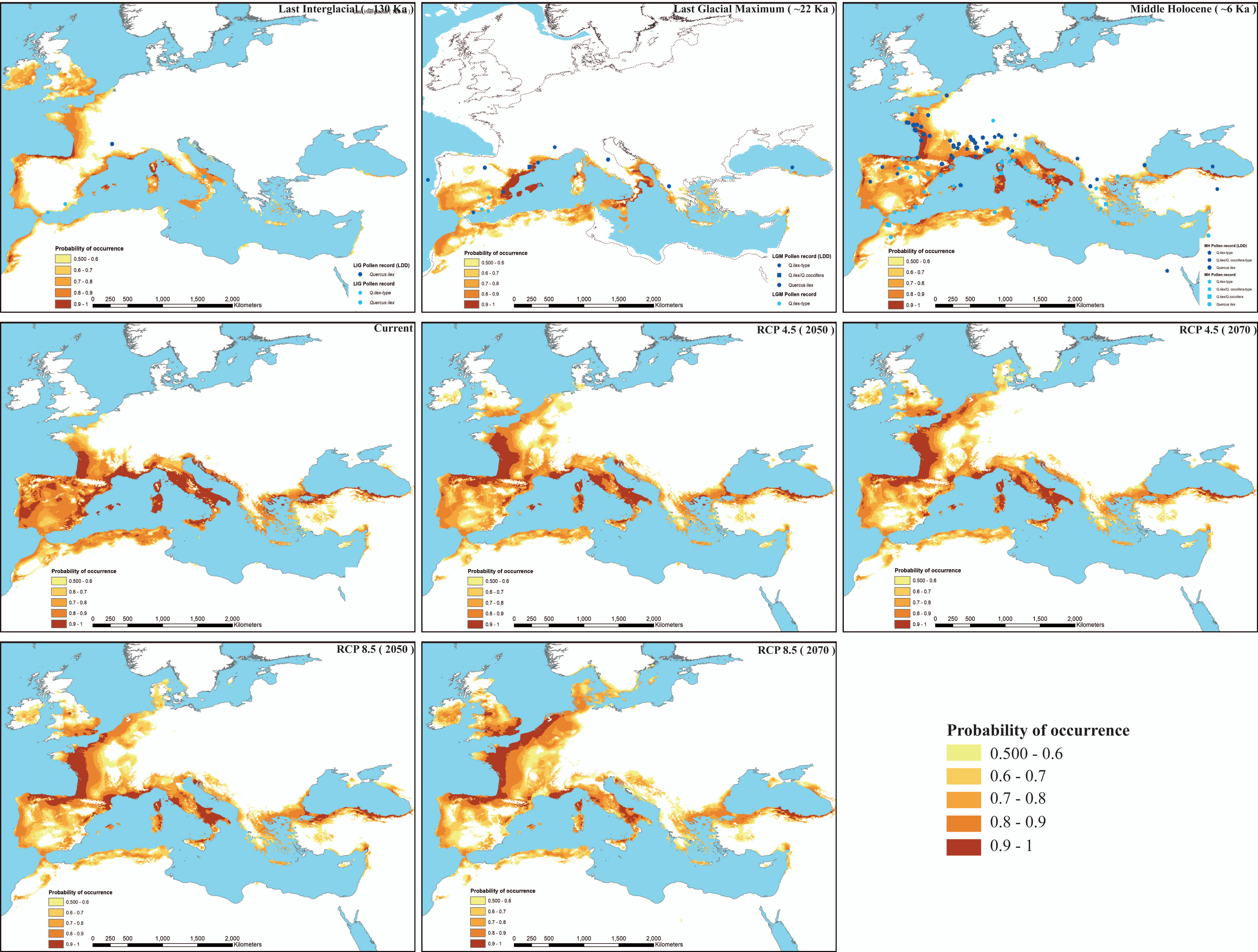

GBM Projections (CCSM4)

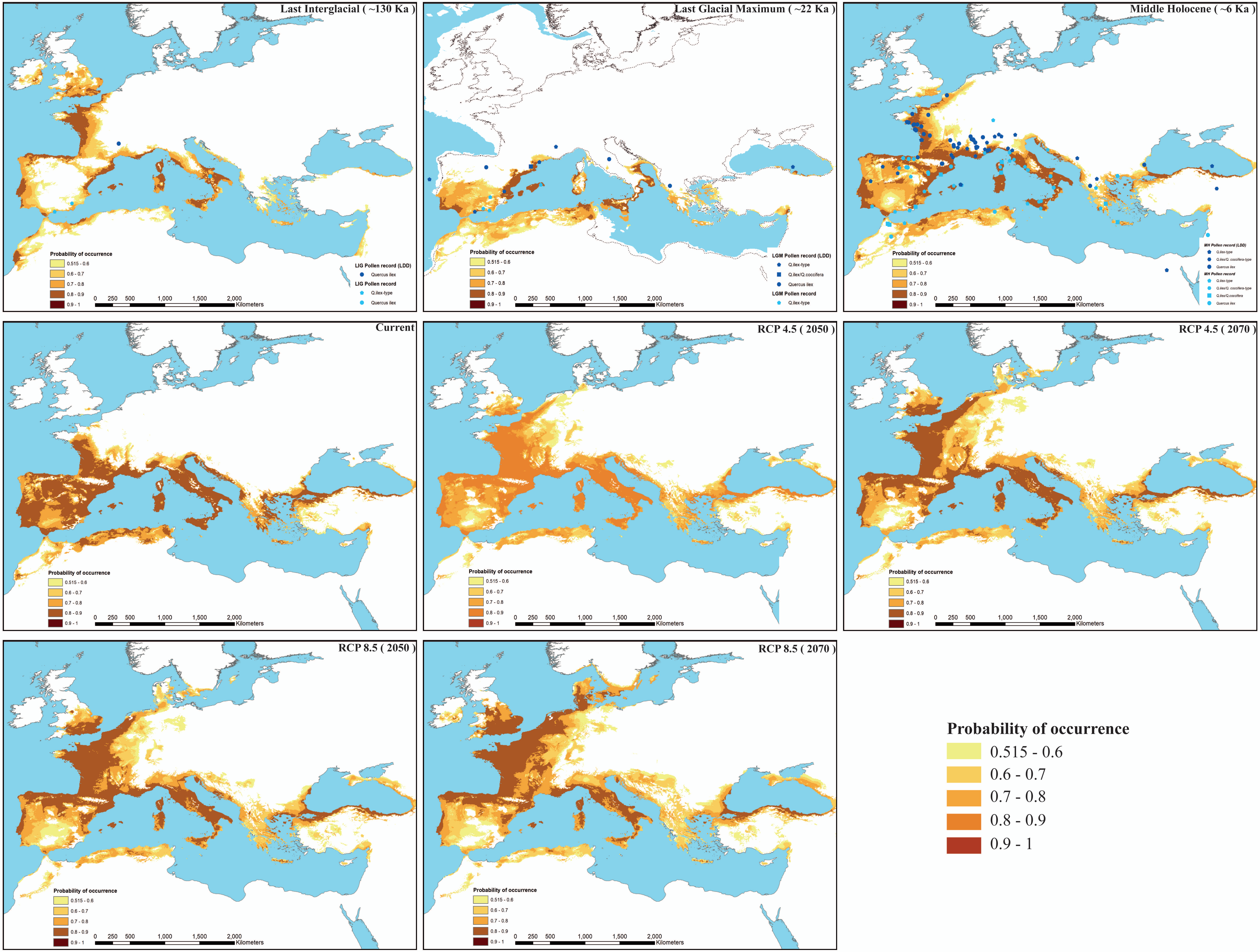

GLM Projections (CCSM4)

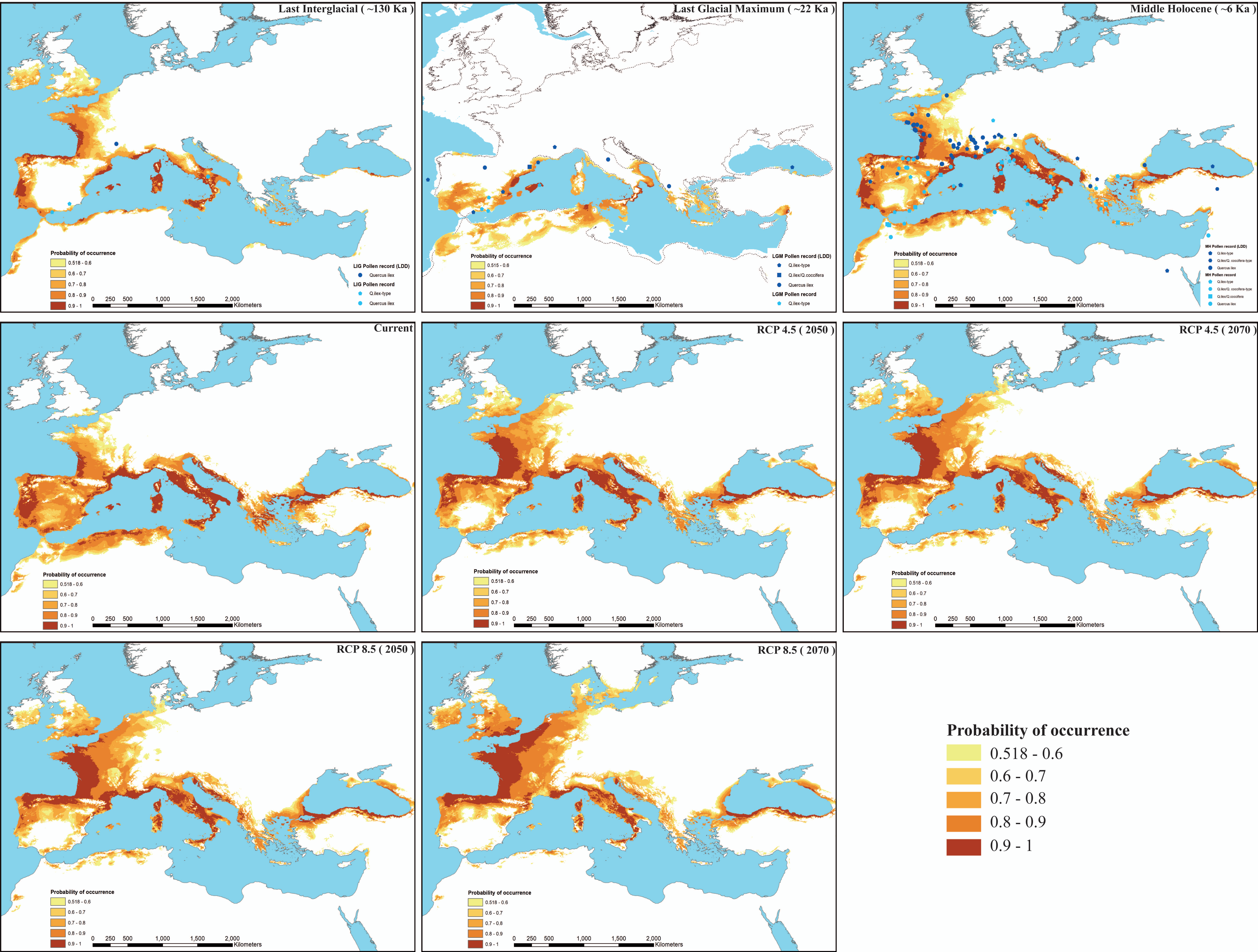

MARS Projections (CCSM4)

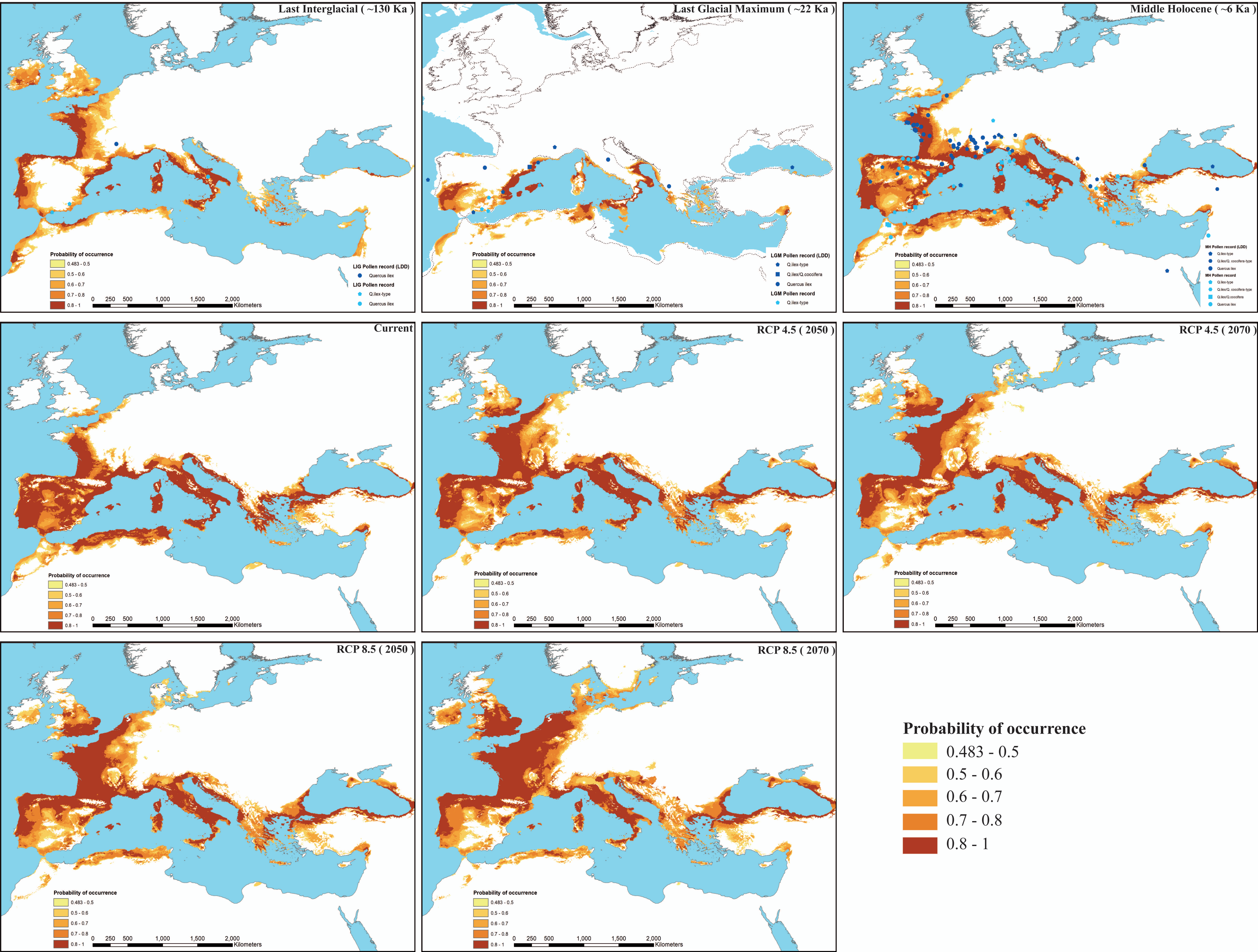

MAXENT Projections (CCSM4)

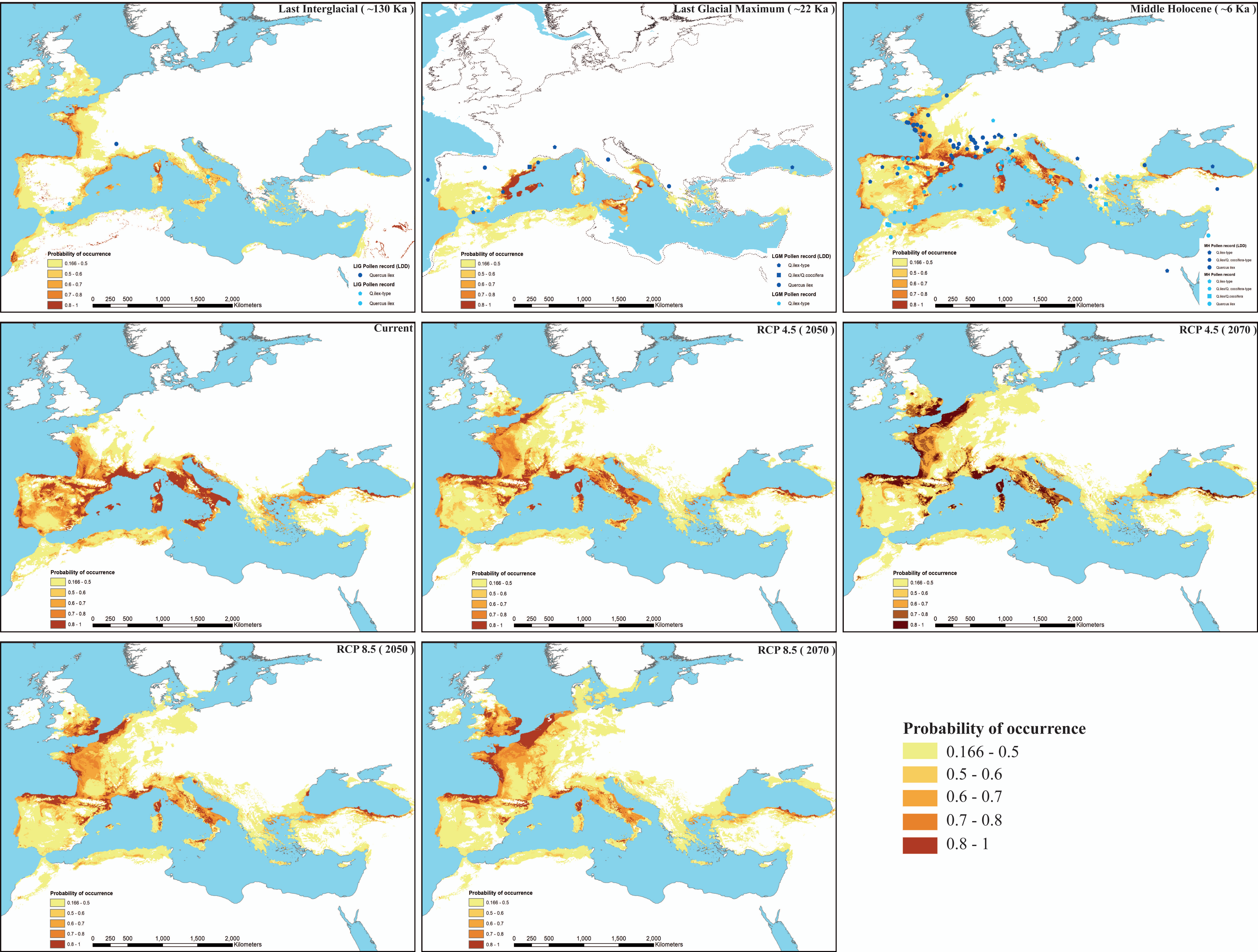

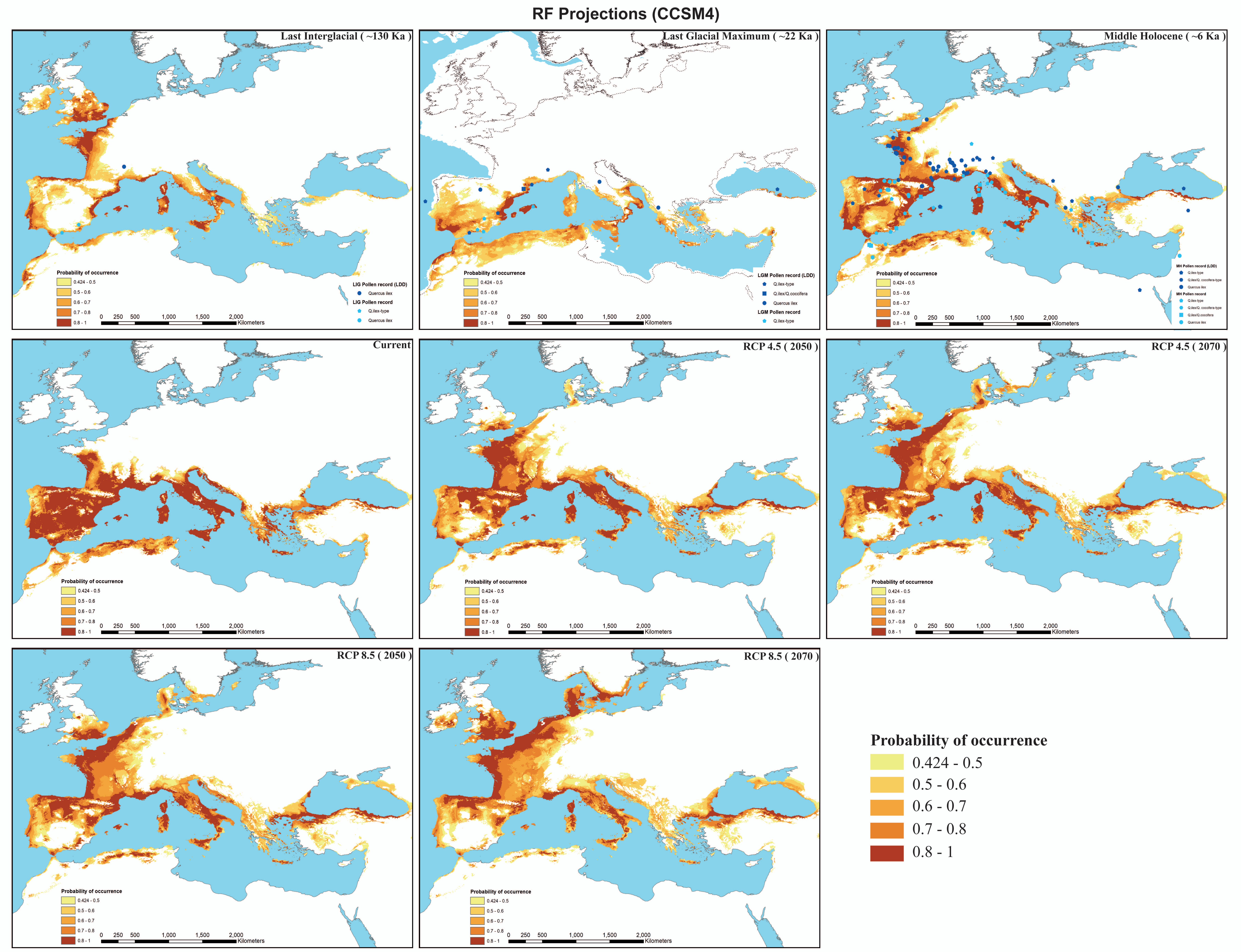

RF Projections (CCSM4)
Last Interglacial (~130 Ka)
Probability of occurrence
0.424 - 0.5
0.5 - 0.6
0.6 - 0.7
0.7 - 0.8
0.8 - 1
0 250 500 1,000 1,500 2,000 Kilometers
LIG Pollen record (LDD)
Quercus ilex
LIG Pollen record
Q.ilex-type
Quercus ilex
Last Glacial Maximum (~22 Ka)
Probability of occurrence
0.424 - 0.5
0.5 - 0.6
0.6 - 0.7
0.7 - 0.8
0.8 - 1
0 250 500 1,000 1,500 2,000 Kilometers
LGM Pollen record (LDD)
Q.ilex-type
Q.ilex/Q.coccifera
Quercus ilex
LGM Pollen record
Q.ilex-type
Middle Holocene (~6 Ka)
Probability of occurrence
0.424 - 0.5
0.5 - 0.6
0.6 - 0.7
0.7 - 0.8
0.8 - 1
0 250 500 1,000 1,500 2,000 Kilometers
MH Pollen record (LDD)
Q.ilex-type
Q.ilex/Q. coccifera-type
Quercus ilex
MH Pollen record
Q.ilex-type
Q.ilex/Q. coccifera-type
Q.ilex/Q.coccifera
Quercus ilex
Current
Probability of occurrence
0.424 - 0.5
0.5 - 0.6
0.6 - 0.7
0.7 - 0.8
0.8 - 1
0 250 500 1,000 1,500 2,000 Kilometers
RCP 4.5 (2050)
Probability of occurrence
0.424 - 0.5
0.5 - 0.6
0.6 - 0.7
0.7 - 0.8
0.8 - 1
0 250 500 1,000 1,500 2,000 Kilometers
RCP 4.5 (2070)
Probability of occurrence
0.424 - 0.5
0.5 - 0.6
0.6 - 0.7
0.7 - 0.8
0.8 - 1
0 250 500 1,000 1,500 2,000 Kilometers
RCP 8.5 (2050)
Probability of occurrence
0.424 - 0.5
0.5 - 0.6
0.6 - 0.7
0.7 - 0.8
0.8 - 1
0 250 500 1,000 1,500 2,000 Kilometers
RCP 8.5 (2070)
Probability of occurrence
0.424 - 0.5
0.5 - 0.6
0.6 - 0.7
0.7 - 0.8
0.8 - 1
0 250 500 1,000 1,500 2,000 Kilometers
Probability of occurrence
0.424 - 0.5
0.5 - 0.6
0.6 - 0.7
0.7 - 0.8
0.8 - 1

SRE Projections (CCSM4)

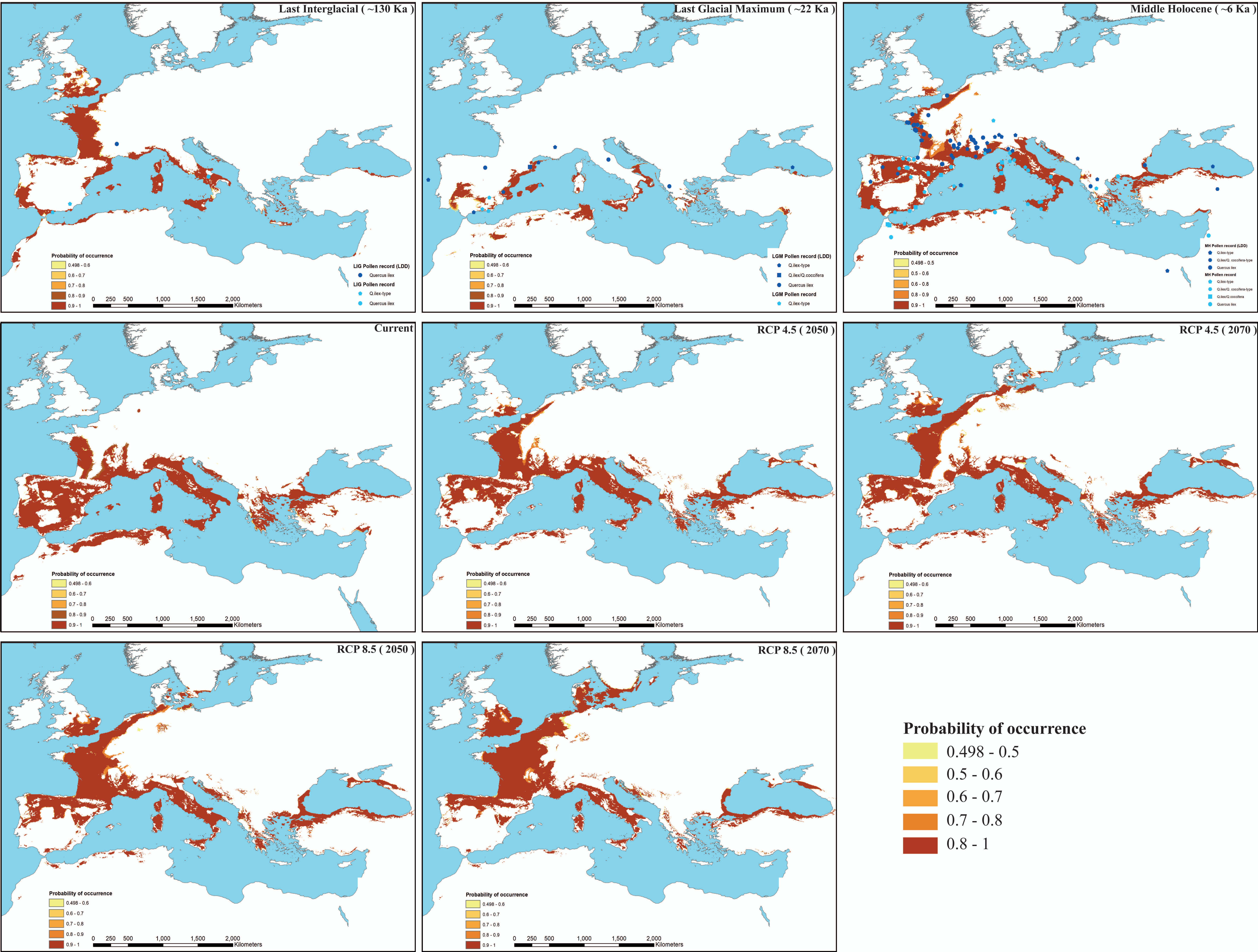

ANN Projections (MIROC-ESM)

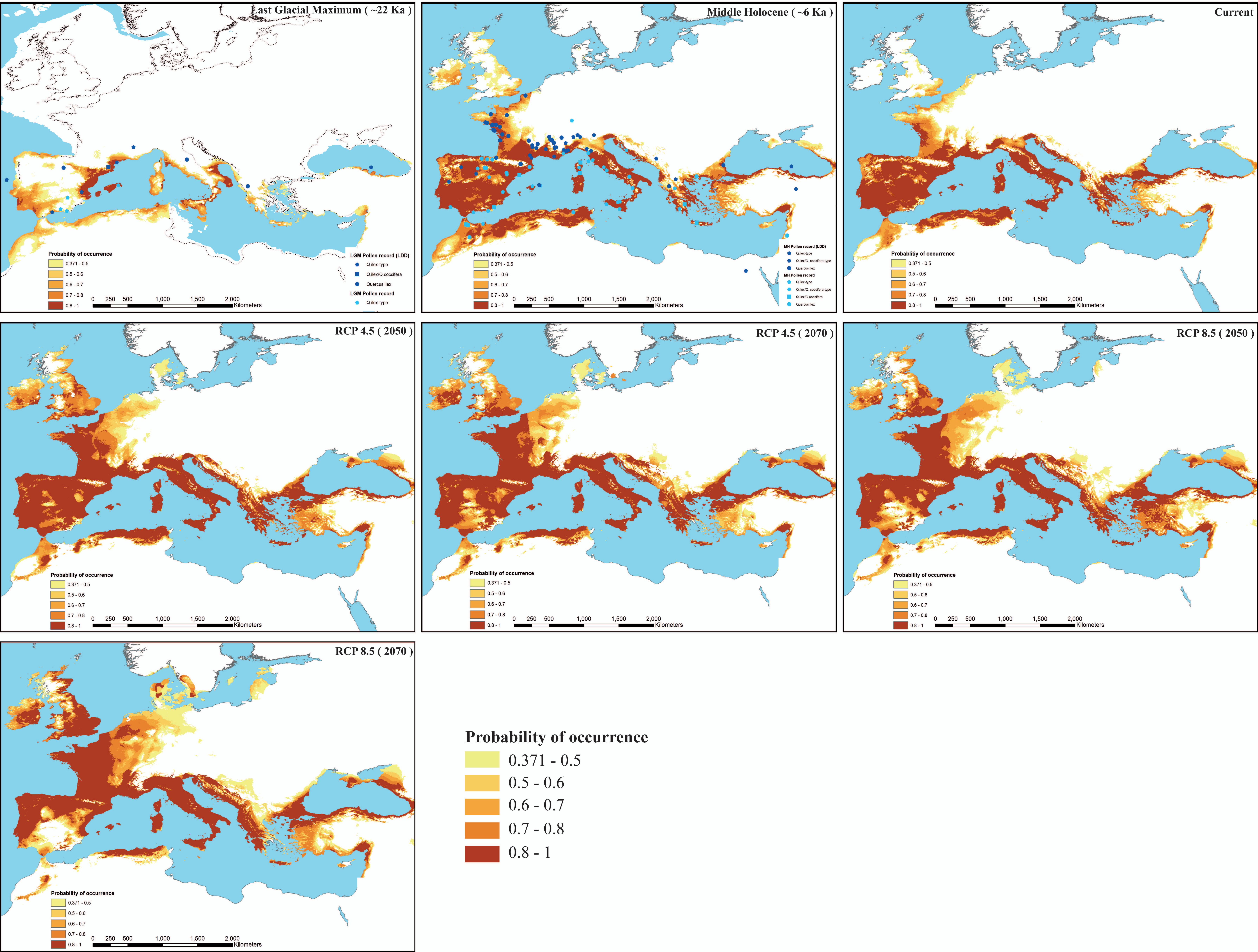

CTA Projections (MIROC-ESM)

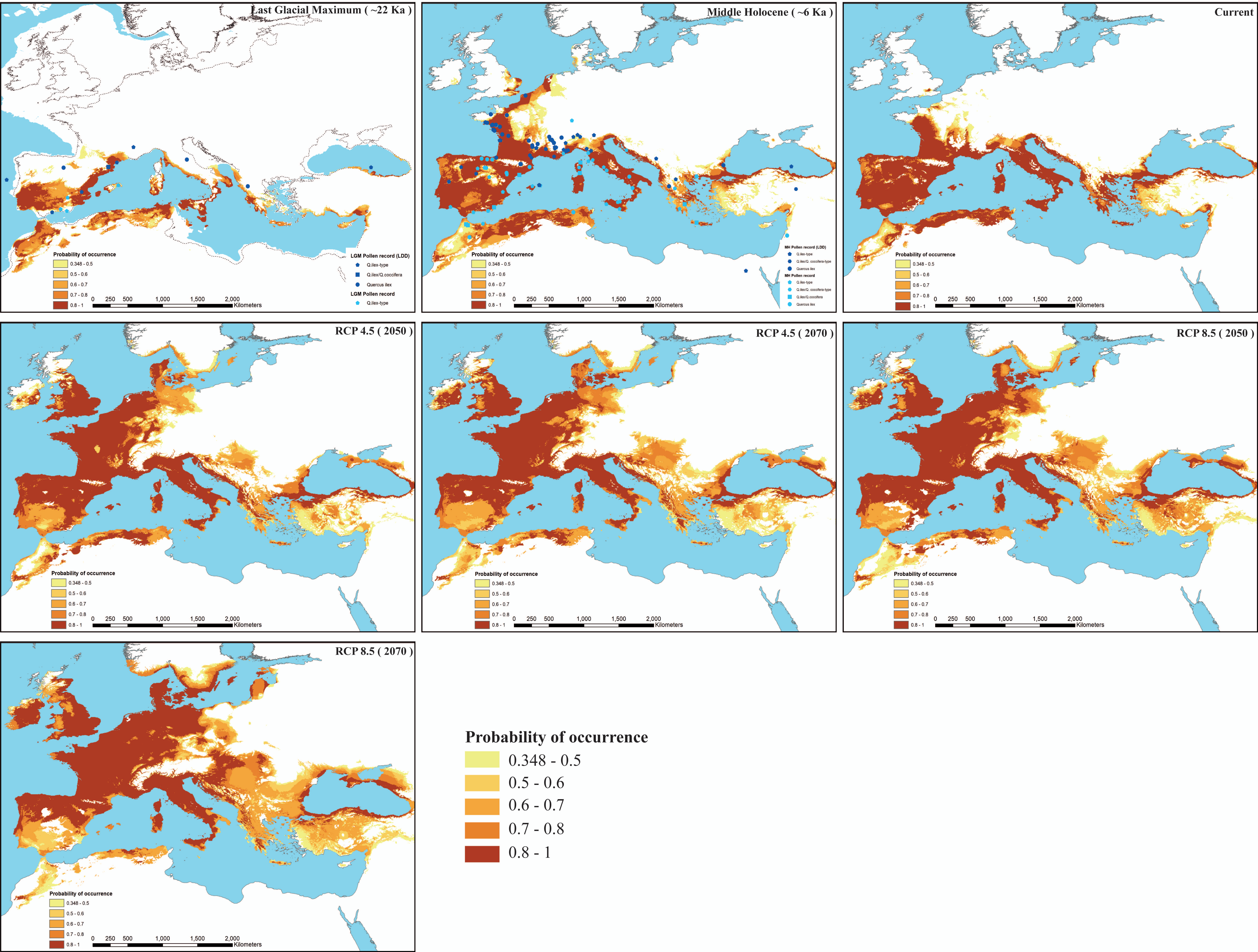

FDA Projections (MIROC-ESM)

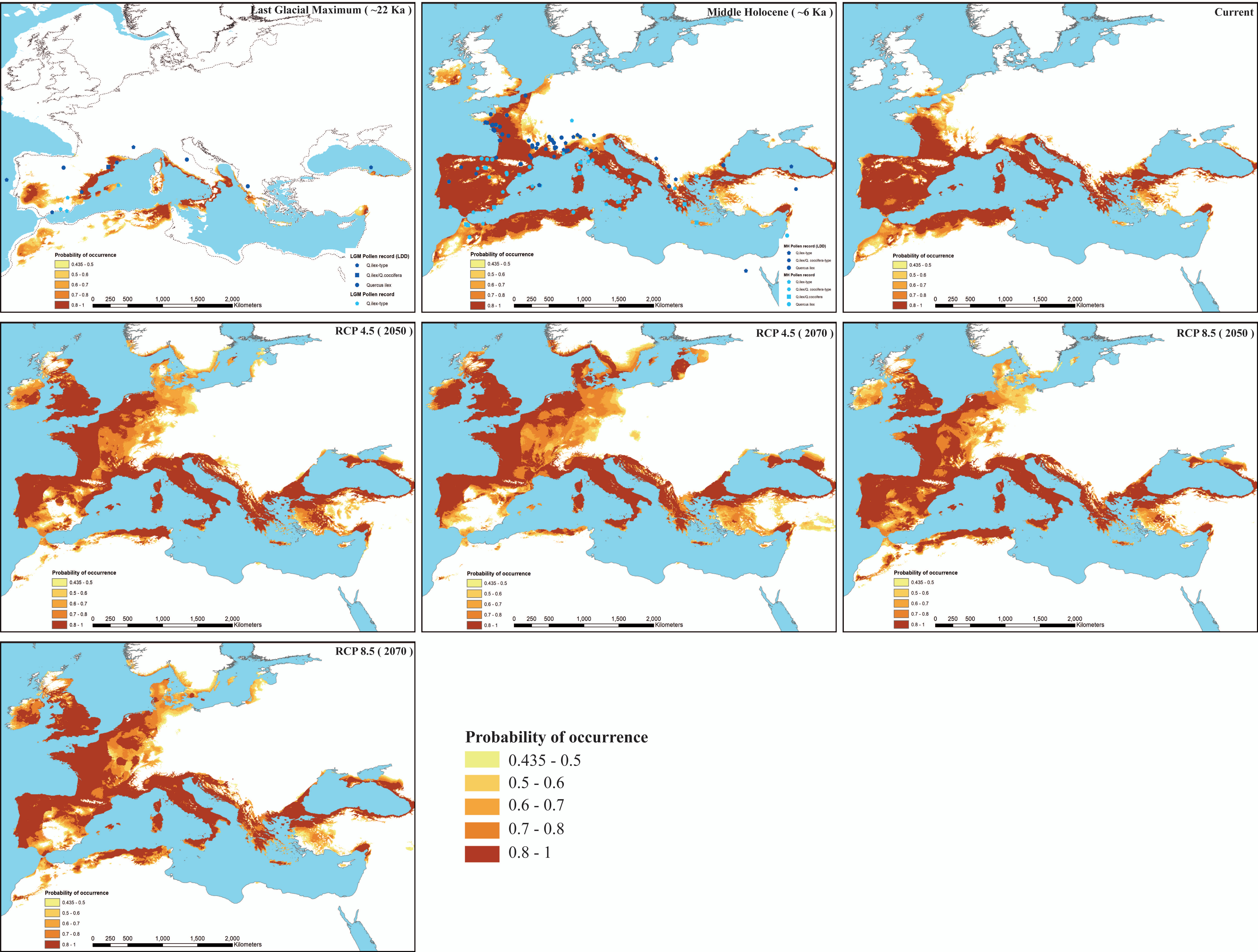

GAM Projections (MIROC-ESM)

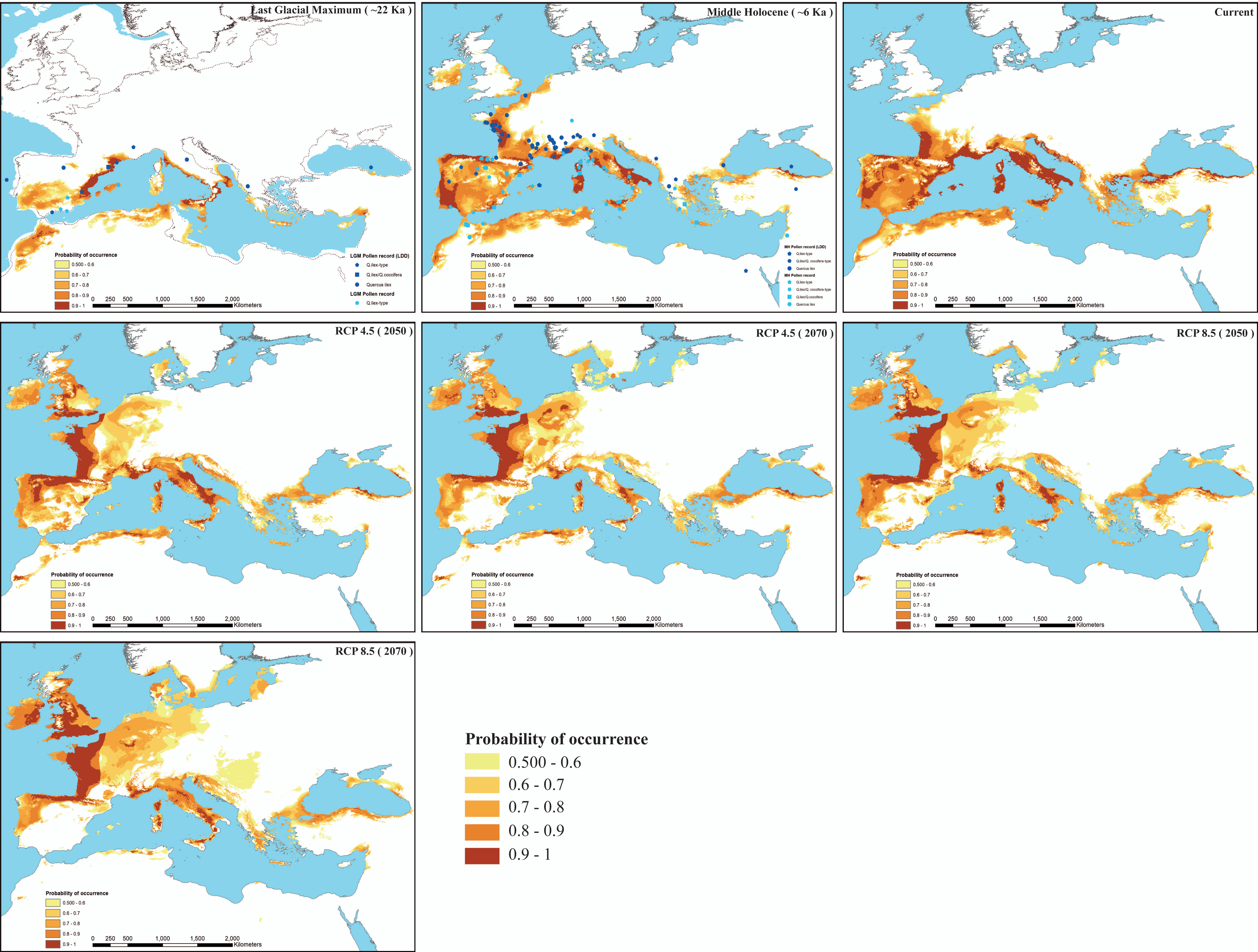

GBM Projections (MIROC-ESM)

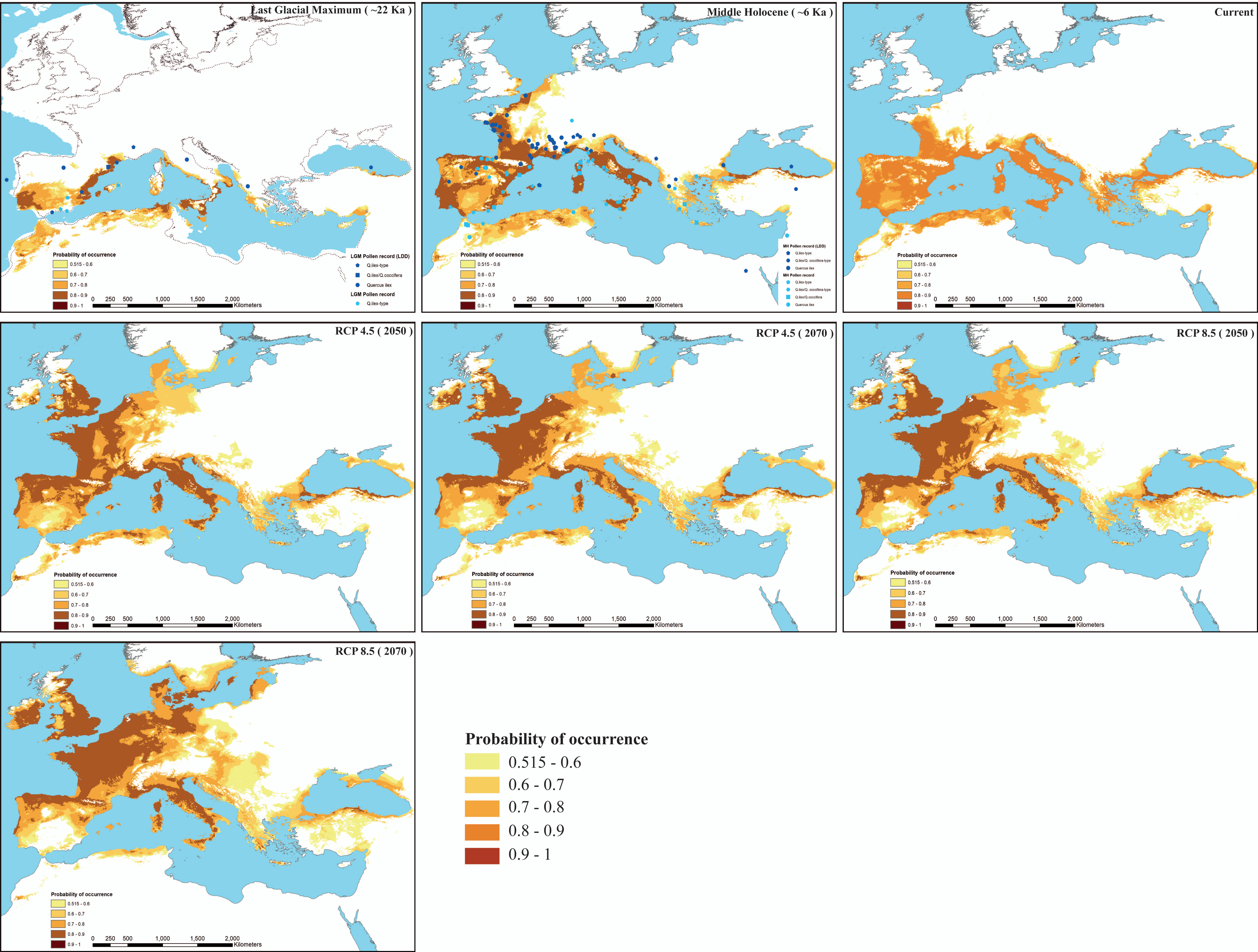

GLM Projections (MIROC-ESM)

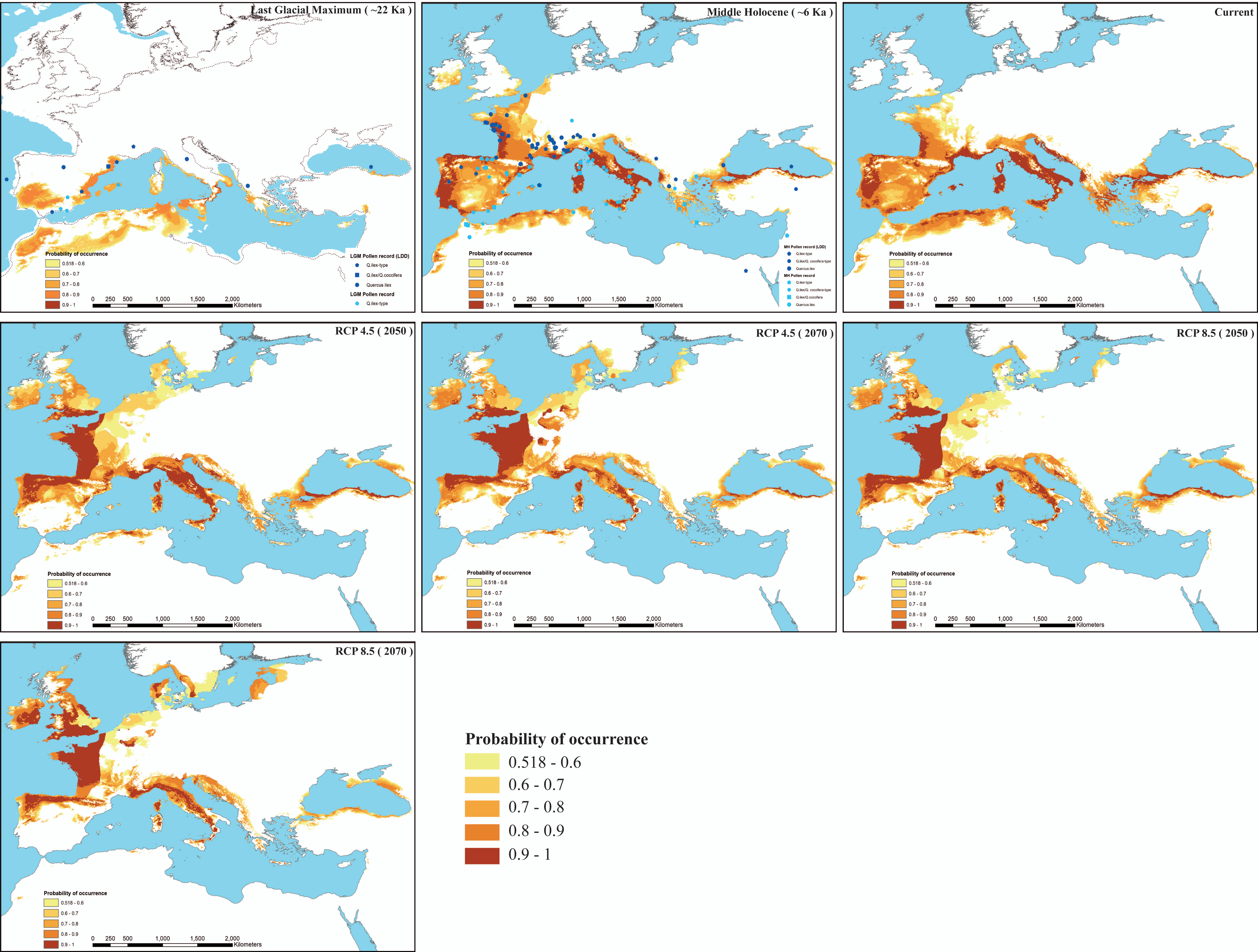

MARS Projections (MIROC-ESM)

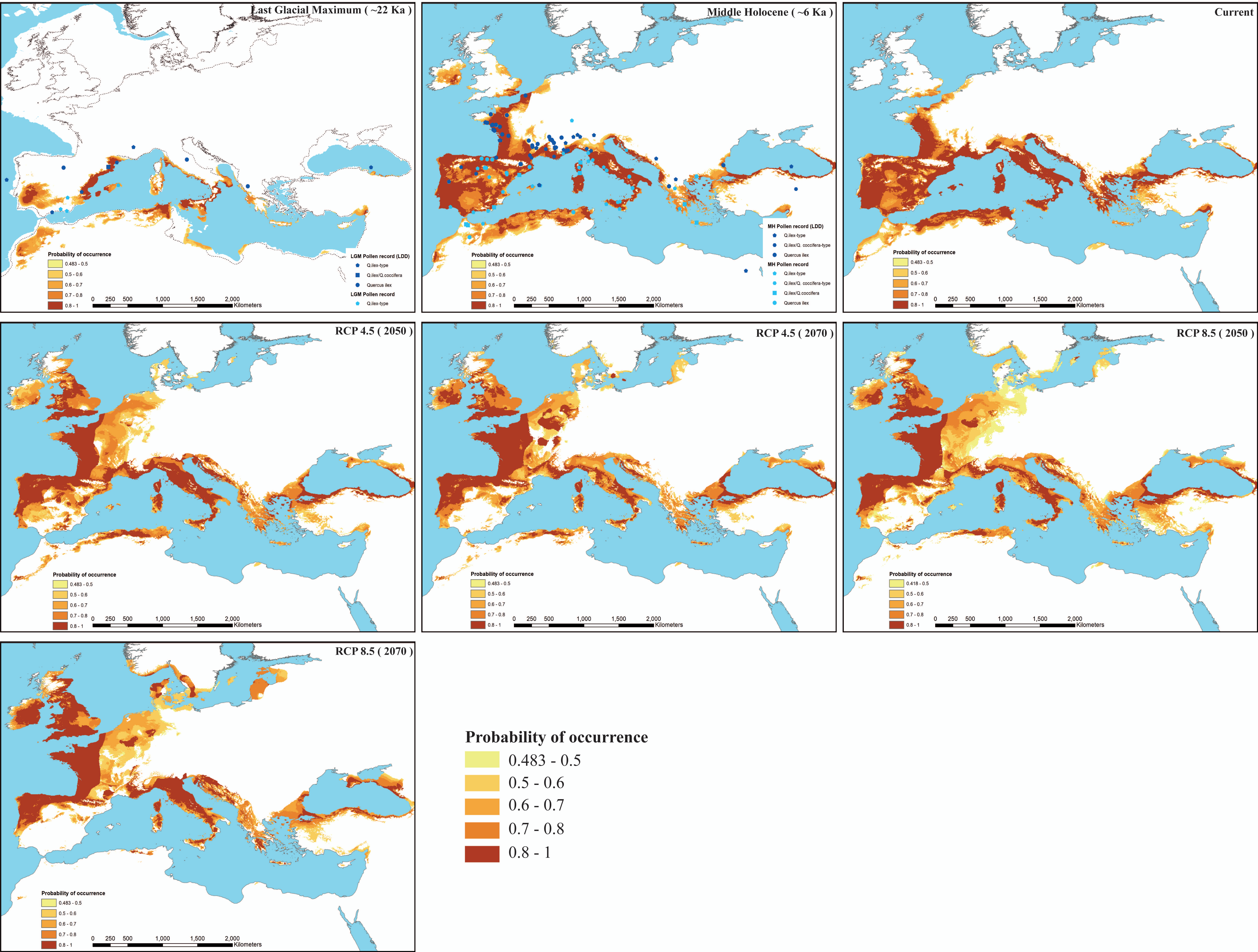

MAXENT Projections (MIROC-ESM)

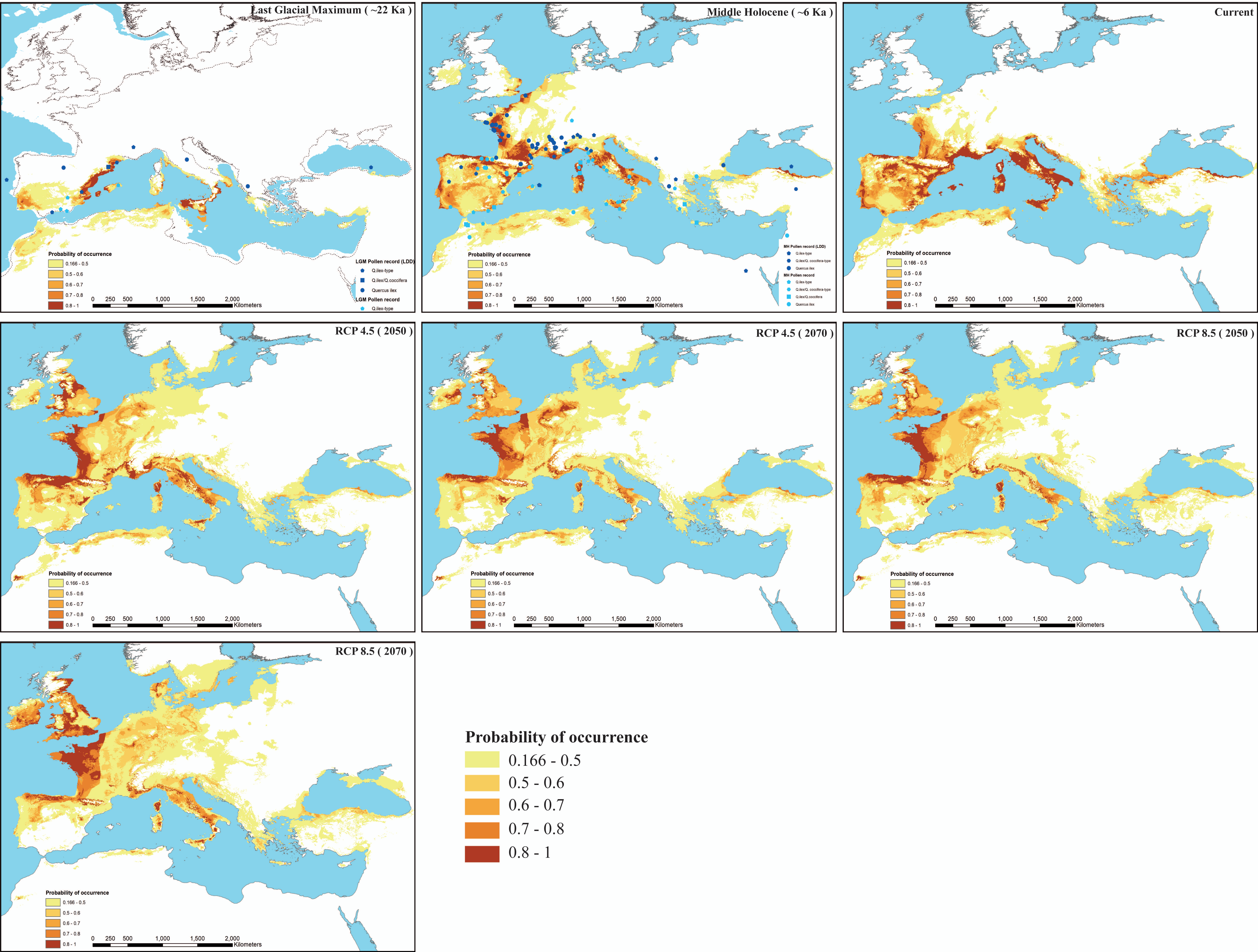

RF Projections (MIROC-ESM)

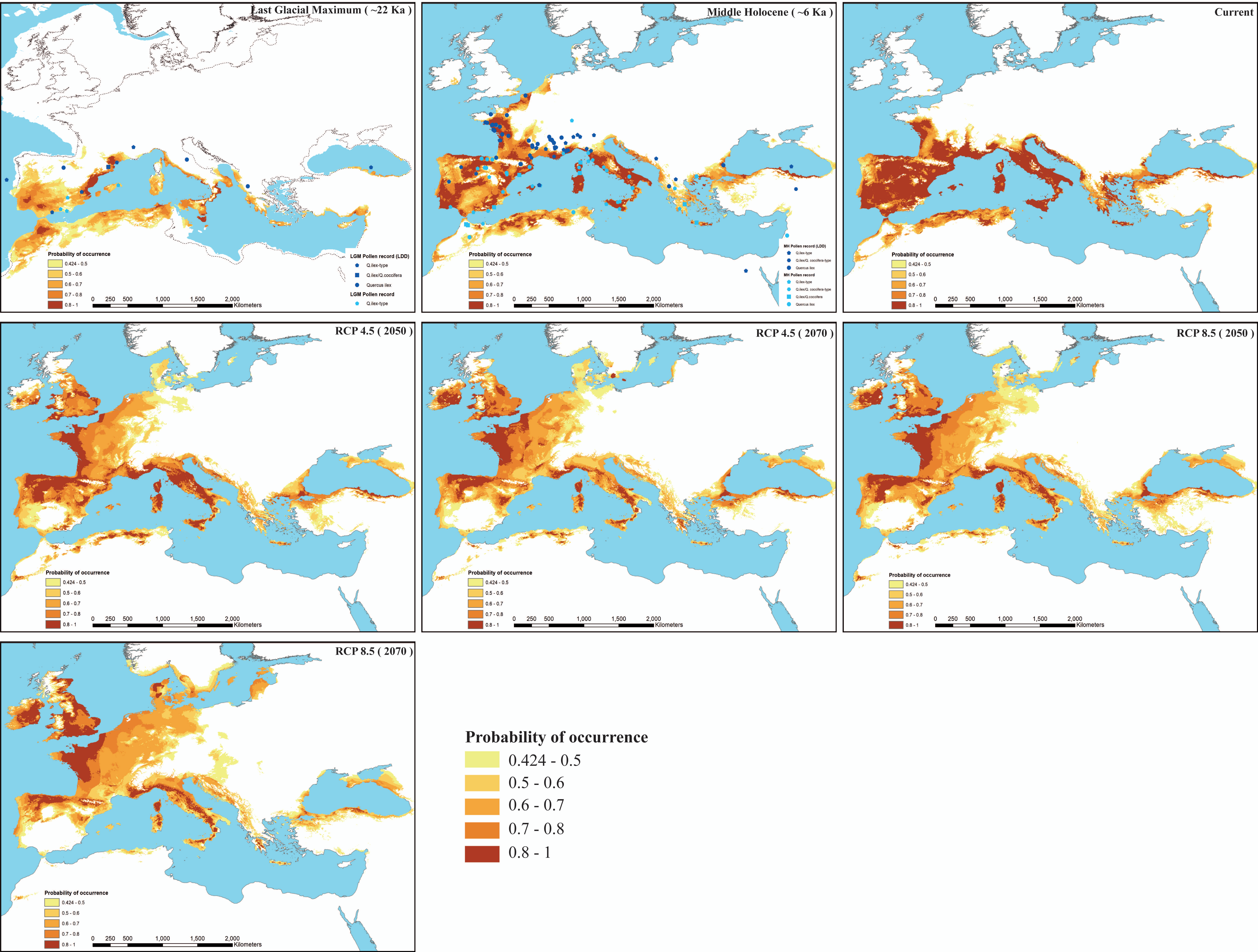

SRE Projections (MIROC-ESM)

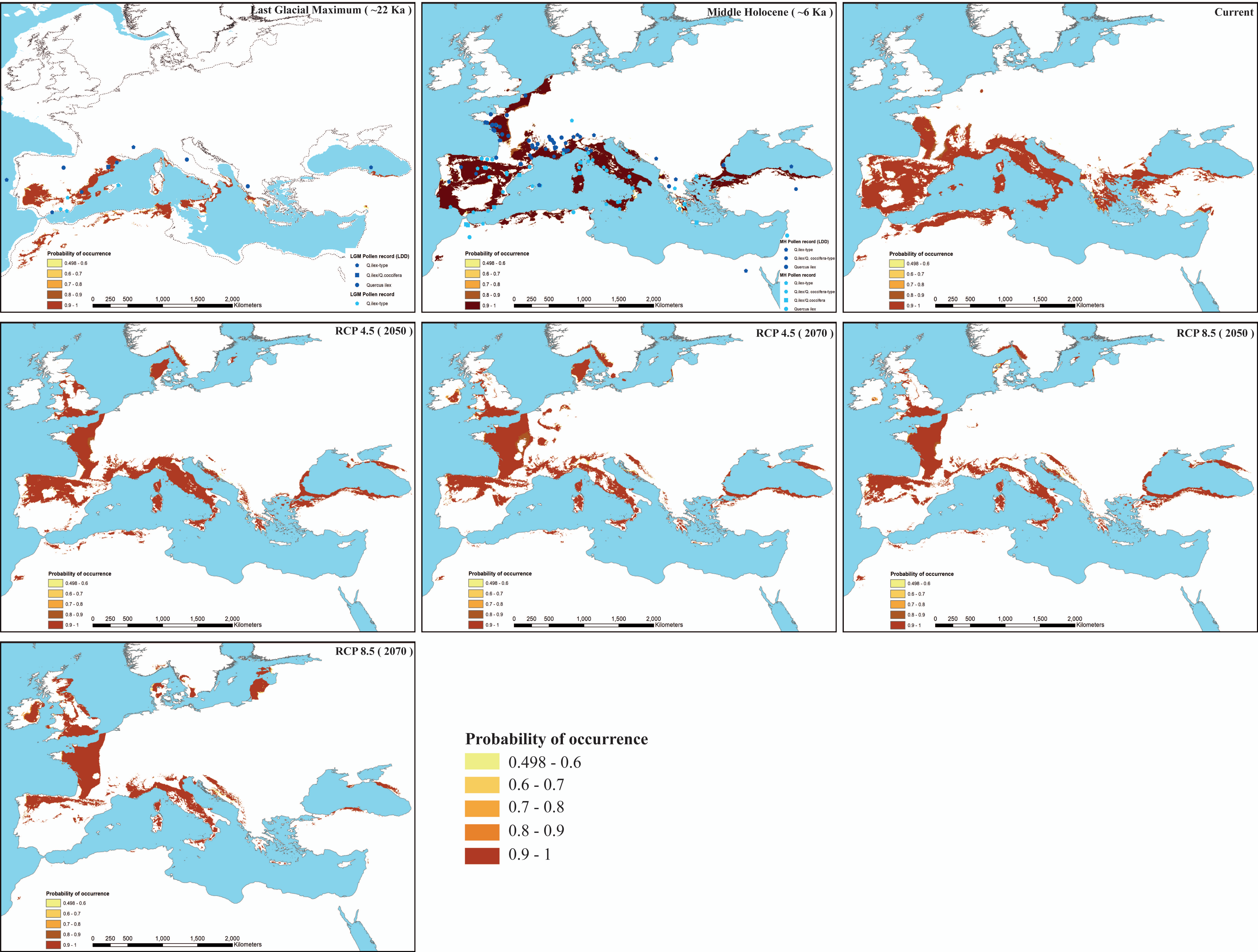

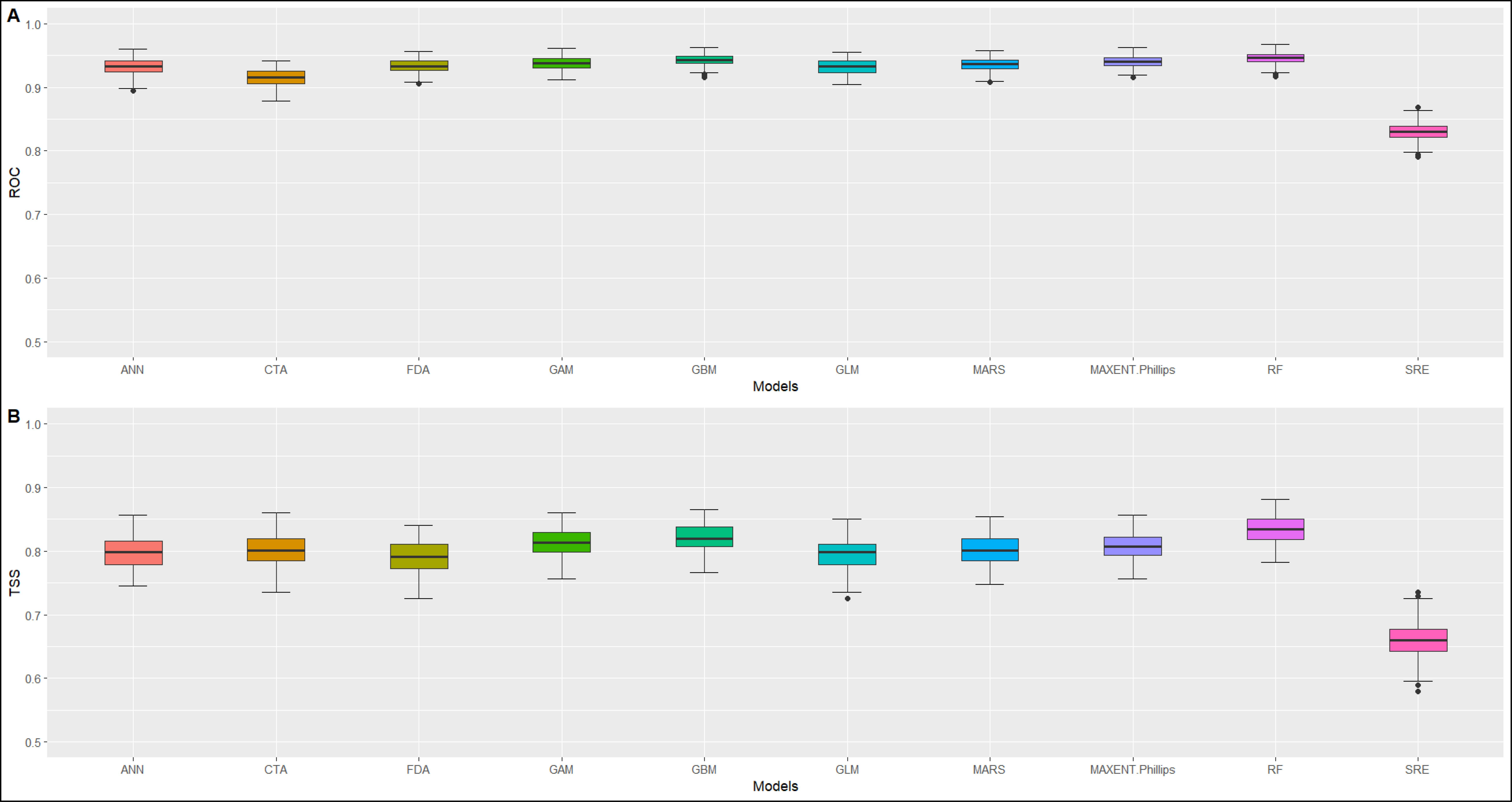
A
ROC
1.0
0.9
0.8
0.7
0.6
0.5
ANN
CTA
FDA
GAM
GBM
GLM
MARS
MAXENT.Phillips
RF
SRE
Models
B
TSS
1.0
0.9
0.8
0.7
0.6
0.5
ANN
CTA
FDA
GAM
GBM
GLM
MARS
MAXENT.Phillips
RF
SRE
Models
