# Supplementary material for: Distribution patterns of Quercus ilex from the last interglacial period to the future by ecological niche modeling
Source: Ecol Evol. 2023 Oct 19;13(10):e10606. doi: 10.1002/ece3.10606 (PMC10585444; doi:10.1002/ece3.10606)
Supplement: Supplementary file 2 — Figure S1–S25. [file ECE3-13-e10606-s004.docx]

| 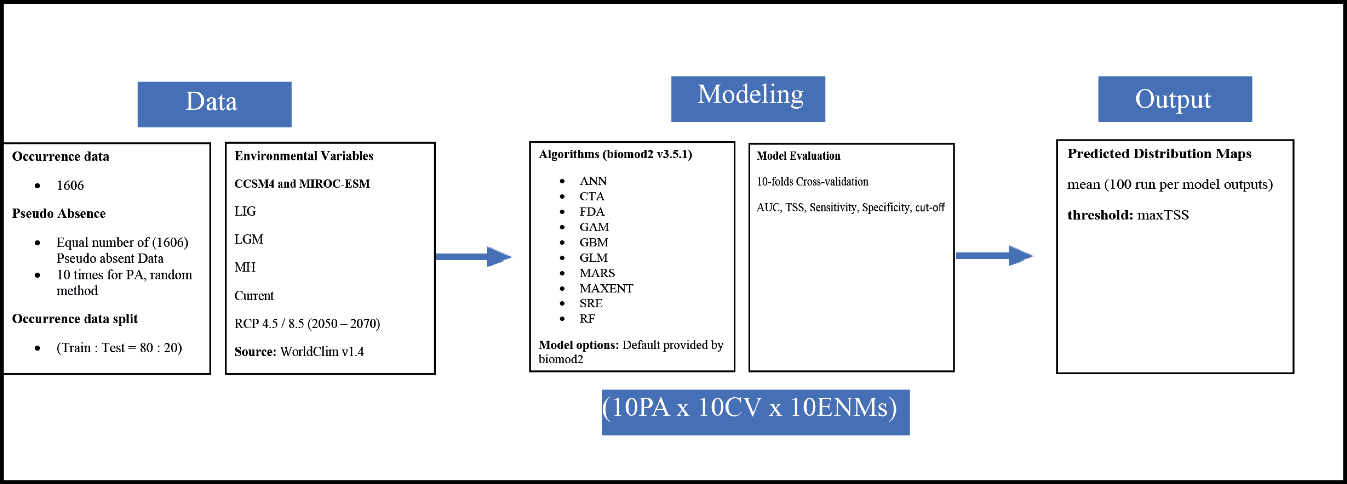  **S1 Figure.** Schematic representation of the model workflow. |
| --- |

| 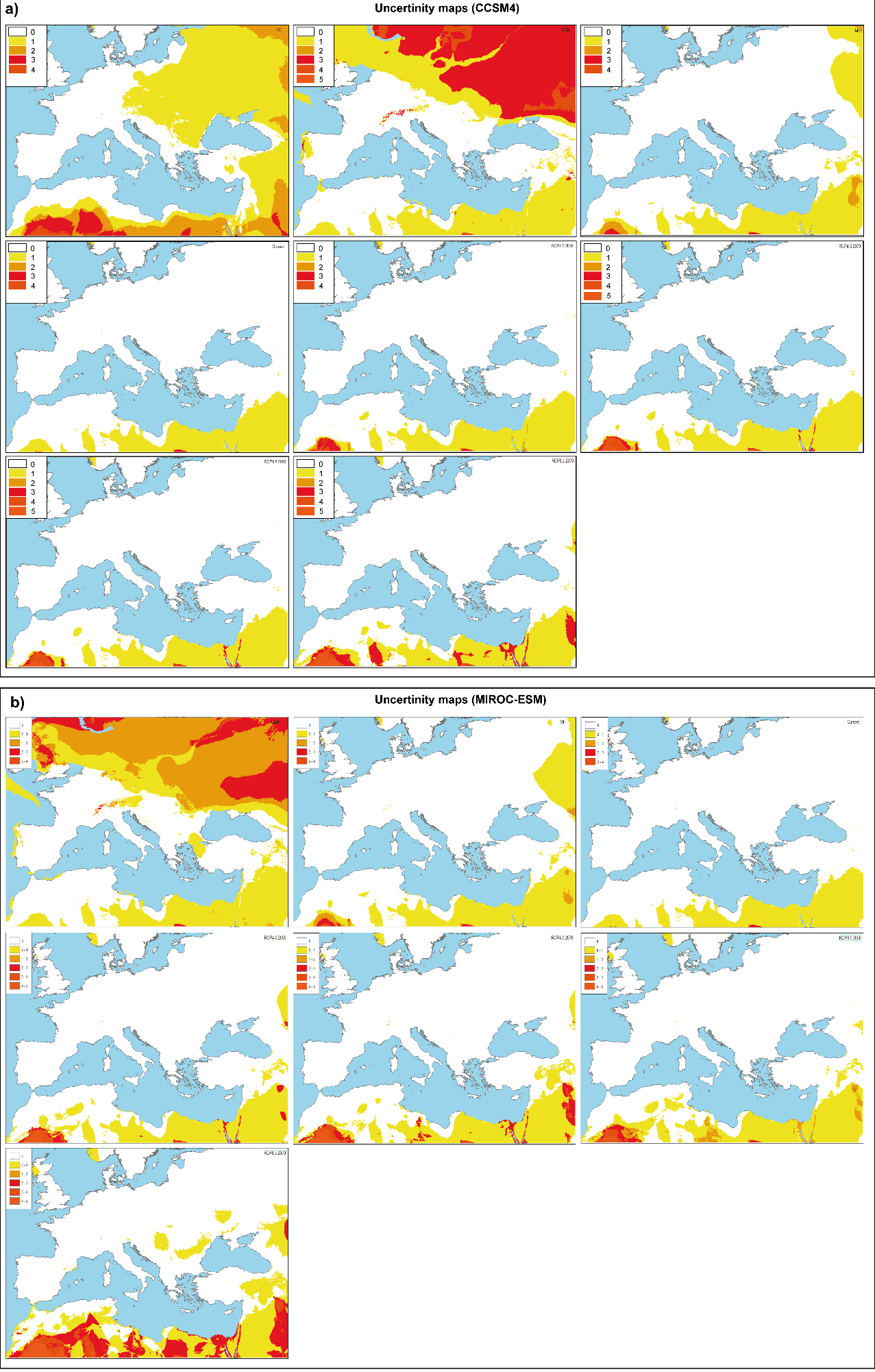  **S2 Figure.** Uncertinity maps of each time periods. |
| --- |

| 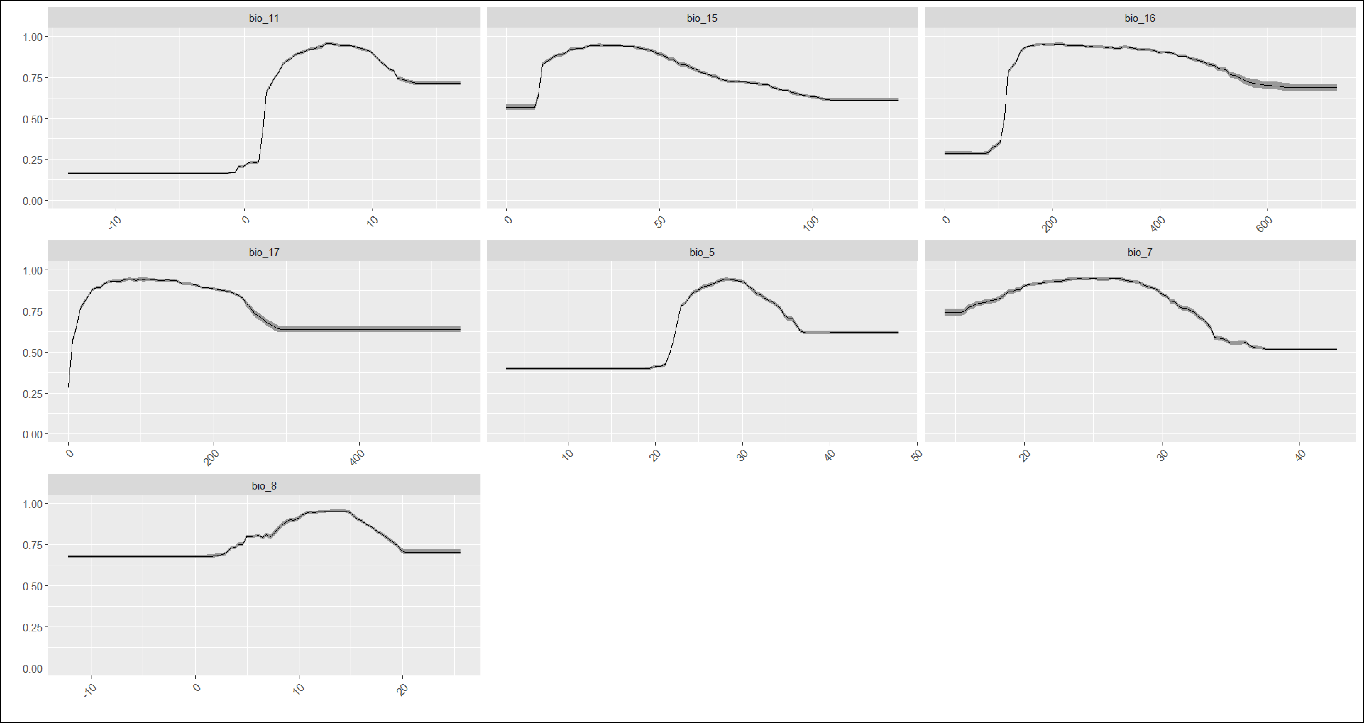  **S3 Figure**. Response curves of RF algorithm showing the tolerance of Q. ilex for the selected environmental variables**.** |
| --- |

| 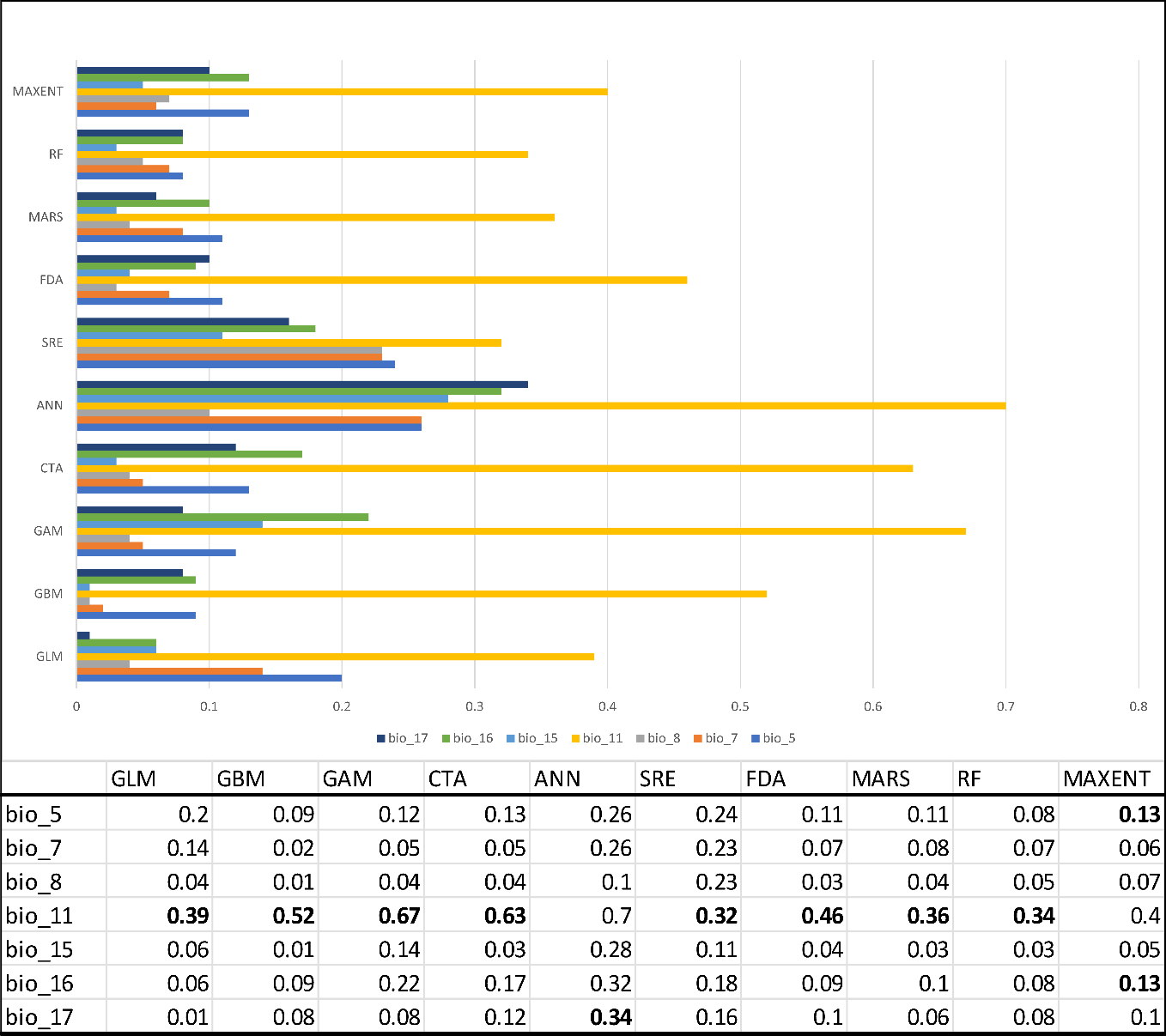  **S4 Figure.** Contributions of the selected environmental variables. |
| --- |


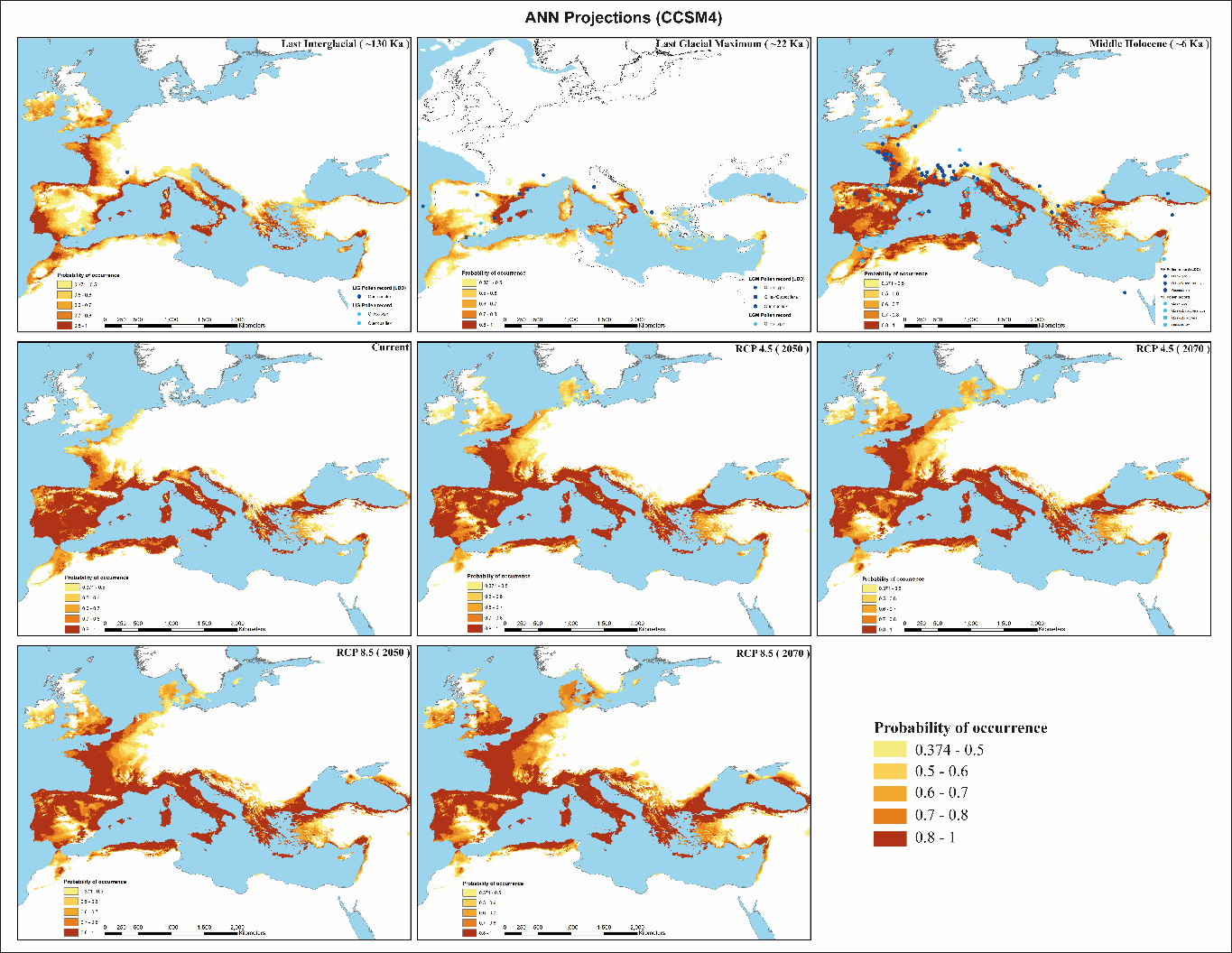


**S5 Figure.** Potential distribution of *Q. ilex* predicted for the LIG, LGM, MH, Current, moderate (RCP 4.5) 2050 and 2070 and pessimistic (RCP 8.5) 2050 and 2070 with ANN algorithm and using CCSM4 climate model

| 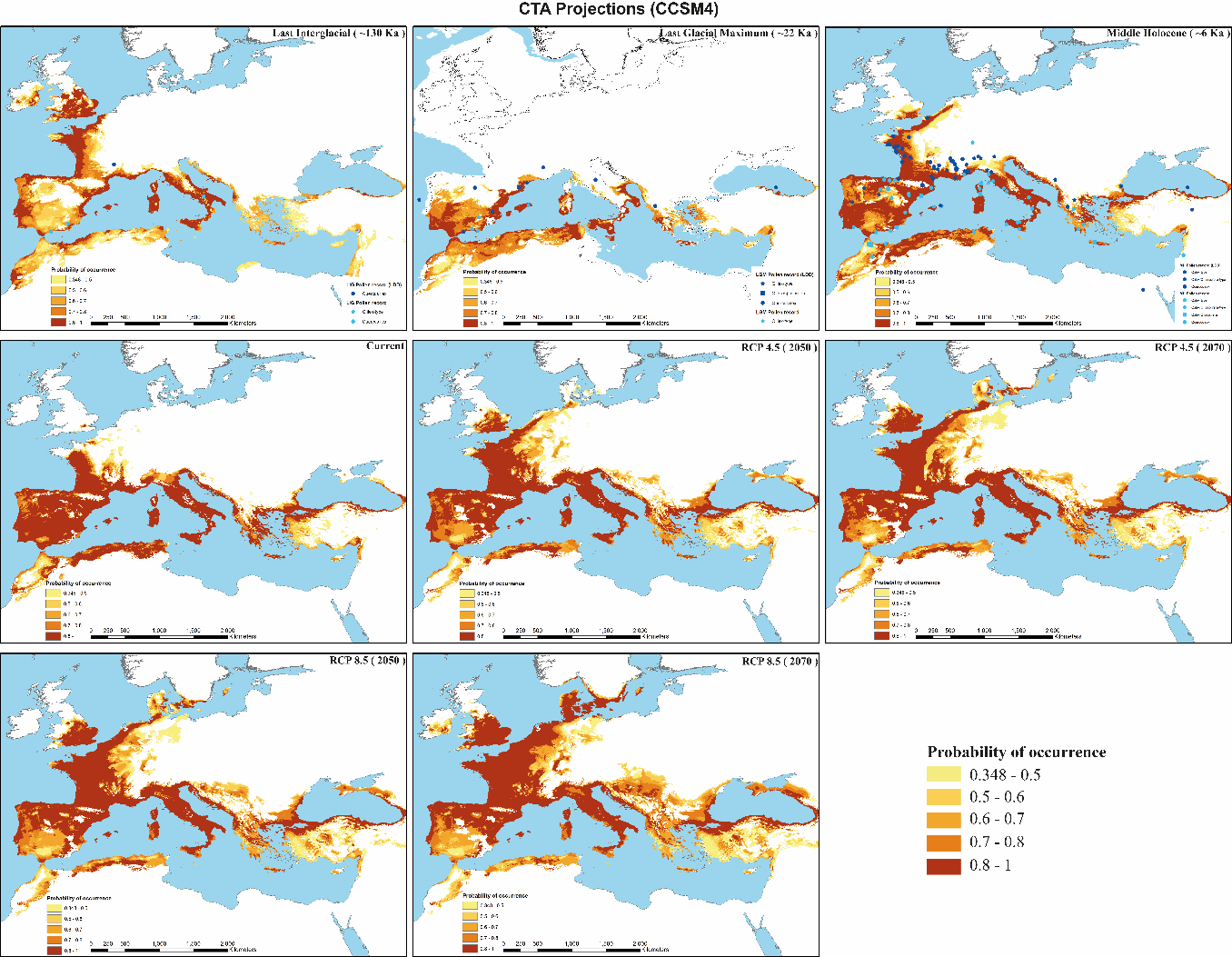  **S6 Figure.** Potential distribution of *Q. ilex* predicted for the LIG, LGM, MH, Current, moderate (RCP 4.5) 2050 and 2070 and pessimistic (RCP 8.5) 2050 and 2070 with CTA algorithm and using CCSM4 climate model. |
| --- |

| 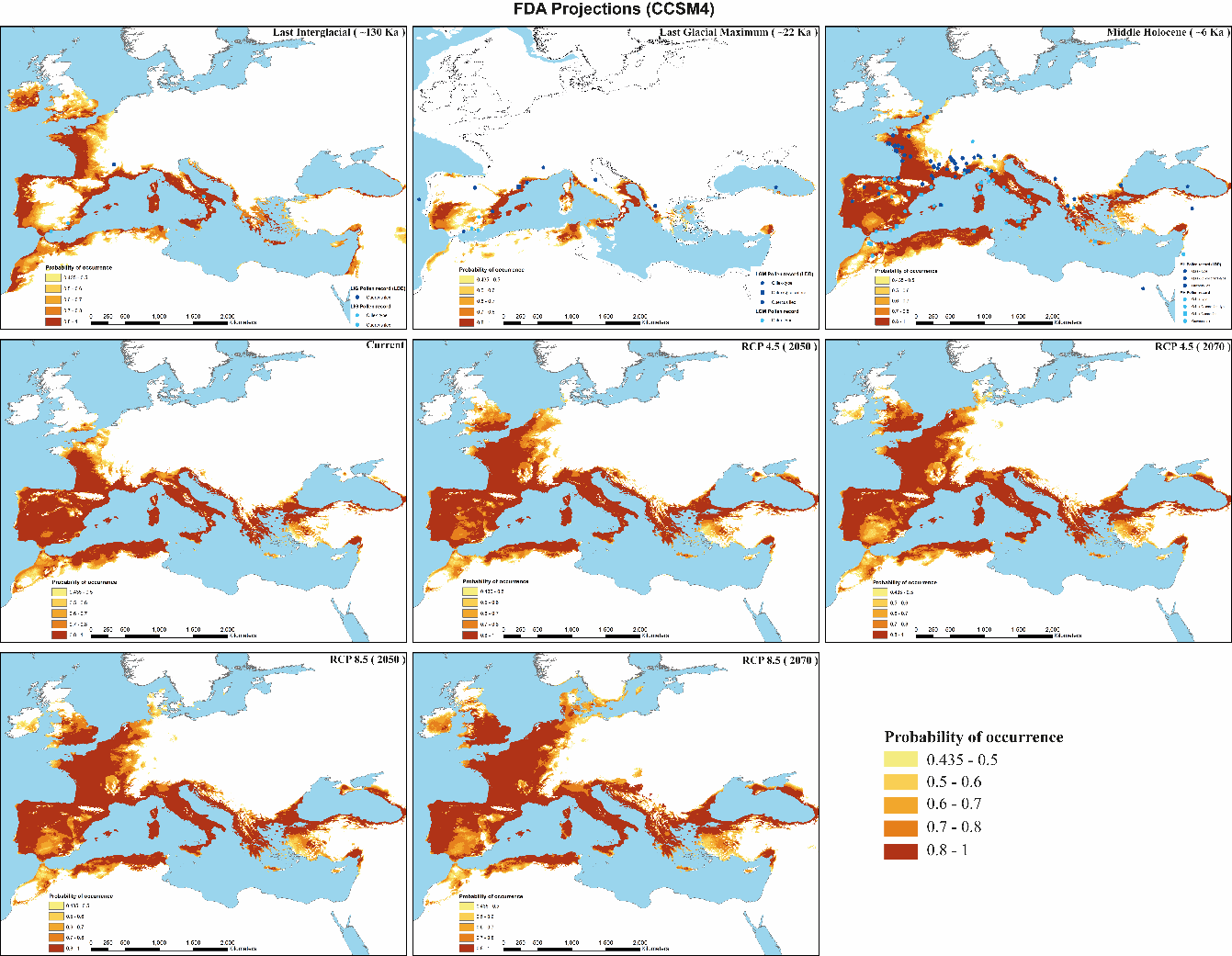  **S7 Figure.** Potential distribution of *Q. ilex* predicted for the LIG, LGM, MH, Current, moderate (RCP 4.5) 2050 and 2070 and pessimistic (RCP 8.5) 2050 and 2070 with FDA algorithm and using CCSM4 climate model. |
| --- |

| 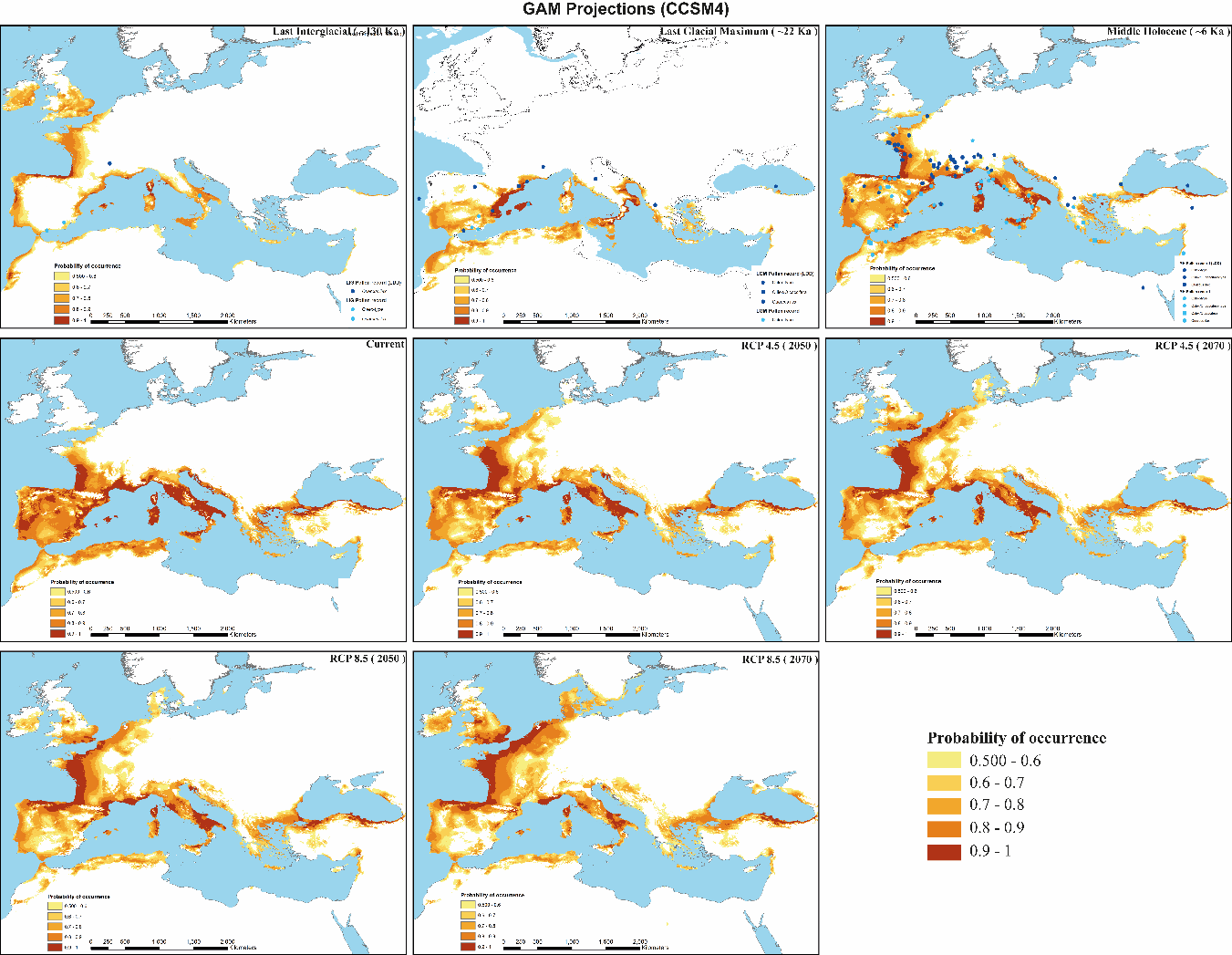  **S8 Figure.** Potential distribution of *Q. ilex* predicted for the LIG, LGM, MH, Current, moderate (RCP 4.5) 2050 and 2070 and pessimistic (RCP 8.5) 2050 and 2070 with GAM algorithm and using CCSM4 climate model. |
| --- |

| 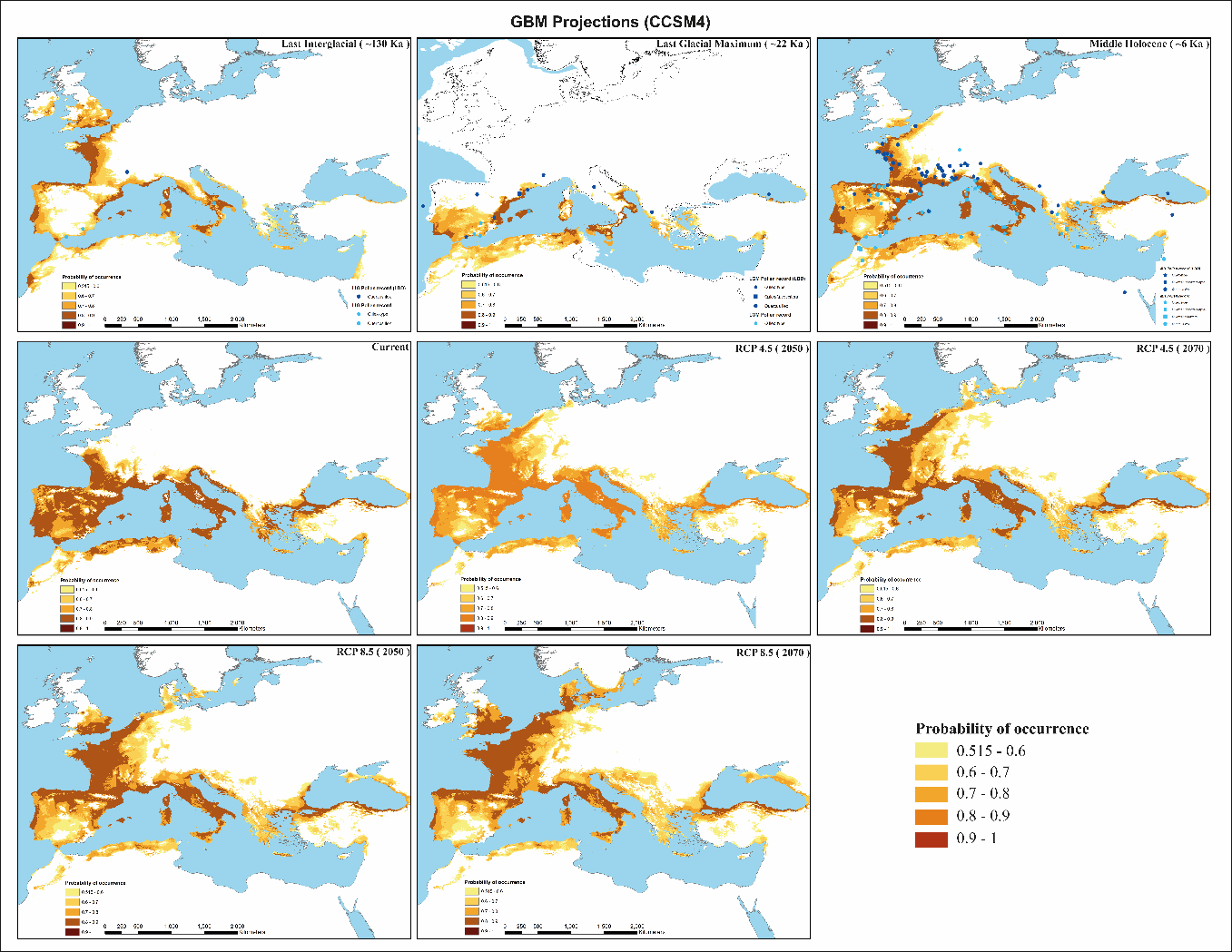  **S9 Figure.** Potential distribution of *Q. ilex* predicted for the LIG, LGM, MH, Current, moderate (RCP 4.5) 2050 and 2070 and pessimistic (RCP 8.5) 2050 and 2070 with GAM algorithm and using CCSM4 climate model. |
| --- |

| 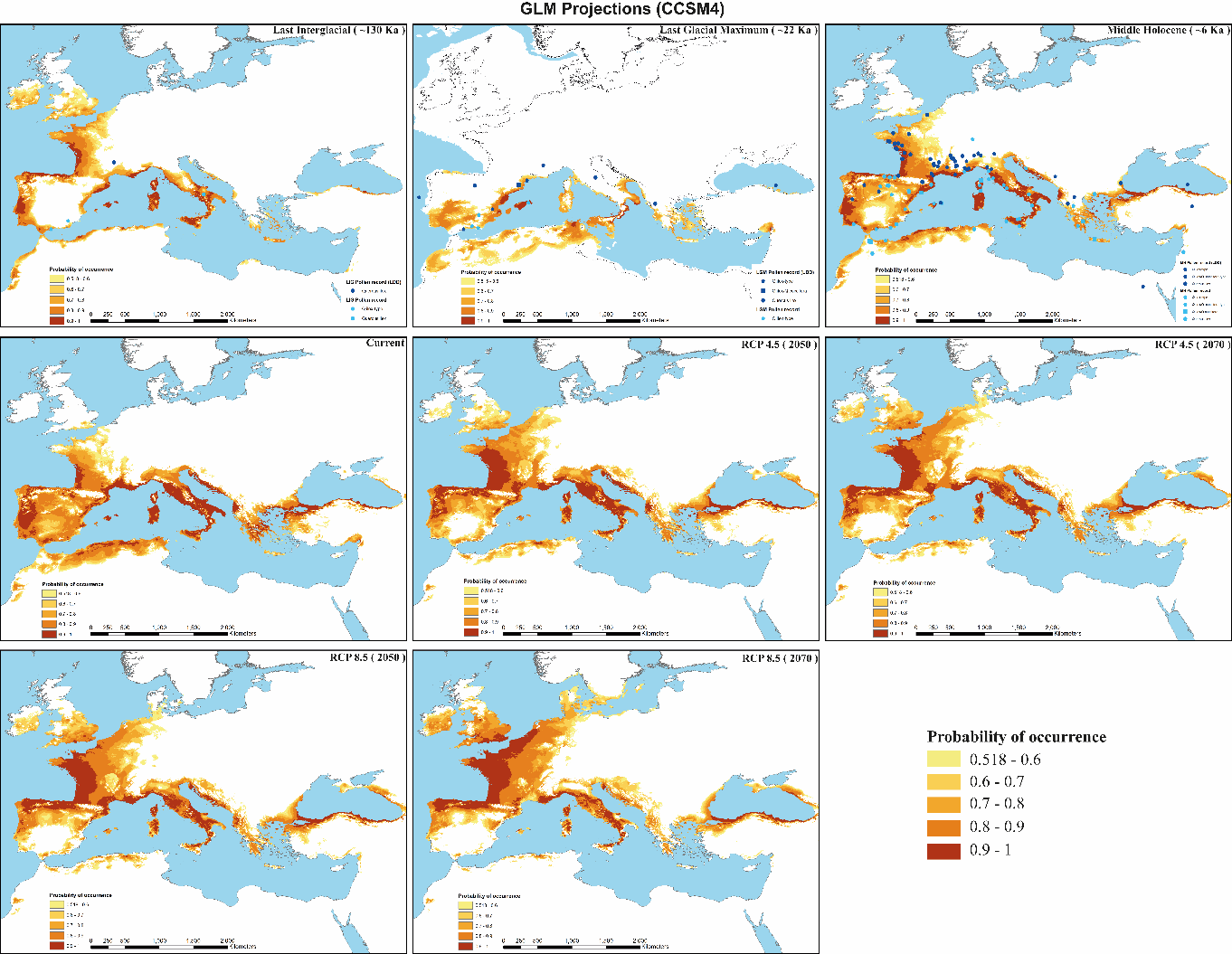  **S10 Figure.** Potential distribution of *Q. ilex* predicted for the LIG, LGM, MH, Current, moderate (RCP 4.5) 2050 and 2070 and pessimistic (RCP 8.5) 2050 and 2070 with GLM algorithm and using CCSM4 climate model. |
| --- |

| 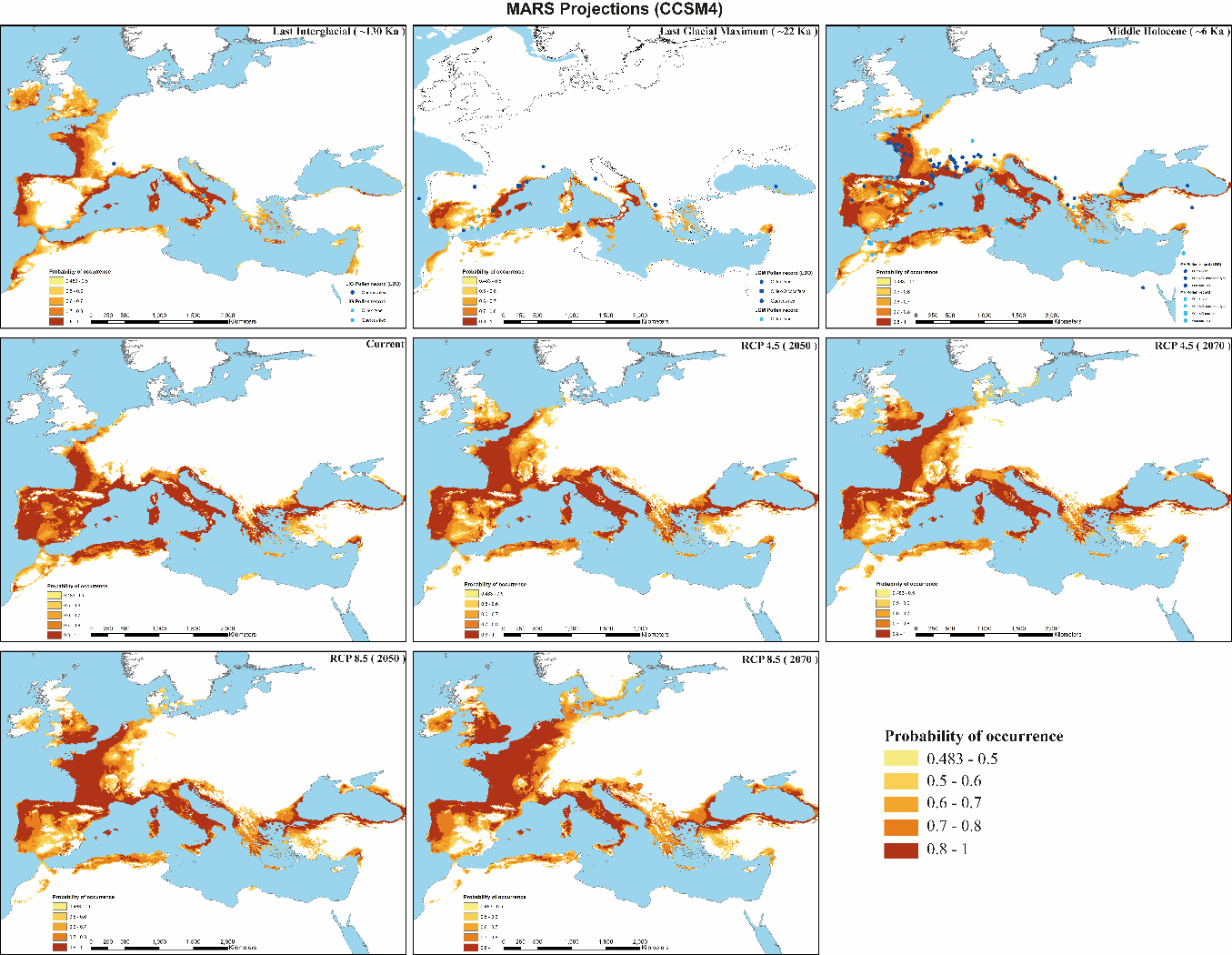  **S11 Figure.** Potential distribution of *Q. ilex* predicted for the LIG, LGM, MH, Current, moderate (RCP 4.5) 2050 and 2070 and pessimistic (RCP 8.5) 2050 and 2070 with MARS algorithm and using CCSM4 climate model. |
| --- |

| 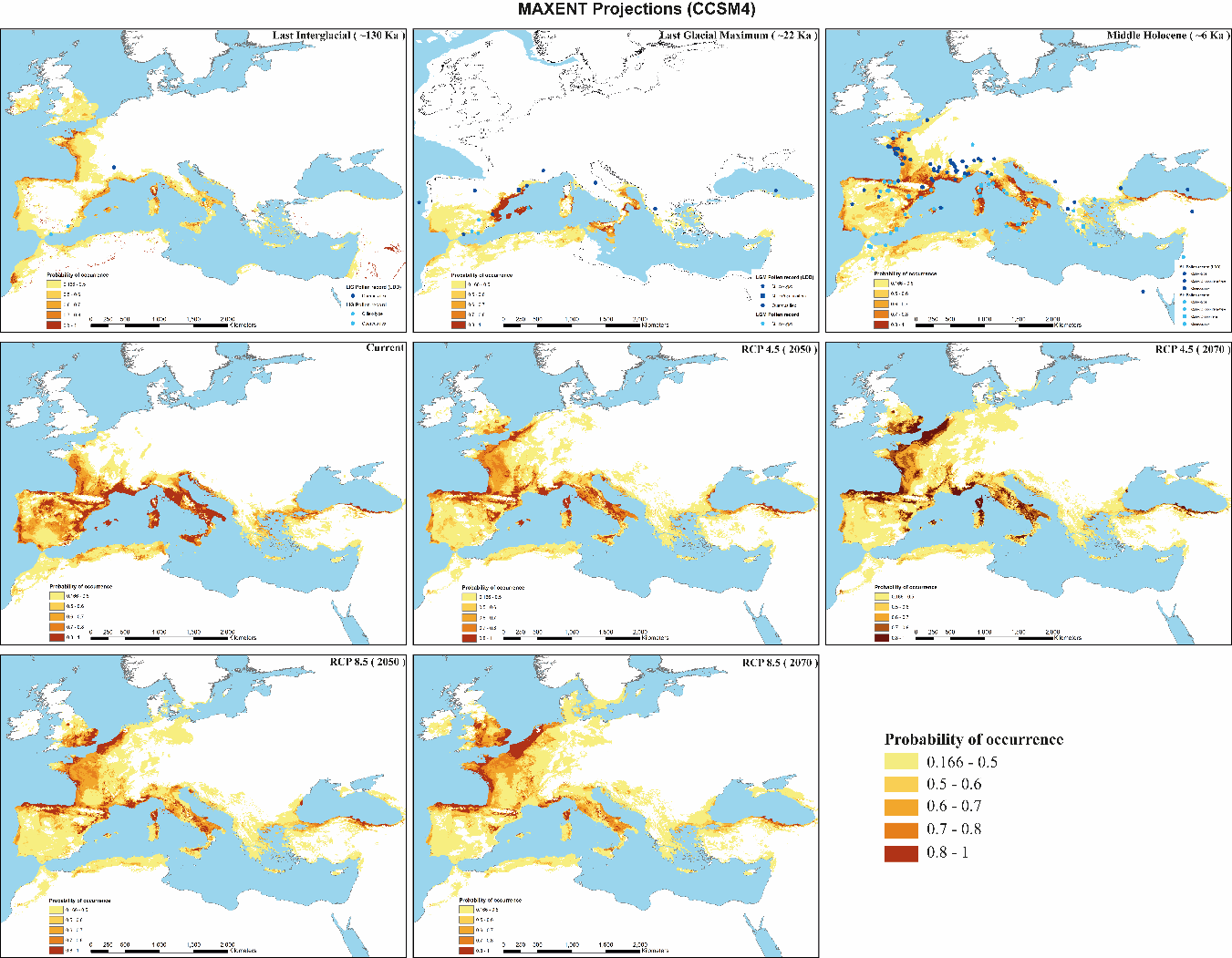  **S12 Figure.** Potential distribution of *Q. ilex* predicted for the LIG, LGM, MH, Current, moderate (RCP 4.5) 2050 and 2070 and pessimistic (RCP 8.5) 2050 and 2070 with MAXENT algorithm and using CCSM4 climate model. |
| --- |

| 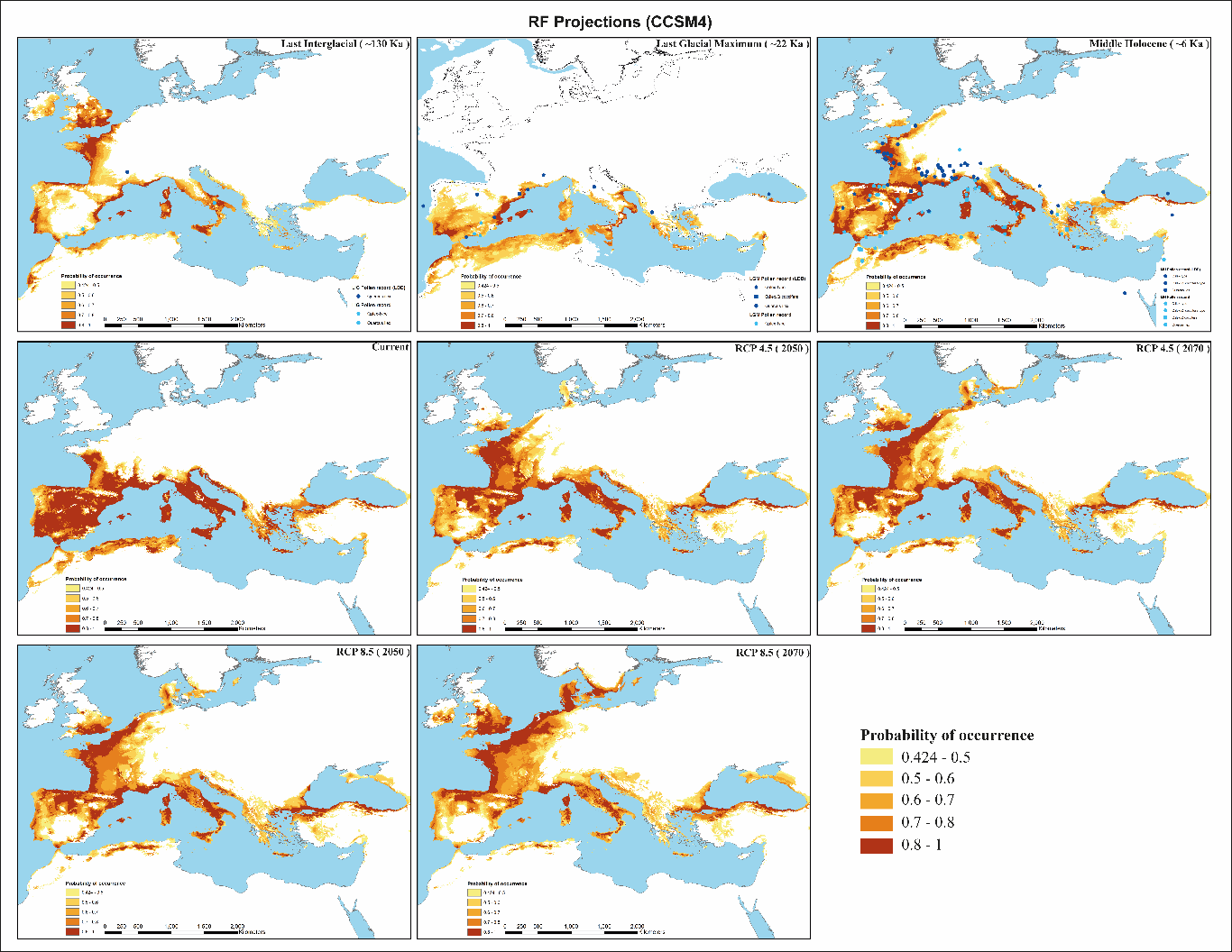  **S13 Figure.** Potential distribution of *Q. ilex* predicted for the LIG, LGM, MH, Current, moderate (RCP 4.5) 2050 and 2070 and pessimistic (RCP 8.5) 2050 and 2070 with RF algorithm and using CCSM4 climate model. |
| --- |

| 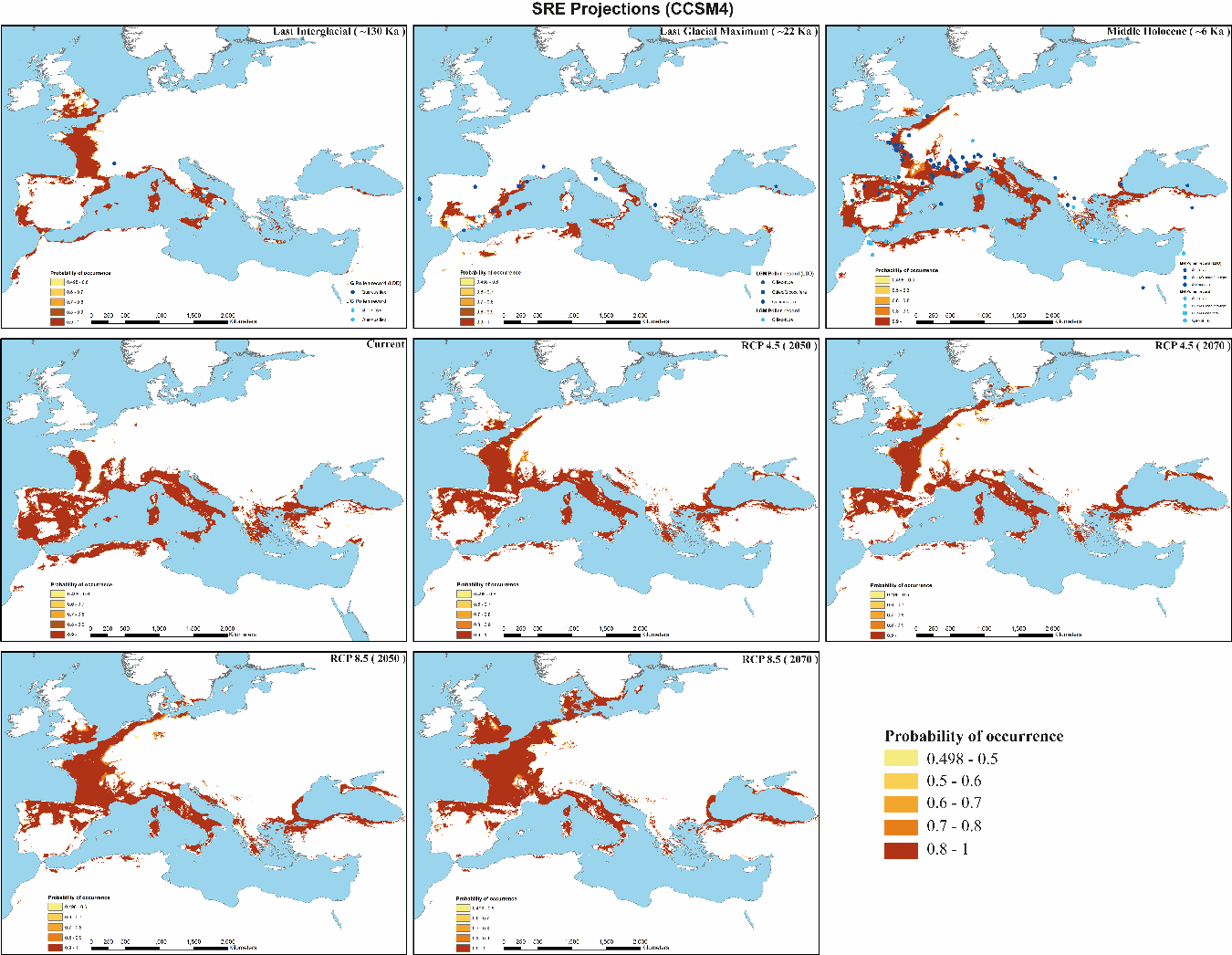  **S14 Figure.** Potential distribution of *Q. ilex* predicted for the LIG, LGM, MH, Current, moderate (RCP 4.5) 2050 and 2070 and pessimistic (RCP 8.5) 2050 and 2070 with SRE algorithm and using CCSM4 climate model. |
| --- |

| 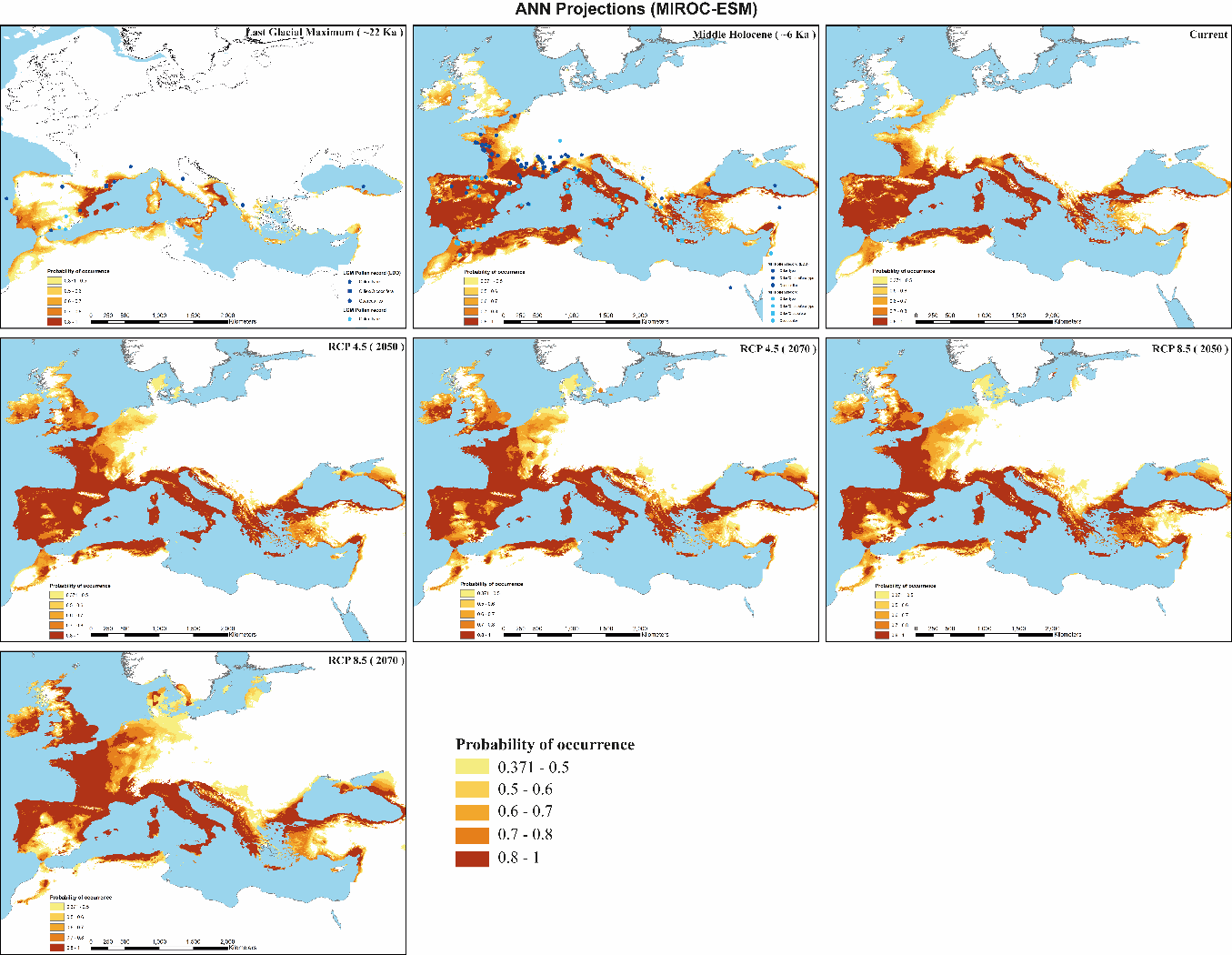  **S15 Figure.** Potential distribution of *Q. ilex* predicted for the LIG, LGM, MH, Current, moderate (RCP 4.5) 2050 and 2070 and pessimistic (RCP 8.5) 2050 and 2070 with ANN algorithm and using MIROC-ESM climate model. |
| --- |

| 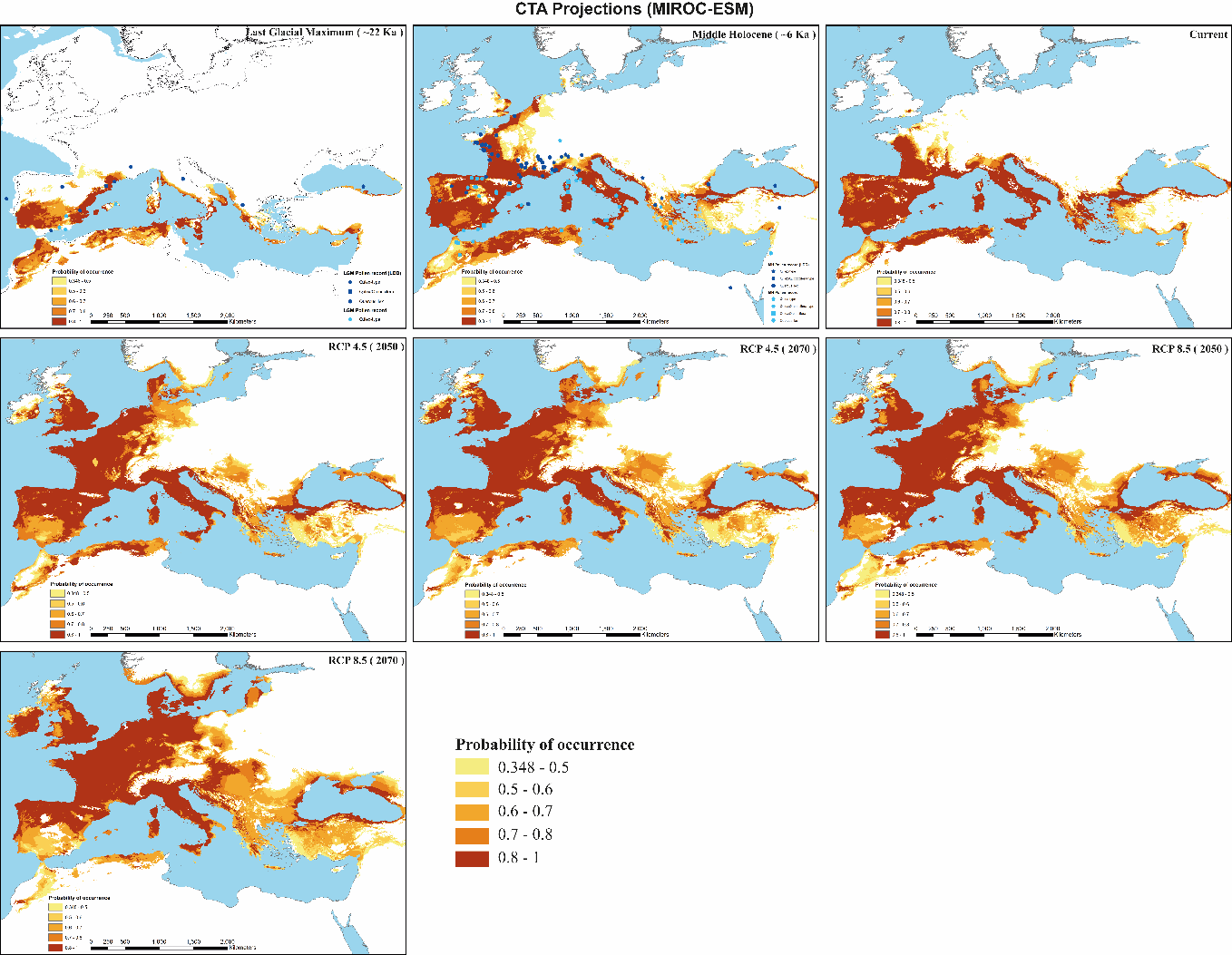  **S16 Figure.** Potential distribution of *Q. ilex* predicted for the LIG, LGM, MH, Current, moderate (RCP 4.5) 2050 and 2070 and pessimistic (RCP 8.5) 2050 and 2070 with CTA algorithm and using MIROC-ESM climate model. |
| --- |

| 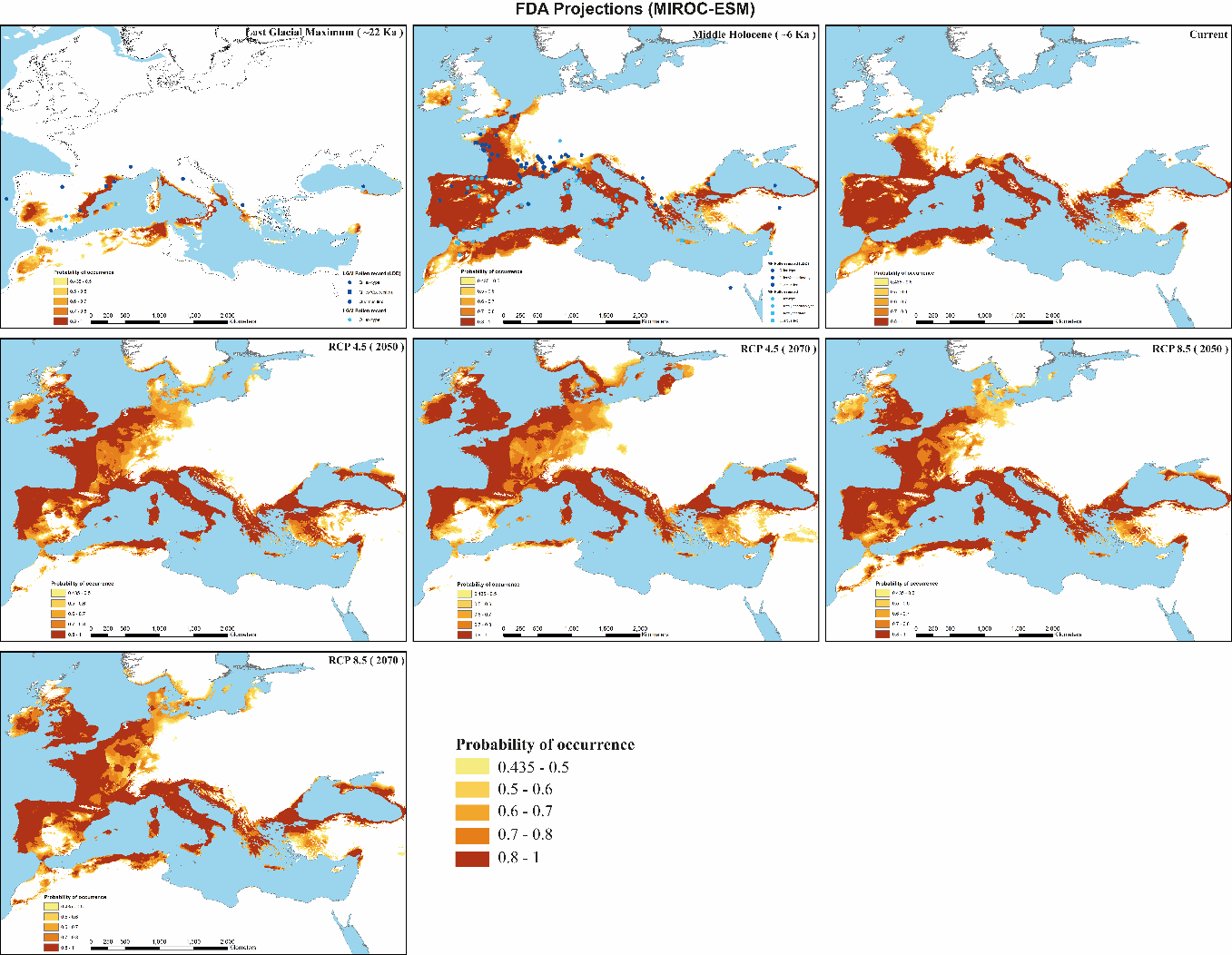  **S17 Figure.** Potential distribution of *Q. ilex* predicted for the LIG, LGM, MH, Current, moderate (RCP 4.5) 2050 and 2070 and pessimistic (RCP 8.5) 2050 and 2070 with FDA algorithm and using MIROC-ESM climate model. |
| --- |

| 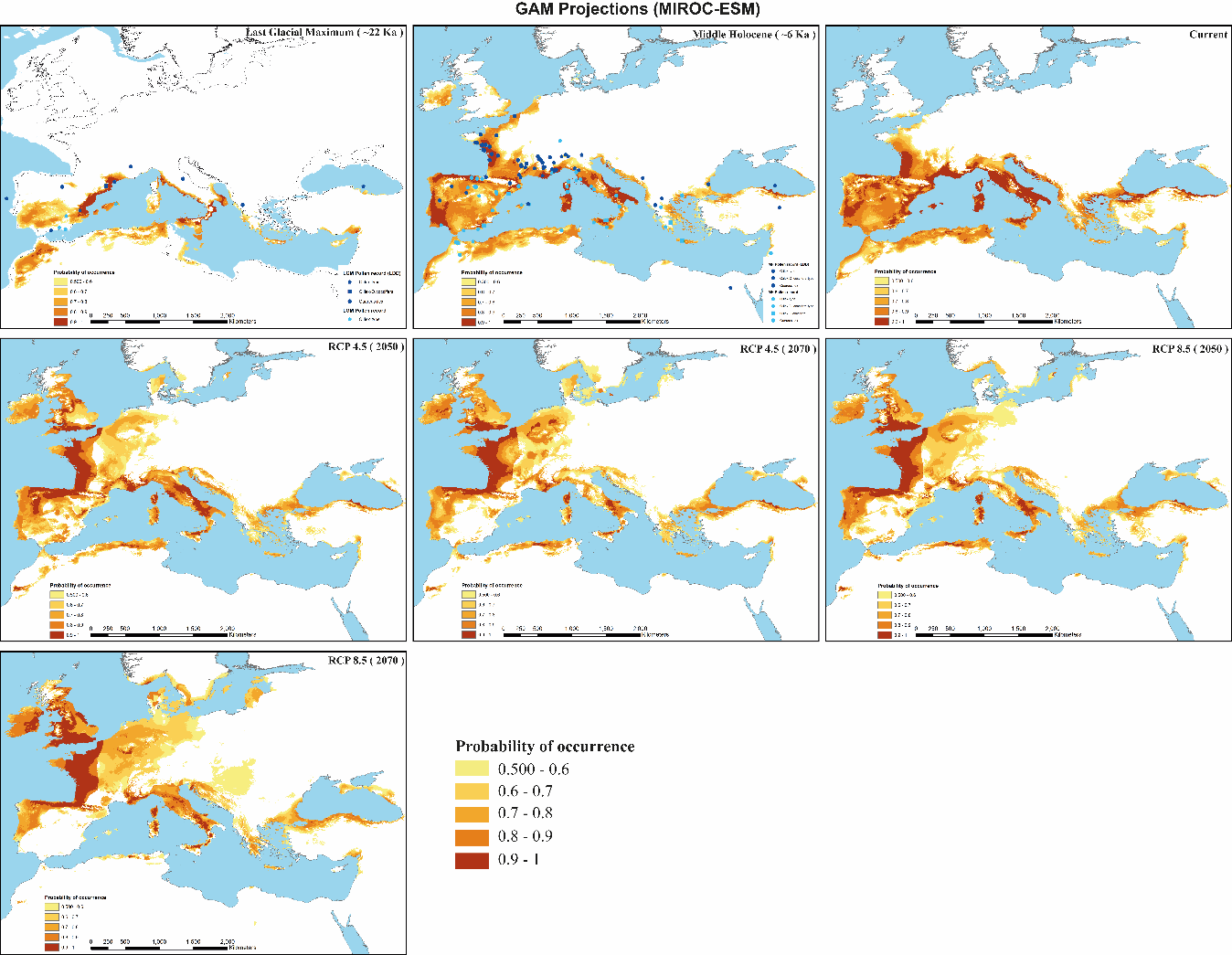  **S18 Figure.** Potential distribution of *Q. ilex* predicted for the LIG, LGM, MH, Current, moderate (RCP 4.5) 2050 and 2070 and pessimistic (RCP 8.5) 2050 and 2070 with GAM algorithm and using MIROC-ESM climate model. |
| --- |

| 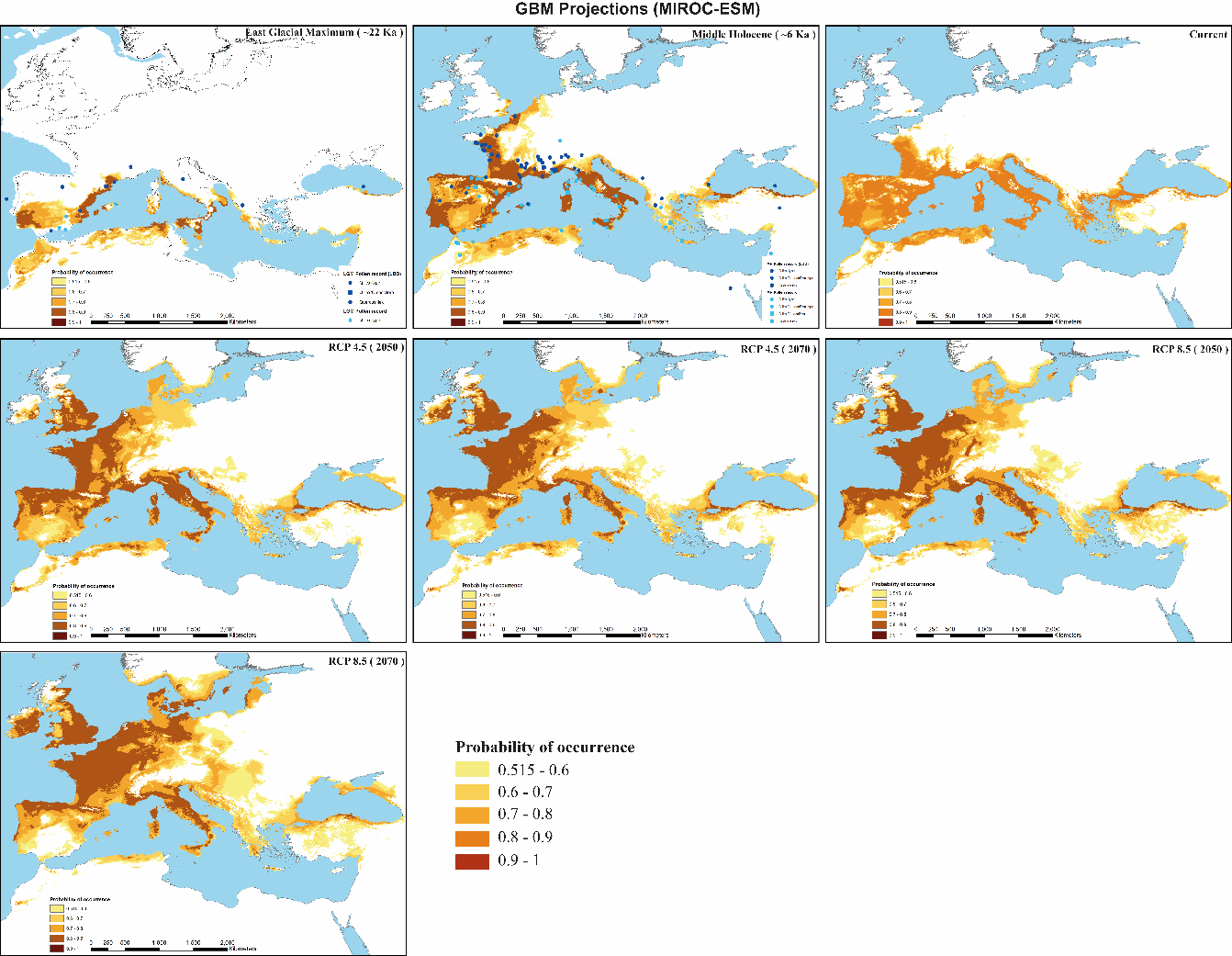  **S19 Figure.** Potential distribution of *Q. ilex* predicted for the LIG, LGM, MH, Current, moderate (RCP 4.5) 2050 and 2070 and pessimistic (RCP 8.5) 2050 and 2070 with GBM algorithm and using MIROC-ESM climate model. |
| --- |

| 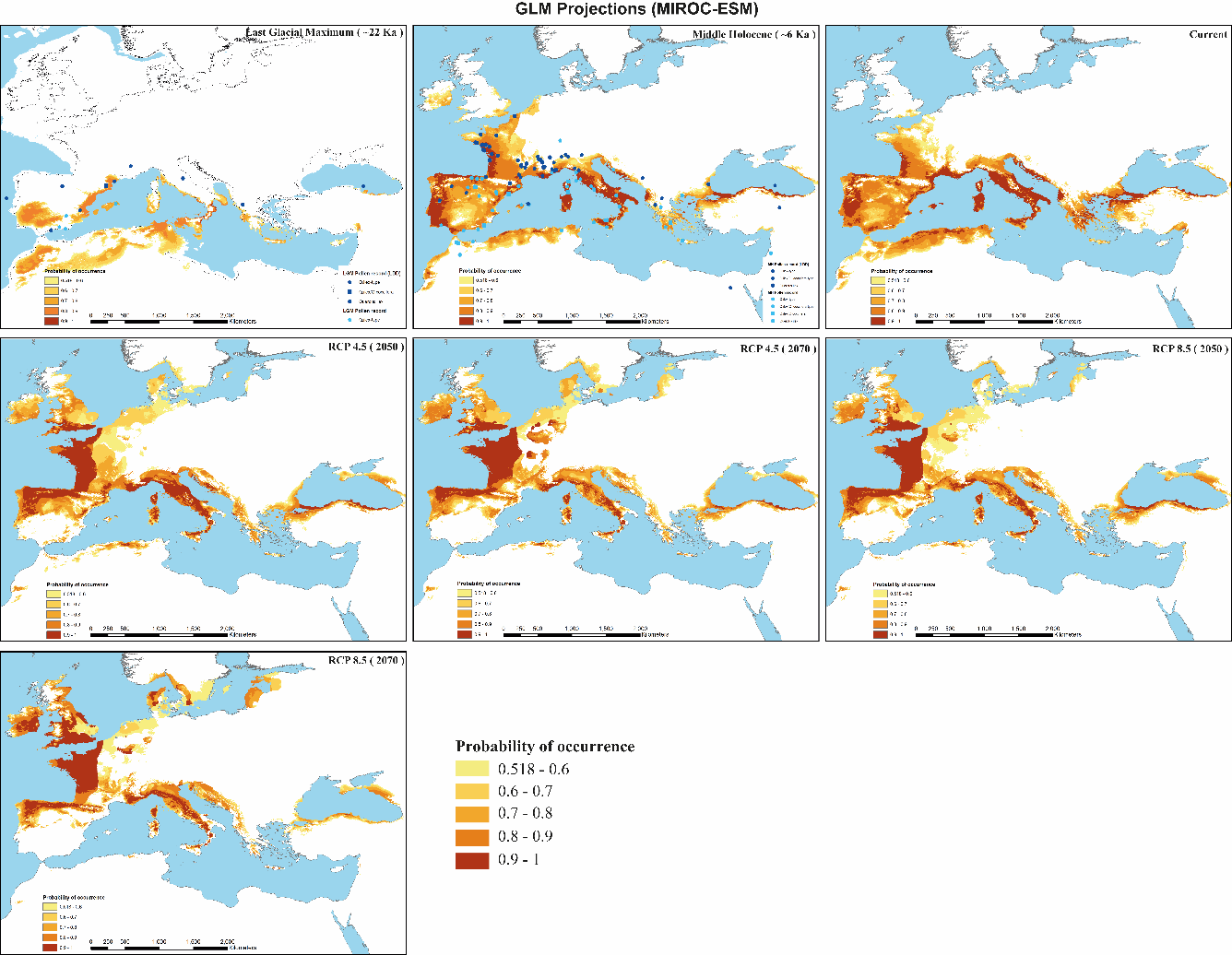  **S20 Figure.** Potential distribution of *Q. ilex* predicted for the LIG, LGM, MH, Current, moderate (RCP 4.5) 2050 and 2070 and pessimistic (RCP 8.5) 2050 and 2070 with GLM algorithm and using MIROC-ESM climate model. |
| --- |

| 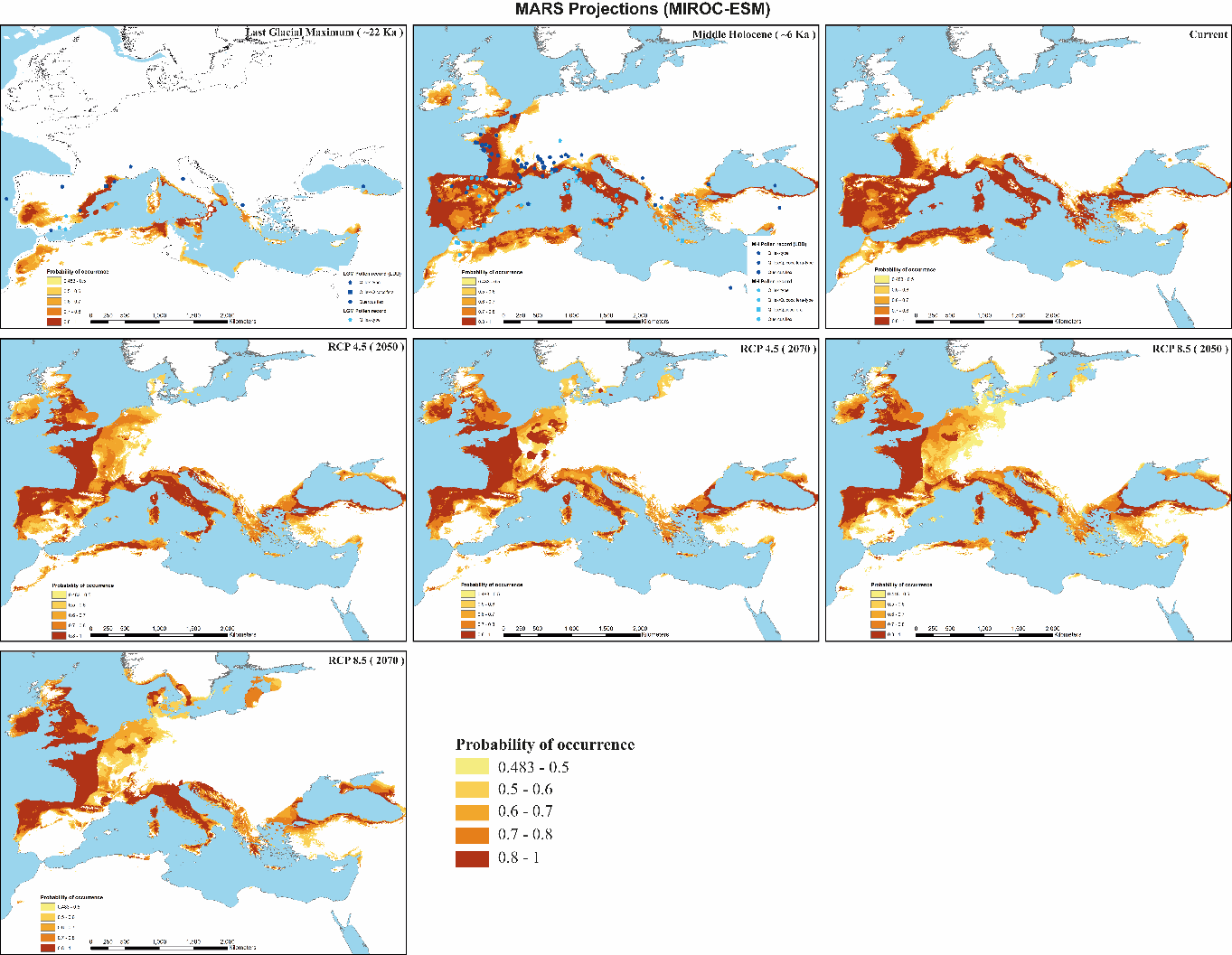  **S21 Figure.** Potential distribution of *Q. ilex* predicted for the LIG, LGM, MH, Current, moderate (RCP 4.5) 2050 and 2070 and pessimistic (RCP 8.5) 2050 and 2070 with MARS algorithm and using MIROC-ESM climate model. |
| --- |

| 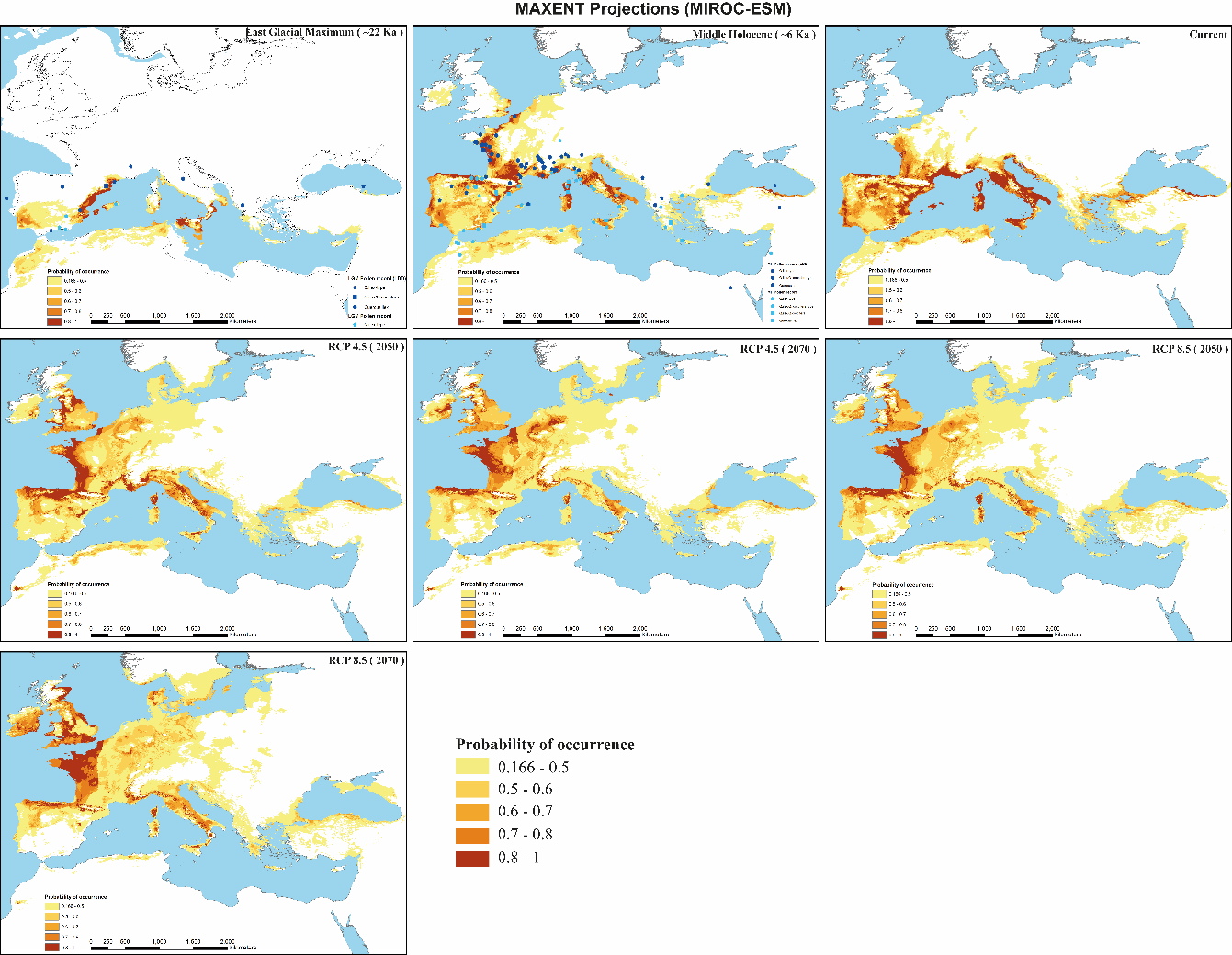  **S22 Figure.** Potential distribution of *Q. ilex* predicted for the LIG, LGM, MH, Current, moderate (RCP 4.5) 2050 and 2070 and pessimistic (RCP 8.5) 2050 and 2070 with MAXENT algorithm and using MIROC-ESM climate model. |
| --- |

| 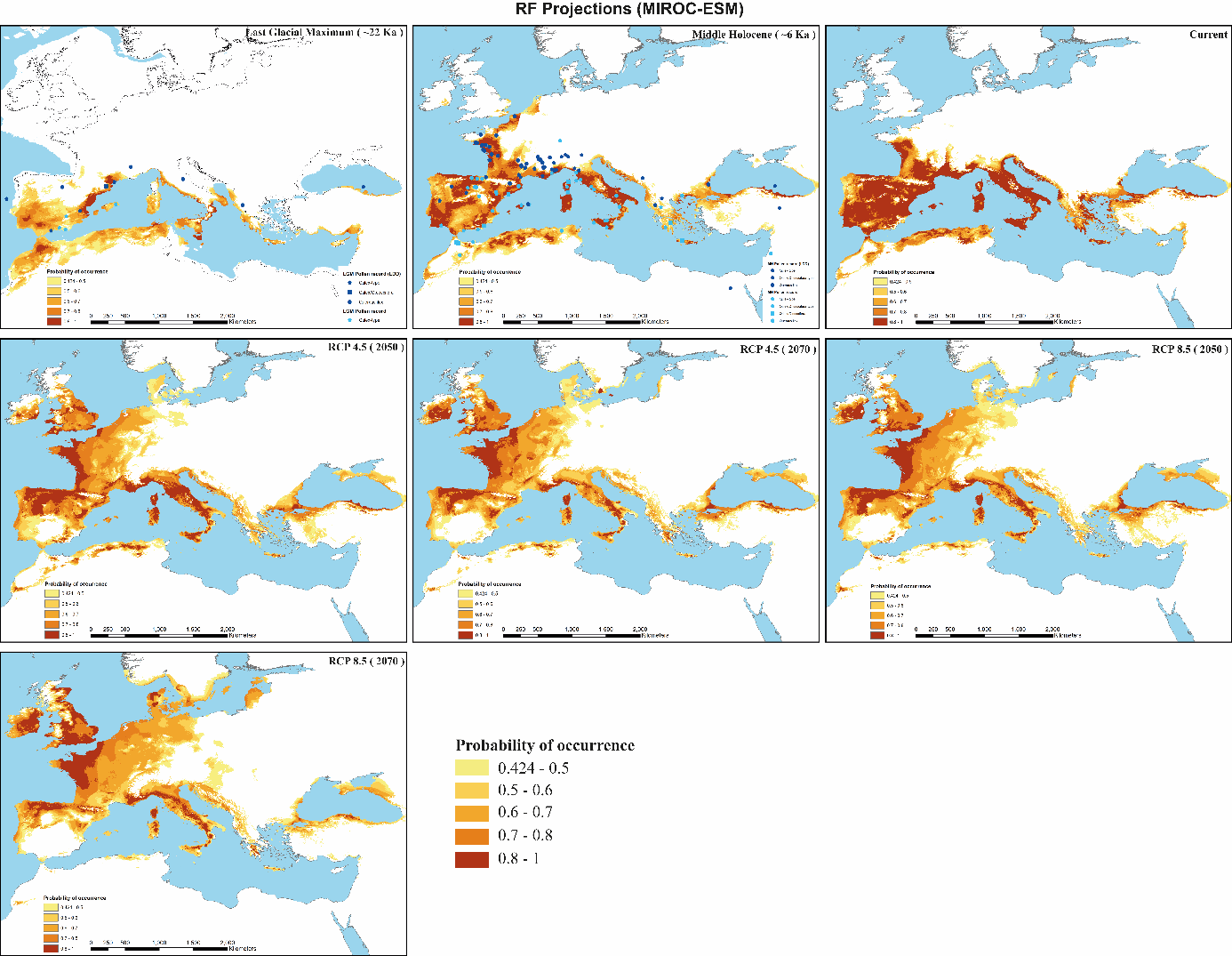  **S23 Figure.** Potential distribution of *Q. ilex* predicted for the LIG, LGM, MH, Current, moderate (RCP 4.5) 2050 and 2070 and pessimistic (RCP 8.5) 2050 and 2070 with RF algorithm and using MIROC-ESM climate model. |
| --- |

| 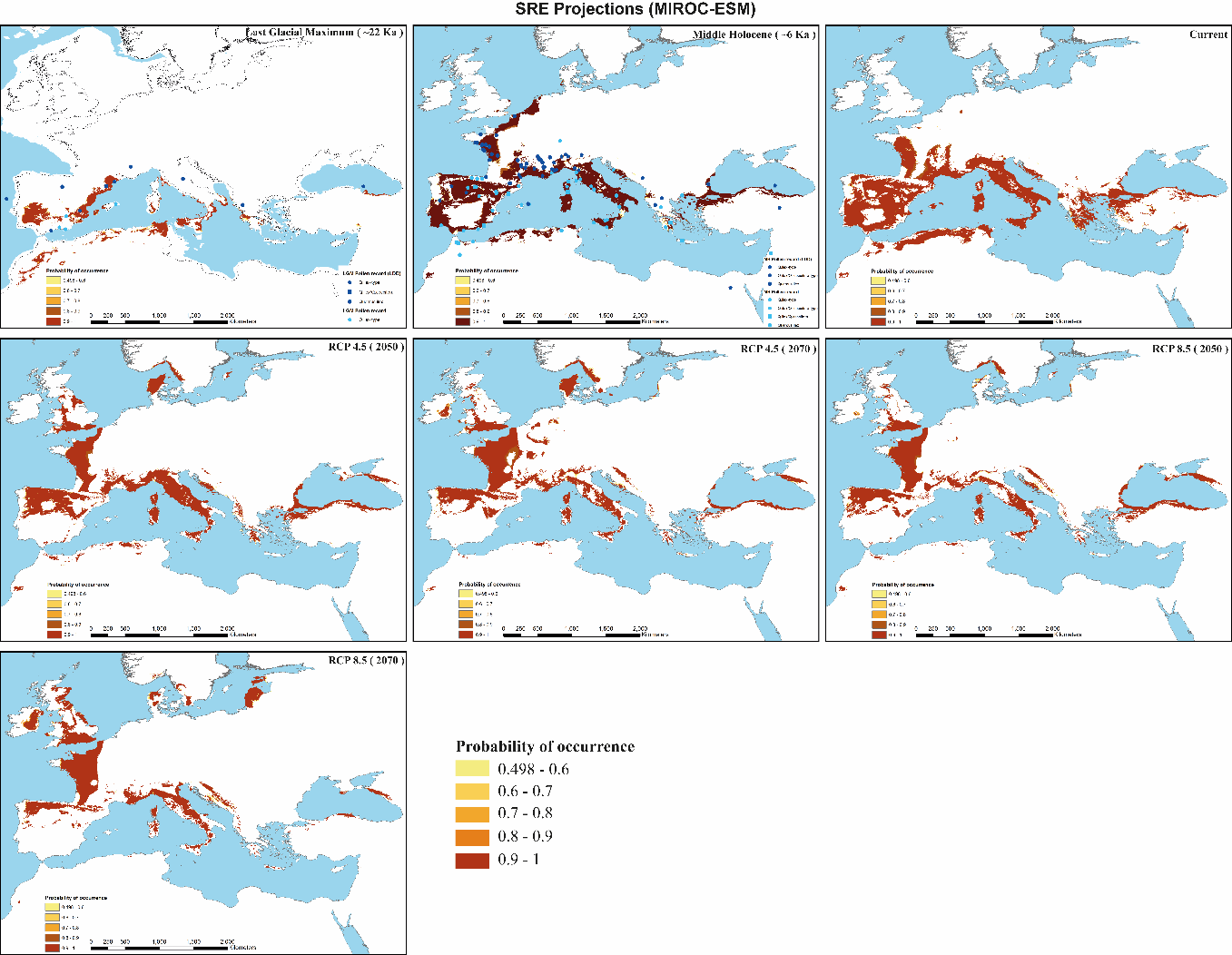  **S24 Figure.** Potential distribution of *Q. ilex* predicted for the LIG, LGM, MH, Current, moderate (RCP 4.5) 2050 and 2070 and pessimistic (RCP 8.5) 2050 and 2070 with SRE algorithm and using MIROC-ESM climate model. |
| --- |

| 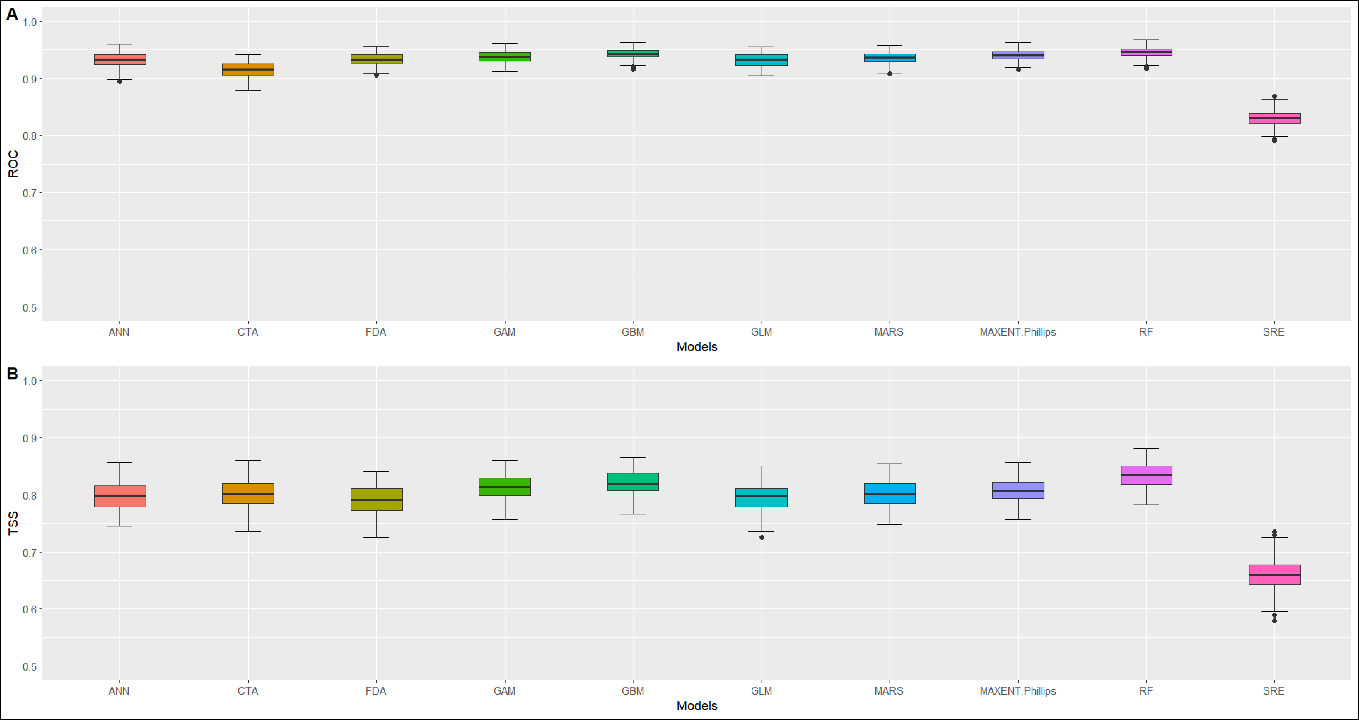  **S25 Figure**. Boxplot of model performance (AUC, TSS, Sensitivity, Specifitiy and Threshold) for 100 repetitions of each algorithm. |
| --- |
