# Supplementary material for: Distribution patterns of Quercus ilex from the last interglacial period to the future by ecological niche modeling
Source: Ecol Evol. 2023 Oct 19;13(10):e10606. doi: 10.1002/ece3.10606 (PMC10585444; doi:10.1002/ece3.10606)
Supplement: Supplementary file 3 — Table S1. [file ECE3-13-e10606-s006.docx]

**S1 Table**. All occurrence records (1606) used in the modeling process (Browicz & Zieliński, 1982; Hedge & Yaltirik, 1982; Akkemik et al., 2021; Global Biodiversity Information Facility (GBIF), 2022; including herbarium specimens).

| **species** | **Longitude** | **Latitude** |
| --- | --- | --- |
| *Quercus ilex* | 12.650 | 43.063 |
| *Quercus ilex* | 14.976 | 44.461 |
| *Quercus ilex* | 11.164 | 43.711 |
| *Quercus ilex* | 1.817 | 41.758 |
| *Quercus ilex* | 11.012 | 43.437 |
| *Quercus ilex* | 1.788 | 41.610 |
| *Quercus ilex* | -2.153 | 43.288 |
| *Quercus ilex* | 12.059 | 43.238 |
| *Quercus ilex* | 2.818 | 41.687 |
| *Quercus ilex* | 4.038 | 43.731 |
| *Quercus ilex* | -4.934 | 43.457 |
| *Quercus ilex* | 11.513 | 43.187 |
| *Quercus ilex* | 13.641 | 45.071 |
| *Quercus ilex* | 11.516 | 43.000 |
| *Quercus ilex* | 5.691 | 43.673 |
| *Quercus ilex* | 4.227 | 43.751 |
| *Quercus ilex* | 13.806 | 44.894 |
| *Quercus ilex* | 5.217 | 43.800 |
| *Quercus ilex* | 2.699 | 39.696 |
| *Quercus ilex* | 16.217 | 43.817 |
| *Quercus ilex* | 6.379 | 43.742 |
| *Quercus ilex* | 16.595 | 43.305 |
| *Quercus ilex* | -2.713 | 47.579 |
| *Quercus ilex* | 14.125 | 44.972 |
| *Quercus ilex* | 13.974 | 44.830 |
| *Quercus ilex* | 17.131 | 42.955 |
| *Quercus ilex* | 15.414 | 39.997 |
| *Quercus ilex* | 16.384 | 43.342 |
| *Quercus ilex* | 13.396 | 41.847 |
| *Quercus ilex* | 14.720 | 44.359 |
| *Quercus ilex* | 3.856 | 43.717 |
| *Quercus ilex* | -4.801 | 43.309 |
| *Quercus ilex* | 17.364 | 42.775 |
| *Quercus ilex* | 12.936 | 43.251 |
| *Quercus ilex* | 3.181 | 39.736 |
| *Quercus ilex* | 2.811 | 39.850 |
| *Quercus ilex* | 2.494 | 39.537 |
| *Quercus ilex* | 16.653 | 42.986 |
| *Quercus ilex* | 16.070 | 43.487 |
| *Quercus ilex* | 9.000 | 40.071 |
| *Quercus ilex* | 14.732 | 44.768 |
| *Quercus ilex* | 16.751 | 43.128 |
| *Quercus ilex* | 3.912 | 39.937 |
| *Quercus ilex* | 2.097 | 41.385 |
| *Quercus ilex* | 3.206 | 41.965 |
| *Quercus ilex* | 11.342 | 43.320 |
| *Quercus ilex* | 9.349 | 42.396 |
| *Quercus ilex* | 14.761 | 44.981 |
| *Quercus ilex* | 2.926 | 39.524 |
| *Quercus ilex* | 4.403 | 44.183 |
| *Quercus ilex* | 3.415 | 43.733 |
| *Quercus ilex* | 2.000 | 41.646 |
| *Quercus ilex* | 15.769 | 43.770 |
| *Quercus ilex* | 2.834 | 42.350 |
| *Quercus ilex* | 12.590 | 38.037 |
| *Quercus ilex* | 9.965 | 44.042 |
| *Quercus ilex* | 10.590 | 43.089 |
| *Quercus ilex* | 2.816 | 43.200 |
| *Quercus ilex* | 26.977 | 37.757 |
| *Quercus ilex* | 20.895 | 37.791 |
| *Quercus ilex* | 13.693 | 45.735 |
| *Quercus ilex* | 10.833 | 45.883 |
| *Quercus ilex* | 1.167 | 45.050 |
| *Quercus ilex* | -2.410 | 38.410 |
| *Quercus ilex* | -4.280 | 37.370 |
| *Quercus ilex* | -2.500 | 43.070 |
| *Quercus ilex* | 6.476 | 43.216 |
| *Quercus ilex* | 9.321 | 41.880 |
| *Quercus ilex* | -1.071 | 46.121 |
| *Quercus ilex* | -0.805 | 46.076 |
| *Quercus ilex* | 7.458 | 43.764 |
| *Quercus ilex* | 2.849 | 42.594 |
| *Quercus ilex* | 5.399 | 43.536 |
| *Quercus ilex* | 4.725 | 44.503 |
| *Quercus ilex* | 1.536 | 43.307 |
| *Quercus ilex* | 2.387 | 43.167 |
| *Quercus ilex* | 4.496 | 43.752 |
| *Quercus ilex* | -0.904 | 45.882 |
| *Quercus ilex* | 7.029 | 43.628 |
| *Quercus ilex* | 4.076 | 44.084 |
| *Quercus ilex* | 6.847 | 43.458 |
| *Quercus ilex* | 6.652 | 43.429 |
| *Quercus ilex* | -1.768 | 46.602 |
| *Quercus ilex* | 4.496 | 44.378 |
| *Quercus ilex* | 4.115 | 44.392 |
| *Quercus ilex* | 1.867 | 43.688 |
| *Quercus ilex* | 6.277 | 43.374 |
| *Quercus ilex* | 2.089 | 43.307 |
| *Quercus ilex* | 9.083 | 42.270 |
| *Quercus ilex* | 9.119 | 42.526 |
| *Quercus ilex* | 8.796 | 42.128 |
| *Quercus ilex* | 9.186 | 41.435 |
| *Quercus ilex* | 9.529 | 42.288 |
| *Quercus ilex* | 8.903 | 42.519 |
| *Quercus ilex* | 8.935 | 41.572 |
| *Quercus ilex* | 9.425 | 42.686 |
| *Quercus ilex* | 9.496 | 42.106 |
| *Quercus ilex* | 8.707 | 42.408 |
| *Quercus ilex* | 8.629 | 42.226 |
| *Quercus ilex* | 8.983 | 42.047 |
| *Quercus ilex* | 8.946 | 41.788 |
| *Quercus ilex* | 9.254 | 41.593 |
| *Quercus ilex* | 9.437 | 42.920 |
| *Quercus ilex* | 8.701 | 41.935 |
| *Quercus ilex* | 9.293 | 42.052 |
| *Quercus ilex* | 9.279 | 42.220 |
| *Quercus ilex* | 8.753 | 41.769 |
| *Quercus ilex* | 9.396 | 42.537 |
| *Quercus ilex* | 9.204 | 42.672 |
| *Quercus ilex* | 9.116 | 41.750 |
| *Quercus ilex* | 4.711 | 43.936 |
| *Quercus ilex* | -1.772 | 47.207 |
| *Quercus ilex* | -0.741 | 47.336 |
| *Quercus ilex* | -1.261 | 47.340 |
| *Quercus ilex* | -1.517 | 47.208 |
| *Quercus ilex* | 6.054 | 43.581 |
| *Quercus ilex* | 4.687 | 44.361 |
| *Quercus ilex* | -2.498 | 47.315 |
| *Quercus ilex* | 6.087 | 43.160 |
| *Quercus ilex* | 5.622 | 43.536 |
| *Quercus ilex* | 5.688 | 43.311 |
| *Quercus ilex* | 5.106 | 44.200 |
| *Quercus ilex* | 1.869 | 43.109 |
| *Quercus ilex* | -0.653 | 47.478 |
| *Quercus ilex* | 5.040 | 43.996 |
| *Quercus ilex* | 1.541 | 44.425 |
| *Quercus ilex* | 1.172 | 43.358 |
| *Quercus ilex* | 2.522 | 42.600 |
| *Quercus ilex* | 5.958 | 44.119 |
| *Quercus ilex* | 5.068 | 43.646 |
| *Quercus ilex* | 5.817 | 43.510 |
| *Quercus ilex* | 4.662 | 43.547 |
| *Quercus ilex* | 4.448 | 44.555 |
| *Quercus ilex* | -1.225 | 45.833 |
| *Quercus ilex* | 4.141 | 44.234 |
| *Quercus ilex* | -1.108 | 44.983 |
| *Quercus ilex* | 1.286 | 43.558 |
| *Quercus ilex* | 6.975 | 44.028 |
| *Quercus ilex* | 6.913 | 43.859 |
| *Quercus ilex* | 4.894 | 44.965 |
| *Quercus ilex* | -2.670 | 43.150 |
| *Quercus ilex* | -3.420 | 43.440 |
| *Quercus ilex* | 3.060 | 42.140 |
| *Quercus ilex* | -3.440 | 42.510 |
| *Quercus ilex* | -3.580 | 43.200 |
| *Quercus ilex* | -2.990 | 42.360 |
| *Quercus ilex* | -3.420 | 42.770 |
| *Quercus ilex* | -3.090 | 42.760 |
| *Quercus ilex* | -5.826 | 39.669 |
| *Quercus ilex* | 2.966 | 43.578 |
| *Quercus ilex* | -5.751 | 39.875 |
| *Quercus ilex* | -6.264 | 39.826 |
| *Quercus ilex* | -3.891 | 40.509 |
| *Quercus ilex* | -1.320 | 42.801 |
| *Quercus ilex* | 4.870 | 43.700 |
| *Quercus ilex* | -4.562 | 43.161 |
| *Quercus ilex* | 5.141 | 44.357 |
| *Quercus ilex* | 4.761 | 44.827 |
| *Quercus ilex* | -2.668 | 41.027 |
| *Quercus ilex* | -3.931 | 38.170 |
| *Quercus ilex* | -4.551 | 36.953 |
| *Quercus ilex* | -6.048 | 39.824 |
| *Quercus ilex* | -5.272 | 41.396 |
| *Quercus ilex* | -1.951 | 42.669 |
| *Quercus ilex* | 3.716 | 43.484 |
| *Quercus ilex* | 6.688 | 43.650 |
| *Quercus ilex* | 2.544 | 43.360 |
| *Quercus ilex* | 3.843 | 44.135 |
| *Quercus ilex* | 0.599 | 42.125 |
| *Quercus ilex* | 6.378 | 43.600 |
| *Quercus ilex* | -2.844 | 38.936 |
| *Quercus ilex* | -5.357 | 39.443 |
| *Quercus ilex* | -5.084 | 36.661 |
| *Quercus ilex* | -5.491 | 39.168 |
| *Quercus ilex* | -3.530 | 42.335 |
| *Quercus ilex* | -6.255 | 43.113 |
| *Quercus ilex* | -2.290 | 39.420 |
| *Quercus ilex* | -2.880 | 40.510 |
| *Quercus ilex* | -4.900 | 39.600 |
| *Quercus ilex* | -4.640 | 39.850 |
| *Quercus ilex* | -4.730 | 40.060 |
| *Quercus ilex* | -3.040 | 38.310 |
| *Quercus ilex* | -4.910 | 39.310 |
| *Quercus ilex* | -4.540 | 39.150 |
| *Quercus ilex* | -4.170 | 38.700 |
| *Quercus ilex* | -5.210 | 39.640 |
| *Quercus ilex* | -4.350 | 39.860 |
| *Quercus ilex* | -2.840 | 40.320 |
| *Quercus ilex* | -2.700 | 39.550 |
| *Quercus ilex* | -3.250 | 40.500 |
| *Quercus ilex* | -3.050 | 38.890 |
| *Quercus ilex* | -3.180 | 39.980 |
| *Quercus ilex* | -4.290 | 38.840 |
| *Quercus ilex* | -2.630 | 40.740 |
| *Quercus ilex* | -3.610 | 40.670 |
| *Quercus ilex* | -3.360 | 40.160 |
| *Quercus ilex* | -2.940 | 40.750 |
| *Quercus ilex* | -5.400 | 39.800 |
| *Quercus ilex* | -3.270 | 38.540 |
| *Quercus ilex* | -4.150 | 39.690 |
| *Quercus ilex* | -4.320 | 40.010 |
| *Quercus ilex* | -2.730 | 40.030 |
| *Quercus ilex* | -1.740 | 39.440 |
| *Quercus ilex* | -2.840 | 38.600 |
| *Quercus ilex* | -3.010 | 38.730 |
| *Quercus ilex* | -6.200 | 40.260 |
| *Quercus ilex* | -2.980 | 39.900 |
| *Quercus ilex* | -2.580 | 38.380 |
| *Quercus ilex* | -4.010 | 40.280 |
| *Quercus ilex* | -4.110 | 40.050 |
| *Quercus ilex* | -4.400 | 39.460 |
| *Quercus ilex* | -3.230 | 40.330 |
| *Quercus ilex* | -4.970 | 39.980 |
| *Quercus ilex* | -3.050 | 40.400 |
| *Quercus ilex* | -2.390 | 40.770 |
| *Quercus ilex* | -4.460 | 39.630 |
| *Quercus ilex* | -3.020 | 40.990 |
| *Quercus ilex* | -2.790 | 39.290 |
| *Quercus ilex* | -2.810 | 39.780 |
| *Quercus ilex* | -3.120 | 39.400 |
| *Quercus ilex* | -3.380 | 37.720 |
| *Quercus ilex* | -3.370 | 38.990 |
| *Quercus ilex* | -3.060 | 38.580 |
| *Quercus ilex* | -3.340 | 40.790 |
| *Quercus ilex* | -3.480 | 40.530 |
| *Quercus ilex* | -3.560 | 38.520 |
| *Quercus ilex* | -1.520 | 39.170 |
| *Quercus ilex* | -3.930 | 39.560 |
| *Quercus ilex* | -6.530 | 39.980 |
| *Quercus ilex* | -4.940 | 39.760 |
| *Quercus ilex* | -4.850 | 38.720 |
| *Quercus ilex* | -5.150 | 39.930 |
| *Quercus ilex* | -3.500 | 39.690 |
| *Quercus ilex* | -2.840 | 40.950 |
| *Quercus ilex* | -2.910 | 40.120 |
| *Quercus ilex* | -2.420 | 40.400 |
| *Quercus ilex* | -4.060 | 38.840 |
| *Quercus ilex* | -4.500 | 40.150 |
| *Quercus ilex* | -2.640 | 40.500 |
| *Quercus ilex* | -5.040 | 39.160 |
| *Quercus ilex* | -4.050 | 40.420 |
| *Quercus ilex* | -1.720 | 38.910 |
| *Quercus ilex* | -4.600 | 38.940 |
| *Quercus ilex* | -4.300 | 38.400 |
| *Quercus ilex* | -3.870 | 39.710 |
| *Quercus ilex* | -3.230 | 40.930 |
| *Quercus ilex* | -4.290 | 38.980 |
| *Quercus ilex* | -3.470 | 39.880 |
| *Quercus ilex* | -4.300 | 39.260 |
| *Quercus ilex* | -2.570 | 39.380 |
| *Quercus ilex* | -3.010 | 40.260 |
| *Quercus ilex* | -3.610 | 39.070 |
| *Quercus ilex* | -2.150 | 39.200 |
| *Quercus ilex* | -1.320 | 38.780 |
| *Quercus ilex* | -1.600 | 39.670 |
| *Quercus ilex* | -3.080 | 39.120 |
| *Quercus ilex* | -3.770 | 38.700 |
| *Quercus ilex* | -1.300 | 39.100 |
| *Quercus ilex* | -3.600 | 39.210 |
| *Quercus ilex* | -3.900 | 39.120 |
| *Quercus ilex* | -4.060 | 39.040 |
| *Quercus ilex* | -2.720 | 38.210 |
| *Quercus ilex* | -0.860 | 38.630 |
| *Quercus ilex* | -1.940 | 39.620 |
| *Quercus ilex* | -2.100 | 38.360 |
| *Quercus ilex* | -3.680 | 40.490 |
| *Quercus ilex* | -2.630 | 38.570 |
| *Quercus ilex* | -2.370 | 39.660 |
| *Quercus ilex* | -2.550 | 38.100 |
| *Quercus ilex* | -4.190 | 40.230 |
| *Quercus ilex* | 3.280 | 42.320 |
| *Quercus ilex* | 2.480 | 42.070 |
| *Quercus ilex* | -4.535 | 43.397 |
| *Quercus ilex* | 10.351 | 43.665 |
| *Quercus ilex* | 12.257 | 44.815 |
| *Quercus ilex* | -2.936 | 43.413 |
| *Quercus ilex* | 5.077 | 43.398 |
| *Quercus ilex* | -1.663 | 42.812 |
| *Quercus ilex* | 15.979 | 43.908 |
| *Quercus ilex* | 9.578 | 40.690 |
| *Quercus ilex* | 15.009 | 37.619 |
| *Quercus ilex* | 10.844 | 43.683 |
| *Quercus ilex* | 14.228 | 40.549 |
| *Quercus ilex* | 1.984 | 42.110 |
| *Quercus ilex* | 28.060 | 36.341 |
| *Quercus ilex* | 13.297 | 38.215 |
| *Quercus ilex* | 12.524 | 42.504 |
| *Quercus ilex* | 15.968 | 43.707 |
| *Quercus ilex* | 17.763 | 40.309 |
| *Quercus ilex* | 1.485 | 38.932 |
| *Quercus ilex* | 25.912 | 36.845 |
| *Quercus ilex* | 3.664 | 43.639 |
| *Quercus ilex* | 16.812 | 42.766 |
| *Quercus ilex* | -5.567 | 42.581 |
| *Quercus ilex* | 8.663 | 44.416 |
| *Quercus ilex* | 1.754 | 41.250 |
| *Quercus ilex* | 12.248 | 42.490 |
| *Quercus ilex* | 12.335 | 45.095 |
| *Quercus ilex* | 2.257 | 42.081 |
| *Quercus ilex* | 15.147 | 44.197 |
| *Quercus ilex* | 16.086 | 41.742 |
| *Quercus ilex* | 18.051 | 42.686 |
| *Quercus ilex* | 4.541 | 43.954 |
| *Quercus ilex* | 13.606 | 45.316 |
| *Quercus ilex* | -3.271 | 36.993 |
| *Quercus ilex* | -5.585 | 33.548 |
| *Quercus ilex* | -1.694 | 42.502 |
| *Quercus ilex* | -4.775 | 37.905 |
| *Quercus ilex* | -1.154 | 42.661 |
| *Quercus ilex* | -5.645 | 43.535 |
| *Quercus ilex* | 2.585 | 42.831 |
| *Quercus ilex* | 8.308 | 40.586 |
| *Quercus ilex* | -4.856 | 37.248 |
| *Quercus ilex* | -4.826 | 36.672 |
| *Quercus ilex* | 3.520 | 43.890 |
| *Quercus ilex* | -3.487 | 42.171 |
| *Quercus ilex* | 3.020 | 39.857 |
| *Quercus ilex* | 9.516 | 40.222 |
| *Quercus ilex* | -7.187 | 39.955 |
| *Quercus ilex* | -1.645 | 42.666 |
| *Quercus ilex* | -9.046 | 29.779 |
| *Quercus ilex* | 12.833 | 37.585 |
| *Quercus ilex* | -6.736 | 41.394 |
| *Quercus ilex* | 2.925 | 43.399 |
| *Quercus ilex* | 9.244 | 39.990 |
| *Quercus ilex* | -2.232 | 42.643 |
| *Quercus ilex* | -0.727 | 42.355 |
| *Quercus ilex* | -2.646 | 36.884 |
| *Quercus ilex* | 9.714 | 40.395 |
| *Quercus ilex* | -4.101 | 42.637 |
| *Quercus ilex* | 1.830 | 43.330 |
| *Quercus ilex* | -4.012 | 40.663 |
| *Quercus ilex* | -1.474 | 42.487 |
| *Quercus ilex* | 21.722 | 36.836 |
| *Quercus ilex* | 0.414 | 40.404 |
| *Quercus ilex* | 3.186 | 39.430 |
| *Quercus ilex* | -5.544 | 40.549 |
| *Quercus ilex* | -1.835 | 42.929 |
| *Quercus ilex* | -2.911 | 37.903 |
| *Quercus ilex* | -2.220 | 37.500 |
| *Quercus ilex* | 10.212 | 42.733 |
| *Quercus ilex* | 14.527 | 44.962 |
| *Quercus ilex* | 12.804 | 41.966 |
| *Quercus ilex* | 14.516 | 44.610 |
| *Quercus ilex* | 17.013 | 43.294 |
| *Quercus ilex* | 13.906 | 42.047 |
| *Quercus ilex* | 24.912 | 35.167 |
| *Quercus ilex* | 11.518 | 42.721 |
| *Quercus ilex* | 14.904 | 44.161 |
| *Quercus ilex* | 14.353 | 45.111 |
| *Quercus ilex* | 15.448 | 43.933 |
| *Quercus ilex* | 14.325 | 44.873 |
| *Quercus ilex* | 1.544 | 43.028 |
| *Quercus ilex* | 1.985 | 42.969 |
| *Quercus ilex* | 1.350 | 43.044 |
| *Quercus ilex* | 1.689 | 44.039 |
| *Quercus ilex* | 2.104 | 43.467 |
| *Quercus ilex* | 3.048 | 43.088 |
| *Quercus ilex* | 1.364 | 43.704 |
| *Quercus ilex* | 0.960 | 44.338 |
| *Quercus ilex* | 0.635 | 43.668 |
| *Quercus ilex* | 1.958 | 43.913 |
| *Quercus ilex* | 1.474 | 43.539 |
| *Quercus ilex* | 1.635 | 42.833 |
| *Quercus ilex* | 3.029 | 42.497 |
| *Quercus ilex* | 2.816 | 42.820 |
| *Quercus ilex* | 3.607 | 44.103 |
| *Quercus ilex* | 3.795 | 43.875 |
| *Quercus ilex* | 5.876 | 43.843 |
| *Quercus ilex* | 2.671 | 42.453 |
| *Quercus ilex* | 6.125 | 43.728 |
| *Quercus ilex* | 5.389 | 43.799 |
| *Quercus ilex* | 5.379 | 43.950 |
| *Quercus ilex* | 5.586 | 43.919 |
| *Quercus ilex* | 2.157 | 42.881 |
| *Quercus ilex* | 7.575 | 44.019 |
| *Quercus ilex* | 7.280 | 44.044 |
| *Quercus ilex* | -0.612 | 45.860 |
| *Quercus ilex* | 4.232 | 44.533 |
| *Quercus ilex* | 5.244 | 45.057 |
| *Quercus ilex* | 0.978 | 44.898 |
| *Quercus ilex* | 2.576 | 43.037 |
| *Quercus ilex* | 5.796 | 43.139 |
| *Quercus ilex* | 0.686 | 45.259 |
| *Quercus ilex* | 3.997 | 43.918 |
| *Quercus ilex* | -1.311 | 47.104 |
| *Quercus ilex* | -0.792 | 45.616 |
| *Quercus ilex* | 1.326 | 44.823 |
| *Quercus ilex* | 1.541 | 44.697 |
| *Quercus ilex* | 2.770 | 42.959 |
| *Quercus ilex* | -1.042 | 44.649 |
| *Quercus ilex* | 5.113 | 44.811 |
| *Quercus ilex* | 1.204 | 45.229 |
| *Quercus ilex* | -2.049 | 46.802 |
| *Quercus ilex* | -1.065 | 45.181 |
| *Quercus ilex* | 3.300 | 43.555 |
| *Quercus ilex* | 5.952 | 43.363 |
| *Quercus ilex* | -0.372 | 47.460 |
| *Quercus ilex* | 5.572 | 44.354 |
| *Quercus ilex* | 0.245 | 47.172 |
| *Quercus ilex* | 4.590 | 44.209 |
| *Quercus ilex* | 5.335 | 43.371 |
| *Quercus ilex* | 4.902 | 44.413 |
| *Quercus ilex* | 3.467 | 43.317 |
| *Quercus ilex* | 2.337 | 42.578 |
| *Quercus ilex* | -2.473 | 41.351 |
| *Quercus ilex* | 1.631 | 44.851 |
| *Quercus ilex* | 6.246 | 44.100 |
| *Quercus ilex* | 5.326 | 44.183 |
| *Quercus ilex* | 7.172 | 43.828 |
| *Quercus ilex* | 3.933 | 44.428 |
| *Quercus ilex* | 0.680 | 43.240 |
| *Quercus ilex* | -2.313 | 47.549 |
| *Quercus ilex* | 4.789 | 44.196 |
| *Quercus ilex* | 4.821 | 44.645 |
| *Quercus ilex* | 5.117 | 44.512 |
| *Quercus ilex* | 4.577 | 44.848 |
| *Quercus ilex* | -1.516 | 46.461 |
| *Quercus ilex* | 4.532 | 44.993 |
| *Quercus ilex* | 2.376 | 43.486 |
| *Quercus ilex* | 5.046 | 43.812 |
| *Quercus ilex* | 2.497 | 42.408 |
| *Quercus ilex* | 4.168 | 43.921 |
| *Quercus ilex* | 2.351 | 42.804 |
| *Quercus ilex* | 1.130 | 44.649 |
| *Quercus ilex* | 0.733 | 44.859 |
| *Quercus ilex* | -1.276 | 45.983 |
| *Quercus ilex* | 2.721 | 43.450 |
| *Quercus ilex* | -1.130 | 45.321 |
| *Quercus ilex* | 9.159 | 41.918 |
| *Quercus ilex* | 3.841 | 44.285 |
| *Quercus ilex* | 2.216 | 43.127 |
| *Quercus ilex* | 3.120 | 43.717 |
| *Quercus ilex* | 6.199 | 43.957 |
| *Quercus ilex* | 2.290 | 43.325 |
| *Quercus ilex* | 2.339 | 42.965 |
| *Quercus ilex* | 4.243 | 44.693 |
| *Quercus ilex* | 5.233 | 43.592 |
| *Quercus ilex* | 9.355 | 41.743 |
| *Quercus ilex* | -1.245 | 44.525 |
| *Quercus ilex* | 4.845 | 45.185 |
| *Quercus ilex* | 6.471 | 43.425 |
| *Quercus ilex* | 0.891 | 44.142 |
| *Quercus ilex* | -1.101 | 45.664 |
| *Quercus ilex* | 5.976 | 43.983 |
| *Quercus ilex* | 3.037 | 44.078 |
| *Quercus ilex* | 2.987 | 42.909 |
| *Quercus ilex* | 6.862 | 43.680 |
| *Quercus ilex* | 5.416 | 43.233 |
| *Quercus ilex* | 3.291 | 43.262 |
| *Quercus ilex* | 3.089 | 43.232 |
| *Quercus ilex* | 3.488 | 43.516 |
| *Quercus ilex* | 8.874 | 42.279 |
| *Quercus ilex* | 1.151 | 44.811 |
| *Quercus ilex* | 1.401 | 44.975 |
| *Quercus ilex* | -1.126 | 44.835 |
| *Quercus ilex* | 1.307 | 43.212 |
| *Quercus ilex* | 4.368 | 43.953 |
| *Quercus ilex* | 5.868 | 43.645 |
| *Quercus ilex* | 1.307 | 44.578 |
| *Quercus ilex* | 5.682 | 44.064 |
| *Quercus ilex* | 5.058 | 45.108 |
| *Quercus ilex* | 6.260 | 43.217 |
| *Quercus ilex* | 2.609 | 43.181 |
| *Quercus ilex* | -2.905 | 47.614 |
| *Quercus ilex* | 1.836 | 44.582 |
| *Quercus ilex* | 3.116 | 43.430 |
| *Quercus ilex* | -1.080 | 47.394 |
| *Quercus ilex* | 1.071 | 43.893 |
| *Quercus ilex* | -1.798 | 46.740 |
| *Quercus ilex* | -2.318 | 47.296 |
| *Quercus ilex* | 4.307 | 44.368 |
| *Quercus ilex* | -0.893 | 46.499 |
| *Quercus ilex* | 6.501 | 43.972 |
| *Quercus ilex* | 13.994 | 45.665 |
| *Quercus ilex* | 17.415 | 40.703 |
| *Quercus ilex* | 22.460 | 40.074 |
| *Quercus ilex* | 2.526 | 39.685 |
| *Quercus ilex* | 13.585 | 43.523 |
| *Quercus ilex* | 14.027 | 38.000 |
| *Quercus ilex* | 12.804 | 45.565 |
| *Quercus ilex* | 12.100 | 42.006 |
| *Quercus ilex* | 9.726 | 44.107 |
| *Quercus ilex* | 15.167 | 43.940 |
| *Quercus ilex* | -0.987 | 47.205 |
| *Quercus ilex* | 11.282 | 42.420 |
| *Quercus ilex* | 23.735 | 37.975 |
| *Quercus ilex* | 20.840 | 39.615 |
| *Quercus ilex* | 2.355 | 41.823 |
| *Quercus ilex* | 11.133 | 42.754 |
| *Quercus ilex* | 1.881 | 42.263 |
| *Quercus ilex* | 12.691 | 42.302 |
| *Quercus ilex* | 2.324 | 41.527 |
| *Quercus ilex* | -5.828 | 43.387 |
| *Quercus ilex* | 11.589 | 43.377 |
| *Quercus ilex* | 1.551 | 41.620 |
| *Quercus ilex* | 8.892 | 40.888 |
| *Quercus ilex* | 10.422 | 42.781 |
| *Quercus ilex* | 1.527 | 42.257 |
| *Quercus ilex* | 10.608 | 43.767 |
| *Quercus ilex* | 8.978 | 40.614 |
| *Quercus ilex* | 9.043 | 40.234 |
| *Quercus ilex* | 18.168 | 40.357 |
| *Quercus ilex* | 23.965 | 35.413 |
| *Quercus ilex* | 8.991 | 39.705 |
| *Quercus ilex* | 14.618 | 37.147 |
| *Quercus ilex* | -8.004 | 37.201 |
| *Quercus ilex* | 14.295 | 45.319 |
| *Quercus ilex* | 23.931 | 39.241 |
| *Quercus ilex* | 13.883 | 45.379 |
| *Quercus ilex* | 10.288 | 43.998 |
| *Quercus ilex* | 0.547 | 40.642 |
| *Quercus ilex* | 22.315 | 40.210 |
| *Quercus ilex* | 12.369 | 44.243 |
| *Quercus ilex* | 16.708 | 40.880 |
| *Quercus ilex* | 20.683 | 38.717 |
| *Quercus ilex* | 10.884 | 42.365 |
| *Quercus ilex* | 14.526 | 40.621 |
| *Quercus ilex* | -4.027 | 43.435 |
| *Quercus ilex* | 10.264 | 43.819 |
| *Quercus ilex* | 21.362 | 39.015 |
| *Quercus ilex* | 10.670 | 45.693 |
| *Quercus ilex* | 16.292 | 39.603 |
| *Quercus ilex* | 10.450 | 43.509 |
| *Quercus ilex* | 11.470 | 43.703 |
| *Quercus ilex* | 8.474 | 44.430 |
| *Quercus ilex* | 10.627 | 43.453 |
| *Quercus ilex* | 11.298 | 43.091 |
| *Quercus ilex* | 16.263 | 39.845 |
| *Quercus ilex* | 10.448 | 43.870 |
| *Quercus ilex* | 2.451 | 42.259 |
| *Quercus ilex* | 8.932 | 44.435 |
| *Quercus ilex* | 13.603 | 45.538 |
| *Quercus ilex* | 12.069 | 43.020 |
| *Quercus ilex* | 17.569 | 42.724 |
| *Quercus ilex* | 12.438 | 41.884 |
| *Quercus ilex* | 12.875 | 35.858 |
| *Quercus ilex* | 12.336 | 37.998 |
| *Quercus ilex* | 13.232 | 38.015 |
| *Quercus ilex* | 13.259 | 37.663 |
| *Quercus ilex* | 15.000 | 37.130 |
| *Quercus ilex* | 13.734 | 37.944 |
| *Quercus ilex* | 13.403 | 38.075 |
| *Quercus ilex* | 13.377 | 37.883 |
| *Quercus ilex* | 11.921 | 36.787 |
| *Quercus ilex* | 13.159 | 38.706 |
| *Quercus ilex* | 14.931 | 38.488 |
| *Quercus ilex* | 12.057 | 37.970 |
| *Quercus ilex* | 13.417 | 37.650 |
| *Quercus ilex* | 14.000 | 37.850 |
| *Quercus ilex* | 12.858 | 38.004 |
| *Quercus ilex* | 13.605 | 37.622 |
| *Quercus ilex* | 15.270 | 40.228 |
| *Quercus ilex* | 9.096 | 41.094 |
| *Quercus ilex* | 14.669 | 37.926 |
| *Quercus ilex* | 15.533 | 38.210 |
| *Quercus ilex* | 14.202 | 40.859 |
| *Quercus ilex* | 15.770 | 39.910 |
| *Quercus ilex* | 10.915 | 42.893 |
| *Quercus ilex* | 9.301 | 44.325 |
| *Quercus ilex* | 7.779 | 43.857 |
| *Quercus ilex* | 9.624 | 44.261 |
| *Quercus ilex* | 8.338 | 44.212 |
| *Quercus ilex* | 9.116 | 44.400 |
| *Quercus ilex* | 11.869 | 43.571 |
| *Quercus ilex* | 11.471 | 43.519 |
| *Quercus ilex* | 11.655 | 43.510 |
| *Quercus ilex* | 11.857 | 43.432 |
| *Quercus ilex* | 10.905 | 43.821 |
| *Quercus ilex* | 10.490 | 42.953 |
| *Quercus ilex* | 10.544 | 44.017 |
| *Quercus ilex* | 10.979 | 43.145 |
| *Quercus ilex* | 10.792 | 43.125 |
| *Quercus ilex* | 11.460 | 42.476 |
| *Quercus ilex* | 10.744 | 42.963 |
| *Quercus ilex* | 12.291 | 43.760 |
| *Quercus ilex* | 11.094 | 42.892 |
| *Quercus ilex* | 8.859 | 39.194 |
| *Quercus ilex* | 9.058 | 40.442 |
| *Quercus ilex* | 2.358 | 41.669 |
| *Quercus ilex* | 1.621 | 41.796 |
| *Quercus ilex* | 14.254 | 44.642 |
| *Quercus ilex* | -6.970 | 38.880 |
| *Quercus ilex* | -6.630 | 38.910 |
| *Quercus ilex* | 12.980 | 41.860 |
| *Quercus ilex* | -5.490 | 34.030 |
| *Quercus ilex* | 0.740 | 41.980 |
| *Quercus ilex* | 0.495 | 45.457 |
| *Quercus ilex* | 2.884 | 43.848 |
| *Quercus ilex* | 4.994 | 45.842 |
| *Quercus ilex* | 3.283 | 44.147 |
| *Quercus ilex* | 0.784 | 44.515 |
| *Quercus ilex* | 4.785 | 45.502 |
| *Quercus ilex* | 0.090 | 47.385 |
| *Quercus ilex* | -1.862 | 46.902 |
| *Quercus ilex* | -1.120 | 46.383 |
| *Quercus ilex* | 1.850 | 43.516 |
| *Quercus ilex* | 1.646 | 43.794 |
| *Quercus ilex* | 0.680 | 43.026 |
| *Quercus ilex* | -1.055 | 45.459 |
| *Quercus ilex* | -1.613 | 46.927 |
| *Quercus ilex* | 0.001 | 47.193 |
| *Quercus ilex* | -1.500 | 47.362 |
| *Quercus ilex* | 1.148 | 44.407 |
| *Quercus ilex* | 0.931 | 43.187 |
| *Quercus ilex* | -0.348 | 47.799 |
| *Quercus ilex* | -1.329 | 46.700 |
| *Quercus ilex* | 23.816 | 40.185 |
| *Quercus ilex* | 1.917 | 41.416 |
| *Quercus ilex* | 2.741 | 41.949 |
| *Quercus ilex* | 26.402 | 39.096 |
| *Quercus ilex* | 13.237 | 41.603 |
| *Quercus ilex* | 1.056 | 41.338 |
| *Quercus ilex* | 2.765 | 42.118 |
| *Quercus ilex* | -2.688 | 43.388 |
| *Quercus ilex* | 12.247 | 44.672 |
| *Quercus ilex* | 1.689 | 45.060 |
| *Quercus ilex* | 1.360 | 44.084 |
| *Quercus ilex* | 0.823 | 44.676 |
| *Quercus ilex* | 1.327 | 44.367 |
| *Quercus ilex* | -0.993 | 46.686 |
| *Quercus ilex* | -2.064 | 47.097 |
| *Quercus ilex* | -0.533 | 47.287 |
| *Quercus ilex* | 21.254 | 39.329 |
| *Quercus ilex* | 21.165 | 39.166 |
| *Quercus ilex* | 12.753 | 42.725 |
| *Quercus ilex* | 15.785 | 38.263 |
| *Quercus ilex* | -0.954 | 41.140 |
| *Quercus ilex* | 26.691 | 37.751 |
| *Quercus ilex* | 10.773 | 43.307 |
| *Quercus ilex* | 22.980 | 36.597 |
| *Quercus ilex* | 10.573 | 45.558 |
| *Quercus ilex* | -2.739 | 42.897 |
| *Quercus ilex* | 23.735 | 38.169 |
| *Quercus ilex* | 3.370 | 39.660 |
| *Quercus ilex* | 0.220 | 40.660 |
| *Quercus ilex* | 0.800 | 41.280 |
| *Quercus ilex* | -5.390 | 43.500 |
| *Quercus ilex* | 27.215 | 36.854 |
| *Quercus ilex* | 14.781 | 41.123 |
| *Quercus ilex* | 23.636 | 39.179 |
| *Quercus ilex* | 17.554 | 40.322 |
| *Quercus ilex* | 2.531 | 41.611 |
| *Quercus ilex* | 19.687 | 39.678 |
| *Quercus ilex* | -6.436 | 41.562 |
| *Quercus ilex* | 16.140 | 38.330 |
| *Quercus ilex* | 25.490 | 35.200 |
| *Quercus ilex* | 16.436 | 38.467 |
| *Quercus ilex* | 18.435 | 40.269 |
| *Quercus ilex* | 14.538 | 42.224 |
| *Quercus ilex* | -2.148 | 42.967 |
| *Quercus ilex* | -2.957 | 42.988 |
| *Quercus ilex* | -3.060 | 43.220 |
| *Quercus ilex* | -3.228 | 43.186 |
| *Quercus ilex* | -1.240 | 42.480 |
| *Quercus ilex* | -2.463 | 43.347 |
| *Quercus ilex* | -1.600 | 42.310 |
| *Quercus ilex* | 18.535 | 42.450 |
| *Quercus ilex* | 15.111 | 41.060 |
| *Quercus ilex* | 13.668 | 42.773 |
| *Quercus ilex* | 11.925 | 42.674 |
| *Quercus ilex* | -3.849 | 37.771 |
| *Quercus ilex* | -8.675 | 39.629 |
| *Quercus ilex* | -0.609 | 38.768 |
| *Quercus ilex* | -4.547 | 36.728 |
| *Quercus ilex* | 12.301 | 42.094 |
| *Quercus ilex* | -7.388 | 42.442 |
| *Quercus ilex* | 9.496 | 39.943 |
| *Quercus ilex* | 16.239 | 39.368 |
| *Quercus ilex* | 0.031 | 40.078 |
| *Quercus ilex* | 22.959 | 40.639 |
| *Quercus ilex* | -4.260 | 40.513 |
| *Quercus ilex* | 11.797 | 42.312 |
| *Quercus ilex* | -2.497 | 47.518 |
| *Quercus ilex* | -6.754 | 42.454 |
| *Quercus ilex* | 16.717 | 40.416 |
| *Quercus ilex* | 13.867 | 42.872 |
| *Quercus ilex* | 4.095 | 39.986 |
| *Quercus ilex* | -0.406 | 39.468 |
| *Quercus ilex* | 13.013 | 43.838 |
| *Quercus ilex* | -3.653 | 43.398 |
| *Quercus ilex* | 13.141 | 41.753 |
| *Quercus ilex* | 12.504 | 43.577 |
| *Quercus ilex* | 12.301 | 44.431 |
| *Quercus ilex* | -4.757 | 41.644 |
| *Quercus ilex* | -2.430 | 42.456 |
| *Quercus ilex* | 15.504 | 44.213 |
| *Quercus ilex* | -2.775 | 42.616 |
| *Quercus ilex* | 12.612 | 43.861 |
| *Quercus ilex* | 14.127 | 42.424 |
| *Quercus ilex* | 13.447 | 43.298 |
| *Quercus ilex* | -5.637 | 40.964 |
| *Quercus ilex* | 11.225 | 43.479 |
| *Quercus ilex* | 14.273 | 41.140 |
| *Quercus ilex* | 12.047 | 44.215 |
| *Quercus ilex* | 15.041 | 37.989 |
| *Quercus ilex* | 18.219 | 42.613 |
| *Quercus ilex* | 15.196 | 40.521 |
| *Quercus ilex* | 0.867 | 41.051 |
| *Quercus ilex* | 12.497 | 41.665 |
| *Quercus ilex* | 4.932 | 45.704 |
| *Quercus ilex* | 9.415 | 39.665 |
| *Quercus ilex* | 16.564 | 38.982 |
| *Quercus ilex* | -4.480 | 41.611 |
| *Quercus ilex* | 16.051 | 40.530 |
| *Quercus ilex* | 18.863 | 42.282 |
| *Quercus ilex* | 9.496 | 40.928 |
| *Quercus ilex* | 17.147 | 40.916 |
| *Quercus ilex* | -3.605 | 40.021 |
| *Quercus ilex* | 10.527 | 43.249 |
| *Quercus ilex* | 18.035 | 40.178 |
| *Quercus ilex* | -0.748 | 47.007 |
| *Quercus ilex* | 16.298 | 38.267 |
| *Quercus ilex* | 16.423 | 41.280 |
| *Quercus ilex* | 14.319 | 41.918 |
| *Quercus ilex* | 13.185 | 43.063 |
| *Quercus ilex* | 17.301 | 40.467 |
| *Quercus ilex* | 12.185 | 42.737 |
| *Quercus ilex* | -3.822 | 40.293 |
| *Quercus ilex* | 0.153 | 42.382 |
| *Quercus ilex* | 16.548 | 43.519 |
| *Quercus ilex* | -4.113 | 40.874 |
| *Quercus ilex* | 9.691 | 39.988 |
| *Quercus ilex* | 13.056 | 43.518 |
| *Quercus ilex* | -0.103 | 40.335 |
| *Quercus ilex* | -6.382 | 39.473 |
| *Quercus ilex* | 12.970 | 41.369 |
| *Quercus ilex* | 12.260 | 45.507 |
| *Quercus ilex* | 8.065 | 43.926 |
| *Quercus ilex* | -2.049 | 47.515 |
| *Quercus ilex* | 0.165 | 40.484 |
| *Quercus ilex* | 11.111 | 43.901 |
| *Quercus ilex* | 14.475 | 41.485 |
| *Quercus ilex* | -1.795 | 40.842 |
| *Quercus ilex* | 12.650 | 41.798 |
| *Quercus ilex* | 9.088 | 39.291 |
| *Quercus ilex* | 15.286 | 37.068 |
| *Quercus ilex* | 9.307 | 39.216 |
| *Quercus ilex* | -0.511 | 39.755 |
| *Quercus ilex* | -1.313 | 41.056 |
| *Quercus ilex* | -0.438 | 39.157 |
| *Quercus ilex* | 19.244 | 42.446 |
| *Quercus ilex* | -4.091 | 41.592 |
| *Quercus ilex* | 9.349 | 40.193 |
| *Quercus ilex* | 8.529 | 39.181 |
| *Quercus ilex* | 18.390 | 40.008 |
| *Quercus ilex* | 12.125 | 43.583 |
| *Quercus ilex* | -2.434 | 42.778 |
| *Quercus ilex* | -1.235 | 44.695 |
| *Quercus ilex* | -2.891 | 43.250 |
| *Quercus ilex* | -4.781 | 41.915 |
| *Quercus ilex* | 11.634 | 42.423 |
| *Quercus ilex* | -7.102 | 42.398 |
| *Quercus ilex* | 0.916 | 45.275 |
| *Quercus ilex* | 15.006 | 40.420 |
| *Quercus ilex* | 16.301 | 38.652 |
| *Quercus ilex* | 1.500 | 42.003 |
| *Quercus ilex* | 23.444 | 40.379 |
| *Quercus ilex* | 8.513 | 39.417 |
| *Quercus ilex* | 16.490 | 39.666 |
| *Quercus ilex* | 14.448 | 41.150 |
| *Quercus ilex* | -4.461 | 40.782 |
| *Quercus ilex* | -3.641 | 37.160 |
| *Quercus ilex* | -2.236 | 37.710 |
| *Quercus ilex* | -0.932 | 46.890 |
| *Quercus ilex* | 21.635 | 39.713 |
| *Quercus ilex* | -2.368 | 41.028 |
| *Quercus ilex* | 12.936 | 43.673 |
| *Quercus ilex* | -1.804 | 41.839 |
| *Quercus ilex* | -1.195 | 42.271 |
| *Quercus ilex* | 1.333 | 41.178 |
| *Quercus ilex* | 16.692 | 41.109 |
| *Quercus ilex* | 1.084 | 41.661 |
| *Quercus ilex* | 13.278 | 45.823 |
| *Quercus ilex* | -1.420 | 46.962 |
| *Quercus ilex* | 13.839 | 42.694 |
| *Quercus ilex* | -0.727 | 42.527 |
| *Quercus ilex* | 14.534 | 45.123 |
| *Quercus ilex* | -6.311 | 37.982 |
| *Quercus ilex* | 12.357 | 42.347 |
| *Quercus ilex* | -2.559 | 42.303 |
| *Quercus ilex* | -3.702 | 42.329 |
| *Quercus ilex* | 14.288 | 37.270 |
| *Quercus ilex* | -4.321 | 40.369 |
| *Quercus ilex* | 16.295 | 41.115 |
| *Quercus ilex* | 13.684 | 43.296 |
| *Quercus ilex* | 12.287 | 43.016 |
| *Quercus ilex* | 8.921 | 39.441 |
| *Quercus ilex* | -5.928 | 42.304 |
| *Quercus ilex* | 13.254 | 43.696 |
| *Quercus ilex* | 3.915 | 43.570 |
| *Quercus ilex* | 16.283 | 40.403 |
| *Quercus ilex* | -7.181 | 40.123 |
| *Quercus ilex* | 16.479 | 41.115 |
| *Quercus ilex* | 2.085 | 41.818 |
| *Quercus ilex* | 13.895 | 40.731 |
| *Quercus ilex* | 12.770 | 43.698 |
| *Quercus ilex* | 1.645 | 43.592 |
| *Quercus ilex* | 1.738 | 41.396 |
| *Quercus ilex* | -5.156 | 36.878 |
| *Quercus ilex* | -1.171 | 40.351 |
| *Quercus ilex* | -5.685 | 42.000 |
| *Quercus ilex* | 12.572 | 44.061 |
| *Quercus ilex* | 12.985 | 41.550 |
| *Quercus ilex* | 1.457 | 41.328 |
| *Quercus ilex* | -1.095 | 41.718 |
| *Quercus ilex* | -5.261 | 36.596 |
| *Quercus ilex* | 14.564 | 36.881 |
| *Quercus ilex* | 15.630 | 41.496 |
| *Quercus ilex* | -5.044 | 38.968 |
| *Quercus ilex* | -5.045 | 41.883 |
| *Quercus ilex* | 8.721 | 39.463 |
| *Quercus ilex* | -0.402 | 42.145 |
| *Quercus ilex* | 15.796 | 40.649 |
| *Quercus ilex* | 20.675 | 38.421 |
| *Quercus ilex* | -6.345 | 38.560 |
| *Quercus ilex* | 1.497 | 44.277 |
| *Quercus ilex* | 0.183 | 41.902 |
| *Quercus ilex* | -2.497 | 41.993 |
| *Quercus ilex* | 3.053 | 41.800 |
| *Quercus ilex* | 12.408 | 42.784 |
| *Quercus ilex* | 14.843 | 40.708 |
| *Quercus ilex* | 14.805 | 40.914 |
| *Quercus ilex* | -2.141 | 40.073 |
| *Quercus ilex* | 15.474 | 40.987 |
| *Quercus ilex* | 2.983 | 39.693 |
| *Quercus ilex* | 25.656 | 35.178 |
| *Quercus ilex* | -4.302 | 43.226 |
| *Quercus ilex* | -0.044 | 40.802 |
| *Quercus ilex* | -5.876 | 38.183 |
| *Quercus ilex* | -0.189 | 38.962 |
| *Quercus ilex* | -1.507 | 46.725 |
| *Quercus ilex* | 8.707 | 39.046 |
| *Quercus ilex* | 17.707 | 40.656 |
| *Quercus ilex* | -3.611 | 42.026 |
| *Quercus ilex* | 12.836 | 43.499 |
| *Quercus ilex* | -5.338 | 42.771 |
| *Quercus ilex* | 13.551 | 37.976 |
| *Quercus ilex* | 13.012 | 37.681 |
| *Quercus ilex* | 17.918 | 40.449 |
| *Quercus ilex* | 14.244 | 37.609 |
| *Quercus ilex* | 13.474 | 42.306 |
| *Quercus ilex* | -6.725 | 42.600 |
| *Quercus ilex* | 15.650 | 40.334 |
| *Quercus ilex* | -5.516 | 40.828 |
| *Quercus ilex* | -5.538 | 36.756 |
| *Quercus ilex* | 19.175 | 42.209 |
| *Quercus ilex* | 16.687 | 40.709 |
| *Quercus ilex* | -7.261 | 40.967 |
| *Quercus ilex* | -3.953 | 41.410 |
| *Quercus ilex* | -4.787 | 37.052 |
| *Quercus ilex* | 13.654 | 41.286 |
| *Quercus ilex* | 0.821 | 41.787 |
| *Quercus ilex* | 12.999 | 45.631 |
| *Quercus ilex* | 0.171 | 42.223 |
| *Quercus ilex* | 9.595 | 39.398 |
| *Quercus ilex* | 14.729 | 42.093 |
| *Quercus ilex* | -0.920 | 41.618 |
| *Quercus ilex* | -0.198 | 38.677 |
| *Quercus ilex* | 16.759 | 40.564 |
| *Quercus ilex* | -3.662 | 43.034 |
| *Quercus ilex* | 1.284 | 41.792 |
| *Quercus ilex* | 14.465 | 41.656 |
| *Quercus ilex* | -4.990 | 36.496 |
| *Quercus ilex* | 12.092 | 42.346 |
| *Quercus ilex* | -0.087 | 40.598 |
| *Quercus ilex* | -5.818 | 41.709 |
| *Quercus ilex* | -3.730 | 40.950 |
| *Quercus ilex* | -4.658 | 40.654 |
| *Quercus ilex* | -4.789 | 42.283 |
| *Quercus ilex* | 4.192 | 43.567 |
| *Quercus ilex* | 14.961 | 41.689 |
| *Quercus ilex* | 16.123 | 38.082 |
| *Quercus ilex* | -4.550 | 41.975 |
| *Quercus ilex* | -0.819 | 42.153 |
| *Quercus ilex* | 13.994 | 40.970 |
| *Quercus ilex* | 12.134 | 42.160 |
| *Quercus ilex* | -7.193 | 37.975 |
| *Quercus ilex* | 4.606 | 45.843 |
| *Quercus ilex* | 0.639 | 41.623 |
| *Quercus ilex* | 2.643 | 41.790 |
| *Quercus ilex* | 0.307 | 41.066 |
| *Quercus ilex* | -0.948 | 47.027 |
| *Quercus ilex* | -3.603 | 38.298 |
| *Quercus ilex* | -7.045 | 38.583 |
| *Quercus ilex* | -4.868 | 39.097 |
| *Quercus ilex* | -3.515 | 40.323 |
| *Quercus ilex* | 15.233 | 40.816 |
| *Quercus ilex* | -0.422 | 40.112 |
| *Quercus ilex* | -5.068 | 40.159 |
| *Quercus ilex* | -3.520 | 41.359 |
| *Quercus ilex* | -7.393 | 38.439 |
| *Quercus ilex* | -0.526 | 42.687 |
| *Quercus ilex* | -5.809 | 42.649 |
| *Quercus ilex* | -3.245 | 43.397 |
| *Quercus ilex* | -1.014 | 39.466 |
| *Quercus ilex* | -4.073 | 41.046 |
| *Quercus ilex* | 14.426 | 40.793 |
| *Quercus ilex* | -2.991 | 41.829 |
| *Quercus ilex* | -7.510 | 40.293 |
| *Quercus ilex* | -3.409 | 38.023 |
| *Quercus ilex* | -3.752 | 42.775 |
| *Quercus ilex* | 0.952 | 41.519 |
| *Quercus ilex* | -2.091 | 41.784 |
| *Quercus ilex* | -2.115 | 42.084 |
| *Quercus ilex* | -4.420 | 41.830 |
| *Quercus ilex* | 3.004 | 41.977 |
| *Quercus ilex* | 0.141 | 42.597 |
| *Quercus ilex* | -5.196 | 41.056 |
| *Quercus ilex* | -4.112 | 41.848 |
| *Quercus ilex* | -1.974 | 43.266 |
| *Quercus ilex* | 0.833 | 43.429 |
| *Quercus ilex* | -2.386 | 36.835 |
| *Quercus ilex* | 1.115 | 41.081 |
| *Quercus ilex* | -4.961 | 41.145 |
| *Quercus ilex* | -3.163 | 41.851 |
| *Quercus ilex* | 14.559 | 41.284 |
| *Quercus ilex* | 16.818 | 43.336 |
| *Quercus ilex* | -0.175 | 42.218 |
| *Quercus ilex* | 8.748 | 39.667 |
| *Quercus ilex* | 1.263 | 42.153 |
| *Quercus ilex* | 12.452 | 43.930 |
| *Quercus ilex* | 9.835 | 43.040 |
| *Quercus ilex* | 15.341 | 41.250 |
| *Quercus ilex* | 1.231 | 42.575 |
| *Quercus ilex* | 17.321 | 42.940 |
| *Quercus ilex* | -1.641 | 42.111 |
| *Quercus ilex* | -4.832 | 38.098 |
| *Quercus ilex* | -3.947 | 36.873 |
| *Quercus ilex* | -6.470 | 37.687 |
| *Quercus ilex* | -3.466 | 36.909 |
| *Quercus ilex* | -4.337 | 36.949 |
| *Quercus ilex* | -6.014 | 40.142 |
| *Quercus ilex* | -3.940 | 43.249 |
| *Quercus ilex* | 25.127 | 39.992 |
| *Quercus ilex* | -6.163 | 41.215 |
| *Quercus ilex* | -1.501 | 40.889 |
| *Quercus ilex* | -7.469 | 37.347 |
| *Quercus ilex* | -6.182 | 39.461 |
| *Quercus ilex* | 0.748 | 42.320 |
| *Quercus ilex* | 20.367 | 39.292 |
| *Quercus ilex* | -3.052 | 37.163 |
| *Quercus ilex* | -8.084 | 37.061 |
| *Quercus ilex* | -4.306 | 37.140 |
| *Quercus ilex* | 0.493 | 42.383 |
| *Quercus ilex* | 26.269 | 39.377 |
| *Quercus ilex* | -4.026 | 37.497 |
| *Quercus ilex* | -4.315 | 37.540 |
| *Quercus ilex* | -2.191 | 37.039 |
| *Quercus ilex* | 1.450 | 38.710 |
| *Quercus ilex* | 1.450 | 39.070 |
| *Quercus ilex* | -5.253 | 37.838 |
| *Quercus ilex* | -4.620 | 42.690 |
| *Quercus ilex* | 18.173 | 40.018 |
| *Quercus ilex* | 8.217 | 40.774 |
| *Quercus ilex* | 8.564 | 40.747 |
| *Quercus ilex* | 15.968 | 41.901 |
| *Quercus ilex* | 11.694 | 42.626 |
| *Quercus ilex* | -5.740 | 41.502 |
| *Quercus ilex* | -6.904 | 40.416 |
| *Quercus ilex* | -5.951 | 43.533 |
| *Quercus ilex* | 15.290 | 37.854 |
| *Quercus ilex* | -5.378 | 36.754 |
| *Quercus ilex* | -3.060 | 40.579 |
| *Quercus ilex* | 16.911 | 41.064 |
| *Quercus ilex* | -7.579 | 39.679 |
| *Quercus ilex* | -3.192 | 42.419 |
| *Quercus ilex* | 14.438 | 37.764 |
| *Quercus ilex* | 22.728 | 37.635 |
| *Quercus ilex* | -1.866 | 38.103 |
| *Quercus ilex* | -4.530 | 42.833 |
| *Quercus ilex* | -6.410 | 38.942 |
| *Quercus ilex* | 1.085 | 42.114 |
| *Quercus ilex* | -0.893 | 39.655 |
| *Quercus ilex* | -0.773 | 39.238 |
| *Quercus ilex* | -2.723 | 41.480 |
| *Quercus ilex* | -5.266 | 42.489 |
| *Quercus ilex* | -6.180 | 40.786 |
| *Quercus ilex* | 8.530 | 39.929 |
| *Quercus ilex* | 1.292 | 41.360 |
| *Quercus ilex* | 13.600 | 37.301 |
| *Quercus ilex* | 11.707 | 43.743 |
| *Quercus ilex* | 9.256 | 41.005 |
| *Quercus ilex* | 11.112 | 42.410 |
| *Quercus ilex* | -3.775 | 40.652 |
| *Quercus ilex* | -0.282 | 47.201 |
| *Quercus ilex* | 11.868 | 42.991 |
| *Quercus ilex* | -6.895 | 40.186 |
| *Quercus ilex* | 17.642 | 40.498 |
| *Quercus ilex* | -4.695 | 41.468 |
| *Quercus ilex* | 0.557 | 41.881 |
| *Quercus ilex* | -5.814 | 43.127 |
| *Quercus ilex* | -1.977 | 39.436 |
| *Quercus ilex* | -2.467 | 41.757 |
| *Quercus ilex* | 0.151 | 40.910 |
| *Quercus ilex* | -2.990 | 38.038 |
| *Quercus ilex* | -3.610 | 37.771 |
| *Quercus ilex* | -3.483 | 41.629 |
| *Quercus ilex* | 12.799 | 37.826 |
| *Quercus ilex* | 15.707 | 41.708 |
| *Quercus ilex* | 14.443 | 35.886 |
| *Quercus ilex* | -5.176 | 37.017 |
| *Quercus ilex* | -2.039 | 42.236 |
| *Quercus ilex* | -3.839 | 43.423 |
| *Quercus ilex* | 1.961 | 42.818 |
| *Quercus ilex* | -0.360 | 47.641 |
| *Quercus ilex* | -5.091 | 38.457 |
| *Quercus ilex* | 17.307 | 40.875 |
| *Quercus ilex* | -8.317 | 37.115 |
| *Quercus ilex* | -3.147 | 41.612 |
| *Quercus ilex* | -5.594 | 38.767 |
| *Quercus ilex* | 13.529 | 45.805 |
| *Quercus ilex* | 19.802 | 41.328 |
| *Quercus ilex* | 14.254 | 42.096 |
| *Quercus ilex* | 0.032 | 42.081 |
| *Quercus ilex* | 17.113 | 40.602 |
| *Quercus ilex* | 13.195 | 41.438 |
| *Quercus ilex* | 8.557 | 40.475 |
| *Quercus ilex* | 17.401 | 43.079 |
| *Quercus ilex* | -4.731 | 36.905 |
| *Quercus ilex* | -6.964 | 41.720 |
| *Quercus ilex* | 13.709 | 43.159 |
| *Quercus ilex* | 9.377 | 41.180 |
| *Quercus ilex* | 15.330 | 41.512 |
| *Quercus ilex* | -6.626 | 39.173 |
| *Quercus ilex* | 8.555 | 40.213 |
| *Quercus ilex* | 4.674 | 45.272 |
| *Quercus ilex* | 14.251 | 41.365 |
| *Quercus ilex* | -2.459 | 37.836 |
| *Quercus ilex* | -3.483 | 42.933 |
| *Quercus ilex* | -8.036 | 37.678 |
| *Quercus ilex* | -7.449 | 37.963 |
| *Quercus ilex* | -4.183 | 42.941 |
| *Quercus ilex* | 9.785 | 40.552 |
| *Quercus ilex* | 9.366 | 40.403 |
| *Quercus ilex* | 9.169 | 40.853 |
| *Quercus ilex* | 16.669 | 40.217 |
| *Quercus ilex* | -2.933 | 37.537 |
| *Quercus ilex* | -8.500 | 39.662 |
| *Quercus ilex* | -7.077 | 41.033 |
| *Quercus ilex* | 17.649 | 43.059 |
| *Quercus ilex* | -4.706 | 37.401 |
| *Quercus ilex* | -4.310 | 41.401 |
| *Quercus ilex* | -2.121 | 42.787 |
| *Quercus ilex* | 1.776 | 42.024 |
| *Quercus ilex* | -4.140 | 42.289 |
| *Quercus ilex* | -3.568 | 41.192 |
| *Quercus ilex* | -5.179 | 40.360 |
| *Quercus ilex* | -5.464 | 40.112 |
| *Quercus ilex* | 8.772 | 40.174 |
| *Quercus ilex* | 2.127 | 43.650 |
| *Quercus ilex* | 13.886 | 42.364 |
| *Quercus ilex* | -0.924 | 42.377 |
| *Quercus ilex* | -2.353 | 37.983 |
| *Quercus ilex* | -5.032 | 42.091 |
| *Quercus ilex* | -6.520 | 42.626 |
| *Quercus ilex* | 24.670 | 35.324 |
| *Quercus ilex* | -0.236 | 39.868 |
| *Quercus ilex* | -6.174 | 37.732 |
| *Quercus ilex* | -1.170 | 46.849 |
| *Quercus ilex* | -8.433 | 38.405 |
| *Quercus ilex* | 16.736 | 39.244 |
| *Quercus ilex* | -1.530 | 41.830 |
| *Quercus ilex* | 15.116 | 37.309 |
| *Quercus ilex* | 17.942 | 40.622 |
| *Quercus ilex* | -1.043 | 38.409 |
| *Quercus ilex* | 14.444 | 42.068 |
| *Quercus ilex* | -5.782 | 41.331 |
| *Quercus ilex* | -1.794 | 41.272 |
| *Quercus ilex* | -5.430 | 36.965 |
| *Quercus ilex* | 13.703 | 41.550 |
| *Quercus ilex* | 12.871 | 42.226 |
| *Quercus ilex* | -8.013 | 38.537 |
| *Quercus ilex* | 11.827 | 43.149 |
| *Quercus ilex* | -4.560 | 40.286 |
| *Quercus ilex* | -1.242 | 46.537 |
| *Quercus ilex* | 16.202 | 43.024 |
| *Quercus ilex* | 20.765 | 38.163 |
| *Quercus ilex* | 0.576 | 40.945 |
| *Quercus ilex* | 13.698 | 42.326 |
| *Quercus ilex* | 1.050 | 42.304 |
| *Quercus ilex* | 0.009 | 38.864 |
| *Quercus ilex* | 14.597 | 41.032 |
| *Quercus ilex* | -2.848 | 36.861 |
| *Quercus ilex* | 23.664 | 35.412 |
| *Quercus ilex* | 12.950 | 42.386 |
| *Quercus ilex* | 13.531 | 43.104 |
| *Quercus ilex* | -5.613 | 42.741 |
| *Quercus ilex* | -1.518 | 41.088 |
| *Quercus ilex* | 0.258 | 42.051 |
| *Quercus ilex* | -5.223 | 42.266 |
| *Quercus ilex* | -3.252 | 40.654 |
| *Quercus ilex* | -3.487 | 41.825 |
| *Quercus ilex* | -5.036 | 37.831 |
| *Quercus ilex* | 7.242 | 43.690 |
| *Quercus ilex* | 13.494 | 42.881 |
| *Quercus ilex* | 13.437 | 42.003 |
| *Quercus ilex* | -6.136 | 42.401 |
| *Quercus ilex* | 12.433 | 37.802 |
| *Quercus ilex* | 5.324 | 44.783 |
| *Quercus ilex* | 17.325 | 43.468 |
| *Quercus ilex* | 12.583 | 43.337 |
| *Quercus ilex* | 14.994 | 36.903 |
| *Quercus ilex* | -6.949 | 37.971 |
| *Quercus ilex* | 0.726 | 40.722 |
| *Quercus ilex* | -5.978 | 37.703 |
| *Quercus ilex* | 5.317 | 44.369 |
| *Quercus ilex* | 8.108 | 44.132 |
| *Quercus ilex* | 23.363 | 38.834 |
| *Quercus ilex* | -4.490 | 41.154 |
| *Quercus ilex* | 1.096 | 41.977 |
| *Quercus ilex* | 12.648 | 42.156 |
| *Quercus ilex* | -0.873 | 47.518 |
| *Quercus ilex* | 13.747 | 41.689 |
| *Quercus ilex* | -4.657 | 39.566 |
| *Quercus ilex* | -4.909 | 41.304 |
| *Quercus ilex* | 15.802 | 41.106 |
| *Quercus ilex* | -5.891 | 40.765 |
| *Quercus ilex* | -8.633 | 38.757 |
| *Quercus ilex* | 16.067 | 41.302 |
| *Quercus ilex* | -3.664 | 37.300 |
| *Quercus ilex* | 11.960 | 42.513 |
| *Quercus ilex* | -6.396 | 41.286 |
| *Quercus ilex* | -6.197 | 41.531 |
| *Quercus ilex* | 11.099 | 42.606 |
| *Quercus ilex* | -2.043 | 37.563 |
| *Quercus ilex* | -2.266 | 37.232 |
| *Quercus ilex* | -0.741 | 39.983 |
| *Quercus ilex* | -2.289 | 40.558 |
| *Quercus ilex* | 13.889 | 41.303 |
| *Quercus ilex* | 14.183 | 41.791 |
| *Quercus ilex* | -0.416 | 38.780 |
| *Quercus ilex* | -6.416 | 38.415 |
| *Quercus ilex* | -4.033 | 39.871 |
| *Quercus ilex* | -0.354 | 42.490 |
| *Quercus ilex* | -4.686 | 41.286 |
| *Quercus ilex* | -3.866 | 41.113 |
| *Quercus ilex* | -2.889 | 37.010 |
| *Quercus ilex* | -1.158 | 40.042 |
| *Quercus ilex* | 15.544 | 40.154 |
| *Quercus ilex* | 13.431 | 41.355 |
| *Quercus ilex* | -0.569 | 39.543 |
| *Quercus ilex* | -6.646 | 38.208 |
| *Quercus ilex* | -4.499 | 37.288 |
| *Quercus ilex* | -0.835 | 40.133 |
| *Quercus ilex* | -3.002 | 37.394 |
| *Quercus ilex* | 21.649 | 37.481 |
| *Quercus ilex* | -1.522 | 37.868 |
| *Quercus ilex* | 15.941 | 39.897 |
| *Quercus ilex* | -5.655 | 40.129 |
| *Quercus ilex* | -3.354 | 39.374 |
| *Quercus ilex* | -6.124 | 37.917 |
| *Quercus ilex* | 5.008 | 45.517 |
| *Quercus ilex* | 2.694 | 39.554 |
| *Quercus ilex* | 24.151 | 41.151 |
| *Quercus ilex* | 13.698 | 42.945 |
| *Quercus ilex* | 21.947 | 38.301 |
| *Quercus ilex* | -6.434 | 41.012 |
| *Quercus ilex* | 10.917 | 43.956 |
| *Quercus ilex* | -7.751 | 38.830 |
| *Quercus ilex* | -4.368 | 38.668 |
| *Quercus ilex* | 12.462 | 42.977 |
| *Quercus ilex* | -5.705 | 40.696 |
| *Quercus ilex* | 14.778 | 38.070 |
| *Quercus ilex* | -2.790 | 41.173 |
| *Quercus ilex* | -7.096 | 39.435 |
| *Quercus ilex* | 13.236 | 43.320 |
| *Quercus ilex* | -0.585 | 38.619 |
| *Quercus ilex* | 4.684 | 43.754 |
| *Quercus ilex* | -3.192 | 39.737 |
| *Quercus ilex* | -1.625 | 41.539 |
| *Quercus ilex* | -4.522 | 41.409 |
| *Quercus ilex* | 21.128 | 39.499 |
| *Quercus ilex* | -2.281 | 41.164 |
| *Quercus ilex* | 13.543 | 37.479 |
| *Quercus ilex* | -0.468 | 38.940 |
| *Quercus ilex* | -3.913 | 40.023 |
| *Quercus ilex* | -6.819 | 41.562 |
| *Quercus ilex* | -5.464 | 40.328 |
| *Quercus ilex* | -5.755 | 37.927 |
| *Quercus ilex* | -6.912 | 40.707 |
| *Quercus ilex* | -3.619 | 38.136 |
| *Quercus ilex* | -0.794 | 40.272 |
| *Quercus ilex* | 12.133 | 44.475 |
| *Quercus ilex* | -0.695 | 39.778 |
| *Quercus ilex* | 22.695 | 37.807 |
| *Quercus ilex* | 22.885 | 39.683 |
| *Quercus ilex* | 11.305 | 43.859 |
| *Quercus ilex* | -4.255 | 43.384 |
| *Quercus ilex* | -6.677 | 41.216 |
| *Quercus ilex* | -4.276 | 41.630 |
| *Quercus ilex* | -3.250 | 37.192 |
| *Quercus ilex* | -7.396 | 39.634 |
| *Quercus ilex* | -0.386 | 40.776 |
| *Quercus ilex* | 16.494 | 40.249 |
| *Quercus ilex* | -0.738 | 41.741 |
| *Quercus ilex* | -4.523 | 38.162 |
| *Quercus ilex* | 0.512 | 45.029 |
| *Quercus ilex* | 9.972 | 44.210 |
| *Quercus ilex* | -6.619 | 37.414 |
| *Quercus ilex* | -1.227 | 41.336 |
| *Quercus ilex* | -4.321 | 38.254 |
| *Quercus ilex* | -4.158 | 36.979 |
| *Quercus ilex* | -5.132 | 43.352 |
| *Quercus ilex* | 12.730 | 41.540 |
| *Quercus ilex* | -5.792 | 39.152 |
| *Quercus ilex* | -4.259 | 42.794 |
| *Quercus ilex* | 14.067 | 42.603 |
| *Quercus ilex* | 16.090 | 40.969 |
| *Quercus ilex* | 15.343 | 44.281 |
| *Quercus ilex* | -5.419 | 38.262 |
| *Quercus ilex* | -3.245 | 41.130 |
| *Quercus ilex* | -7.102 | 37.440 |
| *Quercus ilex* | -6.671 | 41.001 |
| *Quercus ilex* | 14.278 | 37.127 |
| *Quercus ilex* | -5.730 | 40.400 |
| *Quercus ilex* | -2.731 | 42.419 |
| *Quercus ilex* | -2.433 | 37.655 |
| *Quercus ilex* | -3.460 | 37.317 |
| *Quercus ilex* | -5.952 | 37.981 |
| *Quercus ilex* | -5.435 | 41.530 |
| *Quercus ilex* | 16.404 | 40.530 |
| *Quercus ilex* | -5.606 | 42.394 |
| *Quercus ilex* | -3.902 | 41.760 |
| *Quercus ilex* | -6.712 | 37.899 |
| *Quercus ilex* | 0.087 | 40.274 |
| *Quercus ilex* | -3.230 | 37.500 |
| *Quercus ilex* | 16.522 | 39.861 |
| *Quercus ilex* | -4.074 | 37.179 |
| *Quercus ilex* | -6.460 | 37.843 |
| *Quercus ilex* | -0.348 | 39.716 |
| *Quercus ilex* | -2.899 | 42.151 |
| *Quercus ilex* | -4.639 | 36.554 |
| *Quercus ilex* | 16.416 | 40.816 |
| *Quercus ilex* | -0.413 | 39.297 |
| *Quercus ilex* | -6.104 | 38.593 |
| *Quercus ilex* | 16.923 | 40.793 |
| *Quercus ilex* | -6.527 | 38.587 |
| *Quercus ilex* | -8.659 | 37.143 |
| *Quercus ilex* | -4.556 | 40.431 |
| *Quercus ilex* | -2.464 | 42.604 |
| *Quercus ilex* | -5.679 | 38.260 |
| *Quercus ilex* | 22.382 | 39.627 |
| *Quercus ilex* | 18.362 | 39.846 |
| *Quercus ilex* | 8.872 | 39.009 |
| *Quercus ilex* | 16.057 | 40.751 |
| *Quercus ilex* | 23.163 | 38.975 |
| *Quercus ilex* | -3.318 | 41.290 |
| *Quercus ilex* | 9.056 | 39.856 |
| *Quercus ilex* | -7.839 | 38.206 |
| *Quercus ilex* | 3.093 | 42.340 |
| *Quercus ilex* | -6.847 | 39.063 |
| *Quercus ilex* | -8.230 | 38.809 |
| *Quercus ilex* | 13.334 | 43.489 |
| *Quercus ilex* | -3.686 | 41.671 |
| *Quercus ilex* | -8.747 | 37.419 |
| *Quercus ilex* | -3.392 | 38.752 |
| *Quercus ilex* | -1.161 | 38.873 |
| *Quercus ilex* | 16.378 | 43.552 |
| *Quercus ilex* | 9.317 | 40.568 |
| *Quercus ilex* | -0.033 | 38.640 |
| *Quercus ilex* | -4.293 | 36.763 |
| *Quercus ilex* | 5.904 | 44.342 |
| *Quercus ilex* | 23.784 | 40.592 |
| *Quercus ilex* | 26.083 | 37.604 |
| *Quercus ilex* | 10.650 | 43.599 |
| *Quercus ilex* | 14.987 | 37.844 |
| *Quercus ilex* | -1.793 | 40.583 |
| *Quercus ilex* | -3.552 | 40.925 |
| *Quercus ilex* | 12.717 | 42.552 |
| *Quercus ilex* | -8.020 | 38.776 |
| *Quercus ilex* | 20.535 | 39.236 |
| *Quercus ilex* | 15.020 | 37.469 |
| *Quercus ilex* | -3.700 | 36.831 |
| *Quercus ilex* | -8.539 | 37.484 |
| *Quercus ilex* | -5.903 | 41.072 |
| *Quercus ilex* | 9.380 | 39.522 |
| *Quercus ilex* | -4.751 | 40.482 |
| *Quercus ilex* | -2.661 | 37.853 |
| *Quercus ilex* | 10.857 | 43.547 |
| *Quercus ilex* | 12.577 | 45.670 |
| *Quercus ilex* | -7.842 | 37.153 |
| *Quercus ilex* | -1.799 | 42.338 |
| *Quercus ilex* | 14.706 | 41.690 |
| *Quercus ilex* | -7.066 | 42.188 |
| *Quercus ilex* | 13.633 | 42.583 |
| *Quercus ilex* | 18.767 | 42.426 |
| *Quercus ilex* | -6.452 | 40.872 |
| *Quercus ilex* | -4.730 | 40.293 |
| *Quercus ilex* | 22.095 | 37.122 |
| *Quercus ilex* | 8.753 | 40.710 |
| *Quercus ilex* | 13.766 | 45.235 |
| *Quercus ilex* | 9.604 | 40.535 |
| *Quercus ilex* | -5.942 | 39.316 |
| *Quercus ilex* | 16.594 | 38.807 |
| *Quercus ilex* | 24.920 | 37.420 |
| *Quercus ilex* | -6.512 | 42.783 |
| *Quercus ilex* | 10.952 | 42.749 |
| *Quercus ilex* | -0.025 | 47.718 |
| *Quercus ilex* | -6.059 | 40.638 |
| *Quercus ilex* | -6.580 | 40.225 |
| *Quercus ilex* | 5.081 | 44.652 |
| *Quercus ilex* | -0.077 | 41.846 |
| *Quercus ilex* | 12.619 | 41.975 |
| *Quercus ilex* | -6.006 | 43.256 |
| *Quercus ilex* | 11.334 | 42.688 |
| *Quercus ilex* | 12.385 | 44.071 |
| *Quercus ilex* | 13.129 | 42.784 |
| *Quercus ilex* | -5.983 | 41.537 |
| *Quercus ilex* | -8.667 | 37.711 |
| *Quercus ilex* | 25.358 | 35.335 |
| *Quercus ilex* | -6.100 | 39.039 |
| *Quercus ilex* | 19.500 | 40.473 |
| *Quercus ilex* | -5.466 | 42.115 |
| *Quercus ilex* | -6.962 | 40.891 |
| *Quercus ilex* | -7.472 | 38.996 |
| *Quercus ilex* | -2.002 | 40.287 |
| *Quercus ilex* | -8.000 | 39.530 |
| *Quercus ilex* | -5.448 | 37.129 |
| *Quercus ilex* | 15.681 | 40.836 |
| *Quercus ilex* | -8.103 | 38.185 |
| *Quercus ilex* | -1.544 | 40.323 |
| *Quercus ilex* | -6.066 | 39.974 |
| *Quercus ilex* | 16.216 | 39.043 |
| *Quercus ilex* | -6.303 | 39.243 |
| *Quercus ilex* | -2.513 | 41.493 |
| *Quercus ilex* | -3.957 | 37.337 |
| *Quercus ilex* | 16.052 | 40.240 |
| *Quercus ilex* | -2.157 | 38.548 |
| *Quercus ilex* | 25.865 | 40.856 |
| *Quercus ilex* | 12.802 | 43.871 |
| *Quercus ilex* | -6.325 | 40.604 |
| *Quercus ilex* | 22.946 | 39.360 |
| *Quercus ilex* | -7.508 | 39.430 |
| *Quercus ilex* | -4.673 | 38.442 |
| *Quercus ilex* | -5.788 | 42.882 |
| *Quercus ilex* | -5.966 | 41.866 |
| *Quercus ilex* | -6.776 | 39.616 |
| *Quercus ilex* | -4.104 | 38.067 |
| *Quercus ilex* | -2.915 | 37.759 |
| *Quercus ilex* | 14.944 | 36.712 |
| *Quercus ilex* | 11.903 | 42.154 |
| *Quercus ilex* | -6.415 | 43.292 |
| *Quercus ilex* | -0.786 | 38.483 |
| *Quercus ilex* | -6.265 | 42.603 |
| *Quercus ilex* | -0.804 | 39.491 |
| *Quercus ilex* | -6.769 | 38.354 |
| *Quercus ilex* | -8.231 | 37.653 |
| *Quercus ilex* | -7.753 | 39.060 |
| *Quercus ilex* | -0.217 | 38.518 |
| *Quercus ilex* | -5.272 | 40.112 |
| *Quercus ilex* | -5.225 | 36.403 |
| *Quercus ilex* | 0.237 | 41.636 |
| *Quercus ilex* | 13.062 | 37.506 |
| *Quercus ilex* | -6.419 | 41.865 |
| *Quercus ilex* | 24.764 | 35.023 |
| *Quercus ilex* | -0.161 | 47.356 |
| *Quercus ilex* | 14.470 | 45.316 |
| *Quercus ilex* | 12.491 | 44.954 |
| *Quercus ilex* | -6.102 | 40.458 |
| *Quercus ilex* | 16.522 | 39.248 |
| *Quercus ilex* | 25.431 | 37.028 |
| *Quercus ilex* | -7.467 | 40.014 |
| *Quercus ilex* | 13.940 | 41.774 |
| *Quercus ilex* | -1.677 | 38.024 |
| *Quercus ilex* | 16.265 | 38.812 |
| *Quercus ilex* | 12.731 | 42.890 |
| *Quercus ilex* | -2.630 | 41.689 |
| *Quercus ilex* | 15.574 | 41.908 |
| *Quercus ilex* | 13.037 | 42.925 |
| *Quercus ilex* | 22.891 | 38.341 |
| *Quercus ilex* | 6.677 | 43.962 |
| *Quercus ilex* | 4.904 | 43.456 |
| *Quercus ilex* | 4.464 | 43.504 |
| *Quercus ilex* | 7.075 | 44.188 |
| *Quercus ilex* | 4.941 | 44.814 |
| *Quercus ilex* | -5.884 | 39.531 |
| *Quercus ilex* | 8.833 | 35.167 |
| *Quercus ilex* | 1.384 | 45.204 |
| *Quercus ilex* | -0.481 | 46.034 |
| *Quercus ilex* | 1.168 | 44.107 |
| *Quercus ilex* | -1.679 | 47.363 |
| *Quercus ilex* | 0.702 | 45.436 |
| *Quercus ilex* | 0.485 | 45.300 |
| *Quercus ilex* | 24.204 | 35.406 |
| *Quercus ilex* | 24.483 | 35.367 |
| *Quercus ilex* | 24.250 | 40.250 |
| *Quercus ilex* | 22.200 | 38.200 |
| *Quercus ilex* | 14.250 | 37.750 |
| *Quercus ilex* | 2.833 | 36.467 |
| *Quercus ilex* | 9.000 | 34.000 |
| *Quercus ilex* | 15.731 | 40.102 |
| *Quercus ilex* | 26.233 | 39.127 |
| *Quercus ilex* | -5.464 | 36.428 |
| *Quercus ilex* | 9.265 | 39.849 |
| *Quercus ilex* | -7.529 | 37.534 |
| *Quercus ilex* | 19.918 | 39.441 |
| *Quercus ilex* | -5.679 | 39.305 |
| *Quercus ilex* | -4.450 | 42.990 |
| *Quercus ilex* | 8.504 | 40.613 |
| *Quercus ilex* | 23.600 | 40.260 |
| *Quercus ilex* | -8.244 | 37.925 |
| *Quercus ilex* | 24.680 | 41.090 |
| *Quercus ilex* | -3.297 | 38.357 |
| *Quercus ilex* | -4.953 | 36.844 |
| *Quercus ilex* | -6.648 | 37.730 |
| *Quercus ilex* | 9.345 | 39.353 |
| *Quercus ilex* | 22.406 | 36.964 |
| *Quercus ilex* | -5.645 | 35.702 |
| *Quercus ilex* | -4.622 | 34.907 |
| *Quercus ilex* | -5.882 | 35.787 |
| *Quercus ilex* | -3.442 | 34.566 |
| *Quercus ilex* | -5.217 | 35.161 |
| *Quercus ilex* | -5.695 | 41.160 |
| *Quercus ilex* | -2.404 | 36.992 |
| *Quercus ilex* | -6.483 | 32.150 |
| *Quercus ilex* | -8.300 | 30.900 |
| *Quercus ilex* | -3.233 | 36.783 |
| *Quercus ilex* | -5.455 | 32.677 |
| *Quercus ilex* | -6.309 | 32.097 |
| *Quercus ilex* | -5.033 | 33.033 |
| *Quercus ilex* | -4.460 | 42.441 |
| *Quercus ilex* | -4.917 | 40.433 |
| *Quercus ilex* | -2.785 | 38.063 |
| *Quercus ilex* | -7.166 | 42.850 |
| *Quercus ilex* | -9.280 | 29.756 |
| *Quercus ilex* | 8.680 | 35.205 |
| *Quercus ilex* | 8.792 | 36.288 |
| *Quercus ilex* | 8.254 | 41.045 |
| *Quercus ilex* | -6.474 | 31.576 |
| *Quercus ilex* | -5.210 | 33.560 |
| *Quercus ilex* | -3.835 | 34.767 |
| *Quercus ilex* | -5.020 | 35.150 |
| *Quercus ilex* | -4.185 | 34.090 |
| *Quercus ilex* | -8.100 | 31.154 |
| *Quercus ilex* | -3.584 | 35.017 |
| *Quercus ilex* | -10.034 | 29.203 |
| *Quercus ilex* | 24.200 | 38.247 |
| *Quercus ilex* | 21.339 | 40.104 |
| *Quercus ilex* | 23.621 | 41.181 |
| *Quercus ilex* | 22.342 | 40.852 |
| *Quercus ilex* | 21.749 | 38.232 |
| *Quercus ilex* | 8.400 | 39.042 |
| *Quercus ilex* | 15.227 | 38.809 |
| *Quercus ilex* | 10.087 | 36.365 |
| *Quercus ilex* | 28.055 | 36.093 |
| *Quercus ilex* | 11.043 | 37.070 |
| *Quercus ilex* | 6.780 | 35.363 |
| *Quercus ilex* | 10.316 | 42.330 |
| *Quercus ilex* | 11.101 | 42.253 |
| *Quercus ilex* | 10.075 | 42.581 |
| *Quercus ilex* | 9.899 | 43.431 |
| *Quercus ilex* | 8.714 | 39.820 |
| *Quercus ilex* | 8.465 | 39.566 |
| *Quercus ilex* | 9.590 | 39.710 |
| *Quercus ilex* | -4.086 | 34.307 |
| *Quercus ilex* | 27.833 | 36.600 |
| *Quercus ilex* | 26.267 | 37.633 |
| *Quercus ilex* | 24.883 | 37.850 |
| *Quercus ilex* | 24.700 | 40.767 |
| *Quercus ilex* | 24.117 | 39.100 |
| *Quercus ilex* | 25.317 | 36.717 |
| *Quercus ilex* | 24.600 | 38.833 |
| *Quercus ilex* | 24.750 | 37.967 |
| *Quercus ilex* | 20.733 | 39.967 |
| *Quercus ilex* | 24.617 | 38.133 |
| *Quercus ilex* | 26.017 | 35.133 |
| *Quercus ilex* | 25.217 | 37.583 |
| *Quercus ilex* | 7.796 | 44.002 |
| *Quercus ilex* | -0.270 | 40.280 |
| *Quercus ilex* | -0.410 | 39.960 |
| *Quercus ilex* | -0.250 | 39.180 |
| *Quercus ilex* | -1.070 | 39.870 |
| *Quercus ilex* | -1.090 | 39.060 |
| *Quercus ilex* | -1.410 | 39.510 |
| *Quercus ilex* | -0.270 | 40.430 |
| *Quercus ilex* | -0.630 | 40.620 |
| *Quercus ilex* | -0.250 | 40.570 |
| *Quercus ilex* | -0.940 | 39.230 |
| *Quercus ilex* | -0.220 | 40.740 |
| *Quercus ilex* | -1.360 | 40.020 |
| *Quercus ilex* | -0.660 | 38.930 |
| *Quercus ilex* | -0.400 | 38.520 |
| *Quercus ilex* | 1.465 | 36.457 |
| *Quercus ilex* | 3.896 | 36.378 |
| *Quercus ilex* | 10.419 | 36.576 |
| *Quercus ilex* | 3.243 | 43.931 |
| *Quercus ilex* | 0.390 | 40.790 |
| *Quercus ilex* | 0.740 | 40.890 |
| *Quercus ilex* | 2.630 | 42.260 |
| *Quercus ilex* | 1.920 | 41.270 |
| *Quercus ilex* | 2.150 | 42.260 |
| *Quercus ilex* | 1.310 | 41.620 |
| *Quercus ilex* | 1.410 | 42.430 |
| *Quercus ilex* | 0.490 | 41.160 |
| *Quercus ilex* | 0.140 | 41.060 |
| *Quercus ilex* | 0.600 | 41.340 |
| *Quercus ilex* | 1.300 | 41.980 |
| *Quercus ilex* | -2.011 | 37.727 |
| *Quercus ilex* | -4.300 | 34.883 |
| *Quercus ilex* | 24.371 | 38.081 |
| *Quercus ilex* | 0.409 | 40.929 |
| *Quercus ilex* | -8.281 | 37.279 |
| *Quercus ilex* | -1.311 | 39.719 |
| *Quercus ilex* | 1.249 | 38.899 |
| *Quercus ilex* | -5.821 | 37.729 |
| *Quercus ilex* | 4.289 | 39.829 |
| *Quercus ilex* | -0.911 | 41.309 |
| *Quercus ilex* | -0.071 | 41.289 |
| *Quercus ilex* | -3.181 | 42.229 |
| *Quercus ilex* | -7.911 | 31.229 |
| *Quercus ilex* | 8.559 | 36.349 |
| *Quercus ilex* | -4.781 | 43.109 |
| *Quercus ilex* | -7.421 | 42.819 |
| *Quercus ilex* | -4.101 | 39.429 |
| *Quercus ilex* | -3.751 | 40.809 |
| *Quercus ilex* | -0.161 | 42.009 |
| *Quercus ilex* | -6.731 | 39.409 |
| *Quercus ilex* | -6.321 | 38.139 |
| *Quercus ilex* | -6.871 | 38.689 |
| *Quercus ilex* | -1.057 | 47.555 |
| *Quercus ilex* | -0.798 | 47.654 |
| *Quercus ilex* | -2.093 | 47.244 |
| *Quercus ilex* | -0.698 | 46.215 |
| *Quercus ilex* | 3.564 | 44.293 |
| *Quercus ilex* | 4.713 | 45.001 |
| *Quercus ilex* | 4.602 | 45.543 |
| *Quercus ilex* | -3.080 | 33.990 |
| *Quercus ilex* | -5.190 | 33.410 |
| *Quercus ilex* | 25.550 | 40.500 |
| *Quercus ilex* | -1.740 | 41.680 |
| *Quercus ilex* | -0.860 | 42.750 |
| *Quercus ilex* | -8.098 | 39.801 |
| *Quercus ilex* | -3.608 | 37.564 |
| *Quercus ilex* | -3.043 | 42.617 |
| *Quercus ilex* | 24.088 | 35.218 |
| *Quercus ilex* | 6.255 | 35.490 |
| *Quercus ilex* | 1.621 | 35.879 |
| *Quercus ilex* | -4.839 | 33.829 |
| *Quercus ilex* | -4.854 | 33.664 |
| *Quercus ilex* | 3.084 | 36.731 |
| *Quercus ilex* | 2.717 | 35.911 |
| *Quercus ilex* | 0.147 | 35.387 |
| *Quercus ilex* | 22.561 | 39.875 |
| *Quercus ilex* | -8.570 | 37.319 |
| *Quercus ilex* | -8.589 | 38.183 |
| *Quercus ilex* | 23.459 | 37.500 |
| *Quercus ilex* | 9.270 | 35.436 |
| *Quercus ilex* | -1.330 | 34.636 |
| *Quercus ilex* | -0.621 | 34.676 |
| *Quercus ilex* | 0.917 | 34.934 |
| *Quercus ilex* | 5.733 | 34.850 |
| *Quercus ilex* | -1.254 | 34.886 |
| *Quercus ilex* | -8.457 | 31.050 |
| *Quercus ilex* | -5.441 | 32.348 |
| *Quercus ilex* | 4.292 | 36.481 |
| *Quercus ilex* | 6.649 | 43.261 |
| *Quercus ilex* | -0.633 | 35.697 |
| *Quercus ilex* | -6.343 | 34.100 |
| *Quercus ilex* | -3.454 | 37.123 |
| *Quercus ilex* | 2.876 | 36.681 |
| *Quercus ilex* | -4.297 | 33.472 |
| *Quercus ilex* | 7.755 | 36.902 |
| *Quercus ilex* | 2.897 | 36.147 |
| *Quercus ilex* | -5.080 | 39.370 |
| *Quercus ilex* | -4.140 | 38.390 |
| *Quercus ilex* | -3.580 | 39.380 |
| *Quercus ilex* | -6.930 | 42.340 |
| *Quercus ilex* | 3.110 | 39.290 |
| *Quercus ilex* | -0.180 | 40.070 |
| *Quercus ilex* | -6.950 | 37.740 |
| *Quercus ilex* | -0.920 | 38.830 |
| *Quercus ilex* | -5.580 | 43.230 |
| *Quercus ilex* | -7.420 | 37.210 |
| *Quercus ilex* | -4.120 | 36.770 |
| *Quercus ilex* | -7.300 | 42.250 |
| *Quercus ilex* | -4.850 | 39.460 |
| *Quercus ilex* | -1.840 | 39.110 |
| *Quercus ilex* | 14.000 | 37.500 |
| *Quercus ilex* | -2.821 | 41.689 |
| *Quercus ilex* | -1.981 | 41.499 |
| *Quercus ilex* | -2.221 | 41.409 |
| *Quercus ilex* | -2.221 | 41.589 |
| *Quercus ilex* | -0.091 | 41.019 |
| *Quercus ilex* | 1.849 | 42.399 |
| *Quercus ilex* | -2.031 | 38.169 |
| *Quercus ilex* | -7.111 | 42.659 |
| *Quercus ilex* | -4.031 | 42.039 |
| *Quercus ilex* | -5.031 | 43.199 |
| *Quercus ilex* | -4.771 | 42.839 |
| *Quercus ilex* | -6.391 | 42.379 |
| *Quercus ilex* | -3.311 | 43.039 |
| *Quercus ilex* | -5.121 | 42.919 |
| *Quercus ilex* | -6.231 | 43.269 |
| *Quercus ilex* | -6.131 | 41.869 |
| *Quercus ilex* | 35.188 | 42.021 |
| *Quercus ilex* | 31.405 | 41.296 |
| *Quercus ilex* | 31.345 | 41.143 |
| *Quercus ilex* | 27.126 | 37.689 |
| *Quercus ilex* | 27.311 | 37.705 |
| *Quercus ilex* | 27.569 | 36.733 |
| *Quercus ilex* | 28.798 | 41.167 |
| *Quercus ilex* | 29.119 | 41.074 |
| *Quercus ilex* | 25.867 | 40.164 |
| *Quercus ilex* | 26.599 | 38.204 |
| *Quercus ilex* | 36.330 | 41.319 |
