# Supplementary material for: Distribution patterns of Quercus ilex from the last interglacial period to the future by ecological niche modeling
Source: Ecol Evol. 2023 Oct 19;13(10):e10606. doi: 10.1002/ece3.10606 (PMC10585444; doi:10.1002/ece3.10606)
Supplement: Supplementary file 4 — Table S2. [file ECE3-13-e10606-s002.docx]

**S2 Table.** 19 bioclimatic variables obtained from WorldClim version 1.4.

| Abbreviations | Variable name | Unit | Original resouliton | Source |
| --- | --- | --- | --- | --- |
| Bio1 | Annual Mean Temperature | °C | 2.5 minutes | Worldclim |
| Bio2 | Mean Diurnal Range | °C | 2.5 minutes | Worldclim |
| Bio3 | Isothermality | Bio_2/Bio_7 | 2.5 minutes | Worldclim |
| Bio4 | Temperature Seasonality  (standard deviation *100) | °C | 2.5 minutes | Worldclim |
| Bio5 | Max Temperature of Warmest Month | °C | 2.5 minutes | Worldclim |
| Bio6 | Min Temperature of Coldest Month | °C | 2.5 minutes | Worldclim |
| Bio7 | Temperature Annual Range | °C | 2.5 minutes | Worldclim |
| Bio8 | Mean Temperature of Wettest Quarter | °C | 2.5 minutes | Worldclim |
| Bio9 | Mean Temperature of Driest Quarter | °C | 2.5 minutes | Worldclim |
| Bio10 | Mean Temperature of Warmest Quarter | °C | 2.5 minutes | Worldclim |
| Bio11 | Mean Temperature of Coldest Quarter | °C | 2.5 minutes | Worldclim |
| Bio12 | Annual Precipitation | mm/year | 2.5 minutes | Worldclim |
| Bio13 | Precipitation of Wettest Month | mm/month | 2.5 minutes | Worldclim |
| Bio14 | Precipitation of Driest Month | mm/month | 2.5 minutes | Worldclim |
| Bio15 | Precipitation Seasonality | % | 2.5 minutes | Worldclim |
| Bio16 | Precipitation of Wettest Quarter | mm/quarter | 2.5 minutes | Worldclim |
| Bio17 | Precipitation of Driest Quarter | mm/quarter | 2.5 minutes | Worldclim |
| Bio18 | Precipitation of Warmest Quarter | mm/quarter | 2.5 minutes | Worldclim |
| Bio19 | Precipitation of Coldest Quarter | mm/quarter | 2.5 minutes | Worldclim |
