# Supplementary material for: Distribution patterns of Quercus ilex from the last interglacial period to the future by ecological niche modeling
Source: Ecol Evol. 2023 Oct 19;13(10):e10606. doi: 10.1002/ece3.10606 (PMC10585444; doi:10.1002/ece3.10606)
Supplement: Supplementary file 5 — Table S3. [file ECE3-13-e10606-s008.docx]

**S3 Table.** Correlation matrix between 19 bioclimatic variables.

| Variables | bio1 | bio2 | bio3 | bio4 | **bio5** | bio6 | **bio7** | **bio8** | bio9 | bio10 | **bio11** | bio12 | bio13 | bio14 | **bio15** | **bio16** | **bio17** | bio18 | bio19 |
| --- | --- | --- | --- | --- | --- | --- | --- | --- | --- | --- | --- | --- | --- | --- | --- | --- | --- | --- | --- |
| bio1 | 1 |  |  |  |  |  |  |  |  |  |  |  |  |  |  |  |  |  |  |
| bio2 | *0.81* | 1 |  |  |  |  |  |  |  |  |  |  |  |  |  |  |  |  |  |
| bio3 | *0.79* | 0.72 | 1 |  |  |  |  |  |  |  |  |  |  |  |  |  |  |  |  |
| bio4 | -0.28 | 0.02 | -0.66 | 1 |  |  |  |  |  |  |  |  |  |  |  |  |  |  |  |
| **bio5** | *0.91* | *0.9* | 0.58 | 0.11 | 1 |  |  |  |  |  |  |  |  |  |  |  |  |  |  |
| bio6 | *0.88* | 0.52 | *0.85* | -0.68 | 0.62 | 1 |  |  |  |  |  |  |  |  |  |  |  |  |  |
| **bio7** | 0.16 | 0.53 | -0.2 | *0.85* | 0.55 | -0.32 | 1 |  |  |  |  |  |  |  |  |  |  |  |  |
| **bio8** | -0.06 | -0.03 | -0.33 | 0.48 | 0.08 | -0.27 | 0.38 | 1 |  |  |  |  |  |  |  |  |  |  |  |
| bio9 | *0.91* | *0.75* | *0.8* | -0.38 | *0.81* | *0.86* | 0.06 | -0.35 | 1 |  |  |  |  |  |  |  |  |  |  |
| bio10 | *0.93* | *0.84* | 0.57 | 0.08 | *0.99* | 0.67 | 0.48 | 0.11 | *0.81* | 1 |  |  |  |  |  |  |  |  |  |
| **bio11** | *0.95* | 0.67 | *0.88* | -0.58 | 0.74 | *0.98* | -0.15 | -0.22 | *0.91* | *0.77* | 1 |  |  |  |  |  |  |  |  |
| bio12 | -0.69 | -0.75 | -0.43 | -0.21 | -0.79 | -0.38 | -0.55 | -0.29 | -0.54 | -0.79 | -0.51 | 1 |  |  |  |  |  |  |  |
| bio13 | -0.61 | -0.68 | -0.39 | -0.17 | -0.7 | -0.34 | -0.48 | -0.29 | -0.46 | -0.69 | -0.46 | *0.94* | 1 |  |  |  |  |  |  |
| bio14 | -0.69 | -0.72 | -0.44 | -0.16 | -0.79 | -0.42 | -0.51 | -0.12 | -0.63 | -0.77 | -0.54 | *0.86* | 0.67 | 1 |  |  |  |  |  |
| **bio15** | 0.71 | 0.67 | 0.58 | -0.1 | 0.69 | 0.55 | 0.25 | -0.12 | 0.67 | 0.7 | 0.63 | -0.59 | -0.38 | -0.77 | 1 |  |  |  |  |
| **bio16** | -0.62 | -0.69 | -0.39 | -0.19 | -0.71 | -0.33 | -0.5 | -0.31 | -0.46 | -0.7 | -0.45 | *0.95* | 1 | 0.69 | -0.39 | 1 |  |  |  |
| **bio17** | -0.7 | -0.74 | -0.44 | -0.17 | -0.8 | -0.42 | -0.52 | -0.13 | -0.62 | -0.78 | -0.53 | *0.88* | 0.7 | *0.99* | -0.78 | 0.71 | 1 |  |  |
| bio18 | -0.84 | -0.77 | -0.66 | 0.11 | -0.85 | -0.68 | -0.3 | 0.15 | -0.86 | -0.84 | -0.76 | *0.78* | 0.67 | *0.87* | -0.72 | 0.68 | *0.87* | 1 |  |
| bio19 | -0.31 | -0.49 | -0.1 | -0.38 | -0.46 | 0 | -0.57 | -0.53 | -0.1 | -0.45 | -0.13 | *0.83* | *0.87* | 0.53 | -0.23 | *0.88* | 0.56 | 0.33 | 1 |
