# Supplementary material for: Distribution patterns of Quercus ilex from the last interglacial period to the future by ecological niche modeling
Source: Ecol Evol. 2023 Oct 19;13(10):e10606. doi: 10.1002/ece3.10606 (PMC10585444; doi:10.1002/ece3.10606)
Supplement: Supplementary file 6 — Table S4. [file ECE3-13-e10606-s007.docx]

**S4 Table.** Bioclimatic variables used as environmental input in the models.

| **Abbreviations** | **Variable name** | **Unit** | **Original resouliton** | **Source** |
| --- | --- | --- | --- | --- |
| Bio5 | Max Temperature of Warmest Month | °C | 2.5 minutes | Worldclim |
| Bio7 | Temperature Annual Range | °C | 2.5 minutes | Worldclim |
| Bio8 | Mean Temperature of Wettest Quarter | °C | 2.5 minutes | Worldclim |
| Bio11 | Mean Temperature of Coldest Quarter | °C | 2.5 minutes | Worldclim |
| Bio15 | Precipitation Seasonality | % | 2.5 minutes | Worldclim |
| Bio16 | Precipitation of Wettest Quarter | mm/quarter | 2.5 minutes | Worldclim |
| Bio17 | Precipitation of Driest Quarter | mm/quarter | 2.5 minutes | Worldclim |
