# Supplementary material for: Distribution patterns of Quercus ilex from the last interglacial period to the future by ecological niche modeling
Source: Ecol Evol. 2023 Oct 19;13(10):e10606. doi: 10.1002/ece3.10606 (PMC10585444; doi:10.1002/ece3.10606)
Supplement: Supplementary file 7 — Table S5. [file ECE3-13-e10606-s005.docx]

**S5 Table.** Fossil pollen records for the *Quercus ilex* from LIG, LGM and MH periods. Age oldest refers to age of the oldest *Quercus ilex* fossil pollen record in the given locality. LDD refers to both continous and discountinous low number of fossil pollen records which indicate either nearby refugia or long distance dispersal. Full references are given at the bottom of the table.

| **Taxon** | **Country** | **Locality/site** | **Latitude** | **Longitude** | | **Age Oldest** | | **LIG** | **LGM** | **MH** | **Reference** |
| --- | --- | --- | --- | --- | --- | --- | --- | --- | --- | --- | --- |
| *Quercus ilex* | France | Lac du Bouchet | 44.917 | 3.783 | 123400 | | LDD | | Absent | LDD | (Reille & de Beaulieu, 1990; Thouveny et al., 1994) |
| *Quercus ilex* | Lebanon | Chamsine | 33.753 | 35.948 | 12900 | | No data | | No data | LDD | (Hajar et al., 2010) |
| *Quercus ilex* | Lebanon | Aammiq | 33.732 | 35.794 | 14000 | | No data | | No data | LDD | (Hajar et al., 2010) |
| *Quercus ilex* | Greece | Ioannina I | 39.762 | 20.730 | 57971 | | No data | | LDD | LDD | (Bottema, 1974) |
| *Quercus ilex* | Greece | Tenaghi Philippon | 40.964 | 24.263 | 6232 | | No data | | No data | Presence | (Turner & Greig, 1975) |
| *Quercus ilex* | Italy | Lago Trifoglietti | 39.549 | 16.022 | 11439 | | No data | | No data | Presence | (Joannin et al., 2012) |
| *Quercus ilex* | Italy | Lago Grande di Monticchio | 40.931 | 15.605 | 132809 | | Presence | | Absent | Presence | (Watts et al., 1996; Huntley et al., 1999; Allen et al., 2000; Allen et al., 2002; Allen & Huntley, 2009) |
| *Quercus ilex* | Italy | Colfiorito | 43.019 | 12.875 | 20252 | | No data | | LDD | No data | (Brugiapaglia & de Beaulieu, 1995) |
| *Quercus ilex* | Italy | Lago di Massaciuccoli | 43.837 | 10.330 | 12150 | | No data | | No data | Presence | (Mariotti Lippi et al., 2007) |
| *Quercus ilex* | Italy | Lac du Verney-Dessus | 45.693 | 6.872 | 10933 | | No data | | No data | LDD | (Miras et al., 2006) |
| *Quercus ilex* | Italy | Torveraz | 45.695 | 6.860 | 9301 | | No data | | No data | LDD | (Miras et al., 2006) |
| *Quercus ilex* | Morroco | Lake Hachlaf | 33.550 | -4.99 | 6227 | | No data | | No data | Presence | (Nourelbait et al., 2016) |
| *Quercus ilex* | Tunisia | Djebel El Ghorra | 36.597 | 8.394 | 6726 | | No data | | No data | Presence | (Ben Tiba, 2014) |
| *Quercus ilex* | Spain | Quintanar de la Sierra | 42.033 | -3.016 | 23757 | | No data | | LDD | Presence | (Penalba, 1994; Peñalba et al., 1997) |
| *Quercus ilex* | Spain | Laguna Negra | 41.999 | -2.847 | 17708 | | No data | | No data | Presence | (von Engelbrechten, 1998) |
| *Quercus ilex* | Spain | Laguna Guallar | 41.400 | -0.217 | 9095 | | No data | | No data | Presence |  |
| *Quercus ilex* | Spain | Puerto de Los Tornos | 43.150 | -3.433 | 7235 | | No data | | No data | Presence | (Penalba, 1989) |
| *Quercus ilex* | Spain | Saldropo | 43.050 | -2.717 | 9098 | | No data | | No data | Presence | (Penalba, 1989; Penalba, 1994) |
| *Quercus ilex* | Spain | Puerto de Belate | 43.033 | -2.050 | 7650 | | No data | | No data | Presence | (Penalba, 1989; Penalba, 1994) |
| *Quercus ilex* | Spain | Atxuri | 43.250 | -1.551 | 6437 | | No data | | No data | LDD | (Penalba, 1989; Penalba, 1994) |
| *Quercus ilex* | Spain | Lake Banyoles | 42.129 | 2.752 | 31540 | | No data | | LDD | Presence | (Pèrez-Obiol & Julià, 1994) |
| *Quercus ilex* | Andorra | Bosc dels Estanyons | 42.480 | 1.628 | 11899 | | No data | | No data | LDD | (de Beaulieu et al., 2005) |
| *Quercus ilex* | Andorra | Planell de Perafita | 42.479 | 1.566 | 10158 | | No data | | No data | LDD | (Miras et al., 2010) |
| *Quercus ilex* | France | Watten | 50.833 | 2.213 | 8169 | | No data | | No data | LDD | (Gandouin et al., 2009) |
| *Quercus ilex* | France | La vie | 48.548 | -2.258 | 18702 | | No data | | No data | LDD | (Barbier & Visset, 2000) |
| *Quercus ilex* | France | Le Fourneau | 48.444 | -0.191 | 25679 | | No data | | Absent | LDD | (Barbier & Visset, 2000) |
| *Quercus ilex* | France | Locmariaquer | 47.554 | -2.932 | 11400 | | No data | | No data | LDD | (Visset et al., 1996) |
| *Quercus ilex* | France | Castle Suscinio | 47.512 | -2.727 | 7518 | | No data | | No data | LDD | (Visset & Bernard, 2006) |
| *Quercus ilex* | France | Ilot Sully | 47.222 | -1.551 | 7438 | | No data | | No data | Presence | (Visset et al., 2001) |
| *Quercus ilex* | France | Le Gesvres | 47.272 | -1.595 | 8275 | | No data | | No data | LDD | (Ouguerram & Visset, 2001) |
| *Quercus ilex* | France | Saint Viaud Cantin | 47.265 | -2.010 | 6434 | | No data | | No data | LDD | (Bernard, 1996) |
| *Quercus ilex* | France | Petit Marais | 47.217 | -2.102 | 7611 | | No data | | No data | LDD | (Marchand et al., 1999) |
| *Quercus ilex* | France | Petit Rocher | 46.663 | -1.915 | 8952 | | No data | | No data | LDD | (Joly & Visset, 2009) |
| *Quercus ilex* | France | Jaunay | 46.662 | -1.892 | 9010 | | No data | | No data | LDD | (Joly & Visset, 2009) |
| *Quercus ilex* | France | Vertonne | 46.549 | -1.764 | 7893 | | No data | | No data | LDD | (Joly & Visset, 2009) |
| *Quercus ilex* | France | La Grange | 46.089 | -0.775 | 9744 | | No data | | No data | LDD | (Joly & Visset, 2009) |
| *Quercus ilex* | France | Pierre Folle | 47.018 | -1.089 | 6351 | | No data | | No data | LDD | (Bernard, 1996) |
| *Quercus ilex* | France | Chantemerle | 45.916 | 0.016 | 8093 | | No data | | No data | LDD | (Fauquette et al., 1999) |
| *Quercus ilex* | France  France | Lac du Mont de Belier | 45.339 | 2.645 | 12476 | | No data | | No data | LDD | (de Beaulieu et al., 1985) |
| *Quercus ilex* | France | Bonnecombe | 44.566 | 3.125 | 13555 | | No data | | No data | LDD | (de Beaulieu et al., 1985) |
| *Quercus ilex* | France | Lac du Bouchet (BOUCHB53) | 44.916 | 3.783 | 69779 | | No data | | Absent | LDD | (Reille & de Beaulieu, 1988) |
| *Quercus ilex* | France | Lac du Bouchet | 44.916 | 3.783 | 50000 | | No data | | Absent | LDD | (Reille & de Beaulieu, 1988) |
| *Quercus ilex* | France | Hières sur Amby(AMBY80) | 45.790 | 5.283 | 16333 | | No data | | No data | LDD | (Clerc, 1988) |
| *Quercus ilex* | France | Le Grand Lemps(LEMPS85) | 45.473 | 5.416 | 9286 | | No data | | No data | LDD | (Clerc, 1988) |
| *Quercus ilex* | France | Le Grand Lemps(LEMPS83) | 45.473 | 5.416 | 16268 | | No data | | No data | LDD | (Clerc, 1988) |
| *Quercus ilex* | France | Grand Ratz le Pellet | 45.425 | 5.608 | 15500 | | No data | | No data | LDD | (Clerc, 1988) |
| *Quercus ilex* | France | Saint Julien de Ratz(RATZ81) | 45.348 | 5.655 | 19727 | | No data | | Absent | LDD | (Clerc, 1988) |
| *Quercus ilex* | France | Saint Julien de Ratz(RATZ81) | 45.348 | 5.655 | 12348 | | No data | | No data | LDD | (Clerc, 1988) |
| *Quercus ilex* | France | Lac des Boites | 45.056 | 5.882 | 15614 | | No data | | No data | LDD | (Nakagawa, 1998) |
| *Q.ilex/Q.coccifera* | Greece | Lake Kournas | 35.329 | 24.275 | 9554 | | No data | | No data | Presence | (Jouffroy-Bapicot et al., 2021) |
| *Q.ilex/Q.coccifera* | Greece | Lake Lerna | 37.578 | 22.728 | 6788 | | No data | | No data | Presence | (Jahns, 1993) |
| *Q.ilex/Q.coccifera* | France | Piantarella lagoon | 41.373 | 9.219 | 6615 | | No data | | No data | Presence | (Revelles et al., 2019) |
| *Q.ilex/Q.coccifera* | France | Saint Florent | 42.672 | 9.304 | 6301 | | No data | | No data | Presence | (Revelles et al., 2019) |
| *Q.ilex/Q.coccifera* | Spain | Les Palanques | 42.162 | 2.438 | 10098 | | No data | | No data | Presence | (Piqué et al., 2018; Revelles et al., 2018) |
| *Q.ilex/Q.coccifera* | Spain | Creixell | 41.155 | 1.433 | 6575 | | No data | | No data | Presence | (Burjachs & Expósito, 2015) |
| *Q.ilex/Q.coccifera* | Spain | Pego-Oliva | 38.876 | -0.088 | 7003 | | No data | | No data | Presence | (Brisset et al., 2020) |
| *Q.ilex/Q.coccifera* | Spain | Antas | 37.208 | -1.823 | 10498 | | No data | | No data | Presence | (Yll et al., 1997; Pantaléon-Cano et al., 2003) |
| *Q.ilex/Q.coccifera* | Morroco | Dayet M'Had | 35.128 | -5.438 | 5741 | | No data | | No data | Presence | (Cheddadi et al., 2015, 2017) |
| *Q.ilex/Q.coccifera* | Morroco | Bab El Karn | 35.022 | -5.206 | 9163 | | No data | | No data | Presence | (Cheddadi et al., 2016, 2017) |
| *Q.ilex/Q. coccifera*-type | Turkey | Tecer Gölü | 39.431 | 37.083 | 5850 | | No data | | No data | LDD | (Kuzucuoğlu et al., 2011) |
| *Q.ilex/Q. coccifera*-type | France | Bastani | 42.065 | 9.134 | 11732 | | No data | | No data | Presence | (Leroy & Roiron, 1996) |
| *Q.ilex*-type | Black Sea | MD72/5-22 | 42.201 | 36.492 | 17923 | | No data | | No data | LDD | (Shumilovskikh et al., 2012) |
| *Q.ilex*-type | Black Sea | MD72/5-25 | 42.100 | 36.616 | 63928 | | No data | | LDD | No data | (Nowaczyk et al., 2012; Shumilovskikh et al., 2014) |
| *Q.ilex*-type | Morocco | Ifri Oudadane | 35.212 | -3.258 | 10860 | | No data | | No data | Presence | (Linstädter & Kehl, 2012; Morales et al., 2013; Zapata et al., 2013) |
| *Q.ilex*-type | Greece | Akotivika | 37.036 | 22.076 | 6395 | | No data | | No data | Presence | (Engel et al., 2009) |
| *Q.ilex*-type | Greece | Pertouli | 39.524 | 21.477 | 6178 | | No data | | No data | Presence | (Athanasiadis, 1975) |
| *Q.ilex*-type | Greece | Litochoro | 40.138 | 22.546 | 5135 | | No data | | No data | Presence | (Athanasiadis, 1975) |
| *Q.ilex*-type | Greece | Khimaditis Ib | 40.627 | 21.599 | 17503 | | No data | | No data | LDD | (Bottema, 1974) |
| *Q.ilex*-type | Montenegro | Zminje Jezero | 43.155 | 19.07 | 12740 | | No data | | No data | LDD | (Cagliero. et al., 2023) |
| *Q.ilex*-type | Croatia | Bokanjacko | 44.183 | 15.233 | 9559 | | No data | | No data | Presence | (Grüger, 1996) |
| *Q.ilex*-type | Switzerland | Lago di Origlio | 46.051 | 8.934 | 19423 | | No data | | No data | LDD | (Tinner et al., 1999) |
| *Q.ilex*-type | Italy | Biviere di Gela | 37.018 | 14.344 | 7334 | | No data | | No data | Presence | (Colombaroli et al., 2009; Noti et al., 2009) |
| *Q.ilex*-type | Italy | Gorgo Basso(GBASSO) | 37.600 | 12.650 | 10186 | | No data | | No data | Presence | (Tinner et al., 2009) |
| *Q.ilex*-type | Italy | Gorgo Basso(GBASSO) | 37.600 | 12.650 | 10187 | | No data | | No data | Presence | (Calò et al., 2012; Tinner et al., 2009) |
| *Q.ilex*-type | Italy | Lago Preola | 37.616 | 12.633 | 10299 | | No data | | No data | Presence | (Calò et al., 2012) |
| *Q.ilex*-type | Italy | Gorgo Tondo | 37.901 | 13.412 | 5597 | | No data | | No data | Presence | (Tinner et al., 2016) |
| *Q.ilex*-type | Italy | Urgo Pietra Giordano | 37.852 | 14.127 | 6993 | | No data | | No data | Presence | (Tinner et al., 2016) |
| *Q.ilex*-type | Italy | Urio Quattrocchi | 37.901 | 14.395 | 10238 | | No data | | No data | Presence | (Bisculm et al., 2012) |
| *Q.ilex*-type | Italy | Lago di Nemi | 41.716 | 12.700 | 11386 | | No data | | No data | Presence | (Mercuri et al., 2002; Mercuri et al., 2013) |
| *Q.ilex*-type | Italy | Lago Albano | 41.750 | 12.666 | 14039 | | No data | | No data | Presence | (Mercuri et al., 2002; Mercuri et al., 2013) |
| *Q.ilex*-type | Italy | Lago di Martignano | 42.113 | 12.317 | 13335 | | No data | | No data | Presence | (Kelly & Huntley, 1991) |
| *Q.ilex*-type | Italy | Lago dell'Accesa | 42.987 | 10.895 | 11551 | | No data | | No data | Presence | (Vannière et al., 2008; Colombaroli et al., 2009) |
| *Q.ilex*-type | Italy | Lago di Massaciuccoli(MASSACHR) | 42.837 | 10.330 | 6070 | | No data | | No data | Presence | (Colombaroli et al., 2007, 2009) |
| *Q.ilex*-type | Italy | Lago di Massaciuccoli(MASSACHR) | 42.837 | 10.330 | 6941 | | No data | | No data | Presence | (Colombaroli et al., 2007, 2009) |
| *Q.ilex*-type | Italy | Lago del Greppo | 44.119 | 10.673 | 14873 | | No data | | No data | LDD | (Vescovi et al., 2010) |
| *Q.ilex*-type | Italy | Lago Verdarolo | 44.359 | 10.123 | 14427 | | No data | | No data | LDD | (Samartin et al., 2017; Morales-Molino. et al., 2021) |
| *Q.ilex*-type | Italy | Bondone | 46.017 | 11.041 | 18596 | | No data | | Absent | LDD | (Grüger, 1968) |
| *Q.ilex*-type | Italy | Lago del Segrino | 45.828 | 9.264 | 13157 | | No data | | No data | LDD | (Gobet et al., 2000) |
| *Q.ilex*-type | Italy | Ortasee | 45.808 | 8.396 | 15965 | | No data | | No data | LDD | (Rösch. et al., 2012) |
| *Q.ilex*-type | Italy | Lago Piccolo di Avigliana | 45.053 | 7.391 | 20423 | | No data | | Absent | LDD | (Finsinger & Tinner, 2006; Finsinger et al., 2008; Finsinger et al., 2011) |
| *Q.ilex*-type | Italy | Laghi dell'Orgials | 44.233 | 7.133 | 25445 | | No data | | Absent | LDD | (Ortu et al., 2008) |
| *Q.ilex*-type | Italy | Torbiera del Biecai | 44.185 | 7.717 | 19013 | | No data | | Absent | LDD | (Ortu et al., 2008) |
| *Q.ilex*-type | Portugal | Beliche-Guadiana | 37.266 | -7.450 | 13091 | | No data | | No data | Presence | (Fletcher et al., 2007) |
| *Q.ilex*-type | Portugal | Lagoacho das Favas | 40.357 | -7.635 | 8153 | | No data | | No data | Presence | (van der Knaap & van Leeuwen, 1995) |
| *Q.ilex*-type | Portugal | Lagoa Comprida 1 | 40.358 | -7.638 | 13295 | | No data | | No data | LDD | (Janssen & Woldringh, 1981; van der Knaap & van Leeuwen, 1997) |
| *Q.ilex*-type | Portugal | Lagoa Comprida 2 | 40.326 | -7.636 | 12444 | | No data | | No data | Presence | (Janssen & Woldringh, 1981; Van Den Brink & Janssen, 1985) |
| *Q.ilex*-type | Spain | Bajondillo | 36.619 | -4.496 | 142228 | | Presenve | | LDD | Presence | (Cortés-Sánchez et al., 2008) |
| *Q.ilex*-type | Spain | Padul | 37.000 | -3.400 | 23600 | | No data | | Presence | Presence | (Pons & Reille, 1988) |
| *Q.ilex*-type | Spain | Siles | 38.400 | -2.500 | 20300 | | No data | | Presence | Presence | (Carrión, 2002) |
| *Q.ilex*-type | Spain | San Rafael | 36.773 | -2.601 | 20927 | | No data | | Presence | Presence | (Yll et al., 1997; Pantaléon-Cano et al., 2003) |
| *Q.ilex*-type | Spain | Roquetas de Mar | 36.794 | -2.588 | 6999 | | No data | | No data | Presence | (Yll et al., 1997; Pantaléon-Cano et al., 2003) |
| *Q.ilex*-type | Spain | Cucú cueva | 37.638 | -2.258 | 130000 | | Presence | | No data | No data | (González-Ramón et al., 2012) |
| *Q.ilex*-type | Spain | Navarrés(NAVA1) | 39.100 | -0.683 | 19516 | | No data | | LDD | Presence | (Carrion & Dupre, 1996) |
| *Q.ilex*-type | Spain | Navarrés(NAVA2) | 39.100 | 0.683 | 19583 | | No data | | LDD | Presence | (Carrion & Dupre, 1996) |
| *Q.ilex*-type | Spain | Navarrés(NAVA3) | 39.100 | 0.683 | 36577 | | No data | | Absent | Presence | (Carrión & Van Geel, 1999) |
| *Q.ilex*-type | Spain | Albufera Alcudia | 39.792 | 3.119 | 8107 | | No data | | No data | Presence | (Burjachs, 1994) |
| *Q.ilex*-type | Spain | Algendar | 39.94 | 3.958 | 10066 | | No data | | No data | Presence | (Yll et al., 1997) |
| *Q.ilex*-type | Spain | Cala Galdana | 39.936 | 3.965 | 8386 | | No data | | No data | LDD | (Yll et al., 1994) |
| *Q.ilex*-type | Spain | Son Bou | 39.924 | 4.027 | 24418 | | No data | | Presence | LDD | (Yll et al., 1997) |
| *Q.ilex*-type | Spain | Hort Timoner | 39.875 | 4.126 | 9477 | | No data | | No data | Presence | (Yll et al., 1997) |
| *Q.ilex*-type | Spain | Cala'n Porter | 39.870 | 4.131 | 8998 | | No data | | No data | Presence | (Yll et al., 1997) |
| *Q.ilex*-type | Spain | MD95-2039 | 40.578 | -10.348 | 44875 | | No data | | LDD | No data | (Schönfeld et al., 2003; Roucoux et al., 2005) |
| *Q.ilex*-type | Spain | El Maíllo mire | 40.546 | -6.209 | 10553 | | No data | | No data | Presence | (Morales-Molino et al., 2013) |
| *Q.ilex*-type | Spain | El Carrizal lake | 41.318 | -4.144 | 9784 | | No data | | No data | LDD | (Franco-Múgica et al., 2005) |
| *Q.ilex*-type | Spain | Canaleja | 40.900 | -2.450 | 10241 | | No data | | No data | Presence | (Cerrillo-Cuenca & González-Cordero, 2011) |
| *Q.ilex*-type | Spain | Sanabria Marsh | 42.100 | -6.733 | 18089 | | No data | | No data | Presence | (Allen et al., 1996) |
| *Q.ilex*-type | Spain | Ayoó de Vidriales | 42.126 | -6.070 | 18468 | | No data | | No data | LDD | (Morales-Molino & García-Antón, 2014) |
| *Q.ilex*-type | Spain | Espinosa de Cerrato | 41.952 | -3.939 | 11500 | | No data | | No data | Presence | (Morales-Molino et al., 2017) |
| *Q.ilex*-type | Spain | Tubilla del Lago | 41.808 | -3.572 | 7458 | | No data | | No data | LDD | (Morales-Molino et al., 2017) |
| *Q.ilex*-type | Spain | Las Pardillas Lake | 42.043 | -3.045 | 10604 | | No data | | No data | Presence | (Goñi & Hannon, 1999) |
| *Q.ilex*-type | Spain | Puerto de Belate | 43.033 | -2.050 | 8097 | | No data | | No data | Presence | (Penalba, 1989) |
| *Q.ilex*-type | France | Lake Racou | 42.554 | 2.008 | 12216 | | No data | | No data | LDD | (Guiter et al., 2005) |
| *Q.ilex*-type | France | MD99-2348 (103) | 42.692 | 3.841 | 24975 | | No data | | LDD | No data | (Beaudouin et al., 2005; Célia et al., 2007) |
| *Q.ilex*-type | France | Tourbière de Gatimort | 43.575 | 2.785 | 7892 | | No data | | No data | LDD | (Pulido, 2006) |
| *Q.ilex*-type | France | Marais de Fos | 43.436 | 4.936 | 6134 | | No data | | No data | Presence | (Vella & Provansal, 2000) |
| *Q.ilex*-type | France | Le Marais de la Perge (South) | 45.382 | -1.115 | 6314 | | No data | | No data | LDD | (Tastet & Diot, 1995) |
| *Q.ilex*-type | France | Marais de la Perge | 45.379 | -1.01 | 7888 | | No data | | No data | LDD | (Tastet & Diot, 1995) |
| *Q.ilex*-type | France | Tourbière des Nassettes | 44.466 | 3.641 | 8850 | | No data | | No data | LDD | (Pulido, 2006) |
| *Q.ilex*-type | France | Tourbière des Narses Mortes | 44.433 | 3.600 | 8850 | | No data | | No data | LDD | (Jouffroy-Bapicot et al., 2007; Pulido, 2006) |
| *Q.ilex*-type | France | Le Grand Etang de Suze-La-Rousse | 44.323 | 4.833 | 18514 | | No data | | Absent | LDD | (Argant, 1990) |
| *Q.ilex*-type | France | Lac de Praver | 45.073 | 5.856 | 7210 | | No data | | No data | LDD | (Nakagawa, 1998) |
| *Q.ilex*-type | France | Lac des Boites | 45.056 | 5.885 | 11345 | | No data | | No data | LDD | (Nakagawa, 1998) |
| *Q.ilex*-type | France | La Gourre | 44.385 | 6.003 | 15717 | | No data | | No data | Presence | (Muller et al., 2012) |
| *Q.ilex*-type | France | Correo | 44.555 | 5.995 | 29475 | | No data | | LDD | LDD | (Nakagawa, 1998) |
| *Q.ilex*-type | France | Pelléautier | 44.522 | 6.183 | 7768 | | No data | | No data | LDD | (de Beaulieu, 1977) |
| *Q.ilex*-type | France | Biot | 43.800 | 7.100 | 13882 | | No data | | No data | Presence | (Nicol-Pichard & Dubar, 1998) |
| *Q.ilex*-type | France | Lac Long Inférieur | 44.057 | 7.450 | 20811 | | No data | | Absent | LDD | (de Beaulieu, 1977) |
| *Q.ilex*-type | France | Lac des Grenouilles | 44.098 | 7.483 | 14662 | | No data | | No data | Presence | (Finsinger et al., 2021) |
| *Q.ilex*-type | France | Sabion | 44.13 | 7.473 | 13300 | | No data | | No data | LDD | (de Beaulieu, 1977) |
| *Q.ilex*-type | France | Etang d'Ouveillan | 43.266 | 3.000 | 8934 | | No data | | No data | LDD | (Goñi & Fernanda, 1995) |
| *Q.ilex*-type | Egypt | Lake Qarun | 29.47 | 30.637 | 9828 | | No data | | No data | LDD | (Hamdan et al., 2020) |
| *Q.ilex*-type | Germany | Steerenmoos | 47.805 | 8.2 | 10352 | | No data | | No data | Presence | (Rösch et al., 2012) |
| *Q.ilex*-type | Atlantic Ocean | M 15669-1 | 34.891 | -7.815 | 121700 | | Presence | | LDD | Presence | (Hooghiemstra et al., 1992) |
| *Q.ilex*-type | Atlantic Ocean | M 16004-1 | 29.978 | -10.646 | 140000 | | Presence | | Presence | Presence | (Hooghiemstra et al., 1992) |
| *Q.ilex/Q.coccifera* | Spain | Abric Romaní | 41.519 | 1.699 | 110000 | | Presence | | No data | No data | (Biltekin et al., 2019) |
| *Quercus* (evergreen) | Atlantic Ocean | MD952042 | 37.675 | -10.167 | 158050 | | Presence | | No data | No data | (Sánchez Goñi et al., 1999) |
| *Quercus* (evergreen) | Atlantic Ocean | Bay of Biscay | 45.35 | -5.216 | 143279 | | LDD | | No data | No data | (Sanchez Goñi et al., 2012) |
| *Q.ilex-type* | Greece | Gulf of Corinth | 38.144 | 22.758 | - | | Presence | | Presence | Presence | (Kafetzidou et al., 2023) |
| *Q.ilex-type* | Greece | Lake Ohrid | 40.9 | 20.633 | 500000 | | Presence | | Presence | Presence | (Sadori et al., 2016) |
| *Q.ilex-type* | Italy | Azzano Decimo | 45.875 | 12.712 | - | | Presence | | No data | No data | (Pini et al., 2009) |
| *Q.ilex-type* | Italy | Valle di Castiglione | 41.898 | 12.711 | 220000 | | Presence | | No data | No data | (Follieri et al., 1989) |
| *Q.ilex-type* | Greece | Lake Copais | 38.44 | 23.0489 | - | | Presence | | Absent | Presence | (Okuda et al., 2001) |
| *Quercus (evergreen)* | Spain | El Canizar de Villarquemado | 40.5 | -1.3 | 135000 | | Presence | | Absent | Presence | (González-Sampériz et al., 2020) |

**References**

Allen, J.R.M, Watts, W. ., McGee, E., & Huntley, B. (2002). Holocene environmental variability—the record from Lago Grande di Monticchio, Italy. *Quaternary International*, *88*(1), 69–80. <https://doi.org/10.1016/S1040-6182(01)00074-X>

Allen, J. R.M., Huntley, B., & Watts, W. A. (1996). The vegetation and climate of northwest Iberia over the last 14000 yr. *Journal of Quaternary Science*, *11*(2), 125–147. [https://doi.org/10.1002/(SICI)1099-1417(199603/04)11:2<125::AID-JQS232>3.0.CO;2-U](https://doi.org/10.1002/(SICI)1099-1417(199603/04)11:2%3c125::AID-JQS232%3e3.0.CO;2-U)

Allen, J. R. M, & Huntley, B. (2009). Last Interglacial palaeovegetation, palaeoenvironments and chronology: a new record from Lago Grande di Monticchio, southern Italy. *Quaternary Science Reviews*, *28*(15), 1521–1538. <https://doi.org/10.1016/j.quascirev.2009.02.013>

Allen, J. R. M, Watts, W. A., & Huntley, B. (2000). Weichselian palynostratigraphy, palaeovegetation and palaeoenvironment; the record from Lago Grande di Monticchio, southern Italy. *Quaternary International*, *73*–*74*, 91–110. <https://doi.org/10.1016/S1040-6182(00)00067-7>

Argant, J. (1990). Climat et environnement au Quaternaire dans le Bassin du Rhône d’après les données palynologiques. In *Documents des Laboratoires de Géologie, Lyon* (pp. 3–199).

Athanasiadis, N. (1975). Zur postglazialen Vegetationsentwicklung von Litochoro Katerinis und Pertouli Trikalon (Griechenland). *Flora*, *164*(1), 99–132. <https://doi.org/10.1016/S0367-2530(17)31791-7>

Barbier, D., & Visset, L. (2000). Les spécificités d’un Tardiglaciaire armoricain: étude pollinique synthétique à partir de trois tourbières du Nord-Est mayennais (France). *Quaternaire*, *11*, 99–106. <https://doi.org/10.3406/quate.2000.1659>

Beaudouin, C., Suc, J.-P., Acherki, N., Courtois, L., Rabineau, M., Aloïsi, J.-C., Sierro, F. J., & Oberlin, C. (2005). Palynology of the northwestern Mediterranean shelf (Gulf of Lions): First vegetational record for the last climatic cycle. *Marine and Petroleum Geology*, *22*(6), 845–863. <https://doi.org/https://doi.org/10.1016/j.marpetgeo.2005.03.005>

Beaulieu, J.-L. D., Miras, Y., Andrieu-Ponel, V., & Guiter, F. (2005). Vegetation dynamics in north-western Mediterranean regions: Instability of the Mediterranean bioclimate. Plant Biosystems. *An International Journal Dealing with All Aspects of Plant Biology*, *132*(2), 114–126. <https://doi.org/10.1080/11263500500197858>

Ben Tiba, B. (2014). Pollen profile GHORRA01, Djebel El Ghorra, Tunisia. In *European Pollen Database (EPD)*. PANGAEA. <https://doi.org/10.1594/PANGAEA.835810>

Bernard, J. (1996). *Paléoenvironnement du Pays de Retz et du marais breton-vendéen.* Université de Nantes. Doctoral dissertation. Université de Nantes, Nantes, France

Biltekin, D., Burjachs, F., Vallverdú, J., Sharp, W. D., Mertz-Kraus, R., Chacón, M. G., Saladié, P., Bischoff, J. L., & Carbonell, E. (2019). Vegetation and climate record from Abric Romaní (Capellades, northeast Iberia) during the Upper Pleistocene (MIS 5d−3). Quaternary Science Reviews, 220, 154–164. <https://doi.org/10.1016/j.quascirev.2019.07.035>

Bisculm, M., Colombaroli, D., Vescovi, E., van Leeuwen, J. F. N., Henne, P. D., Rothen, J., Procacci, G., Pasta, S., La Mantia, T., & Tinner, W. (2012). Holocene vegetation and fire dynamics in the supra-mediterranean belt of the Nebrodi Mountains (Sicily, Italy). *Journal of Quaternary Science*, *27*(7), 687–698. <https://doi.org/https://doi.org/10.1002/jqs.2551>

Bottema, S. (1974). Late quaternary vegetation history of northwestern Greece. *s.n*, 190.

Brisset, E., Revelles, J., Expósito, I., Bernabeu Aubán, J., & Burjachs, F. (2020). Socio-Ecological Contingencies with Climate Changes over the Prehistory in the Mediterranean Iberia. In *Quaternary* (Vol. 3, Issue 3). <https://doi.org/10.3390/quat3030019>

Brugiapaglia, E., & J.L. de Beaulieu. (1995). Etude de la dynamique végétale tardiglaciaire et Holocène en Italie centrale: le marais de Colfiorito (Ombrie). *Comptes Rendus de l’Académie Des Sciences*, *Serie II*(321), 617–622.

Burjachs, F. (1994). Dinámica de la vegetación durante el Holoceno en la isla de Mallorca. In M. E. B. I. Mateu, M. Dupré, J. Güemes (Ed.), *Trabajos de Palinología básica y aplicada* (pp. 199–210).

Burjachs, F., & Expósito, I. (2015). Charcoal and pollen analysis: Examples of Holocene fire dynamics in Mediterranean Iberian Peninsula. *CATENA*, *135*, 340–349. <https://doi.org/10.1016/j.catena.2014.10.006>

Cagliero, E., L. Paradis, Marchi, N., Lisztes-Szabó, Z., Braun, M., Hubay, K., Sabatier, P., Curovic, M., Spalević, V., Motta, R., Lingua, E., & Finsinger., W. (2023). The role of fire disturbances, human activities and climate change for long-term forest dynamics in upper-montane forests of the central Dinaric Alps. *The Holocene:In Press*.

Calò, C., Henne, P. D., Curry, B., Magny, M., Vescovi, E., La Mantia, T., Pasta, S., Vannière, B., & Tinner, W. (2012). Spatio-temporal patterns of Holocene environmental change in southern Sicily. *Palaeogeography, Palaeoclimatology, Palaeoecology*, *323*–*325*, 110–122. <https://doi.org/10.1016/j.palaeo.2012.01.038>

Carrion, J. S., & Dupre, M. (1996). Late Quaternary Vegetational History at Navarres, Eastern Spain. A Two Core Approach. The New Phytologist, 134(1), 177–191. 10.1111/J.1469-8137.1996.TB01157.X

Carrión, J S, & Van Geel, B. (1999). Fine-resolution Upper Weichselian and Holocene palynological record from Navarrés (Valencia, Spain) and a discussion about factors of Mediterranean forest succession. *Review of Palaeobotany and Palynology*, *106*(3), 209–236. <https://doi.org/10.1016/S0034-6667(99)00009-3>

Carrión, J. S. (2002). Patterns and processes of Late Quaternary environmental change in a montane region of southwestern Europe. *Quaternary Science Reviews*, *21*(18), 2047–2066. <https://doi.org/10.1016/S0277-3791(02)00010-0>

Célia, B., Gwenael, J., Suc, J.-P., Berne, S., & Escarguel, G. (2007). Vegetation dynamics in Southern France during the last 30 ky BP in the light of marine palynology. *Quaternary Science Reviews*, *26*, 1037–1054. <https://doi.org/10.1016/j.quascirev.2006.12.009>

Cerrillo-Cuenca, E., & González-Cordero, A. (2011). Burial prehistoric caves in the interior basin of river Tagus: the complex at Canaleja gorge (Romangordo, Caceres, Spain). *BAR International Series*, *2219*, 21–42.

Cheddadi, R., Bouaissa, O., Rhoujjati, A., & Dezileau, L. (2016). Environmental changes in the Moroccan western Rif mountains over the last 9,000 years. *Quaternaire*, *27*, 15–25. <https://doi.org/10.4000/quaternaire.7517>

Cheddadi, R., Henrot, A.-J., François, L., Boyer, F., Bush, M., Carré, M., Coissac, E., De Oliveira, P. E., Ficetola, F., Hambuckers, A., Huang, K., Lézine, A.-M., Nourelbait, M., Rhoujjati, A., Taberlet, P., Sarmiento, F., Abel-Schaad, D., Alba-Sánchez, F., & Zheng, Z. (2017). Microrefugia, Climate Change, and Conservation of Cedrus atlantica in the Rif Mountains, Morocco . In *Frontiers in Ecology and Evolution*  (Vol. 5). 10.3389/fevo.2017.00114

Cheddadi, R., Nourelbait, M., Bouaissa, O., Tabel, J., Rhoujjati, A., López-Sáez, J. A., Alba-Sánchez, F., Khater, C., Ballouche, A., Dezileau, L., & Lamb, H. (2015). A History of Human Impact on Moroccan Mountain Landscapes. *African Archaeological Review*, *32*, 233–248. <https://doi.org/10.1007/s10437-015-9186-7>

Clerc, J. (1988). *Recherches pollenanalytiques sur la paléo-écologie Tardiglaciaire et Holocène du Bas-Dauphiné.* PhD Thesis, Université St. Jérôme, Marseille, France

Colombaroli, D., Marchetto, A., & Tinner, W. (2007). Long-term interactions between Mediterranean climate, vegetation and fire regime at Lago di Massaciuccoli (Tuscany, Italy). *Journal of Ecology*, *95*(4), 755–770. <https://doi.org/10.1111/j.1365-2745.2007.01240.x>

Colombaroli, D., Tinner, W., Leeuwen, J., Noti, R., Vescovi, E., Vannière, B., Magny, M., Schmidt, R., & Bugmann, H. (2009). Response of broadleaved evergreen Mediterranean forest vegetation to fire disturbance during the Holocene: Insights from the peri-Adriatic region. *Journal of Biogeography*, *36*, 314–326. <https://doi.org/10.1111/j.1365-2699.2008.01987.x>

Cortés-Sánchez, M., Morales-Muñiz, A., Simón-Vallejo, M. D., Bergadà-Zapata, M. M., Delgado-Huertas, A., López-García, P., López-Sáez, J. A., Lozano-Francisco, M. C., Riquelme-Cantal, J. A., Roselló-Izquierdo, E., Sánchez-Marco, A., & Vera-Peláez, J. L. (2008). Palaeoenvironmental and cultural dynamics of the coast of Málaga (Andalusia, Spain) during the Upper Pleistocene and early Holocene. *Quaternary Science Reviews*, *27*(23), 2176–2193. <https://doi.org/10.1016/j.quascirev.2008.03.010>

de Beaulieu, J. L. (1977). *Contribution pollenanalytique à l’histoire tardiglaciaire et Holocène de la végétation des Alpes méridionales françaices*. Doctoral dissertation. Université d’Aix-Marseille, Marseille, France.

de Beaulieu, J. L., Pons, A., & Reille, M. (1985). Recherches pollenanalytiques sur l’histoire tardiglaciaire et holocene de la vegetation des Monts d’Aubrac (Massif Central, France). *Review of Palaeobotany and Palynology*, *44*(1), 37–80. <https://doi.org/https://doi.org/10.1016/0034-6667(85)90028-4>

Engel, M., Knipping, M., Brückner, H., Kiderlen, M., & Kraft, J. (2009). Reconstructing middle to late Holocene palaeogeographies of the lower Messenian plain (southwestern Peloponnese, Greece): Coastline migration, vegetation history and sea level change. *Palaeogeography Palaeoclimatology Palaeoecology*, 257–270. <https://doi.org/10.1016/j.palaeo.2009.10.005>

Fauquette, S., Guiot, J., Menut, M., de Beaulieu, J.-L., Reille, M., & Guenet, P. (1999). Vegetation and climate since the last interglacial in the Vienne area (France). *Global and Planetary Change*, *20*(1), 1–17. <https://doi.org/10.1016/S0921-8181(98)00054-X>

Finsinger, W, Lane, C. S., van Den Brand, G. J., Wagner-Cremer, F., Blockley, S. P. E., & Lotter, A. F. (2011). The lateglacial Quercus expansion in the southern European Alps: rapid vegetation response to a late Allerød climate warming? *Journal of Quaternary Science*, *26*(7), 694–702. <https://doi.org/https://doi.org/10.1002/jqs.1493>

Finsinger, Walter, Belis, C., Blockley, S., Eicher, U., Leuenberger, M., Lotter, A., & Ammann, B. (2008). Temporal patterns in lacustrine stable isotopes as evidence for climate change during the late glacial in the Southern European Alps. *Journal of Paleolimnology*, *40*, 885–895. <https://doi.org/10.1007/s10933-008-9205-7>

Finsinger, W., & Tinner, W. (2006). Holocene vegetation and land-use changes in response to climatic changes in the forelands of the southwestern Alps, Italy. *Journal of Quaternary Science*, *21*(3), 243–258. <https://doi.org/10.1002/jqs.971>

Finsinger, W., Vanel, Q., Ribolini, A., & Tinner, W. (2021). Early to late Holocene vegetation and fire dynamics at the treeline in the Maritime Alps. *Vegetation History and Archaeobotany*, *30*. <https://doi.org/10.1007/s00334-020-00795-x>

Fletcher, W., Boski, T., & Moura, D. (2007). Palynological evidence for environmental and climatic change in the lower Guadiana valley, Portugal, during the last 13 000 years. *Holocene*, *17*, 481–494. <https://doi.org/10.1177/0959683607077027>

Follieri, M., Magri, D., & Sadori, L. (1989). Pollen stratigraphical synthesis from Valle di Castiglione (Roma). *Quaternary International*, *3*–*4*, 81–84. <https://doi.org/10.1016/1040-6182(89)90076-1>

Franco-Múgica, F., García-Antón, M., Maldonado-Ruiz, J., Morla-Juaristi, C., & Sainz-Ollero, H. (2005). Ancient pine forest on inland dunes in the Spanish northern meseta. *Quaternary Research*, *63*(1), 1–14. <https://doi.org/10.1016/j.yqres.2004.08.004>

Gandouin, E., Ponel, P., Andrieu-Ponel, V., Guiter, F., de Beaulieu, jacques-L., Djamali, M., Franquet, E., Vliet-Lanoë, B., Alvitre, M., Meurisse, M., Brocandel, M., & Brulhet, J. (2009). 10,000 years of vegetation history of the Aa palaeoestuary, St-Omer Basin, northern France. *Review of Palaeobotany and Palynology*, *156*, 307–318. <https://doi.org/10.1016/j.revpalbo.2009.03.008>

Gobet, E., Tinner, W., Hubschmid, P., Jansen, I., Wehrli, M., Ammann, B., & Wick, L. (2000). Influence of human impact and bedrock differences on the vegetational history of the Insubrian Southern Alps. *Vegetation History and Archaeobotany*, *9*(3), 175–187. <https://doi.org/10.1007/BF01299802>

Goñi, M. F. S., & Hannon, G. E. (1999). High-altitude vegetational pattern on the Iberian Mountain Chain (north-central Spain) during the Holocene. *The Holocene*, *9*(1), 39–57. <https://doi.org/10.1191/095968399671230625>

Goñi, S., & Fernanda, M. (1995). Analyse palynologique de l’Etang d’Ouveillan. In *Temps et espace da ns le bassin de lAude du Neolithique a lAge du Fer* (pp. 265–275). CNRS.

González-Ramón, A., Andreo, B., Ruiz-Bustos, A., Richards, D. A., López-Sáez, J. A., & Alba-Sánchez, F. (2012). Late Quaternary paleoenvironmental record from a sedimentary fill in Cucú cave, Almería, SE Spain. *Quaternary Research*, *77*(2), 264–272. <https://doi.org/https://doi.org/10.1016/j.yqres.2011.12.001>

González-Sampériz, P., Gil-Romera, G., García-Prieto, E., Aranbarri, J., Moreno, A., Morellón, M., Sevilla-Callejo, M., Leunda, M., Santos, L., Franco-Múgica, F., Andrade, A., Carrión, J. S., & Valero-Garcés, B. L. (2020). Strong continentality and effective moisture drove unforeseen vegetation dynamics since the last interglacial at inland Mediterranean areas: The Villarquemado sequence in NE Iberia. *Quaternary Science Reviews*, *242*, 106425. <https://doi.org/10.1016/j.quascirev.2020.106425>

Grüger, E. (1996). Vegetation change. In and S. B. J. Chapman, R. Shiel (Ed.), *The changing face of Dalmatia. Archaeological and ecological investigations in a Mediterranean Landscape* (pp. 33–44). Leicester University Press,.

Grüger, J. (1968). Untersuchungen zur spätglazialen und frühpostglazialen Vegetationsentwicklung der Südalpen im Umkreis des Gardassees. *Botanische Jahrbücher Für Systematik Pflanzengeschichte Und Pflanzengeographie*, *88*, 163–199.

Guiter, F., Andrieu-Ponel, V., Digerfeldt, G., Reille, M., de Beaulieu, jacques-L., & Ponel, P. (2005). Vegetation history and lake-level changes from the Younger Dryas to the present in Eastern Pyrenees (France): Pollen, plant macrofossils and lithostratigraphy from Lake Racou (2000 m a.s.l.). *Vegetation History and Archaeobotany*, *14*, 99–118. <https://doi.org/10.1007/s00334-005-0065-z>

Hamdan, M. A., R. J. Flower, F. A. Hassan, and S. A. G. Leroy. 2020. “Geochemical and Palynological Analysis of Faiyum Lake Sediments, Egypt: Implications for Holocene Paleoclimate.” Journal of African Earth Sciences 167:103864. doi: <https://doi.org/10.1016/j.jafrearsci.2020.103864>.

Hajar, L., Haïdar-Boustani, M., Khater, C., & Cheddadi, R. (2010). Environmental changes in Lebanon during the Holocene: Man vs. climate impacts. Journal of Arid Environments, 74(7), 746–755. <https://doi.org/10.1016/j.jaridenv.2008.11.002>

Huntley, B., Watts, W. A., Allen, J. R. M., & Zolitschka, B. (1999). Palaeoclimate, chronology and vegetation history of the Weichselian Lateglacial: comparative analysis of data from three cores at Lago Grande di Monticchio, southern Italy. *Quaternary Science Reviews*, *18*(7), 945–960. <https://doi.org/10.1016/S0277-3791(99)00007-4>

Hooghiemstra, H., Stalling, H., Agwu, C. O. C., & Dupont, L. M. (1992). Vegetational and climatic changes at the northern fringe of the sahara 250,000–5000 years BP: evidence from 4 marine pollen records located between Portugal and the Canary Islands. *Review of Palaeobotany and Palynology*, *74*(1), 1–53. <https://doi.org/10.1016/0034-6667(92)90137-6>

Jahns, S. (1993). On the Holocene vegetation history of the Argive Plain (Peloponnese, southern Greece). *Vegetation History and Archaeobotany*, *2*(4), 187–203. <https://doi.org/10.1007/BF00198161>

Janssen, C., & Woldringh, R. (1981). A preliminary radiocarbon dated pollen sequence from the Serra da Estrela, Portugal. *Finisterra*, *16*(32 SE-Artigos). <https://doi.org/10.18055/Finis2176>

Joannin, S., Elisabetta, B., de Beaulieu, Jacques-L., Bernardo, L., Magny, M., Peyron, O., & Vannière, B. (2012). Pollen-based reconstruction of Holocene vegetation and climate in Southern Italy: the case of Lago di Trifoglietti. *Climate of the Past Discussions*, *8*, 2223–2279. <https://doi.org/10.5194/cpd-8-2223-2012>

Joly, C., & Visset, L. (2009). Evolution of vegetation landscapes since the Late Mesolithic on the French West Atlantic coast. *Review of Palaeobotany and Palynology*, *154*, 124–179. <https://doi.org/10.1016/j.revpalbo.2008.12.011>

Jouffroy-Bapicot, I., Pedrotta, T., Debret, M., Field, S., Sulpizio, R., Zanchetta, G., Sabatier, P., Roberts, N., Tinner, W., Walsh, K., & Vannière, B. (2021). Olive groves around the lake. A ten-thousand-year history of a Cretan landscape (Greece) reveals the dominant role of humans in making this Mediterranean ecosystem. *Quaternary Science Reviews*, *267*, 107072. <https://doi.org/10.1016/j.quascirev.2021.107072>

Jouffroy-Bapicot, I., Pulido, M., Baron, S., Galop, D., Monna, F., Lavoie, M., Ploquin, A., Petit, C., de Beaulieu, jacques-L., & Richard, H. (2007). Environmental impact of early palaeometallurgy: Pollen and geochemical analysis. *Vegetation History and Archaeobotany*, *16*, 251–258. <https://doi.org/10.1007/s00334-006-0039-9>

Kafetzidou, A., Fatourou, E., Panagiotopoulos, K., Marret, F., & Kouli, K. (2023). Vegetation Composition in a Typical Mediterranean Setting (Gulf of Corinth, Greece) during Successive Quaternary Climatic Cycles. In *Quaternary* (Vol. 6, Issue 2). <https://doi.org/10.3390/quat6020030>

Kelly, M. G., & Huntley, B. (1991). An 11 000-year record of vegetation and environment from Lago di Martignano, Latium, Italy. *Journal of Quaternary Science*, *6*(3), 209–224. <https://doi.org/https://doi.org/10.1002/jqs.3390060304>

Kuzucuoğlu, C., Dörfler, W., Kunesch, S., & Goupille, F. (2011). Mid- to late-Holocene climate change in central Turkey: The Tecer Lake record. *The Holocene*, *21*(1), 173–188. <https://doi.org/10.1177/0959683610384163>

Leroy, S. A. G., & Roiron, P. (1996). Latest Pliocene pollen and leaf floras from Bernasso palaeolake (Escandorgue Massif, Hérault, France). *Review of Palaeobotany and Palynology*, *94*(3), 295–328. <https://doi.org/10.1016/S0034-6667(96)00016-4>

Linstädter, J., & Kehl, M. (2012). The Holocene archaeological sequence and sedimentological processes at Ifri Oudadane, NE Morocco. *Journal of Archaeological Science*, *39*(10), 3306–3323. [https://doi.org/10.1016/j.jas.2012.05.025](https://doi.org/https://doi.org/10.1016/j.jas.2012.05.025)

Marchand, G., Tessier, M., & Bernard, J. (1999). Les occupations mésolithiques et néolithiques de la Fillauderie (Saint-Père-en-Retz, Loire-Atlantique) et la préhistoire récente de la basse vallée du Boivre. *Revue Archéologique De l’Ouest*, *16*(1), 39–65. <https://doi.org/10.3406/RAO.1999.1087>

Mariotti Lippi, M., Lippi, M. M., Guido, M., Menozzi, B. I., Bellini, C., & Montanari, C. (2007). The Massaciuccoli Holocene pollen sequence and the vegetation history of the coastal plains by the Mar Ligure (Tuscany and Liguria, Italy). *Vegetation History and Archaeobotany*, *16*(4), 267–277. <https://doi.org/10.1007/S00334-006-0090-6>

Mercuri, A M, Bandini Mazzanti, M., Florenzano, A., Montecchi, M. C., & Rattighieri, E. (2013). Olea, Juglans and Castanea: The OJC group as pollen evidence of the development of human-induced environments in the Italian peninsula. *Quaternary International*, *303*, 24–42. <https://doi.org/10.1016/j.quaint.2013.01.005>

Mercuri, A., Accorsi, C. & Bandini Mazzanti, M (2002). The long history of Cannabis and its cultivation by the Romans in central Italy, shown by pollen records from Lago Albano and Lago di Nemi. *Vegetation History and Archaeobotany*, *11*(4), 263–276. <https://doi.org/10.1007/s003340200039>

Miras, Y., Millet, L., Guiter, F., Ponel, P., Beaulieu, J. L. de, & Goslar, T. (2006). Dynamique des écosystèmes et impact de l’homme dans le secteur du col du Petit Saint Bernard au cours de l’Holocène. Actes du Programme Interreg “Alpis Graïa.” *Aoste*, 31–50.

Miras, Y, Ejarque, A., Orengo, H., Riera, S., Martínez, J. M., & Alexandre, P. (2010). Prehistoric impact on landscape and vegetation at high altitudes: An integrated palaeoecological and archaeological approach in the eastern Pyrenees (Perafita valley, Andorra). *Plant Biosystems*, *144*. <https://doi.org/10.1080/11263504.2010.491980>

Morales-Molino, C., Steffen, M., Samartin, S., van Leeuwen, J. F. N., Hürlimann, D., Vescovi, E., & Tinner, W. (2021). Long-Term Responses of Mediterranean Mountain Forests to Climate Change, Fire and Human Activities in the Northern Apennines (Italy). *Ecosystems (New York, N.Y.)*, *24*(6), 1361–1377. <https://doi.org/10.1007/s10021-020-00587-4>

Morales-Molino, C., & García-Antón, M. (2014). Vegetation and fire history since the last glacial maximum in an inland area of the western Mediterranean Basin (Northern Iberian Plateau, NW Spain). *Quaternary Research*, *81*(1), 63–77. <https://doi.org/10.1016/j.yqres.2013.10.010>

Morales-Molino, C., García-Antón, M., Postigo-Mijarra, J. M., & Morla, C. (2013). Holocene vegetation, fire and climate interactions on the westernmost fringe of the Mediterranean Basin. *Quaternary Science Reviews*, *59*, 5–17. <https://doi.org/10.1016/j.quascirev.2012.10.027>

Morales-Molino, C., Tinner, W., García-Antón, M., & Colombaroli, D. (2017). The historical demise of Pinus nigra forests in the Northern Iberian Plateau (south-western Europe). *Journal of Ecology*, *105*(3), 634–646. <https://doi.org/10.1111/1365-2745.12702>

Morales, J., Pérez-Jordà, G., Peña-Chocarro, L., Zapata, L., Ruíz-Alonso, M., López-Sáez, J. A., & Linstädter, J. (2013). The origins of agriculture in North-West Africa: macro-botanical remains from Epipalaeolithic and Early Neolithic levels of Ifri Oudadane (Morocco). *Journal of Archaeological Science*, *40*(6), 2659–2669. <https://doi.org/10.1016/j.jas.2013.01.026>

Muller, S. D., Miramont, C., Bruneton, H., Carré, M., Sottocornola, M., Court-Picon, M., de Beaulieu, J.-L., Nakagawa, T., & Schevin, P. (2012). A palaeoecological perspective for the conservation and restoration of wetland plant communities in the central French Alps, with particular emphasis on alder carr vegetation. *Review of Palaeobotany and Palynology*, *171*, 124–139. <https://doi.org/10.1016/j.revpalbo.2011.12.005>

Nakagawa, T. (1998). Etudes palynologiques dans les Alpes Françaises centrales et méridionales: histoire de la végétation tardiglaciaire et holocène. PhD Thesis, Université de Droit dEconomie et des Sciences dAix-Marseille III, France, 206 pp

Nicol-Pichard, S., & Dubar, M. (1998). Reconstruction of Late-glacial and Holocene environments in southeast France based on the study of a 66-m long core from Biot, Alpes Maritimes. *Vegetation History and Archaeobotany*, *7*(1), 11–15. <https://doi.org/10.1007/BF01393413>

Noti, R., Leeuwen, J., Colombaroli, D., Vescovi, E., Salvatore, P., La Mantia, T., & Tinner, W. (2009). Mid- and late-Holocene vegetation and fire history at Biviere di Gela, a coastal lake in southern Sicily, Italy. *Vegetation History and Archaeobotany*, *18*, 371–387. <https://doi.org/10.1007/s00334-009-0211-0>

Nourelbait, M., Rhoujjati, A., Benkaddour, A., Carré, M., Eynaud, F., Martinez, P., & Cheddadi, R. (2016). Climate change and ecosystems dynamics over the last 6000 years in the Middle Atlas, Morocco. *Climate of the Past*, *12*, 1029–1042. <https://doi.org/10.5194/cp-12-1029-2016>

Nowaczyk, N. R., Arz, H. W., Frank, U., Kind, J., & Plessen, B. (2012). Dynamics of the Laschamp geomagnetic excursion from Black Sea sediments. *Earth and Planetary Science Letters*, *351*–*352*, 54–69. <https://doi.org/10.1016/j.epsl.2012.06.050>

Okuda, M., Yasuda, Y., & Setoguchi, T. (2001). Middle to Late Pleistocene vegetation history and climatic changes at Lake Kopais, Southeast Greece. *Boreas*, *30*(1), 73–82. <https://doi.org/10.1111/j.1502-3885.2001.tb00990.x>

Ortu, E., Peyron, O., Bordon, A., de Beaulieu, Jacques-L., Siniscalco, C., & Caramiello, R. (2008). Lateglacial and Holocene climate oscillations in the South-western Alps: An attempt at quantitative reconstruction. *Quaternary International - QUATERN INT*, *190*, 71–88. <https://doi.org/10.1016/j.quaint.2008.04.004>

Ouguerram, A., & Visset, L. (2001). Histoire de la végétation et première mise en évidence d’un milieu marin pendant l’HoIocène dans la vallée de l’Erdre et le Val de Gesvres (Bassin versant de la Loire, Massif armoricain, France). *Quaternaire*, *12*(3), 189–199.

Pantaléon-Cano, J., Yll, E. I., Pérez-Obiol, R., & Roure, J. M. (2003). Palynological evidence for vegetational history in semi-arid areas of the western Mediterranean (Almería, Spain). *Holocene*, *13*(1), 109–119. <https://doi.org/10.1191/0959683603hl598rp>

Peñalba, M. C., Arnold, M., Guiot, J., Duplessy, J.-C., & de Beaulieu, J.-L. (1997). Termination of the Last Glaciation in the Iberian Peninsula Inferred from the Pollen Sequence of Quintanar de la Sierra. *Quaternary Research*, *48*(2), 205–214. <https://doi.org/10.1006/qres.1997.1922>

Penalba, M.C. (1989). Dynamique de vegetation Tardiglaciaire et Holocene du centre-nord de l'Espagne d'après l'analyse pollinique. PhD Thesis, Université dAix-Marseille, Marseille, France

Penalba, M.C.. (1994). The History of the Holocene Vegetation in Northern Spain from Pollen Analysis. *Journal of Ecology*, *82*(4), 815–832. <https://doi.org/10.2307/2261446>

Pèrez-Obiol, R., & Julià, R. (1994). Climatic Change on the Iberian Peninsula Recorded in a 30,000-Yr Pollen Record from Lake Banyoles. *Quaternary Research*, *41*(1), 91–98. <https://doi.org/10.1006/qres.1994.1010>

Pini, R., Ravazzi, C., & Donegana, M. (2009). Pollen stratigraphy, vegetation and climate history of the last 215ka in the Azzano Decimo core (plain of Friuli, north-eastern Italy). *Quaternary Science Reviews*, *28*(13), 1268–1290. <https://doi.org/10.1016/j.quascirev.2008.12.017>

Piqué, R., Revelles, J., Burjachs, F., Caruso Fermé, L., & Pérez-Obiol, R. (2018). Interdisciplinary approach to the landscape and firewood exploitation during the Holocene at La Garrotxa (Girona, NE Iberia). *Quaternary International*, *463*, 401–413. <https://doi.org/10.1016/j.quaint.2016.11.025>

Pons, A., & Reille, M. (1988). The holocene- and upper pleistocene pollen record from Padul (Granada, Spain): A new study. *Palaeogeography, Palaeoclimatology, Palaeoecology*, *66*(3), 243–263. <https://doi.org/10.1016/0031-0182(88)90202-7>

Pulido, M. (2006). *Conséquenses de l’anthropisation sur la dynamique postglaciaire de la végétation dans le sud du Massif Central, France*. Université d’Aix-Marseille, Marseille, France.

Reille, M., & de Beaulieu, J. L. (1988). History of the Würm and Holocene vegetation in western velay (Massif Central, France): A comparison of pollen analysis from three corings at Lac du Bouchet. *Review of Palaeobotany and Palynology*, *54*(3), 233–248. <https://doi.org/10.1016/0034-6667(88)90016-4>

Reille, M., & de Beaulieu, J. L. (1990). Pollen analysis of a long upper Pleistocene continental sequence in a Velay maar (Massif Central, France). *Palaeogeography, Palaeoclimatology, Palaeoecology*, *80*(1), 35–48. <https://doi.org/10.1016/0031-0182(90)90032-3>

Revelles, J., Burjachs, F., Palomo, A., Piqué, R., Iriarte, E., Pérez-Obiol, R., & Terradas, X. (2018). Human-environment interaction during the Mesolithic- Neolithic transition in the NE Iberian Peninsula. Vegetation history, climate change and human impact during the Early-Middle Holocene in the Eastern Pre-Pyrenees. *Quaternary Science Reviews*, *184*, 183–200. <https://doi.org/10.1016/j.quascirev.2017.08.025>

Revelles, J., Ghilardi, M., Rossi, V., Currás, A., López-Bultó, O., Brkojewitsch, G., & Vacchi, M. (2019). Coastal landscape evolution of Corsica island (W. Mediterranean): palaeoenvironments, vegetation history and human impacts since the early Neolithic period. *Quaternary Science Reviews*, *225*, 105993. <https://doi.org/10.1016/j.quascirev.2019.105993>

Rösch, M., E. Fischer, Lechterbeck, J., & Wick., L. (2012). Pollenanalysen an drei Bohrkernen aus dem Profundal des Ortasees (Piemont, Italien). In A. S. and U. Tegtmeier (Ed.), *Verzweigungen, eine Würdigung für A.J. Kalis und J. Meurers- Balke.* (pp. 225–247). Frankfurter Archäologische Schriften.

Roucoux, K. H., de Abreu, L., Shackleton, N. J., & Tzedakis, P. C. (2005). The response of NW Iberian vegetation to North Atlantic climate oscillations during the last 65kyr. *Quaternary Science Reviews*, *24*(14), 1637–1653. <https://doi.org/10.1016/j.quascirev.2004.08.022>

Sadori, L., Koutsodendris, A., Panagiotopoulos, K., Masi, A., Bertini, A., Combourieu-Nebout, N., Francke, A., Kouli, K., Kousis, I., Joannin, S., Maria Mercuri, A., Peyron, O., Torri, P., Wagner, B., Zanchetta, G., Sinopoli, G., & Donders, T. H. (2016). Pollen-based paleoenvironmental and paleoclimatic change at Lake Ohrid (south-eastern Europe) during the past 500 ka. *Biogeosciences*, *13*(5), 1423–1427. <https://doi.org/10.5194/bg-13-1423-2016-corrigendum>

Samartin, S., Heiri, O., Joos, F., Renssen, H., Franke, J., Brönnimann, S., & Tinner, W. (2017). Warm Mediterranean mid-Holocene summers inferred from fossil midge assemblages. *Nature Geoscience*, *10*(3), 207–212. <https://doi.org/10.1038/ngeo2891>

Sánchez Goñi, M. F., Eynaud, F., Turon, J. L., & Shackleton, N. J. (1999). High resolution palynological record off the Iberian margin: Direct land-sea correlation for the Last Interglacial complex. *Earth and Planetary Science Letters*, *171*(1), 123–137. <https://doi.org/10.1016/S0012-821X(99)00141-7>

Sanchez Goñi, M., Bakker, P., Desprat, S., Carlson, A., Van Meerbeeck, C., Peyron, O., Naughton, F., Fletcher, W., Eynaud, F., Rossignol, L., & Renssen, H. (2012). European climate optimum and enhanced Greenland melt during the Last Interglacial. *Geology*, *40*, 627–630. <https://doi.org/10.1130/G32908.1>

Schönfeld, J., Zahn, R., & Abreu, L. (2003). Surface and deep water response to rapid climate changes at the Western Iberian Margin. *Global and Planetary Change*, *36*, 237–264. <https://doi.org/10.1016/S0921-8181(02)00197-2>

Shumilovskikh, L., Fleitmann, D., Nowaczyk, N., Behling, H., Marret, F., Wegwerth, A., & Arz, H. (2014). Orbital- and millennial-scale environmental changes between 64 and 20 ka BP recorded in Black Sea sediments. *Climate of the Past*, *10*, 939–954. <https://doi.org/10.5194/cp-10-939-2014>

Shumilovskikh, L. S., Tarasov, P., Arz, H. W., Fleitmann, D., Marret, F., Nowaczyk, N., Plessen, B., Schlütz, F., & Behling, H. (2012). Vegetation and environmental dynamics in the southern Black Sea region since 18kyr BP derived from the marine core 22-GC3. *Palaeogeography, Palaeoclimatology, Palaeoecology*, *337*–*338*, 177–193. <https://doi.org/10.1016/j.palaeo.2012.04.015>

Tastet, J.-P., & Diot, M.-F. (1995). Paléo-environnements holocènes et limites chronoclimatiques enregistrés dans un marais estuarien de la Gironde (France) [ Holocene paleo-environments and chrono-climatic limits recorded in a reclaimed marsh of the Gironde estuary (France).]. *Quaternaire*, 63–75.

Thouveny, N., de Beaulieu, J.-L., Bonifay, E., Creer, K. M., Guiot, J., Icole, M., Johnsen, S., Jouzel, J., Reille, M., Williams, T., & Williamson, D. (1994). Climate variations in Europe over the past 140 kyr deduced from rock magnetism. *Nature*, *371*(6497), 503–506. <https://doi.org/10.1038/371503a0>

Tinner, W., Hubschmid, P., Wehrli, M., Ammann, B., & Conedera, M. (1999). Long-term forest fire ecology and dynamics in southern Switzerland. *Journal of Ecology*, *87*(2), 273–289. <https://doi.org/10.1046/j.1365-2745.1999.00346.x>

Tinner, W., Leeuwen, J., Colombaroli, D., Vescovi, E., Knaap, W. O., Henne, P., Salvatore, P., D’Angelo, S., & La Mantia, T. (2009). Holocene environmental and climatic changes at Gorgo Basso, a coastal lake in southern Sicily, Italy. *Quaternary Science Reviews*, *28*, 1498–1510. <https://doi.org/10.1016/j.quascirev.2009.02.001>

Tinner, W., Vescovi, E., Leeuwen, J., Colombaroli, D., Henne, P., Kaltenrieder, P., Morales-Molino, C., Beffa, G., Gnägi, B., Knaap, W., La Mantia, T., & Salvatore, P. (2016). Holocene vegetation and fire history of the mountains of Northern Sicily (Italy). *Vegetation History and Archaeobotany*, *25*. <https://doi.org/10.1007/s00334-016-0569-8>

Turner, J., & Greig, J. R. A. (1975). Some Holocene pollen diagrams from Greece. *Review of Palaeobotany and Palynology*, *20*(3), 171–204. <https://doi.org/10.1016/0034-6667(75)90020-2>

van den Brink, L. M., & Janssen, C. R. (1985). The effect of human activities during cultural phases on the development of montane vegetation in the Serra de Estrela, Portugal. *Review of Palaeobotany and Palynology*, *44*(3), 193–215. <https://doi.org/10.1016/0034-6667(85)90016-8>

van der Knaap, W. O., & van Leeuwen, J. F. N. (1995). Holocene vegetation succession and degradation as responses to climatic change and human activity in the Serra de Estrela, Portugal. *Review of Palaeobotany and Palynology*, *89*(3), 153–211. <https://doi.org/10.1016/0034-6667(95)00048-0>

van der Knaap, W. O., & van Leeuwen, J. F. N. (1997). Late Glacial and early Holocene vegetation succession, altitudinal vegetation zonation, and climatic change in the Serra da Estrela, Portugal. *Review of Palaeobotany and Palynology*, *97*(3), 239–285. <https://doi.org/10.1016/S0034-6667(97)00008-0>

Vannière, B., Colombaroli, D., Charpon, E., Leroux, A., Tinner, W., & Magny, M. (2008). Climate versus human-driven fire regimes in Mediterranean landscapes: the Holocene record of Lago dell’Accesa (Tuscany, Italy). *Quaternary Science Reviews*, *27*, 1181–1196. <https://doi.org/10.1016/j.quascirev.2008.02.011>

Vella, C., & Provansal, M. (2000). Relative sea-level rise and neotectonic events during the last 6500yr on the southern eastern Rhône delta, France. *Marine Geology*, *170*(1), 27–39. <https://doi.org/10.1016/S0025-3227(00)00063-3>

Vescovi, E., Ammann, B., Ravazzi, C., & Tinner, W. (2010). A new Late-glacial and Holocene record of vegetation and fire history from Lago del Greppo, northern Apennines, Italy. *Vegetation History and Archaeobotany*, *19*, 219–233. <https://doi.org/10.1007/s00334-010-0243-5>

Visset, L., & Bernard, J. (2006). Évolution du littoral et du paysage, de la presqu’île de Rhuys à la rivière d’Étel (Massif armoricain – France), du Néolithique au Moyen Âge. *ArcheoSciences*, *30*, 143–156.

Visset, L., Hauray, G., Charrieau, L., & Rouzeau, N. (2001). Paléoenvironnement urbain : Histoire du comblement des vallées de la métropole nantaise, du Tardiglaciaire à la fin de l’Holocène. *Annales de Bretagne et Des Pays de L’Ouest*, *108*, 147–165.

Visset, L., L’helgouach, J., & Bernard, J. (1996). La tourbière submergée de la pointe de Kerpenhir à Locmariaquer (Morbihan). *Etude Environnementale et Mise En Évidence de Déforestations et de Pratiques Agricoles Néolithiques.*

von Engelbrechten, S. (1998). *Late-glacial and Holocene Vegetation and Environmental History of the Sierra de Urbion, North-Central Spain*. Doctoral dissertation. University of Dublin.

Watts, W. A., Allen, J. R. M., & Huntley, B. (1996). Vegetation history and palaeoclimate of the last glacial period at Lago Grande di Monticchio, Southern Italy. *Quaternary Science Reviews*, *15*(2), 133–153. <https://doi.org/10.1016/0277-3791(95)00093-3>

Yll, E.-I., Perez-Obiol, R., Pantaleon-Cano, J., & Roure, J. M. (1997). Palynological Evidence for Climatic Change and Human Activity during the Holocene on Minorca (Balearic Islands). *Quaternary Research*, *48*(3), 339–347. <https://doi.org/10.1006/qres.1997.1925>

Yll, E. I., Pérez‐Obiol, R., & Julià, R. (1994). Vegetational change in the Balearic Islands (Spain) during the Holocene. *Historical Biology*, *9*(1–2), 83–89. <https://doi.org/10.1080/10292389409380490>

Zapata, L., López-Sáez, J. A., Ruiz-Alonso, M., Linstädter, J., Pérez-Jordà, G., Morales, J., Kehl, M., & Peña-Chocarro, L. (2013). Holocene environmental change and human impact in NE Morocco: Palaeobotanical evidence from Ifri Oudadane. *The Holocene*, *23*(9), 1286–1296. <https://doi.org/10.1177/0959683613486944>
