# Supplementary material for: Distribution patterns of Quercus ilex from the last interglacial period to the future by ecological niche modeling
Source: Ecol Evol. 2023 Oct 19;13(10):e10606. doi: 10.1002/ece3.10606 (PMC10585444; doi:10.1002/ece3.10606)
Supplement: Supplementary file 8 — Table S6. [file ECE3-13-e10606-s001.docx]

**S6 Table.** Areas spatial cell numbers of habitat loss, habitat gain and habitat stability over time under future conditions, for each time period using CCSM4 climate model.

| **Models** | **Year** | **Scenario** | **Stable absent** | **Stable presence** | **Habitat Loss** | **Habitat Loss (%)** | **Habitat Gain** | **Habitat Gain (%)** | **Species Range Change (%)** |
| --- | --- | --- | --- | --- | --- | --- | --- | --- | --- |
| ANN | 2050 | RCP4.5 | 545030 | 119247 | 6436 | 5 | 37248 | 30 | 25 |
|  |  | RCP8.5 | 519257 | 114600 | 7516 | 6 | 66588 | 55 | 48 |
|  | 2070 | RCP4.5 | 536532 | 116313 | 9370 | 7 | 45746 | 36 | 29 |
|  |  | RCP8.5 | 506823 | 116076 | 9607 | 8 | 75455 | 60 | 52 |
| CTA | 2050 | RCP4.5 | 542762 | 115151 | 6965 | 6 | 43083 | 35 | 30 |
|  |  | RCP8.5 | 519257 | 114600 | 7516 | 6 | 66588 | 55 | 48 |
|  | 2070 | RCP4.5 | 524737 | 113511 | 8605 | 7 | 61108 | 50 | 43 |
|  |  | RCP8.5 | 483911 | 111556 | 10560 | 9 | 101934 | 83 | 75 |
| FDA | 2050 | RCP4.5 | 546715 | 116426 | 7795 | 6 | 37025 | 30 | 24 |
|  |  | RCP8.5 | 532463 | 116145 | 8076 | 7 | 51277 | 41 | 35 |
|  | 2070 | RCP4.5 | 536468 | 117091 | 7130 | 6 | 47272 | 38 | 32 |
|  |  | RCP8.5 | 505919 | 112465 | 11756 | 9 | 77821 | 63 | 53 |
| GAM | 2050 | RCP4.5 | 564784 | 100893 | 13184 | 12 | 29100 | 26 | 14 |
|  |  | RCP8.5 | 551222 | 95578 | 18499 | 16 | 42662 | 37 | 21 |
|  | 2070 | RCP4.5 | 554135 | 95758 | 18319 | 16 | 39749 | 35 | 19 |
|  |  | RCP8.5 | 527204 | 84892 | 29185 | 26 | 66680 | 58 | 33 |
| GBM | 2050 | RCP4.5 | 567705 | 98475 | 13707 | 12 | 28074 | 25 | 13 |
|  |  | RCP8.5 | 554358 | 92922 | 19260 | 17 | 41421 | 37 | 20 |
|  | 2070 | RCP4.5 | 557340 | 93078 | 19104 | 17 | 38439 | 34 | 17 |
|  |  | RCP8.5 | 530529 | 81697 | 30485 | 27 | 65250 | 58 | 31 |
| GLM | 2050 | RCP4.5 | 555140 | 93872 | 24618 | 21 | 34331 | 29 | 8 |
|  |  | RCP8.5 | 542159 | 83957 | 34533 | 29 | 47312 | 40 | 11 |
|  | 2070 | RCP4.5 | 545310 | 87569 | 30921 | 26 | 44161 | 37 | 11 |
|  |  | RCP8.5 | 522307 | 68512 | 49978 | 42 | 67164 | 57 | 15 |
| MARS | 2050 | RCP4.5 | 558650 | 103026 | 11699 | 10 | 34586 | 30 | 20 |
|  |  | RCP8.5 | 543925 | 99925 | 14800 | 13 | 49311 | 43 | 30 |
|  | 2070 | RCP4.5 | 546874 | 99195 | 15530 | 14 | 46362 | 40 | 27 |
|  |  | RCP8.5 | 519091 | 91682 | 23043 | 20 | 74145 | 65 | 45 |
| MAXENT | 2050 | RCP4.5 | 558036 | 100981 | 12319 | 11 | 36625 | 32 | 21 |
|  |  | RCP8.5 | 542272 | 100688 | 12612 | 11 | 52389 | 46 | 35 |
|  | 2070 | RCP4.5 | 542825 | 99229 | 14071 | 12 | 51836 | 46 | 33 |
|  |  | RCP8.5 | 520231 | 94688 | 18612 | 16 | 74430 | 66 | 49 |
| RF | 2050 | RCP4.5 | 570080 | 90364 | 14921 | 14 | 32596 | 31 | 17 |
|  |  | RCP8.5 | 551219 | 85157 | 20128 | 19 | 51457 | 49 | 30 |
|  | 2070 | RCP4.5 | 553769 | 86022 | 19263 | 18 | 48907 | 46 | 28 |
|  |  | RCP8.5 | 527176 | 77219 | 28066 | 27 | 75500 | 72 | 45 |
| SRE | 2050 | RCP4.5 | 612648 | 46838 | 26878 | 36 | 21597 | 29 | -7 |
|  |  | RCP8.5 | 599873 | 40207 | 33509 | 45 | 34372 | 47 | 1 |
|  | 2070 | RCP4.5 | 605157 | 41287 | 32429 | 44 | 29088 | 39 | -5 |
|  |  | RCP8.5 | 586595 | 30079 | 43637 | 59 | 47650 | 65 | 5 |
