# Supplementary material for: Distribution patterns of Quercus ilex from the last interglacial period to the future by ecological niche modeling
Source: Ecol Evol. 2023 Oct 19;13(10):e10606. doi: 10.1002/ece3.10606 (PMC10585444; doi:10.1002/ece3.10606)
Supplement: Supplementary file 9 — Table S7. [file ECE3-13-e10606-s009.docx]

**S7 Table.** Areas spatial cell numbers of habitat loss, habitat gain and habitat stability over time under future conditions, for each time period using MIROC-ESM climate model.

| **Models** | **Year** | **Scenario** | **Stable absent** | **Stable presence** | **Habitat Loss** | **Habitat Loss (%)** | **Habitat Gain** | **Habitat Gain (%)** | **Species Range Change (%)** |  |
| --- | --- | --- | --- | --- | --- | --- | --- | --- | --- | --- |
|  |  |  |  |  |  |  |  |  |  |  |
| ANN | 2050 | RCP4.5 | 527537 | 124150 | 1533 | 1 | 54741 | 44 | 42 |  |
|  |  | RCP8.5 | 456909 | 115463 | 6653 | 5 | 128936 | 106 | 100 |  |
|  | 2070 | RCP4.5 | 516974 | 120841 | 4842 | 4 | 65304 | 52 | 48 |  |
|  |  | RCP8.5 | 485961 | 110626 | 15057 | 12 | 96317 | 77 | 65 |  |
| CTA | 2050 | RCP4.5 | 490888 | 118741 | 3375 | 3 | 94957 | 78 | 75 |  |
|  |  | RCP8.5 | 456909 | 115463 | 6653 | 5 | 128936 | 106 | 100 |  |
|  | 2070 | RCP4.5 | 465175 | 116810 | 5306 | 4 | 120670 | 99 | 94 |  |
|  |  | RCP8.5 | 409707 | 108377 | 13739 | 11 | 176138 | 144 | 133 |  |
| FDA | 2050 | RCP4.5 | 510457 | 111490 | 12731 | 10 | 73283 | 59 | 49 |  |
|  |  | RCP8.5 | 489425 | 104132 | 20089 | 16 | 94315 | 76 | 60 |  |
|  | 2070 | RCP4.5 | 499481 | 106081 | 18140 | 15 | 84259 | 68 | 53 |  |
|  |  | RCP8.5 | 464531 | 91975 | 32246 | 26 | 119209 | 96 | 70 |  |
| GAM | 2050 | RCP4.5 | 537747 | 87318 | 26759 | 23 | 56137 | 49 | 26 |  |
|  |  | RCP8.5 | 520934 | 75391 | 38686 | 34 | 72950 | 64 | 30 |  |
|  | 2070 | RCP4.5 | 523310 | 68941 | 45136 | 40 | 70574 | 62 | 22 |  |
|  |  | RCP8.5 | 486987 | 57796 | 56281 | 49 | 106897 | 94 | 44 |  |
| GBM | 2050 | RCP4.5 | 541135 | 84639 | 27543 | 25 | 54644 | 49 | 24 |  |
|  |  | RCP8.5 | 524922 | 72551 | 39631 | 35 | 70857 | 63 | 28 |  |
|  | 2070 | RCP4.5 | 527076 | 66185 | 45997 | 41 | 68703 | 61 | 20 |  |
|  |  | RCP8.5 | 492899 | 55238 | 56944 | 51 | 102880 | 92 | 41 |  |
| GLM | 2050 | RCP4.5 | 535769 | 76558 | 41932 | 35 | 53702 | 45 | 10 |  |
|  |  | RCP8.5 | 531012 | 63874 | 54616 | 46 | 58459 | 49 | 3 |  |
|  | 2070 | RCP4.5 | 529946 | 61916 | 56574 | 48 | 59525 | 50 | 2 |  |
|  |  | RCP8.5 | 527602 | 45452 | 73038 | 62 | 61869 | 52 | -9 |  |
| MARS | 2050 | RCP4.5 | 538167 | 92851 | 21874 | 19 | 55069 | 48 | 29 |  |
|  |  | RCP8.5 | 525964 | 81994 | 32731 | 29 | 67272 | 59 | 30 |  |
|  | 2070 | RCP4.5 | 528177 | 80006 | 34719 | 30 | 65059 | 57 | 26 |  |
|  |  | RCP8.5 | 511406 | 62711 | 52014 | 45 | 81830 | 71 | 26 |  |
| MAXENT | 2050 | RCP4.5 | 513560 | 98352 | 14948 | 13 | 81101 | 72 | 58 |  |
|  |  | RCP8.5 | 496547 | 90412 | 22888 | 20 | 98114 | 87 | 66 |  |
|  | 2070 | RCP4.5 | 502600 | 88829 | 24471 | 22 | 92061 | 81 | 60 |  |
|  |  | RCP8.5 | 467709 | 71750 | 41550 | 37 | 126952 | 112 | 75 |  |
| RF | 2050 | RCP4.5 | 535664 | 88418 | 16867 | 16 | 67012 | 64 | 48 |  |
|  |  | RCP8.5 | 516889 | 78862 | 26423 | 25 | 85787 | 81 | 56 |  |
|  | 2070 | RCP4.5 | 524816 | 76781 | 28504 | 27 | 77860 | 74 | 47 |  |
|  |  | RCP8.5 | 493575 | 64542 | 40743 | 39 | 109101 | 104 | 65 |  |
| SRE | 2050 | RCP4.5 | 613545 | 34787 | 38929 | 53 | 20700 | 28 | -25 |  |
|  |  | RCP8.5 | 611889 | 23112 | 50604 | 69 | 22356 | 30 | -38 |  |
|  | 2070 | RCP4.5 | 605169 | 23681 | 50035 | 68 | 29076 | 39 | -28 |  |
|  |  | RCP8.5 | 605838 | 15230 | 58486 | 79 | 28407 | 39 | -41 |  |
